# Historeceptomic Fingerprints for Drug-Like Compounds

**DOI:** 10.3389/fphys.2015.00371

**Published:** 2015-12-18

**Authors:** Evgeny Shmelkov, Arsen Grigoryan, James Swetnam, Junyang Xin, Doreen Tivon, Sergey V. Shmelkov, Timothy Cardozo

**Affiliations:** ^1^Department of Biochemistry and Molecular Pharmacology, New York University School of MedicineNew York, NY, USA; ^2^Google Inc., Mountain ViewCA, USA; ^3^GeneCentrix Inc.New York, NY, USA; ^4^Department of Neuroscience and Physiology, New York University School of MedicineNew York, NY, USA; ^5^Department of Psychiatry, New York University School of MedicineNew York, NY, USA

**Keywords:** polypharmacology, molecular docking simulation, gene expression, mechanism of drug action, drug target

## Abstract

Most drugs exert their beneficial and adverse effects through their combined action on several different molecular targets (polypharmacology). The true molecular fingerprint of the direct action of a drug has two components: the ensemble of all the receptors upon which a drug acts and their level of expression in organs/tissues. Conversely, the fingerprint of the adverse effects of a drug may derive from its action in bystander tissues. The ensemble of targets is almost always only partially known. Here we describe an approach improving upon and integrating both components: *in silico* identification of a more comprehensive ensemble of targets for any drug weighted by the expression of those receptors in relevant tissues. Our system combines more than 300,000 experimentally determined bioactivity values from the ChEMBL database and 4.2 billion molecular docking scores. We integrated these scores with gene expression data for human receptors across a panel of human tissues to produce drug-specific tissue-receptor (historeceptomics) scores. A statistical model was designed to identify significant scores, which define an improved fingerprint representing the unique activity of any drug. These multi-dimensional historeceptomic fingerprints describe, in a novel, intuitive, and easy to interpret style, the holistic, *in vivo* picture of the mechanism of any drug's action. Valuable applications in drug discovery and personalized medicine, including the identification of molecular signatures for drugs with polypharmacologic modes of action, detection of tissue-specific adverse effects of drugs, matching molecular signatures of a disease to drugs, target identification for bioactive compounds with unknown receptors, and hypothesis generation for drug/compound phenotypes may be enabled by this approach. The system has been deployed at drugable.org for access through a user-friendly web site.

## Introduction

**Figure 1:**
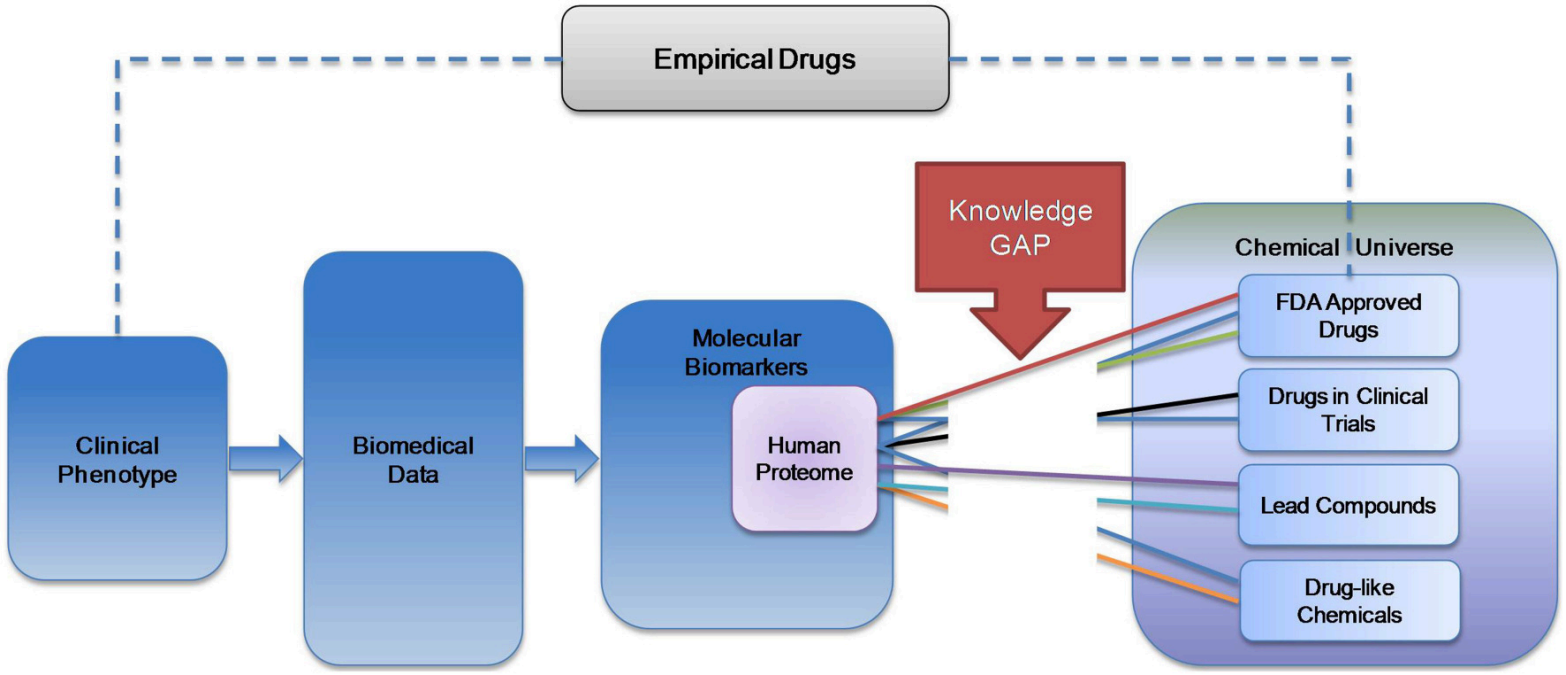
**Knowledge gap in the spectrum of public health information**. While the majority of drugs in clinical use were discovered empirically, high throughput omics technologies generate the basis for inferring targets for rational drug design. However, it remains unclear how to integrate large sets of omics data on potential drug targets with chemicals that may interact with these targets.

Enormous quantities of “omics” data characterizing both normal and diseased tissues continue to accumulate, leading to the development of increasingly complex molecular biomarkers for diseases. The majority of drugs in current clinical use were discovered by phenotypic screens, leaving their precise mechanism of action unknown. Many if not most of these drugs likely act polypharmacologically (on multiple receptors simultaneously). These two trends result in a growing knowledge gap between the efforts to mechanistically and genomically characterize diseases on the molecular level and the chemicals used for their treatment (Figure 1).

Polypharmacology partly addresses this gap and has gained increasing attention in the field of drug discovery (Peters, 2011). At least some approved drugs exhibit polypharmacological signatures by interacting with multiple targets(Ashburn and Thor, 2004; Keiser et al., 2007, 2009; Mestres et al., 2009; Durrant et al., 2010; Yang et al., 2011). The identification of more of the ensemble of these targets is essential for both understanding the mechanism of drug action and predicting toxicity (Cereto-Massagué et al., 2015). Moreover, the development of compounds that rationally interact with multiple targets is appealing in the case of complex multigenic diseases, such as cancer (Knight et al., 2010) or psychiatric disorders (Metz and Hajduk, 2010; Allen and Roth, 2011; Brown and Okuno, 2012). Improved polypharmacological profiles of a drug can be identified only by a more comprehensive analysis of drug-target interactions on a proteome-wide scale (Xie et al., 2012).

In recent years, growing databases of compound-receptor bioactivities have become available (Wang et al., 2009; Sharman et al., 2011; Gaulton et al., 2012). However, the complete universe of bioactivity scores between putative or actual drugs/compounds and their receptors is still far from approachable. A number of ligand-based and structure-based *in silico* approaches emerged to address the off-target identification aspect of this issue (Rognan, 2013). Ligand-based approaches are based on an assumption that chemically similar structures are more likely to have similar pharmacological profiles. The idea behind the structure-based off-target identification approaches is based on inverse docking (Chen and Zhi, 2001), where a single compound is docked to multiple targets and the potential biological targets are ranked based on the docking (Chen and Zhi, 2001; Paul et al., 2004; Gao et al., 2008; Yang et al., 2009; Durrant et al., 2010; Li et al., 2010a,b; Grinter et al., 2011).

The combination of *in silico* target identification methods and growing databases of experimental bioactivity scores improves the feasibility of using these methods to identify a significant subset of the complete ensemble of receptors for known drugs and drug-like compounds by computational approaches. However, a gap would still remain between the polypharmacology of a drug and its pharmacodynamics, i.e., the distribution of its receptor targets in the human body. In order for the affinity of a drug for a given receptor in a given tissue to be a significant factor, the receptor has to be expressed in this tissue. For example, no matter how high the affinity of LSD is for the serotonin 5-HT2a receptor (HTR2A), this drug-target interaction is not physiologically significant in uterine tissue as HTR2A is not expressed there. The true fingerprint of drug action is the totality (“omics”) of receptors for which a drug has affinity, weighted by the expression levels of these receptors in the tissues (“histos”) across human body. Hence we introduced the term “historeceptomic fingerprint” for the holistic signature of drug action. Thus, here, we aim to develop a novel approach for the identification of historeceptomic fingerprints for any given drug/compound.

## Methods

### Chemical library

Chemical structures in Drugable were obtained from three sources: DrugBank, PubChem, and ChEMBL. 1423 approved and 4752 experimental drugs were imported from DrugBank 2.5 via the XML format release. An additional 1,138,288 compounds were imported from the SDF format release of ChEMBL 14. Additionally, PubChem compound identifiers from the SDF release were assigned to 1,006,895 DrugBank or ChEMBL compounds in Drugable on the basis of equal canonical SMILES strings as computed from RDKit (Landrum, 2008). Overall 1,141,434 unique chemical structures are represented in Drugable.

### Compound-compound associations

Compound-compound associations were evaluated as a chemical similarity measure between two compounds and derived as Tanimoto distance between their molecular fingerprints as implemented in the RDKit PostgreSQL extension. Briefly, given a molecule, all linear and non-linear fragments of different size were enumerated and hashed into a bit string called a *fingerprint*. The *Tanimoto coefficient, T*, for two fingerprints was calculated as the number of bits in which they differ divided by the number of non-zero bits they have in common. The Tanimoto distance was defined as 1—*T*. Compounds are shown in the “Similar Compounds” section of a compound page if their Tanimoto distance is less than 0.5.

### Protein library

20,266 Human proteins were imported from the XML release of UniProt into drugable.org.

### Structure library

3D Structures for the human proteins imported as above were obtained from two sources, the Pocketome (Abagyan and Kufareva, 2009) and ModBase (Pieper et al., 2006). 6857 experimental structures come from the Pocketome and 64,801 homology models are available from ModBase.

Consideration of receptor flexibility is crucial for structure-based drug design and the conformational ensembles of protein receptors derived from Pocketome are a practical alternative to mimic receptor flexibility. However, blindly adding certain conformations to an ensemble may be counterproductive (Rueda et al., 2010). To ensure the high quality of selected conformers, we performed retrospective virtual screening experiments and only structures with high separation power of known ligand binders from decoys were selected. Initially, for a benchmark screen, pockets on Pocketome human proteins (Table 1) were screened against a custom chemical library consisting of compounds solved crystallographically with several proteins and 100 random chemical decoys in order to measure the docking quality of the pockets. Having established that only the highest quality pockets could produce accurate docking scores, a subset of 6857 high quality X-ray conformations of 570 human protein targets from Pocketome was imported into the data warehouse. The 4.2 billion scores generated for Pubchem Bioassay, ChEMBL, and DrugBank compounds against these 6857 high quality pockets on 570 protein targets from the Pocketome have been integrated into the drugable.org historeceptomics system. Where there are multiple conformations for a pocket, the best score was retained. An additional complete matrix of docking scores of 4313 unique chemotypes from drugbank against ModBase homology model database is available in raw form from the authors. As a complete matrix, this data can be used for routine mathematical transformations to study symmetries and trends in the data that relate to polypharmacology. In all, docking to the largest possible set of pockets representing the druggable human genome was evaluated in this study.

**Table 1:** **Assessment of docking performance**.

**Data set**	**No. of pockets**	**Mean AUC**
All	7553	0.57
All TP ≥ 5 and TN ≥ 5	6017	0.569
Homology only and TP ≥ 5 and TN ≥ 5	2128	0.528
Pocketome only and TP ≥ 5 and TN ≥ 5	3889	0.591

TP and TN are the numbers of positive and negative bioactivity values available for a given pocket on a protein. Since estimation of AUC for pockets with a very small number of bioactivity values may not be fair, we also provide estimates obtained on pockets with at least 5 positive and 5 negative bioactivity values.

### Pharmareceptomic (bioactivity or docking) scores

In order to score the probability of interaction of compounds to a comprehensive set of protein targets, we used the largest available set of experimentally obtained bioactivities and *in silico* predicted compound-protein docking associations.

### Source of *in vitro* binding data

1,062,908 experimental compound-protein binding affinity measurements were downloaded from ChEMBL 14 PostgreSQL release. We used only binding measurements annotated with a confidence score ≥7, “assay type” field of “B,” or direct protein-ligand binding, and “standard_type” field of “Kd,” “Ki,” or “Potency.” All compound-protein associations obtained from ChEMBL are linked to their original scientific publications in PubMed where data was available from ChEMBL.

### Source of *in silico* docking data

More than four billion compound-protein associations were derived from *in silico* docking experiments. The AutoDock docking program was used for the docking calculations and all the parameters were set to default values. AutoDock addresses the docking issue as a global optimization problem of an energy function, implementing an iterated local search global optimizer, using the Broyden-Fletcher-Goldfarb-Shanno criterion for local search (Trott and Olson, 2010).

Target Structure Preparation: The approach is intended to be proteome wide. Therefore, many targets with unknown biological function are expected to be available from structural genomics efforts for this approach. In order to simulate the realistic situation wherein the specific functional site on a new crystallographically resolved target receptor with unknown biological function is unknown, we rendered pockets on all receptors blindly based only on the structure coordinates and randomly selected one pocket per receptor. This pocket was then defined as the binding site for docking. Receptors were then set-up by deleting the chains, heteroatoms, and prosthetic groups not involved in the binding site definition using ICM Browser (Molsoft LLC, La Jolla CA). Protein atom types were assigned, and hydrogen atoms and missing heavy atoms were added. The added or zero occupancy side chains and polar hydrogen atoms were optimized and assigned the lowest energy. Tautomeric states of histidines and the rotations of asparagine and glutamine side chain amidic groups were optimized to improve the hydrogen-bonding patterns. The cognate ligands were deleted from the complexes only after hydrogen optimization. Following this receptor preparation, we used the prepare_receptor4.py script (a part of the AutoDock Tools distribution) with default settings to convert the PDB models produced by ICM to the native PDBQT format of AutoDock.

Ligand Structure Preparation: For each compound, bond orders, tautomeric forms, stereochemistry, hydrogen atoms, and protonation states were assigned automatically by the AutoDock chemical conversion procedure. Each ligand was assigned the modified X-Score force field atom types and charges implemented in Arg. Canonical SMILES of each ligand to be screened were matched to the appropriate PubChem 3D structure (Bolton et al., 2011) to be used as a starting conformation for AutoDock docking.

After each docking simulation a stack of diverse binding poses was generated, and their respective docking scores were evaluated using the AutoDock scoring function (Trott and Olson, 2010). Three docking runs were performed for each compound-pocket pair; all binding poses accumulated after each run were merged in a single conformational stack and ranked based on their binding scores; finally, the conformation with the best docking score was retained.

### Predicted pharmareceptomics score (probability) of compound-target interaction

In our approach, the pharmareceptomics score is equivalent to the estimated probability that the compound will interact with the target at a physiologically significant level. For experimental bioactivities, the pharmareceptomics score is set equal to experimental affinity. For docking scores, we used the relationship between binding affinity and docking score published in Husby et al. (2015) to estimate a pharmareceptomics score from a docking score.

### Protein target–gene expression associations

Gene expression patterns of protein targets from a diverse set of tissues and cell types were derived from the “GeneAtlas U133A, gcrma” dataset (Su et al., 2004) via the BioGPS web-tool (http://biogps.org/, accessed on 5/7/2013; Wu et al., 2009, 2013). If for a given gene, data from multiple probes/experiments were available, the mean of those values was used. For each target protein, the level of expression in each tissue was normalized with regard to its level of expression in all tissues of the dataset and projected into the Z-score.

### Data access

The system (“Drugable”) is accessible via user-friendly interface at http://drugable.org/. A flexible free-text search index is available for common names of compounds and targets, medical conditions, etc. Chemical drawer allows user to search by chemical similarity or substructure.

For example when searching Drugable by compound common name, the user is presented with compound chemical structure, compound information (Number of Hydrogen Bond Donors and Hydrogen Bond Acceptors, Number of Rotatable Bonds, Number of Rings, Walden-Crippen LogP, Indication, Pharmacology, Mechanism of Action etc.), and a table of compound-protein associations (experimentally derived and/or predicted by *in silico* docking experiments) available for this specific compound. The resulting table gives a list of protein targets of the compound of interest with reported or predicted affinity, including protein target UniProt accession ID, the measured activity value and type or docking score. Note that all the experimentally obtained activities are displayed in nM. In addition, a list of compounds that are chemically similar to the compound of interest is also presented. Furthermore, tissue-specific levels of expression for all genes, correspond to the protein targets of the compound of interest, are presented as a heat map.

Alternatively, a user may want to search for a particular protein of interest. In this case, the user is presented with details of the protein target, such as X-ray structure (if available), protein name synonyms, gene names, organism this protein belongs to, and UniProt accession ID.

Furthermore, users may search for a medical condition of interest. In this case user is presented with a list of drugs/drug-like compounds as well as protein targets associated with this medical condition.

## Results

### Generation of bioactivity scores

**Figure 2:**
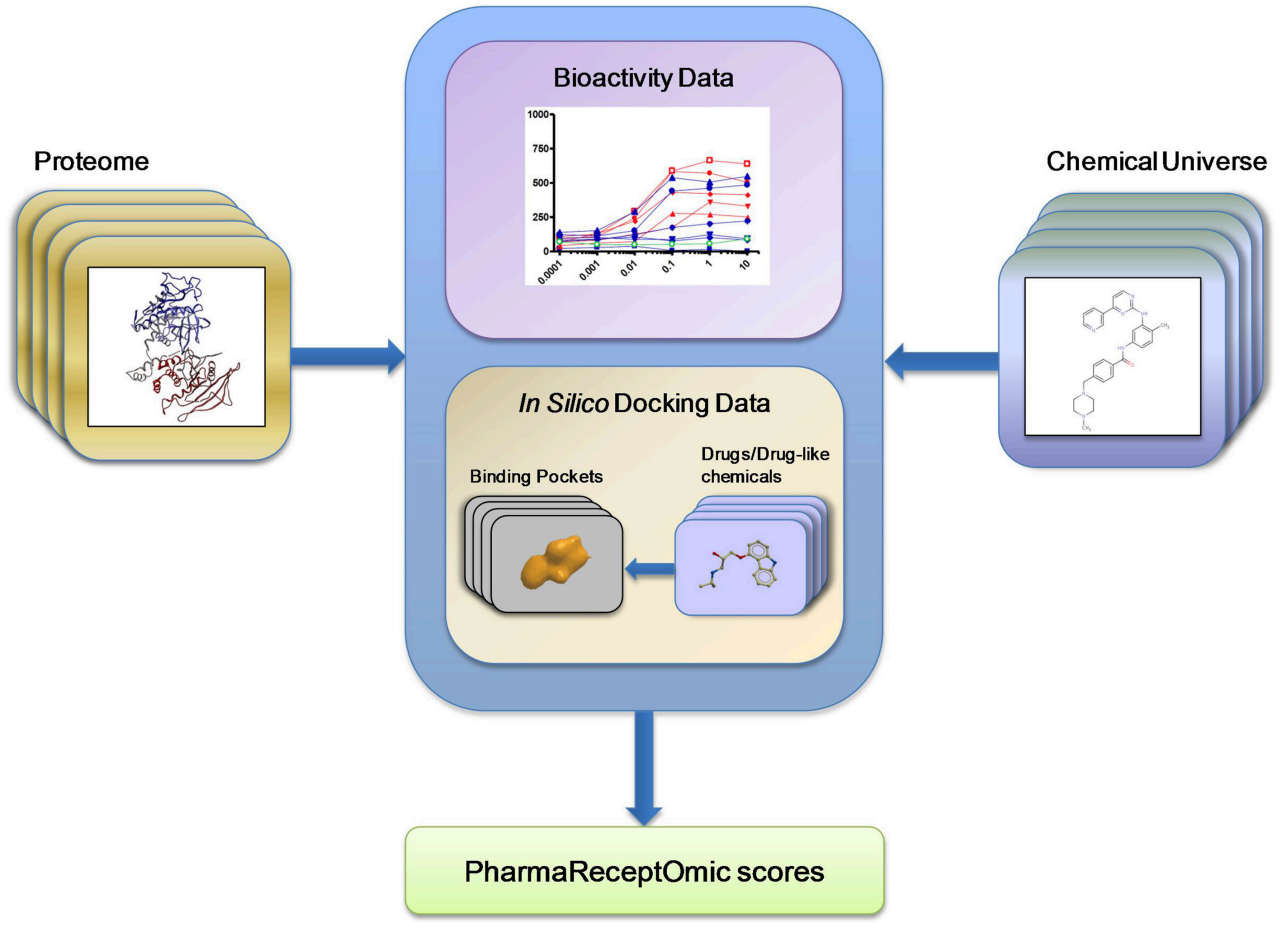
**Pharmareceptomics: a tool for connecting proteome with the chemical universe**. Pharmareceptomic or bioactivity “score,” a measure of compound-target interaction, was derived from either compound-protein bioactivity data or binding energy data estimated by *in silico* docking.

First, we generated bioactivity probability scores for the compound-receptor pairs by executing the largest computational molecular docking reported to date (see Section Methods). A benchmark docking screen was performed against 3D structural models of human proteins (Table 1). The mean area under the receiver operating curve (AUC) for benchmark docking was 0.59 (with about 23% of structures having separation power above 0.7) when performed on 3D structural models from Pocketome (Kufareva et al., 2012), but only 0.53 (with 8.5% of structures above AUC of 0.7) on ModBase (Pieper et al., 2006) homology models proteins (Table 2 and Supplementary Table 1). This result suggests that only the docking scores achieved with the highest quality Pocketome pockets should be included in our “omics” set of compound-receptor scores, which are used to predict mechanistic signatures solely from chemotype. The Pocketome currently includes 6857 pockets derived from high quality crystallographic structures of 570 target human proteins. Therefore, for our “omics” set we docked over 600,000 unique non-overlapping chemical structures from PubChem Bioassay, ChEMBL, and DrugBank against these 6857 pockets for a total of 4.2 billion pairwise docking scores between compounds and targets. These “omics” *in silico* docking scores together with the compound–receptor affinities obtained experimentally, constitute the bioactivity scores data set (Figure 2), which comprise a significant fraction of the druggable targets encoded in the human genome, by one estimate to be around 4000 targets (Reardon, 2013).

**Table 2:** **Number of receptors from the benchmark study with AUC above a certain threshold**.

**AUC threshold**	**Source**	**No. of receptors**	**% of receptors**
0.9	All	77	1.3
	Homology	20	0.9
	Pocketome	57	1.5
0.8	All	389	6.5
	Homology	55	2.6
	Pocketome	334	8.6
0.7	All	1090	18.1
	Homology	180	8.5
	Pocketome	910	23.4
0.6	All	2575	42.8
	Homology	551	25.9
	Pocketome	2024	52.0

### Generation of historeceptomic scores

**Figure 3:**
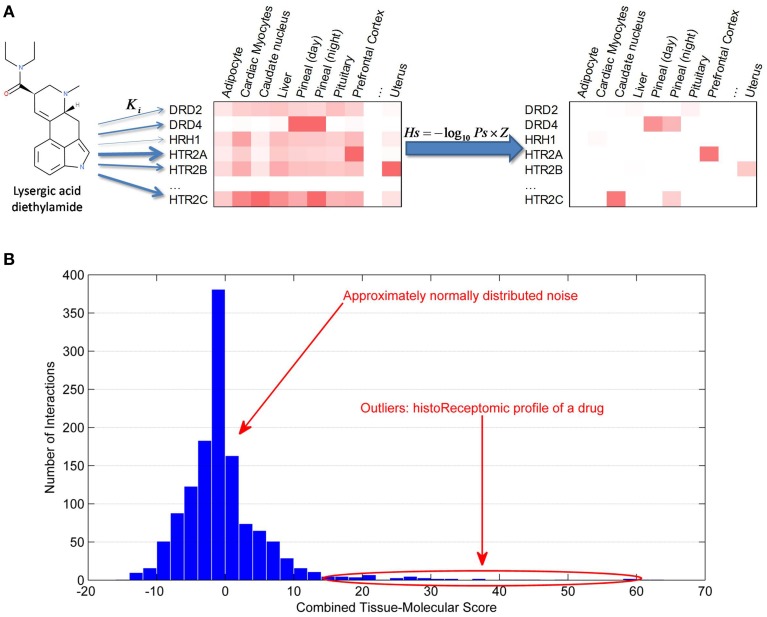
**Generation of historeceptomic profile of a drug/compound**. **(A)** Calculation of historeceptomic scores. The arrow thickness represents the strength of affinity between a drug/compound and the protein targets. Left heatmap displays gene expression data of protein targets. Right heatmap represents historeceptomic scores calculated using the formula shown, where Ps—pharmareceptomic scores and Z—normalized gene expression level. **(B)** Historeceptomic profile of a drug/compound. The majority of the tissue-specific drug: receptor interactions are physiologically insignificant and their combined scores are normally distributed, while a few outlier interactions with significantly larger scores constitute the true historeceptomic profile of the drug/compound. These tissue-specific interactions are characterized by both high compound-target affinity and high target expression in that specific tissue.

To address the issue of physiological significance of drug targets detected in the first step, we endeavored to calculate a tissue-specific (historeceptomic) compound-receptor score (Figure 3A). Tissue-specific gene expression data on protein targets were obtained from the BioGPS database. The level of expression of each receptor in every tissue was normalized with regard to its expression level in all tissues of the dataset by calculating its standard score (Z-score, see Section Methods). Each compound-receptor association in each tissue was scored by integrating their bioactivity with the receptor expression in a given tissue as follows:

$$Hs=-\log_{10}\;Ps\times Z,$$

where *Hs* is a historeceptomic score, *Ps* is a bioactivity score, and *Z* is the gene-expression Z-score.

By this method, for any given drug/compound, thousands of historeceptomic scores can be generated, but only a tiny fraction of these, which measure the probability that the compound will affect the receptor in a physiologically significant way, are important. The average drug may have hundreds of low affinity receptors, resulting in a set of scores numbering in the tens of thousands across all tissues in the human body. To identify the physiologically significant compound-receptor interactions out of the large number of all on-/off-target interactions of a given compound, we used the generalized extreme Studentized deviate test as a statistical novelty detection approach using the α = 0.0001 level of significance (Figure 3B). Statistically significant historeceptomic scores of a given drug/compound form its historeceptomic fingerprint.

Fingerprints were pre-calculated for all known drugs into an integrated system suitable for searching with any chemical structure to find its historeceptomic fingerprint. The system includes the 4.2 billion docking scores with experimental affinity scores in a graph linking drugs/compounds to protein targets in order to maximize the sensitivity of target detection for any drug.

### Illustrative use case

Historeceptomics fingerprints may specifically localize *in vivo* significant mechanisms of action of a polypharmacologic drug, translating purely molecular data into a clinically interpretable profile. An example is shown in Figure 3A. Lysergic acid diethylamide (LSD) is a hallucinogenic drug in humans, which makes it difficult to study in animal models, as many hallucinations are only represented internally and can only be communicated verbally. We calculated the historeceptomics profile for LSD. In this case, the inputs into our system were only molecular in nature: the affinity scores and the expression data. We did not use docking in this example. Our historeceptomics approach identified the 5HT2A receptor in the prefrontal cortex (PFC) as the most significant of tissue-target pair associated with the phenotype induced by LSD. Independently, we analyzed the preclinical and clinical literature on LSD targets, which is exclusively *non-molecular* data. The textbook and literature consensus from animal neuroperturbation studies, pharmacologic studies and clinical neuroimaging is that 5HT2A is the primary molecular target of LSD, and that, specifically, its activity in the PFC is responsible for its effects. Thus, there are many non-molecular clinical and translational papers in the literature, none of which were input to our system, that clearly establish 5HT2A specifically in the PFC not only as a key pathway for LSD psychosis, but also as the epicenter of the very similar psychoses seen in human schizophrenia (Arvanov et al., 1999; Vollenweider and Geyer, 2001; Muschamp et al., 2004; Nichols, 2004). The historeceptomics approach predicts this finding independently of animal or clinical studies.

## Discussion

This report takes on the two major challenges of precisely describing the holistic pharmacodynamics of drugs. First, we expanded the graph of experimental scores linking drugs/compounds to protein targets, which has been used in prior methods such as SEA (Keiser et al., 2007), to include the data from the largest computational molecular docking of compounds to protein pockets yet reported. This should increase the sensitivity of target detection. Second, we addressed, for the first time, the systematic integration of bioactivity/docking scores between drugs/compounds and proteins with the expression patterns of those proteins in human tissues, thus mapping the pharmacology of drugs into human physiologic space.

The integration of bioactivity/docking scores of compound-receptors with the expression patterns of those receptors in human tissues increases the specificity of the results by eliminating noise and selecting only physiologically significant drug-target interactions. Thus, although for many models/pockets the docking scores correlate only moderately with affinity due to the limited ability to take induced fit into account, this lack of specificity is abrogated by our integration of the gene expression such that many false positives are likely to be culled. While sensitivity is low, it can be steadily improved from our pioneering prototype by (1) improved binding site (pocket) selection methods and (2) natural growth and improved curation of the crystallographic and bioactivity databases.

There are 20,198 reviewed human proteins in UniProt, of which 4300 have human crystal structures in the PDB (21.3% of total). An additional 20–30% of these can likely be modeled reliably by homology. Thus, up to 50% of the “proteome” might already be surveyed by docking. Estimates of the druggable genome range from 8 to 12 thousand targets. The existing structures are probably highly enriched in these targets so, one can speculate that 40–50% of the druggable genome is already accessible by docking. These are highly speculative estimates, but since the number of crystal structures and the power of computation is growing rapidly, it is not unreasonable to speculate that a low resolution representation of the majority of the druggable genome could be available for docking soon.

The system has been deployed for access through a user-friendly web site: drugable.org. For compounds resulting from phenotype screens, where their mechanism of action is not known, searching the site can identify possible mechanisms of action. Similarly, where the tissue pattern of a disease is known, drug activity detected by our approach in tissues not included in the pattern could be suggestive of the mechanism of the adverse effects of a drug. Since the historeceptomic fingerprints contain both a specific pattern of targets and a specific pattern of tissues, they could potentially be matched to complex biomarkers of disease derived from exhaustive molecular profiling, which can have a similar gene-tissue signature. Our novel approach thus potentially fills a currently existing gap between burgeoning “omics” data and drugs/drug-like compounds (Figure 1).

## Author contributions

ES, AG, JS designed the study, performed experiments, analyzed data, and assisted in writing the manuscript; JX and DT performed experiments and analyzed data; SS. designed the study, analyzed data, and wrote the manuscript; TC conceived and designed the study, analyzed data, and wrote the manuscript.

## Funding

This work was supported by an American Recovery and Reinvestment Act grant from the National Library of Medicine (RC LM010994 to TC). Additional support was provided by Google, Inc. via their Exacycle for Visiting Faculty award program (to JS and TC).

### Conflict of interest statement

The authors declare that the research was conducted in the absence of any commercial or financial relationships that could be construed as a potential conflict of interest. Timothy Cardozo and Sergey V. Shmelkov are shareholders in Genecentrix Inc., a company founded after this study was completed, but based, in part, on this research. No funding was received from Genecentrix Inc. for the present study.
