# Supplementary material for: Historeceptomic Fingerprints for Drug-Like Compounds
Source: Front Physiol. 2015 Dec 18;6:371. doi: 10.3389/fphys.2015.00371 (PMC4683199; doi:10.3389/fphys.2015.00371)
Supplement: Supplementary file 1 [file Table1.PDF]

PROTEIN FAMILY	AUC
Protein kinase C delta type	0.43764251
Acetylcholinesterase	0.513333333
TRAF2 and NCK-interacting protein kinase	0.579931973
Lethal factor	0.410839161
Serum albumin	0.5625
Macrophage migration inhibitory factor	1
Coagulation factor XI	0.790849673
Coagulation factor X	0.527878655
Prothrombin	0.645573381
Peroxisome proliferator-activated receptor delta	0.544973545
Dihydrofolate reductase	0.504385965
Proto-oncogene tyrosine-protein kinase Src	0.572429792
Muscarinic acetylcholine receptor M1	0.585107461
Nitric oxide synthase, inducible	0.637443296
Dual specificity mitogen-activated protein kinase kinase 1	0.487609329
Mitogen-activated protein kinase kinase kinase 19	0.591008772
Peptidyl-prolyl cis-trans isomerase A	0.927927928
Aurora kinase A	0.523089431
Gamma-aminobutyric acid receptor subunit gamma-1	0.666666667
Transthyretin	1
Beta-secretase 1	0.844192217
Heat shock protein HSP 90-alpha	0.886792453
Tyrosine-protein kinase Blk	0.674382716
Coagulation factor VII	0.745627112
Signal transducer and activator of transcription 3	0.371794872
Fatty acid-binding protein, heart	0.324175824
AP2-associated protein kinase 1	0.489740082
Thermolysin	0.584864865
Calcium/calmodulin-dependent protein kinase type II subunit beta	0.385267275
Deoxyuridine 5'-triphosphate nucleotidohydrolase, mitochondrial	0.64690027
Cathepsin K	0.585259279
Amyloid beta A4 protein	0.616715914
Receptor-type tyrosine-protein phosphatase F	0.522727273
Ephrin type-A receptor 5	0.556451613
Tyrosine-protein kinase ITK/TSK	0.666910153
Scytalone dehydratase	0.455223881
Nitric oxide synthase, endothelial	0.410912191
Thymidylate synthase	0.626984127
Proto-oncogene tyrosine-protein kinase Src	0.495579976
Coagulation factor X	0.528931187
Histamine N-methyltransferase	0.833333333
RAC-alpha serine/threonine-protein kinase	0.59461473
TGF-beta receptor type-1	0.64968815
Ephrin type-B receptor 4	0.552897574
Serine/threonine-protein kinase D2	0.229120879
Gamma-aminobutyric acid receptor subunit alpha-1	0.588820454
Urokinase-type plasminogen activator	0.550337579
Casein kinase II subunit alpha	0.497773906
Tyrosine-protein kinase Lck	0.666933307
Induced myeloid leukemia cell differentiation protein Mcl-1	0.835849057
Mitogen-activated protein kinase 8	0.518518519
Vesicular acetylcholine transporter	0.205882353
Dual specificity protein kinase TTK	0.516830295

PROTEIN FAMILY	AUC
Cyclin-dependent kinase 2	0.395129022
Heat shock protein HSP 90-alpha	0.865566038
Heat shock protein HSP 90-alpha	0.816037736
Calcium/calmodulin-dependent protein kinase type II subunit delta	0.454040404
Neuraminidase	0.333333333
Aldo-keto reductase family 1 member C3	0.666666667
Alpha-2A adrenergic receptor	0.476088943
Casein kinase II subunit alpha	0.432249322
Tyrosine-protein kinase Lck	0.614488551
Tyrosine-protein kinase Lck	0.676082392
Egl nine homolog 1	0.445454545
AP2-associated protein kinase 1	0.534199726
Baculoviral IAP repeat-containing protein 3	0.909090909
Thymidylate synthase	0.800865801
cGMP-specific 3',5'-cyclic phosphodiesterase	0.844006568
Glycogen phosphorylase, muscle form	0.428571429
Cyclin-dependent kinase 16	0.533006536
Adenosine deaminase	0.811728395
Serine/threonine-protein kinase STK11	0.612021858
Liver carboxylesterase 1	0.546309389
Dihydrofolate reductase	0.542443818
Methionine aminopeptidase 2	0.513465447
Transthyretin	0.892857143
Peroxisome proliferator-activated receptor gamma	0.745052083
Ribosomal protein S6 kinase alpha-3	0.590163934
Estrogen receptor beta	0.677515498
Cyclin-dependent kinase 2	0.363220643
Leukotriene A-4 hydrolase	1
Serine/threonine-protein kinase PAK 7	0.504723347
Adenosylhomocysteinase	0.196598101
Mitogen-activated protein kinase kinase kinase 4	0.461471861
Alpha-1A adrenergic receptor	0.421472185
Fibroblast growth factor receptor 3	0.566165689
Casein kinase I isoform alpha-like	0.530505952
cAMP-dependent protein kinase catalytic subunit beta	0.516007533
Tyrosine-protein kinase Fgr	0.572537112
Motilin receptor	0.243902439
3-hydroxy-3-methylglutaryl-coenzyme A reductase	0.580882353
Nitric oxide synthase, inducible	0.460121269
Protein kinase C alpha type	0.607270645
MAP kinase-activated protein kinase 2	0.516584767
Peptidyl-prolyl cis-trans isomerase FKBP1A	0.748059529
Vascular endothelial growth factor receptor 2	0.608261486
Queuine tRNA-ribosyltransferase	0.657142857
Interferon-induced, double-stranded RNA-activated protein kinase	0.581168831
Pepsin A-5	0.375
Acetylcholinesterase	1
Glycogen synthase kinase-3 beta	0.517658239
Mineralocorticoid receptor	0.383616119
Peroxisome proliferator-activated receptor gamma	0.734895833
Caspase-4	0.666666667
Prothrombin	0.609837351
E3 ubiquitin-protein ligase XIAP	0.572193347

PROTEIN FAMILY	AUC
Prothrombin	0.45227374
Tyrosine-protein phosphatase non-receptor type 11	0.538461538
Glutamate carboxypeptidase 2	0.701298701
Serine/threonine-protein kinase pim-1	0.717709019
Cationic trypsin	0.662350598
Dipeptidyl peptidase 4	0.709030848
Solute carrier family 15 member 1	0.841704036
5-hydroxytryptamine receptor 2A	0.485867207
Carbonic anhydrase 2	0.764765529
Coagulation factor X	0.529353104
Adenosine kinase	0.9
Phosphatidylinositol 4,5-bisphosphate 3-kinase catalytic subunit gamma isoform	0.551005747
Metabotropic glutamate receptor 1	0.841346154
Prostaglandin E2 receptor EP2 subtype	0.492871386
WD repeat-containing protein 5	0.152173913
BDNF/NT-3 growth factors receptor	0.391101695
Leucine-rich repeat serine/threonine-protein kinase 2	0.456002331
Aldose reductase	0.486111111
Integrin alpha-IIb	0.460233029
Prothrombin	0.598471487
M-phase inducer phosphatase 3	0.571428571
Galectin-7	0.752873563
Kallikrein-7	1
Peroxisome proliferator-activated receptor gamma	0.730121528
Melanin-concentrating hormone receptor 1	0.620590705
NADPH oxidase 4	0.391581633
Cell division protein ZipA	0
Aldose reductase	0.444444444
Alcohol dehydrogenase E chain	0.297222222
Glutamine--tRNA ligase	0.95
UDP-glucuronosyltransferase 2B7	1
Tyrosine-protein kinase FRK	0.569327731
Proto-oncogene tyrosine-protein kinase Src	0.652014652
Tyrosine-protein kinase Mer	0.588259442
Solute carrier family 22 member 6	0.666666667
Glutamate receptor ionotropic, NMDA 1	0.282608696
AMP deaminase 3	0.619002525
Serine/threonine-protein kinase 36	0.476870748
Cationic trypsin	0.666360813
Myosin light chain kinase 3	0.562142857
cAMP-specific 3',5'-cyclic phosphodiesterase 4B	0.69390507
Cathepsin B	0.606850676
Acetylcholinesterase	1
Coagulation factor XI	0.581699346
Thymidylate synthase	0.607503608
Lysosomal alpha-glucosidase	0.203703704
Renin	0.757694129
Beta-secretase 1	0.780586675
Ectonucleotide pyrophosphatase/phosphodiesterase family member 1	0.416666667
Carbonic anhydrase 2	0.767871395
cAMP-dependent protein kinase catalytic subunit alpha	0.424803264
Alpha-galactosidase A	1
MAP kinase-activated protein kinase 2	0.755151515

PROTEIN FAMILY	AUC
Serine/threonine-protein kinase B-raf	0.645514456
Mitogen-activated protein kinase 1	0.673315118
Peptidyl-prolyl cis-trans isomerase FKBP1A	0.743072038
Serine/threonine-protein kinase PAK 1	0.540372671
Myosin-IIIb	0.517304189
Serum albumin	0.506944444
72 kDa type IV collagenase	0.451344467
Coagulation factor VII	0.717700258
Cationic trypsin	0.662131519
Mitogen-activated protein kinase 10	0.577813853
Phosphatidylinositol 4,5-bisphosphate 3-kinase catalytic subunit gamma isoform	0.593390805
Poly [ADP-ribose] polymerase 3	1
Dipeptidyl peptidase 4	0.678555462
Vascular endothelial growth factor receptor 3	0.604110208
Methionine aminopeptidase 2	0.52921748
Amine oxidase [flavin-containing] B	0.608710462
Cyclin-dependent kinase 13	0.567736185
cAMP-dependent protein kinase catalytic subunit alpha	0.468813757
Peroxisome proliferator-activated receptor alpha	0.239547038
Cathepsin S	0.719963785
RAC-beta serine/threonine-protein kinase	0.426074634
Estrogen receptor	0.679691695
cAMP-specific 3',5'-cyclic phosphodiesterase 4D	0.717430717
Tyrosine-protein kinase ABL1	0.518992744
NADPH oxidase 4	0.485969388
Kinesin-like protein KIF11	0.557692308
Interstitial collagenase	0.479369423
Estrogen receptor	0.607627081
Dipeptidyl peptidase 4	0.701380955
Glucagon receptor	0.684311224
6,7-dimethyl-8-ribityllumazine synthase	0.484693878
Interstitial collagenase	0.45057121
Transthyretin	1
Glutathione S-transferase P	0.8
Coagulation factor X	0.565998718
Beta-lactamase OXA-10	1
Lethal factor	0.560314685
Acetylcholinesterase	0.46
Serine/threonine-protein kinase 36	0.409333333
Peptidyl-prolyl cis-trans isomerase FKBP4	0.523809524
Peptidyl-prolyl cis-trans isomerase FKBP1A	0.800339983
RAC-beta serine/threonine-protein kinase	0.384506377
Leucine-rich repeat serine/threonine-protein kinase 2	0.427847346
Carnitine O-acetyltransferase	0.111111111
Methionine aminopeptidase 2	0.546239837
Nuclear receptor corepressor 2	0.333333333
5'-AMP-activated protein kinase catalytic subunit alpha-1	0.408181818
3-hydroxy-3-methylglutaryl-coenzyme A reductase	0.639705882
Tissue alpha-L-fucosidase	0.537296037
3-dehydroquinate dehydratase	0.611111111
Dual specificity mitogen-activated protein kinase kinase 1	0.362973761
Estrogen receptor	0.724013335
Prostasin	0.782608696

PROTEIN FAMILY	AUC
Androgen receptor	0.642939192
Acetylcholinesterase	0.393333333
Growth factor receptor-bound protein 2	0.53030303
Serum albumin	0.423611111
Leukotriene A-4 hydrolase	0.333333333
Retinoic acid receptor RXR-alpha	0.515063168
Tissue-type plasminogen activator	0.450267591
Prothrombin	0.625675154
Tyrosine-protein kinase Fes/Fps	0.436898396
Thermolysin	0.560540541
Aminopeptidase N	0.333333333
Beta-lactamase TEM	0.479411765
Nitric oxide synthase, inducible	0.6437437
Corticosteroid 11-beta-dehydrogenase isozyme 1	0.589410589
Serine/threonine-protein kinase MARK2	0.652380952
Cruzipain	0.977777778
Serine hydroxymethyltransferase, cytosolic	0.5
Estrogen receptor	0.639084034
Serine/threonine-protein kinase MARK2	0.601190476
Prothrombin	0.423077149
Casein kinase II subunit alpha	0.43960511
Peroxisome proliferator-activated receptor gamma	0.778993056
Ribonuclease pancreatic	0.329192547
cAMP-specific 3',5'-cyclic phosphodiesterase 4D	0.537814038
Ribosomal protein S6 kinase alpha-1	0.519686907
MAP kinase-interacting serine/threonine-protein kinase 1	0.566358025
Papain	0.489770867
Heat shock protein HSP 90-alpha	0.879716981
Coagulation factor X	0.576511065
Glycogen synthase kinase-3 beta	0.537393918
Carbonic anhydrase 2	0.761920644
Acidic mammalian chitinase	1
Tyrosine-protein kinase Lck	0.676582342
3-phosphoinositide-dependent protein kinase 1	0.369230769
Coagulation factor VII	0.717253031
Tyrosine-protein kinase BTK	0.46668956
Mitogen-activated protein kinase 14	0.728256346
Tyrosine-protein kinase ABL1	0.564198752
Serine/threonine-protein kinase 10	0.545965608
Urokinase-type plasminogen activator	0.541258598
Dipeptidyl peptidase 4	0.70031295
Cyclin-dependent kinase 17	0.537037037
Renin	0.724668561
Dihydrofolate reductase	0.565644618
Glutathione S-transferase A1	0.466666667
Glutamate receptor ionotropic, kainate 2	0.513460703
Acetylcholinesterase	1
Renin	0.607481061
Carbonic anhydrase 1	0.713751426
cAMP-dependent protein kinase catalytic subunit alpha	0.422908773
cAMP-specific 3',5'-cyclic phosphodiesterase 4D	0.777777778
Calcium/calmodulin-dependent protein kinase type II subunit gamma	0.574404762
Kinesin-like protein KIF11	0.778846154

PROTEIN FAMILY	AUC
Macrophage metalloelastase	0.337710084
Caspase-5	0.25
Ephrin type-A receptor 1	0.590416667
Mitogen-activated protein kinase kinase kinase 9	0.44335512
6,7-dimethyl-8-ribityllumazine synthase	0.418367347
Serum albumin	0.493055556
Prostaglandin G/H synthase 1	0.7
Protein S100-B	0.333333333
STE20-like serine/threonine-protein kinase	0.519661222
Peptidyl-prolyl cis-trans isomerase A	0.89527027
Angiotensin-converting enzyme	0.616508152
Tyrosine-protein phosphatase non-receptor type 1	0.666835203
Dipeptidyl peptidase 4	0.685972505
cAMP-specific 3',5'-cyclic phosphodiesterase 4D	0.617845118
Alcohol dehydrogenase class 4 mu/sigma chain	0.35
Caspase-3	0.880050505
Carbonic anhydrase 2	0.764846818
Glycogen synthase kinase-3 beta	0.558654526
Coagulation factor VII	0.792436891
Glucosylceramidase	0.734962406
D(2) dopamine receptor	0.546409281
Dihydrofolate reductase	0.391081871
Peripheral plasma membrane protein CASK	0.603386809
Aldo-keto reductase family 1 member C3	0.666666667
Glutamate receptor ionotropic, kainate 1	0.272115385
Serine/threonine-protein kinase Chk2	0.611111111
Serine/threonine-protein kinase tousled-like 1	0.590909091
Squalene monooxygenase	1
Stromelysin-1	0.600756193
Platelet-activating factor receptor	0.449876543
Beta-lactamase	0.202380952
Coagulation factor VII	0.805704631
Peroxisome proliferator-activated receptor gamma	0.790711806
Phosphatidylinositol 4,5-bisphosphate 3-kinase catalytic subunit gamma isoform	0.708333333
Phosphatidylinositol 4-kinase type 2-beta	0.3928
Acetylcholinesterase	0.393333333
Coagulation factor X	0.564153329
Serine/threonine-protein kinase BRSK2	0.526229508
Serum albumin	0.368055556
Hepatocyte growth factor receptor	0.620835686
Cathepsin S	0.659868719
Aldose reductase	0.638888889
Tyrosine-protein kinase JAK2	0.596590241
3-phosphoshikimate 1-carboxyvinyltransferase	0.622222222
Beta-1,4-galactosyltransferase 1	1
5-hydroxytryptamine receptor 3B	0.789473684
Prostaglandin E2 receptor EP3 subtype	0.40186279
Cyclin-dependent kinase 2	0.362730688
Interleukin-1 receptor-associated kinase 4	0.745355191
Beta-lactamase	0.196236559
Serum albumin	0.451388889
Acetylcholinesterase	0.513333333
Serine/threonine-protein kinase 36	0.467346939

PROTEIN FAMILY	AUC
Caspase-3	0.935606061
Tyrosine-protein kinase ABL1	0.555084746
Serine hydroxymethyltransferase, cytosolic	0.5
Phenylethanolamine N-methyltransferase	0.532077206
Caspase-5	0.2
Cathepsin L1	0.61370725
Metallo-beta-lactamase L1	0.87962963
cAMP-specific 3',5'-cyclic phosphodiesterase 4D	0.624579125
Cyclin-dependent kinase 2	0.355748816
Peroxisome proliferator-activated receptor delta	0.796296296
Renin	0.77639678
Beta-lactamase	0.198611111
Cathepsin S	0.711226799
Tryptase alpha/beta-1	0.266042781
Breast cancer type 1 susceptibility protein	0.125
Serine/threonine-protein kinase pim-1	0.680678078
Prolyl endopeptidase	0.293181818
Somatostatin receptor type 5	0.638043478
Acetylcholinesterase	0.433333333
Cyclin-dependent kinase 2	0.326310632
Collagenase 3	0.412695652
Fibroblast growth factor 1	0.427350427
NT-3 growth factor receptor	0.457142857
3-hydroxy-3-methylglutaryl-coenzyme A reductase	0.639705882
5'-AMP-activated protein kinase catalytic subunit alpha-1	0.54978355
Serine/threonine-protein kinase SBK1	0.5
Dual specificity mitogen-activated protein kinase kinase 7	0.381481481
Thymidylate kinase	0.785714286
Coagulation factor X	0.52785465
Mitogen-activated protein kinase kinase kinase 5	0.376865672
Peroxisome proliferator-activated receptor gamma	0.745833333
Transthyretin	0.946428571
Serum albumin	0.4375
Coagulation factor X	0.556871369
Cathepsin E	0.479166667
Cyclin-dependent kinase 2	0.374081333
Proprotein convertase subtilisin/kexin type 9	0.25
Chaperone activity of bc1 complex-like, mitochondrial	0.512711864
Prothrombin	0.64393494
Carbonic anhydrase 2	0.766595583
Type II inositol 1,4,5-trisphosphate 5-phosphatase	0.375
Tyrosine-protein kinase BTK	0.521634615
Tyrosine-protein kinase Fyn	0.596059113
Abelson tyrosine-protein kinase 2	0.58361678
Casein kinase II subunit alpha	0.417247387
Stromelysin-1	0.432090057
Peroxisome proliferator-activated receptor gamma	0.658072917
Mitogen-activated protein kinase 14	0.692450409
Sodium-dependent noradrenaline transporter	0.567162106
Serine/threonine-protein kinase D3	0.566563467
Guanine deaminase	0.277777778
Mitogen-activated protein kinase 3	0.86
Serum albumin	0.465277778

PROTEIN FAMILY	AUC
Metabotropic glutamate receptor 3	0.352941176
Serine/threonine-protein kinase pim-1	0.696905859
Platelet-activating factor receptor	0.497283951
Histamine N-methyltransferase	0.833333333
Disintegrin and metalloproteinase domain-containing protein 17	0.588866778
Cholinesterase	0.815421748
Beta-secretase 1	0.835126769
Serine/threonine-protein kinase B-raf	0.600233844
Microtubule-associated serine/threonine-protein kinase 1	0.410493827
Estrogen receptor	0.690408538
Uridine 5'-monophosphate synthase	0.2
Protein kinase C gamma type	0.512169312
Serine/threonine-protein kinase PLK1	0.455691367
Fatty acid-binding protein, adipocyte	0.917763158
Cationic trypsin	0.662865749
Mitogen-activated protein kinase kinase kinase 19	0.538925439
E3 ubiquitin-protein ligase XIAP	0.652079002
Lysozyme	0.768518519
Phenylethanolamine N-methyltransferase	0.7578125
TRAF2 and NCK-interacting protein kinase	0.597222222
Peroxisome proliferator-activated receptor gamma	0.768489583
Tubulin beta-2B chain	0
Metabotropic glutamate receptor 1	0.694711538
Chymotrypsin-C	0.495015949
Dual specificity tyrosine-phosphorylation-regulated kinase 1A	0.617969822
Dipeptidyl peptidase 4	0.722194625
Fibroblast growth factor receptor 1	0.66462585
Phenylethanolamine N-methyltransferase	0.532444853
Glycogen phosphorylase, muscle form	0.285714286
Acetylcholinesterase	1
Serum albumin	0.548611111
Ribonuclease pancreatic	0.381987578
Transient receptor potential cation channel subfamily M member 6	0.409836066
Insulin receptor-related protein	0.580357143
Tyrosine-protein kinase ABL1	0.556900726
Aldose reductase	0.555555556
CREB-binding protein	0.444444444
Nitric oxide synthase, endothelial	0.419579426
Cyclin-dependent kinase 2	0.340703087
Fibroblast growth factor receptor 2	0.576003086
Tyrosine-protein kinase ABL1	0.567182758
cGMP-dependent protein kinase 2	0.552586207
Ribonuclease pancreatic	0.394409938
Deoxyuridine 5'-triphosphate nucleotidohydrolase, mitochondrial	0.637466307
Serine/threonine-protein kinase BRSK1	0.559259259
Integrin alpha-L	0.957317073
Bromodomain-containing protein 4	0.375
Prothrombin	0.557905621
MAP kinase-activated protein kinase 2	0.676363636
Calcium/calmodulin-dependent protein kinase kinase 2	0.576736111
Receptor-type tyrosine-protein phosphatase epsilon	0.5
Gamma-aminobutyric acid receptor subunit theta	0.333333333
Baculoviral IAP repeat-containing protein 2	0.5

PROTEIN FAMILY	AUC
Glutathione S-transferase P	0.75
Tyrosine-protein kinase Fyn	0.606470588
Androgen receptor	0.643635742
Mitogen-activated protein kinase 15	0.53627451
Tyrosine-protein kinase Fyn	0.552427868
Dihydroorotate dehydrogenase (quinone), mitochondrial	0.704166667
Phosphorylase b kinase gamma catalytic chain, liver/testis isoform	0.311320755
Phosphatidylinositol 4-kinase beta	0.354152933
Methionine aminopeptidase 2	0.510924797
Myotonin-protein kinase	0.449951409
Peptidyl-prolyl cis-trans isomerase A	0.907657658
Mitogen-activated protein kinase kinase kinase 19	0.51399776
Androgen receptor	0.640825105
Cathepsin L1	0.636607387
Aurora kinase C	0.608796296
RAC-alpha serine/threonine-protein kinase	0.6050447
Tyrosine-protein phosphatase non-receptor type 2	0.548824593
Aldose reductase	0.5
Serine/threonine-protein kinase SIK3	0.569444444
Beta-lactamase TEM	0.479411765
Carbonic anhydrase 2	0.760075814
Deoxyuridine 5'-triphosphate nucleotidohydrolase, mitochondrial	0.584231806
Proto-oncogene tyrosine-protein kinase Src	0.506275946
Cationic trypsin	0.673543599
Cationic trypsin	0.641078543
Inhibitor of nuclear factor kappa-B kinase subunit epsilon	0.484242424
Receptor tyrosine-protein kinase erbB-4	0.43622449
Mitogen-activated protein kinase 10	0.585281385
Hepatocyte growth factor receptor	0.352096547
Mitogen-activated protein kinase kinase kinase 1	0.462962963
Tyrosine-protein kinase receptor TYRO3	0.391786903
Peroxisome proliferator-activated receptor gamma	0.7328125
Tyrosine-protein kinase Lck	0.667483252
Carbonic anhydrase 2	0.758286707
Neuromedin-B receptor	0.322752986
Beta-lactamase	0.406666667
Acetylcholinesterase	0.393333333
Mitogen-activated protein kinase 14	0.665175475
Hepatocyte growth factor receptor	0.597736082
Tyrosine-protein kinase Lck	0.624987501
Casein kinase I isoform gamma-3	0.369799692
Heat shock protein HSP 90-alpha	0.885613208
Seminal ribonuclease	0.833333333
Beta-lactamase	0.209876543
Mast/stem cell growth factor receptor Kit	0.776101008
Nuclear receptor corepressor 2	0.5
Aurora kinase A	0.506928105
Mitogen-activated protein kinase 14	0.69890068
Cationic trypsin	0.680504781
Cationic trypsin	0.650303644
3-hydroxy-3-methylglutaryl-coenzyme A reductase	0.742647059
Vascular endothelial growth factor receptor 2	0.677916621
Death-associated protein kinase 1	0.529090909

PROTEIN FAMILY	AUC
Peroxisome proliferator-activated receptor gamma	0.7515625
Aurora kinase A	0.570569106
Leukotriene A-4 hydrolase	1
Rho-associated protein kinase 1	0.380333333
Beta-2 adrenergic receptor	0.547769265
Bromodomain-containing protein 4	0.25
Estrogen receptor	0.693205209
Serine/threonine-protein kinase Nek2	0.42
Dual specificity tyrosine-phosphorylation-regulated kinase 1A	0.633670034
Peroxisome proliferator-activated receptor gamma	0.789322917
cAMP-specific 3',5'-cyclic phosphodiesterase 4B	0.84223301
Carboxypeptidase B2	0.724137931
AMP deaminase 1	0.641156463
Dihydrofolate reductase	0.559548722
cAMP-specific 3',5'-cyclic phosphodiesterase 4C	0.700892857
Receptor-type tyrosine-protein phosphatase C	0.689814815
Carbonic anhydrase 2	0.755246666
Beta-lactamase OXA-10	1
CREB-binding protein	0.5
Histone deacetylase 7	0.825
cAMP-specific 3',5'-cyclic phosphodiesterase 4D	0.724812225
Transthyretin	0.642857143
DNA polymerase alpha catalytic subunit	0.196078431
Serine/threonine-protein kinase PLK1	0.427425516
Aurora kinase B	0.68470483
Fibroblast growth factor receptor 2	0.501929012
Suppressor of tumorigenicity 14 protein	0.341194969
Serine/threonine-protein kinase Chk1	0.650980392
Kinesin-like protein KIF11	0.826923077
Serine/threonine-protein kinase Nek11	0.37755102
Acetylcholinesterase	1
Mitogen-activated protein kinase 14	0.669978499
Cationic trypsin	0.642643711
Prothrombin	0.581773879
Glutamate receptor ionotropic, kainate 2	0.624294399
Receptor-type tyrosine-protein phosphatase alpha	0.980676329
cAMP-dependent protein kinase catalytic subunit alpha	0.413290586
Coagulation factor VII	0.707960644
Cationic trypsin	0.666236374
Mitogen-activated protein kinase 14	0.658603829
Coagulation factor X	0.552659033
Thymidylate synthase	0.642135642
cAMP-dependent protein kinase catalytic subunit alpha	0.625036433
Prothrombin	0.62404468
Sialidase-2	0.166666667
Serine/threonine-protein kinase B-raf	0.661670918
cAMP-dependent protein kinase catalytic subunit alpha	0.421742932
Solute carrier family 15 member 1	0.761659193
Urokinase-type plasminogen activator	0.556681583
Cyclin-dependent kinase 2	0.355320105
Heat shock protein HSP 90-alpha	0.879716981
Chymase	0.776699029
Ephrin type-A receptor 6	0.351785714

PROTEIN FAMILY	AUC
Beta-secretase 1	0.753832547
Angiotensin-converting enzyme	0.574388587
Dipeptidyl peptidase 2	0.565335753
Liver carboxylesterase 1	0.383823529
Acetylcholinesterase	1
Thyroid hormone receptor beta	0.108058608
Serine/threonine-protein kinase B-raf	0.630527211
Aldo-keto reductase family 1 member C1	0.854166667
Estrogen receptor	0.68942411
Receptor-type tyrosine-protein phosphatase beta	0.5625
Caspase-8	0.692307692
Calcitonin gene-related peptide type 1 receptor	0.659482759
Coagulation factor X	0.557385268
Prostaglandin D2 receptor 2	0.626928312
Mitogen-activated protein kinase kinase kinase kinase 5	0.48121292
Protein S100-B	0
Serine/threonine-protein kinase Chk1	0.613235294
Cationic trypsin	0.656320304
Beta-lactamase OXA-10	1
Prothrombin	0.448836003
Estrogen receptor beta	0.67413269
Beta-lactamase	0.201388889
Leucine-rich repeat serine/threonine-protein kinase 2	0.446678322
Epidermal growth factor receptor	0.526041667
cGMP-specific 3',5'-cyclic phosphodiesterase	0.91543514
Nitric oxide synthase, endothelial	0.385905087
Fibroblast growth factor receptor 2	0.442708333
Nuclear receptor corepressor 2	0.333333333
Tyrosine-protein phosphatase non-receptor type 1	0.670389778
Retinoic acid receptor gamma	0.631307339
Tyrosine-protein kinase JAK2	0.599418329
Prothrombin	0.419777594
Chymotrypsinogen A	0.738184932
STE20/SPS1-related proline-alanine-rich protein kinase	0.416161616
Wee1-like protein kinase	0.561309524
cGMP-dependent protein kinase 2	0.596551724
Cationic trypsin	0.656341108
Trypsin	0.975
Carboxypeptidase A1	0.173611111
Prothrombin	0.444469802
Dipeptidyl peptidase 4	0.703119567
Phosphatidylinositol 4,5-bisphosphate 3-kinase catalytic subunit gamma isoform	0.50933908
Adenosylhomocysteinase	0.279667722
Inosine-5'-monophosphate dehydrogenase 1	0.791666667
Insulin-like growth factor 1 receptor	0.67605042
Histone deacetylase 6	0.978723404
Plasminogen	0.404431287
Peptidyl-prolyl cis-trans isomerase FKBP1A	0.792738469
Aldose reductase	0.5
Phosphoglycerate kinase	0.675
Coagulation factor X	0.522072548
Hepatocyte growth factor receptor	0.633104299
Calcium/calmodulin-dependent protein kinase type II subunit alpha	0.438374126

PROTEIN FAMILY	AUC
Metallo-beta-lactamase L1	0.805555556
Ephrin type-A receptor 4	0.441568627
M-phase inducer phosphatase 3	0.642857143
Acetylcholinesterase	0.513333333
Sodium- and chloride-dependent glycine transporter 1	0.818181818
Cyclin-dependent kinase 15	0.475113122
Alpha-2B adrenergic receptor	0.529867055
Mitogen-activated protein kinase kinase kinase 19	0.572228443
Prothrombin	0.44710899
Peptidyl-prolyl cis-trans isomerase NIMA-interacting 1	0.727513228
Dihydroorotate dehydrogenase (quinone), mitochondrial	0.590530303
Glucocorticoid receptor	0.68633362
Lanosterol 14-alpha demethylase	0
Interleukin-1 receptor-associated kinase 1	0.628365385
Serine/threonine-protein kinase MRCK alpha	0.433060109
Ribonuclease pancreatic	0.381987578
Uridine 5'-monophosphate synthase	0.15
Endothelin-converting enzyme 1	0.380519481
Carnitine O-acetyltransferase	0.5
Casein kinase II subunit alpha	0.54209284
Acetylcholinesterase	0.393333333
Aldose reductase	0.527777778
Proto-oncogene tyrosine-protein kinase Src	0.578852259
Serine/threonine-protein kinase DCLK2	0.409848485
Urokinase-type plasminogen activator	0.542146018
Proto-oncogene tyrosine-protein kinase Src	0.543199023
Glutathione S-transferase P	0.725
Mitogen-activated protein kinase 14	0.711159662
Macrophage metalloelastase	0.371323529
Uridine 5'-monophosphate synthase	0.4
Beta-lactamase OXA-10	1
Receptor-type tyrosine-protein phosphatase beta	0.5375
Carbonic anhydrase 2	0.764731219
Gamma-aminobutyric acid receptor subunit alpha-5	0.532263242
SHC-transforming protein 1	0.9
Protein-tyrosine kinase 2-beta	0.63872549
Amyloid beta A4 protein	0.559307725
Proto-oncogene tyrosine-protein kinase Src	0.495018315
Heat shock protein HSP 90-alpha	0.883254717
Fatty-acid amide hydrolase 1	0.607212333
Peroxisome proliferator-activated receptor gamma	0.764756944
Renin	0.641335227
Casein kinase I isoform gamma-3	0.449152542
Dihydroorotate dehydrogenase (quinone), mitochondrial	0.68280303
Cyclin-dependent kinase 1	0.619402985
M-phase inducer phosphatase 3	0.642857143
N-acetylated-alpha-linked acidic dipeptidase 2	0.684210526
3-phosphoinositide-dependent protein kinase 1	0.341176471
Retinoic acid receptor RXR-alpha	0.59159378
Melatonin receptor type 1B	0.178672032
Casein kinase II subunit alpha'	0.368165785
Peptidyl-prolyl cis-trans isomerase A	0.898085586
Urokinase-type plasminogen activator	0.568242049

PROTEIN FAMILY	AUC
Cathepsin S	0.680149389
Cathepsin S	0.681711182
Chymotrypsinogen B	0.279885057
Testis-specific serine/threonine-protein kinase 1	0.389400922
Alcohol dehydrogenase class 4 mu/sigma chain	0.366666667
Bromodomain-containing protein 4	0.25
Arginase-1	0.214285714
Fatty acid-binding protein, adipocyte	0.819078947
Glycogen synthase kinase-3 beta	0.547869519
Serine/threonine-protein kinase Chk1	0.63311359
Casein kinase II subunit alpha	0.408923732
Fibroblast growth factor receptor 4	0.635602679
Serine/threonine-protein kinase Sgk1	0.785714286
Phosphoglycerate kinase	0.7125
Renin	0.786221591
Prostaglandin E2 receptor EP2 subtype	0.517397807
Melatonin receptor type 1A	0.482765152
Orotidine 5'-phosphate decarboxylase	0.485714286
Xaa-Pro dipeptidase	0.5
CREB-binding protein	0.555555556
Serine/threonine-protein kinase PLK1	0.456073338
Nitric oxide synthase, endothelial	0.378942882
Caspase-1	0.711052111
Cationic trypsin	0.679507488
cAMP-dependent protein kinase catalytic subunit alpha	0.436170213
Beta-lactamase	0.201149425
Mitogen-activated protein kinase 11	0.609114583
Thymidylate synthase	0.686710657
Phosphatidylinositol 4,5-bisphosphate 3-kinase catalytic subunit gamma isoform	0.693247126
Serum albumin	0.423611111
Mitogen-activated protein kinase 8	0.574795082
M-phase inducer phosphatase 3	0.5
Coagulation factor VII	0.656479825
Serine/threonine-protein kinase PAK 1	0.621635611
Egl nine homolog 1	0.254545455
Acetylcholinesterase	0.486666667
Nitric oxide synthase, inducible	0.637317288
Ephrin type-B receptor 4	0.539083558
Ephrin type-B receptor 4	0.549191375
Queuine tRNA-ribosyltransferase	0.7
Glutamate receptor ionotropic, kainate 1	0.298557692
Ribosyldihydronicotinamide dehydrogenase [quinone]	0.144444444
Estrogen receptor	0.713520226
Phosphatidylinositol 4,5-bisphosphate 3-kinase catalytic subunit gamma isoform	0.665948276
Growth factor receptor-bound protein 2	0.617424242
Proto-oncogene tyrosine-protein kinase Src	0.502002442
Nociceptin receptor	0.431821237
Ephrin type-A receptor 7	0.537808642
Squalene synthase	0.863636364
TGF-beta receptor type-1	0.632276507
Glycogen phosphorylase, liver form	0.285714286
Serine/threonine-protein kinase 33	0.581521739
Chymotrypsinogen A	0.579475309

PROTEIN FAMILY	AUC
Hepatocyte growth factor receptor	0.488927739
Fibroblast growth factor receptor 3	0.651209677
Death-associated protein kinase 2	0.533539732
Serine/threonine-protein kinase Nek3	0.297413793
Orotidine 5'-phosphate decarboxylase	0.45
Furin	0.86746988
Aldo-keto reductase family 1 member C3	0.722222222
Caspase-3	0.931818182
Aldo-keto reductase family 1 member C3	0.611111111
Cyclin-dependent kinase 2	0.369773804
Mitogen-activated protein kinase 14	0.664643267
Proto-oncogene tyrosine-protein kinase Src	0.47042735
Tyrosine-protein kinase ABL1	0.559535177
Transthyretin	0.821428571
Integrin alpha-L	0.780487805
Androgen receptor	0.395872031
Dipeptidyl peptidase 4	0.721449506
Adenosine deaminase	0.817901235
Thymidylate kinase	0.671588685
Glutamate receptor 3	0.424242424
Thermolysin	0.590810811
Serine/threonine-protein kinase Chk1	0.637990196
cAMP-specific 3',5'-cyclic phosphodiesterase 4D	0.775446775
Focal adhesion kinase 1	0.512037037
Prostaglandin G/H synthase 1	0.6
Tubulin--tyrosine ligase	0.117647059
Glutamate receptor ionotropic, kainate 3	0.2375
Beta-lactamase OXA-10	1
Serine/threonine-protein kinase/endoribonuclease IRE1	0.463979417
Estrogen receptor	0.543181053
Prostaglandin G/H synthase 1	0.8
Stromelysin-1	0.445644063
Calcium/calmodulin-dependent protein kinase type II subunit beta	0.423717949
Urokinase-type plasminogen activator	0.561835249
Nitric oxide synthase, inducible	0.638545867
3-phosphoinositide-dependent protein kinase 1	0.400904977
Indoleamine 2,3-dioxygenase 1	0.571428571
Purine nucleoside phosphorylase	0.44445199
Lethal factor	0.393356643
Ephrin type-A receptor 6	0.530182927
Aminopeptidase N	0.666666667
Glucocorticoid receptor	0.624863623
Carbonic anhydrase 1	0.700040101
Carbonic anhydrase 4	0.825545541
Prostaglandin D2 receptor	0.383442266
Histone deacetylase 6	0.635416667
Dual specificity mitogen-activated protein kinase kinase 1	0.407069971
Serine/threonine-protein kinase DCLK1	0.569716776
Heat shock protein HSP 90-alpha	0.884433962
Thymidylate synthase	0.727295303
Estradiol 17-beta-dehydrogenase 1	0.666666667
Carbonic anhydrase 2	0.767343354
Macrophage metalloelastase	0.34112395

PROTEIN FAMILY	AUC
5-hydroxytryptamine receptor 5A	0.576238687
Glutathione S-transferase Mu 1	0.6
cAMP-dependent protein kinase catalytic subunit alpha	0.616584086
Cathepsin S	0.713829787
Glutamate receptor 1	0.480633803
Serine/threonine-protein kinase Chk1	0.597512438
Tyrosine-protein kinase Lck	0.661283872
Coagulation factor VII	0.694941364
Mu-type opioid receptor	0.509127053
Gamma-aminobutyric acid receptor subunit beta-2	0.5125
Caspase-6	0.7
Glycogen phosphorylase, muscle form	0.285714286
WD repeat-containing protein 5	0.565217391
Chymotrypsinogen A	0.707876712
D(2) dopamine receptor	0.620541165
Peptidyl-prolyl cis-trans isomerase A	0.942567568
Serine/threonine-protein kinase Nek4	0.538135593
Coagulation factor VII	0.761329756
Dipeptidyl peptidase 4	0.707168049
Adenosine deaminase	0.804526749
MAP kinase-activated protein kinase 2	0.666060606
Thymidylate synthase	0.709366097
Phosphatidylinositol 4,5-bisphosphate 3-kinase catalytic subunit gamma isoform	0.716954023
Glycogen synthase kinase-3 beta	0.560488861
Tyrosine-protein phosphatase non-receptor type 1	0.632668383
Disintegrin and metalloproteinase domain-containing protein 17	0.63369292
Glutamate receptor 2	0.318055556
Protein kinase C zeta type	0.7
Choline O-acetyltransferase	0.714285714
Phosphatidylinositol 4-phosphate 5-kinase type-1 alpha	0.570165094
cAMP-specific 3',5'-cyclic phosphodiesterase 4D	0.641414141
Carnitine O-acetyltransferase	0.111111111
Serine/threonine-protein kinase PLK1	0.373567609
Carbonic anhydrase 2	0.769637051
Thyroid hormone receptor alpha	0.124264706
Amine oxidase [flavin-containing] B	0.600875912
Aldose reductase	0.5
Proto-oncogene tyrosine-protein kinase Src	0.505665446
Epidermal growth factor receptor	0.720974576
Beta-lactamase	0.209770115
Tyrosine-protein kinase JAK3	0.5659583
Thymidylate synthase	0.55721393
Fibroblast growth factor receptor 2	0.573688272
Casein kinase II subunit alpha	0.520751377
Acetylcholinesterase	1
Acetylcholinesterase	1
Serum albumin	0.409722222
cAMP-specific 3',5'-cyclic phosphodiesterase 4D	0.680782181
3-phosphoinositide-dependent protein kinase 1	0.38959276
Dual specificity protein kinase CLK1	0.466819222
Renin	0.659545898
Nitric oxide synthase, endothelial	0.409775504
Adenosine kinase	0.966666667

PROTEIN FAMILY	AUC
Cationic trypsin	0.661034434
Mast/stem cell growth factor receptor Kit	0.556216931
Arginase-1	0.214285714
cAMP-dependent protein kinase catalytic subunit alpha	0.464587584
Coagulation factor VII	0.815543629
Peroxisome proliferator-activated receptor alpha	0.226190476
Tryptase alpha/beta-1	0.272058824
Peptidyl-prolyl cis-trans isomerase FKBP1A	0.810443261
Coagulation factor X	0.543328098
Cathepsin K	0.48553128
Carbonic anhydrase 2	0.753685234
Cationic trypsin	0.64813476
Ephrin type-A receptor 3	0.525925926
Ribonuclease pancreatic	0.381987578
Cathepsin B	0.614700375
Nitric oxide synthase, inducible	0.638640373
Beta-lactamase	0.220679012
3-phosphoshikimate 1-carboxyvinyltransferase	0.666666667
Nitric oxide synthase, inducible	0.62594506
Dual specificity protein kinase CLK1	0.540331808
Angiotensin-converting enzyme	0.633831522
Macrophage migration inhibitory factor	1
Serine/threonine-protein kinase MARK2	0.578174603
Calcium/calmodulin-dependent protein kinase kinase 1	0.393867925
Serine/threonine-protein kinase Chk1	0.62254902
Calcium/calmodulin-dependent protein kinase kinase 1	0.492362983
Amine oxidase [flavin-containing] B	0.608953771
Corticosteroid 11-beta-dehydrogenase isozyme 1	0.56043956
Aurora kinase A	0.588845528
Cyclin-dependent kinase 9	0.349881094
Phosphatidylinositol 4,5-bisphosphate 3-kinase catalytic subunit alpha isoform	0.753411092
Mitogen-activated protein kinase 1	0.665118397
cAMP-specific 3',5'-cyclic phosphodiesterase 4D	0.731546232
Chymotrypsinogen A	0.751883562
Homeodomain-interacting protein kinase 2	0.370934959
Prothrombin	0.448328219
Beta-secretase 1	0.836821934
Mitogen-activated protein kinase 14	0.693196005
Peroxisome proliferator-activated receptor gamma	0.744704861
Prostasin	0.847826087
Liver carboxylesterase 1	0.421465844
Neuropeptide Y receptor type 2	0.083333333
TRAF2 and NCK-interacting protein kinase	0.46179402
Dipeptidyl peptidase 4	0.704063385
Urokinase-type plasminogen activator	0.566173332
3-phosphoshikimate 1-carboxyvinyltransferase	0.677777778
Peroxisome proliferator-activated receptor alpha	0.196864111
Phenylethanolamine N-methyltransferase	0.646139706
Dual specificity mitogen-activated protein kinase kinase 1	0.389941691
Vascular endothelial growth factor receptor 1	0.577267637
cAMP-specific 3',5'-cyclic phosphodiesterase 4C	0.589285714
Ephrin type-A receptor 2	0.489015152
Ribonuclease pancreatic	0.301242236

PROTEIN FAMILY	AUC
CREB-binding protein	0.666666667
Melanocortin receptor 5	0.674176747
Mitogen-activated protein kinase 14	0.637415037
3-phosphoshikimate 1-carboxyvinyltransferase	0.733333333
Chymase	0.973647712
Cathepsin B	0.653341103
Glycogen synthase kinase-3 beta	0.578677511
Histamine N-methyltransferase	0.833333333
Fibroblast growth factor receptor 2	0.491512346
Chymotrypsinogen A	0.646232877
Serine/threonine-protein kinase tousled-like 1	0.432973806
Urokinase-type plasminogen activator	0.567948249
Deoxyuridine 5'-triphosphate nucleotidohydrolase, mitochondrial	0.539083558
Lysozyme	0.87037037
Acetylcholinesterase	1
Queuine tRNA-ribosyltransferase	0.671428571
Casein kinase II subunit alpha	0.387340302
Insulin-like growth factor 1 receptor	0.637815126
Cyclin-dependent kinase 2	0.342091295
Gamma-aminobutyric acid receptor subunit alpha-6	0.58436853
Estrogen receptor beta	0.561574789
Beta-lactamase	0.201149425
Calcitonin gene-related peptide type 1 receptor	0.502938871
Activin receptor type-1B	0.598958333
Renin	0.802793561
Cytochrome P450 130	1
Serine/threonine-protein kinase pim-1	0.683772219
Glucocorticoid receptor	0.532845248
Dihydrofolate reductase	0.19486532
Aurora kinase A	0.576862228
Serine/threonine-protein kinase VRK2	0.487931034
Cationic trypsin	0.641829649
cAMP-specific 3',5'-cyclic phosphodiesterase 4B	0.861380798
Cationic trypsin	0.678535571
Glutamate carboxypeptidase 2	0.623376623
3-oxoacyl-[acyl-carrier-protein] synthase 3	1
Tyrosine-protein kinase Fyn	0.571601522
Urokinase-type plasminogen activator	0.548187181
Epidermal growth factor receptor	0.722595339
Bifunctional purine biosynthesis protein PURH	0.426470588
Serine/threonine-protein kinase 38-like	0.45390625
3-phosphoinositide-dependent protein kinase 1	0.384162896
Neutrophil collagenase	0.634650456
Retinoic acid receptor beta	0.698113208
Macrophage migration inhibitory factor	1
Serum albumin	0.534722222
Peroxisome proliferator-activated receptor alpha	0.231416957
Calcium/calmodulin-dependent protein kinase type II subunit gamma	0.388888889
Serine/threonine-protein kinase pim-1	0.708722844
cGMP-inhibited 3',5'-cyclic phosphodiesterase B	0.6
Tyrosine-protein kinase Fes/Fps	0.475315126
Kappa-type opioid receptor	0.556097974
Macrophage metalloelastase	0.350315126

PROTEIN FAMILY	AUC
Non-receptor tyrosine-protein kinase TYK2	0.563058896
Peroxisome proliferator-activated receptor gamma	0.78125
Plasminogen	0.390551564
Glutathione S-transferase P	0.775
Ribonuclease pancreatic	0.400621118
Fibroblast growth factor receptor 3	0.542888563
Heat shock protein HSP 90-alpha	0.853773585
Cyclin-dependent kinase 2	0.357647395
Cyclin-dependent kinase 8	0.635057471
Progesterone receptor	0.52595406
Dihydroorotate dehydrogenase (quinone), mitochondrial	0.678787879
Retinoic acid receptor RXR-alpha	0.647230321
Receptor-type tyrosine-protein phosphatase F	0.659090909
Histone deacetylase 6	0.29787234
Coagulation factor X	0.521775123
Kinesin-like protein KIF11	0.721153846
Phenylethanolamine N-methyltransferase	0.7546875
Methionine aminopeptidase 1	0.632246377
Tyrosine-protein kinase ABL1	0.579178461
Protein S100-B	0.166666667
Carbonic anhydrase 2	0.764381783
Fibroblast growth factor 1	0.367521368
Mitogen-activated protein kinase 1	0.694262295
Cyclin-dependent kinase 2	0.410399314
Orotidine 5'-phosphate decarboxylase	0.614285714
Dipeptidyl peptidase 4	0.709527594
Mitogen-activated protein kinase 8	0.518149883
Melatonin receptor type 1B	0.811468813
Thymidylate kinase	0.714285714
Urokinase-type plasminogen activator	0.546548526
Ribosomal protein S6 kinase beta-1	0.335847382
Transthyretin	0.714285714
Glutathione S-transferase A1	0.366666667
Tyrosine-protein kinase Fes/Fps	0.532620321
Thymidylate synthase	0.66955267
Estrogen receptor beta	0.731774559
Queuine tRNA-ribosyltransferase	0.714285714
Nischarin	0.658950617
Serum albumin	0.354166667
Urokinase-type plasminogen activator	0.566790777
Protein kinase C epsilon type	0.377586207
cAMP-dependent protein kinase catalytic subunit alpha	0.496939668
Heat shock protein HSP 90-beta	0.7
Multidrug resistance-associated protein 1	0.4375
Myosin light chain kinase 2, skeletal/cardiac muscle	0.563973064
3-hydroxy-3-methylglutaryl-coenzyme A reductase	0.639705882
Tyrosine-protein kinase Fes/Fps	0.485026738
Prothrombin	0.455459989
Caspase-3	0.839646465
Nuclear receptor subfamily 1 group I member 2	0
Beta-secretase 1	0.86276533
Multidrug resistance-associated protein 1	0.71875
cAMP-specific 3',5'-cyclic phosphodiesterase 4B	0.881607335

PROTEIN FAMILY	AUC
Glutamate carboxypeptidase 2	0.519480519
cAMP-specific 3',5'-cyclic phosphodiesterase 4B	0.878910464
Growth factor receptor-bound protein 2	0.492424242
Acetylcholinesterase	0.406666667
RAC-alpha serine/threonine-protein kinase	0.593231162
Urokinase-type plasminogen activator	0.550445671
Tyrosine-protein kinase JAK2	0.566843034
Cationic trypsin	0.651317684
Transthyretin	0.642857143
Mitogen-activated protein kinase 14	0.674954917
Papain	0.470913008
Cathepsin B	0.632490637
Epidermal growth factor receptor	0.749692027
Renin	0.783854167
Chaperone activity of bc1 complex-like, mitochondrial	0.474576271
Retinoic acid receptor RXR-gamma	0.509411765
Androgen receptor	0.599325447
Thermolysin	0.541081081
Phosphatidylinositol 4-phosphate 5-kinase type-1 alpha	0.603183962
Substance-K receptor	0.503220435
Insulin-like growth factor 1 receptor	0.592560554
Peptidyl-prolyl cis-trans isomerase NIMA-interacting 1	0.748917749
Coagulation factor X	0.517005021
Estrogen receptor	0.639084034
Estrogen receptor	0.719203061
Thermolysin	0.594594595
Abelson tyrosine-protein kinase 2	0.556689342
Dihydrofolate reductase	0.556376574
Metallo-beta-lactamase L1	0.80026455
Adenosine kinase	0.916666667
Heparanase	0.5
cAMP-specific 3',5'-cyclic phosphodiesterase 4B	0.881337648
Integrin alpha-L	0.817073171
Thymidylate synthase	0.647757911
Cyclin-dependent kinase 11A	0.527113238
Glutathione S-transferase P	0.725
Angiopoietin-1 receptor	0.634191176
Thyroid hormone receptor beta	0.172619048
Aldehyde oxidase	0.714285714
Tyrosine-protein kinase Lck	0.659584042
Misshapen-like kinase 1	0.562739464
Leukotriene A-4 hydrolase	0.833333333
Leukotriene A-4 hydrolase	1
Dual specificity protein kinase CLK3	0.455731225
Beta-secretase 1	0.842423349
Serine/threonine-protein kinase MAK	0.355889724
Ephrin type-A receptor 6	0.591768293
Mitogen-activated protein kinase 14	0.642495492
Mast/stem cell growth factor receptor Kit	0.571245369
Ephrin type-B receptor 4	0.543463612
Serine/threonine-protein kinase N1	0.51979638
Renin	0.705137311
BMP-2-inducible protein kinase	0.483512545

PROTEIN FAMILY	AUC
Bifunctional purine biosynthesis protein PURH	0.408823529
Peptidyl-prolyl cis-trans isomerase A	0.415847666
Transthyretin	0.928571429
Dual specificity mitogen-activated protein kinase kinase 3	0.510695187
Mitogen-activated protein kinase 14	0.682983077
Ephrin type-B receptor 4	0.549528302
Lactoylglutathione lyase	0.771498771
Prothrombin	0.435518228
Retinoic acid receptor alpha	0.747763158
Thymidine phosphorylase	0.403940887
N(G),N(G)-dimethylarginine dimethylaminohydrolase 1	0.222222222
Peptidyl-prolyl cis-trans isomerase NIMA-interacting 1	0.716931217
Macrophage-stimulating protein receptor	0.495531281
Farnesyl pyrophosphate synthase	0.780555556
Cyclin-dependent kinase 2	0.369569655
Serine/threonine-protein kinase STK11	0.567395264
Serine/threonine-protein kinase mTOR	0.669503012
Peptidyl-prolyl cis-trans isomerase A	0.922297297
Renin	0.643821023
Mast/stem cell growth factor receptor Kit	0.518240578
Epithelial discoidin domain-containing receptor 1	0.663875598
Calcium/calmodulin-dependent protein kinase type II subunit gamma	0.512605042
Glucocorticoid receptor	0.518633362
Tyrosine-protein phosphatase non-receptor type 1	0.716384752
Orotidine 5'-phosphate decarboxylase	0.421428571
Thymidylate synthase	0.584415584
Proto-oncogene tyrosine-protein kinase Src	0.575506716
Arginase-1	0.328571429
Beta-lactamase	0.193548387
Hormonally up-regulated neu tumor-associated kinase	0.546568627
Acetylcholinesterase	1
Aurora kinase A	0.499084967
Angiotensin-converting enzyme	0.6015625
Thymidylate synthase	0.655122655
Phosphatidylinositol 4-phosphate 3-kinase C2 domain-containing subunit gamma	0.523399015
Beta-lactamase	0.195402299
Thermolysin	0.606486486
Queuine tRNA-ribosyltransferase	0.614285714
Plasminogen	0.401940742
Cyclin-dependent kinase 11A	0.492946708
Serine/threonine-protein kinase PknB	0.423469388
Serine/threonine-protein kinase Chk1	0.643137255
Uridine 5'-monophosphate synthase	0.2
Beta-lactamase	0.206896552
Caspase-1	0.650121501
Dual specificity tyrosine-phosphorylation-regulated kinase 1B	0.470238095
Prothrombin	0.433515711
Phosphatidylinositol 4-phosphate 3-kinase C2 domain-containing subunit gamma	0.54679803
Aminopeptidase N	1
Ephrin type-A receptor 4	0.639615385
Dihydrofolate reductase	0.555408971
Endothelin B receptor	0.500601251
Cyclin-dependent kinase 18	0.537593985

PROTEIN FAMILY	AUC
Neutrophil collagenase	0.673279912
Beta-secretase 1	0.78515625
Thymidylate synthase	0.53814262
6-phosphogluconate dehydrogenase, decarboxylating	0.574074074
Receptor-interacting serine/threonine-protein kinase 4	0.481428571
Glucosylceramidase	0.773496241
Serine/threonine-protein kinase Chk2	0.628240741
Queuine tRNA-ribosyltransferase	0.685714286
Carboxypeptidase A1	0.204077061
Fibroblast growth factor receptor 1	0.698639456
Vascular endothelial growth factor receptor 2	0.590193705
Peptidyl-prolyl cis-trans isomerase A	0.904279279
Trypsin-3	0.745762712
Tyrosine-protein kinase receptor Tie-1	0.510080645
Heat shock protein HSP 90-alpha	0.877358491
Renin	0.758404356
Calcium/calmodulin-dependent protein kinase kinase 2	0.533333333
Amine oxidase [flavin-containing] B	0.597858881
Serine/threonine-protein kinase MAK	0.374269006
Dual specificity protein kinase CLK3	0.518181818
Misshapen-like kinase 1	0.474616858
Ribonuclease pancreatic	0.360248447
3-hydroxy-3-methylglutaryl-coenzyme A reductase	0.536764706
Protein kinase C zeta type	1
Nitric oxide synthase, inducible	0.63719128
Tryptase alpha/beta-1	0.192179144
Thymidylate synthase	0.612354892
Metabotropic glutamate receptor 3	0.509803922
Cationic trypsin	0.687427704
cAMP-dependent protein kinase catalytic subunit alpha	0.449285922
Hepatocyte growth factor receptor	0.629096195
Trypsin	0.958333333
Serine/threonine-protein kinase 17B	0.586939103
Peptidyl-prolyl cis-trans isomerase A	0.939189189
G protein-coupled receptor kinase 7	0.471889952
Proto-oncogene tyrosine-protein kinase Src	0.517606838
Peptidyl-prolyl cis-trans isomerase FKBP1A	0.784126628
cAMP-specific 3',5'-cyclic phosphodiesterase 4A	0.730576441
Serine/threonine-protein kinase Chk2	0.651600753
Serine/threonine-protein kinase B-raf	0.64317602
Fibroblast growth factor receptor 3	0.63691349
Peptidyl-prolyl cis-trans isomerase NIMA-interacting 1	0.651515152
Prothrombin	0.444893377
Calcium/calmodulin-dependent protein kinase type II subunit gamma	0.523634454
Papain	0.453137625
Growth factor receptor-bound protein 2	0.568181818
Tyrosine-protein kinase JAK2	0.590770135
ALK tyrosine kinase receptor	0.537997159
Calcium/calmodulin-dependent protein kinase kinase 2	0.555675287
Serum albumin	0.423611111
Acetylcholinesterase	1
SRSF protein kinase 1	0.558589871
Cyclin-dependent kinase 2	0.490160052

PROTEIN FAMILY	AUC
Orotidine 5'-phosphate decarboxylase	0.4
Insulin receptor	0.66934943
C-X-C chemokine receptor type 3	0.118644068
Cationic trypsin	0.656160356
Serum albumin	0.395833333
Tyrosine-protein kinase BTK	0.410027473
Macrophage migration inhibitory factor	0
Receptor tyrosine-protein kinase erbB-3	0.551020408
Tyrosine-protein phosphatase non-receptor type 1	0.673576638
Hepatocyte growth factor receptor	0.613812544
Heat shock protein HSP 90-alpha	0.865566038
Bromodomain-containing protein 4	0.125
Peptidyl-prolyl cis-trans isomerase NIMA-interacting 1	0.753727754
Tyrosine-protein kinase receptor UFO	0.540650407
Carbonic anhydrase 12	0.575651303
Myosin light chain kinase, smooth muscle	0.467003367
Tyrosine-protein kinase ZAP-70	0.625289352
Lanosterol 14-alpha demethylase	0.09375
Cholinesterase	0.831057251
Coagulation factor X	0.561794243
Dihydrofolate reductase	0.632517514
S-ribosylhomocysteine lyase	0.843406593
Gamma-aminobutyric acid receptor subunit beta-2	0.806875
Activin receptor type-1	0.6
Beta-secretase 1	0.774100825
Mu-type opioid receptor	0.528040465
Glyceraldehyde-3-phosphate dehydrogenase	0.714285714
Aldo-keto reductase family 1 member C1	0.875
Focal adhesion kinase 1	0.602941176
Metabotropic glutamate receptor 1	0.699519231
Insulin-like growth factor 1 receptor	0.618067227
Urokinase-type plasminogen activator	0.530338409
Estrogen receptor beta	0.662806986
Neuroendocrine convertase 2	0.5
Glutamate carboxypeptidase 2	0.772727273
Gamma-aminobutyric acid receptor subunit beta-3	0.568940662
Activin receptor type-2A	0.449776786
Serine/threonine-protein kinase Chk1	0.628358209
Macrophage migration inhibitory factor	1
Transthyretin	0.714285714
Calcium/calmodulin-dependent protein kinase kinase 1	0.452358491
Gamma-aminobutyric acid receptor subunit alpha-2	0.541656663
Estrogen receptor	0.654186057
Activated CDC42 kinase 1	0.663840459
cAMP-specific 3',5'-cyclic phosphodiesterase 4D	0.551282051
Dihydrofolate reductase	0.56646347
Alcohol dehydrogenase E chain	0.316666667
Receptor tyrosine-protein kinase erbB-3	0.469444444
Abelson tyrosine-protein kinase 2	0.547052154
Cathepsin B	0.596910112
Cyclin-dependent kinase 19	0.575
Beta-lactamase	0.206790123
Ephrin type-A receptor 7	0.554638365

PROTEIN FAMILY	AUC
Estrogen receptor beta	0.581425846
Serine/threonine-protein kinase PRP4 homolog	nan
Vascular endothelial growth factor receptor 3	0.604110208
Coagulation factor X	0.531397439
Papain	0.389711823
Urokinase-type plasminogen activator	0.538794174
Cationic trypsin	0.661718302
C-C chemokine receptor type 8	0.773484848
Peptidyl-prolyl cis-trans isomerase FKBP1A	0.789242415
STE20-like serine/threonine-protein kinase	0.582879613
Peptidyl-prolyl cis-trans isomerase FKBP1A	0.769019822
Peroxisome proliferator-activated receptor gamma	0.724826389
Cathepsin B	0.602887853
Myosin light chain kinase, smooth muscle	0.414814815
Cyclin-dependent kinase 15	0.469834087
Receptor-interacting serine/threonine-protein kinase 4	0.512142857
Cyclin-dependent kinase 4	0.490686275
Serine/threonine-protein kinase LATS2	0.514467593
Heat shock protein HSP 90-alpha	0.877358491
Focal adhesion kinase 1	0.483333333
Ephrin type-A receptor 8	0.533015407
Cyclin-dependent kinase 2	0.482790299
Prostaglandin E2 receptor EP3 subtype	0.585965502
Chymotrypsinogen A	0.610518293
3-dehydroquinate dehydratase	1
Myosin light chain kinase, smooth muscle	0.538047138
Protein-tyrosine kinase 2-beta	0.606811146
Retinoic acid receptor RXR-alpha	0.541423712
MAP kinase-activated protein kinase 2	0.73030303
cAMP-dependent protein kinase catalytic subunit alpha	0.490964733
Ephrin type-A receptor 3	0.582098765
Nociceptin receptor	0.402133737
Cellular retinoic acid-binding protein 1	0.388888889
Serum albumin	0.4375
Protein S100-B	0.5
Cationic trypsin	0.651397516
Bromodomain-containing protein 4	0.75
Dual specificity protein phosphatase 3	0.464285714
T-cell surface glycoprotein CD4	0.75
Coagulation factor VII	0.702593918
Fatty acid-binding protein, intestinal	0.2
Purine nucleoside phosphorylase	0.505337838
Mu-type opioid receptor	0.567206707
Hepatocyte growth factor receptor	0.690335306
Lysophosphatidic acid receptor 3	0.209090909
3-hydroxy-3-methylglutaryl-coenzyme A reductase	0.720588235
Cyclin-dependent kinase 18	0.523391813
Serine/threonine-protein kinase B-raf	0.630420918
Fatty-acid amide hydrolase 1	0.573825503
Glutathione S-transferase P	0.75
Ephrin type-A receptor 1	0.598447712
Gamma-aminobutyric acid receptor subunit pi	0.333333333
Chymase	0.851595007

PROTEIN FAMILY	AUC
Seminal ribonuclease	0.666666667
Acetylcholinesterase	1
Estrogen receptor beta	0.721313186
Prothrombin	0.437628371
Caspase-8	0.582417582
Protein S100-B	0.166666667
Mitogen-activated protein kinase kinase kinase 15	0.57748538
Ribonuclease pancreatic	0.472049689
Prothrombin	0.570733104
Inositol-trisphosphate 3-kinase A	0.5
Prothrombin	0.452627046
Caspase-5	0.95
Phenylethanolamine N-methyltransferase	0.481893382
Carbonic anhydrase 1	0.712283123
Glutamate carboxypeptidase 2	0.619047619
Vitamin D3 receptor	0.224489796
Progesterone receptor	0.575583288
Beta-lactamase	0.192727273
Cationic trypsin	0.655411255
Serum albumin	0.548611111
Nitric oxide synthase, inducible	0.627709173
Amine oxidase [flavin-containing] B	0.604671533
Serine/threonine-protein kinase Nek2	0.530769231
Cyclin-dependent kinase 5	0.50392773
Eukaryotic translation initiation factor 2-alpha kinase 4	0.423245614
Cholinesterase	0.815612297
Bacterial leucyl aminopeptidase	0.181818182
Disintegrin and metalloproteinase domain-containing protein 17	0.606252618
Serum albumin	0.479166667
Peptidyl-prolyl cis-trans isomerase FKBP1A	0.789466932
Tyrosine-protein kinase Mer	0.616174056
Cholinesterase	0.840256606
Caspase-8	0.747252747
Guanine deaminase	0.444444444
Estrogen receptor beta	0.633822127
Serine/threonine-protein kinase DCLK1	0.62208657
Dual specificity mitogen-activated protein kinase kinase 4	0.477463312
Death-associated protein kinase 1	0.545061728
Dihydrofolate reductase	0.354532164
Cyclin-dependent kinase 1	0.782476506
Chymotrypsinogen A	0.645205479
Myotonin-protein kinase	0.56462585
Nitric oxide synthase, endothelial	0.399829497
UDP-3-O-[3-hydroxymyristoyl] N-acetylglucosamine deacetylase	0.125
cAMP-specific 3',5'-cyclic phosphodiesterase 4A	0.71679198
Death-associated protein kinase 1	0.50969697
Arginase-1	0.271428571
Myosin-IIIa	0.52003643
Plasma kallikrein	0.580645161
Transthyretin	0.785714286
Peptidyl-prolyl cis-trans isomerase A	0.920045045
Corticotropin-releasing factor receptor 2	nan
Cruzipain	0.955555556

PROTEIN FAMILY	AUC
Hepatocyte growth factor receptor	0.58641649
Serum albumin	0.4375
Heat shock protein HSP 90-alpha	0.841981132
Glycogen synthase kinase-3 beta	0.60314268
Serum albumin	0.340277778
Beta-1,4-galactosyltransferase 1	0.53125
Androgen receptor	0.454223287
Coagulation factor X	0.478463151
Cationic trypsin	0.628952569
3-phosphoshikimate 1-carboxyvinyltransferase	0.588888889
Prothrombin	0.42794827
Macrophage colony-stimulating factor 1 receptor	0.635962493
Platelet-activating factor receptor	0.613580247
Lanosterol 14-alpha demethylase	0.1875
Mitogen-activated protein kinase 11	0.643442623
Tyrosine-protein phosphatase non-receptor type 1	0.634736778
Coagulation factor X	0.578011689
Coagulation factor XI	0.441176471
Nitric oxide synthase, endothelial	0.427536232
Serine/threonine-protein kinase PLK1	0.489304813
Cyclin-dependent kinase 2	0.348358648
Carbonic anhydrase 2	0.770741312
cGMP-specific 3',5'-cyclic phosphodiesterase	0.908045977
Macrophage metalloelastase	0.346769958
Cathepsin S	0.714644636
Cathepsin K	0.479371764
Aldose reductase	0.666666667
Receptor-interacting serine/threonine-protein kinase 4	0.566526611
Retinoic acid receptor RXR-alpha	0.606656948
Serine/threonine-protein kinase pim-1	0.711340206
Carbonic anhydrase 2	0.764522719
Cyclin-dependent kinase 2	0.354197289
Fatty acid-binding protein, adipocyte	0.424342105
Peptidyl-prolyl cis-trans isomerase NIMA-interacting 1	0.737614238
Bromodomain-containing protein 4	0
Dipeptidyl peptidase 4	0.6918931
Prostasin	0.869565217
Cyclin-dependent kinase 3	0.559027778
Thermolysin	0.583783784
Cationic trypsin	0.634355435
Mitogen-activated protein kinase 1	0.69298725
Eukaryotic translation initiation factor 2-alpha kinase 4	0.409297052
Hepatocyte growth factor receptor	0.615133897
Dipeptidyl peptidase 4	0.706596791
Carbonic anhydrase 2	0.76423768
cGMP-specific 3',5'-cyclic phosphodiesterase	0.916256158
Prothrombin	0.527260763
Caspase-1	0.739357394
WD repeat-containing protein 5	0.565217391
Peptidyl-prolyl cis-trans isomerase FKBP1A	0.555263327
cAMP-dependent protein kinase catalytic subunit alpha	0.570824832
Dual specificity tyrosine-phosphorylation-regulated kinase 2	0.53875969
Aldose reductase	0.444444444

PROTEIN FAMILY	AUC
Proto-oncogene tyrosine-protein kinase Src	0.51015873
Mitogen-activated protein kinase 10	0.50465368
cAMP-dependent protein kinase catalytic subunit alpha	0.41926552
Methionine aminopeptidase 2	0.506859756
Casein kinase I isoform alpha	0.666883117
Dual specificity protein kinase TTK	0.514375877
Methionine aminopeptidase 1	0.759057971
Papain	0.499596014
Beta-secretase 1	0.816774764
Caspase-8	0.736263736
Egl nine homolog 1	0.381818182
Peroxisome proliferator-activated receptor gamma	0.78984375
Lactoylglutathione lyase	0.749078624
Dihydrofolate reductase	0.553179874
Myotonin-protein kinase	0.409135083
Steroid hormone receptor ERR1	1
Thymidylate synthase	0.642135642
Squalene synthase	0.840909091
Chymotrypsinogen A	0.708113804
Glutamate receptor ionotropic, NMDA 2B	0.297496318
Glycogen synthase kinase-3 beta	0.577682992
Somatostatin receptor type 4	0.626571961
Serine/threonine-protein kinase BRSK2	0.366393443
Mitogen-activated protein kinase 10	0.549780702
Phosphatidylinositol 4,5-bisphosphate 3-kinase catalytic subunit gamma isoform	0.635057471
Prolyl endopeptidase	0.290909091
Neutrophil elastase	0.553981938
Prothrombin	0.432127331
Mitogen-activated protein kinase 14	0.651737412
Mitogen-activated protein kinase 10	0.497294372
Androgen receptor	0.409265324
Tyrosine-protein kinase Lck	0.674682532
Glutamate carboxypeptidase 2	0.632034632
Coagulation factor X	0.569100242
Transthyretin	0.857142857
cAMP-specific 3',5'-cyclic phosphodiesterase 4D	0.697487697
Sodium- and chloride-dependent glycine transporter 1	0.575757576
Tyrosine-protein kinase ABL1	1
Kynureninase	0.357142857
Bone morphogenetic protein 1	0.66779661
Glutathione reductase, mitochondrial	0.540740741
Serine/threonine-protein kinase pim-1	0.665668203
Transthyretin	0.821428571
Beta-secretase 1	0.818322524
Peptidyl-prolyl cis-trans isomerase A	0.927364865
Arginase-1	0.271428571
3-phosphoinositide-dependent protein kinase 1	0.381900452
Tyrosine-protein kinase BTK	0.429601648
Cathepsin K	0.56288497
Cathepsin B	0.611423221
Beta-secretase 1	0.815669222
Vascular endothelial growth factor receptor 2	0.608561606
Transient receptor potential cation channel subfamily M member 6	0.393442623

PROTEIN FAMILY	AUC
Tyrosine-protein phosphatase non-receptor type 1	0.697493412
Growth factor receptor-bound protein 2	0.628787879
Orotidine 5'-phosphate decarboxylase	0.428571429
Tyrosine-protein kinase ABL1	0.598958156
Acetylcholinesterase	0.46
Coagulation factor VII	0.711886305
Dipeptidyl peptidase 4	0.711067508
Bone morphogenetic protein receptor type-2	0.637426901
MAP/microtubule affinity-regulating kinase 4	0.622410546
Chaperone activity of bc1 complex-like, mitochondrial	0.447557471
Lethal(3)malignant brain tumor-like protein 1	0.083333333
Thermolysin	0.614054054
Liver carboxylesterase 1	0.458191931
Carbonic anhydrase 2	0.766690596
Glutamate carboxypeptidase 2	0.463203463
Carbonic anhydrase 2	0.755356984
Death-associated protein kinase 2	0.524767802
Amine oxidase [flavin-containing] B	0.615766423
Vascular endothelial growth factor receptor 2	0.623676743
Dual specificity mitogen-activated protein kinase kinase 5	0.643004115
Serine/threonine-protein kinase PLK1	0.402215432
WD repeat-containing protein 5	0.467391304
Coagulation factor X	0.580120854
Homeodomain-interacting protein kinase 3	0.512820513
Glycogen phosphorylase, muscle form	0.285714286
Phenylethanolamine N-methyltransferase	0.722886029
Farnesyl pyrophosphate synthase	0.622222222
Seminal ribonuclease	1
Dihydrofolate reductase	0.59318555
Protein S100-B	0
Pteridine reductase 1	0.608225108
Tyrosine-protein kinase Lck	0.661433857
Cationic trypsin	0.627008032
Purine nucleoside phosphorylase	0.470238095
Endochitinase	0.666666667
Farnesyl pyrophosphate synthase	0.621527778
Mitogen-activated protein kinase 6	0.439964158
Tyrosine-protein kinase CSK	0.513062409
Retinoic acid receptor gamma	0.573566514
cAMP-dependent protein kinase catalytic subunit alpha	0.411104634
Mitogen-activated protein kinase 1	0.685428051
Insulin-like growth factor 1 receptor	0.672689076
Macrophage metalloelastase	0.442095588
Collagenase 3	0.467627329
Peptidyl-prolyl cis-trans isomerase A	0.898648649
Mitogen-activated protein kinase kinase kinase 15	0.42816092
MAP kinase-activated protein kinase 2	0.578624079
Hepatocyte growth factor receptor	0.470181466
Serum albumin	0.326388889
Gamma-aminobutyric acid receptor subunit alpha-5	0.604213483
Prolyl endopeptidase	0.378409091
Queuine tRNA-ribosyltransferase	0.657142857
Caspase-3	0.943181818

PROTEIN FAMILY	AUC
Beta-secretase 1	0.850825472
Thymidine phosphorylase	0.556650246
Beta-lactamase	0.22
Heat shock protein HSP 90-alpha	0.882075472
Alcohol dehydrogenase E chain	0.233333333
Peptidyl-prolyl cis-trans isomerase FKBP1A	0.803387004
Fatty acid-binding protein, heart	0.376373626
cAMP-dependent protein kinase catalytic subunit alpha	0.435004372
Serum albumin	0.326388889
Lethal(3)malignant brain tumor-like protein 1	0.291666667
TRAF2 and NCK-interacting protein kinase	0.531179138
Leukotriene A-4 hydrolase	1
Chymase	0.747572816
Fatty acid-binding protein, heart	0.357142857
Cationic trypsin	0.653729839
Cruzipain	0.988888889
3-phosphoshikimate 1-carboxyvinyltransferase	0.633333333
Estrogen receptor	0.701874888
Trifunctional purine biosynthetic protein adenosine-3	0.728618421
Fatty acid-binding protein, adipocyte	0.858552632
Inosine-5'-monophosphate dehydrogenase 2	0.796511628
Estrogen receptor	0.690397351
Thymidylate kinase	0.699341754
Serine/threonine-protein kinase Nek3	0.375
Platelet-derived growth factor receptor alpha	0.598006645
Stromelysin-2	0.24375
Retinoic acid receptor gamma	0.706422018
Coagulation factor VII	0.732397232
Corticosteroid 11-beta-dehydrogenase isozyme 1	0.603896104
Receptor-type tyrosine-protein kinase FLT3	0.537666135
Tyrosine-protein phosphatase non-receptor type 1	0.656156156
Carbonic anhydrase 2	0.762691691
Insulin receptor-related protein	0.56505102
Fibroblast growth factor receptor 2	0.489583333
Dual specificity protein kinase CLK3	0.559469697
Kinesin-like protein KIF11	0.336538462
Eukaryotic translation initiation factor 2-alpha kinase 4	0.503401361
Estrogen receptor	0.68284634
Epidermal growth factor receptor	0.729692797
Coagulation factor X	0.517047139
Proto-oncogene tyrosine-protein kinase receptor Ret	0.553095815
Tryptase alpha/beta-1	0.19986631
Nischarin	0.464506173
Retinoic acid receptor RXR-alpha	0.565719145
Lethal(3)malignant brain tumor-like protein 1	0
Serine/threonine-protein kinase MARK1	0.578912467
Estrogen receptor	0.685654197
Uridine 5'-monophosphate synthase	0.25
Cationic trypsin	0.6595214
Dipeptidyl peptidase 4	0.673463812
Maltase-glucoamylase, intestinal	0.133333333
Peroxisome proliferator-activated receptor gamma	0.7296875
Rho-associated protein kinase 1	0.407833333

PROTEIN FAMILY	AUC
Coagulation factor X	0.577441077
Amine oxidase [flavin-containing] B	0.618783455
Cyclin-dependent kinase 2	0.363220643
Activin receptor type-1	0.669753086
Lactoylglutathione lyase	0.606572482
Macrophage-stimulating protein receptor	0.553459119
Orotidine 5'-phosphate decarboxylase	0.385714286
Cathepsin L1	0.647824897
Sodium- and chloride-dependent glycine transporter 1	0.818181818
cAMP-specific 3',5'-cyclic phosphodiesterase 4B	0.867044229
Dihydrofolate reductase	0.591984351
Coagulation factor IX	0.404142582
Mitogen-activated protein kinase 14	0.636114579
UDP-3-O-[3-hydroxymyristoyl] N-acetylglucosamine deacetylase	0.75
Tyrosine-protein kinase JAK2	0.569606114
Thymidylate synthase	0.566378066
Phosphatidylinositol 4,5-bisphosphate 3-kinase catalytic subunit gamma isoform	0.594827586
RAF proto-oncogene serine/threonine-protein kinase	0.621925134
Estrogen receptor	0.702736263
Estrogen receptor	0.536804188
TRAF2 and NCK-interacting protein kinase	0.676870748
Multidrug resistance-associated protein 1	0.21875
UDP-3-O-[3-hydroxymyristoyl] N-acetylglucosamine deacetylase	0.875
Cathepsin S	0.690334993
Retinoic acid receptor RXR-alpha	0.564747328
Dihydrofolate reductase	0.657810936
6,7-dimethyl-8-ribityllumazine synthase	0.418367347
Serine/threonine-protein kinase N2	0.483258929
Carbonic anhydrase 5B, mitochondrial	0.534040369
Glutathione S-transferase A1	0.3
Cyclin-dependent kinase 1	0.614980652
Suppressor of tumorigenicity 14 protein	0.33490566
MAP kinase-activated protein kinase 2	0.723636364
Peptidyl-prolyl cis-trans isomerase A	0.946509009
Thymidine kinase, cytosolic	0.969230769
Cationic trypsin	0.63431677
Tryptase alpha/beta-1	0.156417112
3-dehydroquinate dehydratase	0.9
Transthyretin	1
Retinoic acid receptor RXR-alpha	0.631681244
Uridine 5'-monophosphate synthase	0.15
Alcohol dehydrogenase class 4 mu/sigma chain	0.533333333
Stromelysin-1	0.429828141
3-dehydroquinate dehydratase	0.85
Serine/threonine-protein kinase PknB	0.506559767
Orotidine 5'-phosphate decarboxylase	0.464285714
Serine/threonine-protein kinase Nek2	0.487692308
Proto-oncogene tyrosine-protein kinase Src	0.543125763
Maltase-glucoamylase, intestinal	0.133333333
Fibroblast growth factor receptor 4	0.627232143
Ephrin type-A receptor 8	0.608950844
MAP kinase-interacting serine/threonine-protein kinase 1	0.525757576
Bone morphogenetic protein 1	0.801694915

PROTEIN FAMILY	AUC
Queuine tRNA-ribosyltransferase	0.628571429
Aldose reductase	0.472222222
Fibroblast growth factor receptor 1	0.684013605
Peptidyl-prolyl cis-trans isomerase A	0.899211712
Heat shock protein HSP 90-alpha	0.856132075
Tryptase delta	0.498263889
Acetylcholinesterase	0.446666667
Prothrombin	0.417202268
Dual specificity mitogen-activated protein kinase kinase 4	0.474279835
Tyrosine-protein kinase Lyn	0.669148936
Aurora kinase A	0.545821138
Prostaglandin G/H synthase 1	0.6
Prostaglandin G/H synthase 1	0.7
Dual specificity tyrosine-phosphorylation-regulated kinase 1A	0.461591221
Calcium/calmodulin-dependent protein kinase kinase 2	0.474497126
Thyroid hormone receptor beta	0.051739927
Cyclin-dependent kinase 2	0.355115956
Cationic trypsin	0.632834557
Myotonin-protein kinase	0.370262391
Tyrosine-protein phosphatase non-receptor type 1	0.587562052
Queuine tRNA-ribosyltransferase	0.685714286
3-hydroxy-3-methylglutaryl-coenzyme A reductase	0.772058824
Transthyretin	0.678571429
Nitric oxide synthase, inducible	0.643617692
Prostacyclin receptor	0.791322073
Mitogen-activated protein kinase kinase kinase kinase 5	0.5
Kinesin-like protein KIF11	0.836538462
5-hydroxytryptamine receptor 5A	0.649806076
Angiotensin-converting enzyme	0.556046196
Carbonic anhydrase 2	0.761444301
3-dehydroquinate dehydratase	0.861111111
Nitric oxide synthase, inducible	0.657699093
Glycogen phosphorylase, muscle form	0.285714286
Beta-lactamase	0.21577381
Proto-oncogene tyrosine-protein kinase Src	0.713675214
Mast/stem cell growth factor receptor Kit	0.51114702
Cyclin-dependent kinase 13	0.418003565
Baculoviral IAP repeat-containing protein 3	1
Proto-oncogene tyrosine-protein kinase Src	0.552405372
Peroxisome proliferator-activated receptor gamma	0.77734375
Peptidyl-prolyl cis-trans isomerase A	0.968468468
Serine/threonine-protein kinase DCLK3	0.447271045
Urokinase-type plasminogen activator	0.556446944
Serine/threonine-protein kinase 35	0.422222222
Epidermal growth factor receptor	0.722129237
Coagulation factor X	0.534190498
Serum albumin	0.381944444
Peroxisomal N(1)-acetyl-spermine/spermidine oxidase	1
Cruzipain	0.966666667
Cruzipain	0.988888889
Serine/threonine-protein kinase MRCK alpha	0.683060109
Ephrin type-A receptor 3	0.511419753
Tyrosine-protein kinase ZAP-70	0.613462669

PROTEIN FAMILY	AUC
Phosphatidylinositol 4,5-bisphosphate 3-kinase catalytic subunit gamma isoform	0.561781609
Cyclin-dependent kinase 2	0.344418586
Tyrosine-protein phosphatase non-receptor type 1	0.632116811
Dipeptidyl peptidase 4	0.69097412
Farnesyl pyrophosphate synthase	0.517361111
Trypsin	0.875
Acidic mammalian chitinase	1
Prothrombin	0.532047592
Proto-oncogene tyrosine-protein kinase Src	0.487692308
Phenylethanolamine N-methyltransferase	0.527113971
Coagulation factor XI	0.58496732
Protein kinase C beta type	0.414545455
6,7-dimethyl-8-ribityllumazine synthase	0.428571429
Integrin alpha-IIb	0.43768997
Serum albumin	0.354166667
SRSF protein kinase 3	0.491864831
Phosphatidylinositol 4,5-bisphosphate 3-kinase catalytic subunit gamma isoform	0.597701149
Arginase-1	0.357142857
Dual specificity protein kinase CLK3	0.542045455
Purine nucleoside phosphorylase	0.327380952
Macrophage metalloelastase	0.392463235
3-phosphoshikimate 1-carboxyvinyltransferase	0.6
Coagulation factor X	0.565953341
Disintegrin and metalloproteinase domain-containing protein 17	0.556556347
Leukotriene A-4 hydrolase	1
Serum albumin	0.4375
Fatty-acid amide hydrolase 1	0.536475051
Peptidyl-prolyl cis-trans isomerase A	0.913851351
Macrophage migration inhibitory factor	0.9375
Coagulation factor X	0.546393526
Prostaglandin G/H synthase 1	0.6
Dihydrofolate reductase	0.610044582
Adenosine kinase	0.966666667
Cationic trypsin	0.658864542
cAMP-dependent protein kinase catalytic subunit alpha	0.385601865
Aurora kinase A	0.537145682
Amine oxidase [flavin-containing] A	0.617667598
Cytochrome P450 130	1
Interleukin-1 receptor-associated kinase 1	0.519444444
Glutamate receptor ionotropic, NMDA 1	0.434782609
Fatty-acid amide hydrolase 1	0.558943683
Peptidyl-prolyl cis-trans isomerase NIMA-interacting 1	0.775372775
Serum albumin	0.4375
Myosin light chain kinase 2, skeletal/cardiac muscle	0.526895944
Serine/threonine-protein kinase N1	0.421945701
Thymidylate synthase	0.664502165
Ephrin type-A receptor 6	0.4875
Proto-oncogene tyrosine-protein kinase Src	0.549499389
Serine/threonine-protein kinase TBK1	0.685625
Sodium channel protein type 4 subunit alpha	0.576923077
Serine/threonine-protein kinase pim-1	0.685055958
Fatty acid-binding protein, adipocyte	0.865131579
Receptor tyrosine-protein kinase erbB-4	0.5315

PROTEIN FAMILY	AUC
Androgen receptor	0.424907127
Thymidylate synthase	0.589466089
Cationic trypsin	0.68489734
Beta-1,4-galactosyltransferase 1	1
Tyrosine-protein phosphatase non-receptor type 1	0.650931544
Dual specificity mitogen-activated protein kinase kinase 2	0.555458769
Dehydrosqualene synthase	0.652777778
Ribosyldihydronicotinamide dehydrogenase [quinone]	0.1
Mast/stem cell growth factor receptor Kit	0.591997152
Beta-2 adrenergic receptor	0.562280306
Glutathione S-transferase P	0.75
Bifunctional dihydrofolate reductase-thymidylate synthase	0.512130638
Activated CDC42 kinase 1	0.416504382
Beta-secretase 1	0.81928066
Aurora kinase A	0.55408867
Serine/threonine-protein kinase NLK	0.599137931
TGF-beta receptor type-1	0.623960499
Trypsin	0.441666667
Phosphatidylinositol 4-kinase beta	0.410514173
NUAK family SNF1-like kinase 2	0.497377622
Serine/threonine-protein kinase MARK2	0.463095238
Hepatocyte growth factor receptor	0.593243481
Abelson tyrosine-protein kinase 2	0.371598639
Ephrin type-A receptor 3	0.507407407
Cationic trypsin	0.630486231
Eukaryotic translation initiation factor 2-alpha kinase 4	0.403628118
Calcium/calmodulin-dependent protein kinase type II subunit beta	0.334615385
Serum albumin	0.395833333
5-hydroxytryptamine receptor 5A	0.580687222
Transthyretin	0.821428571
SRSF protein kinase 3	0.514392991
Mitogen-activated protein kinase 9	0.593091335
Cyclin-dependent kinase 2	0.333333333
Dihydrofolate reductase	0.592029843
Cationic trypsin	0.662563987
Collagenase 3	0.454409938
Deoxyuridine 5'-triphosphate nucleotidohydrolase, mitochondrial	0.617924528
Mitogen-activated protein kinase kinase kinase 3	0.419936373
Death-associated protein kinase 1	0.532121212
Heat shock protein HSP 90-alpha	0.851415094
Carboxypeptidase B2	0.75862069
Tryptophan 2,3-dioxygenase	1
Aldose reductase	0.458333333
Type II inositol 1,4,5-trisphosphate 5-phosphatase	0.375
Cationic trypsin	0.671402282
Glycogen phosphorylase, liver form	0
Wee1-like protein kinase	0.586904762
E3 ubiquitin-protein ligase XIAP	0.596257796
Ribosyldihydronicotinamide dehydrogenase [quinone]	0.172222222
Cyclin-dependent kinase 13	0.38057041
Fatty-acid amide hydrolase 1	0.541387025
Transthyretin	0.821428571
Receptor tyrosine-protein kinase erbB-2	0.373529412

PROTEIN FAMILY	AUC
Peroxisome proliferator-activated receptor delta	0.582010582
ALK tyrosine kinase receptor	0.55755132
Thymidylate kinase	0.732142857
Tyrosine-protein kinase ABL1	0.526098236
Serum albumin	0.368055556
Cyclin-dependent kinase 2	0.33966193
Calcium/calmodulin-dependent protein kinase kinase 1	0.566295547
Thermolysin	0.571891892
Beta-secretase 1	0.854068396
Methionine aminopeptidase 2	0.512195122
Cationic trypsin	0.64183371
Receptor tyrosine-protein kinase erbB-4	0.569387755
Angiopoietin-1 receptor	0.677696078
Interleukin-1 receptor-associated kinase 4	0.625409836
MAP kinase-interacting serine/threonine-protein kinase 2	0.539399093
Prolyl endopeptidase	0.297727273
3-phosphoinositide-dependent protein kinase 1	0.354298643
Bcl-2-like protein 1	0.757257257
Serine/threonine-protein kinase Chk1	0.617910448
Macrophage migration inhibitory factor	0.8125
Serine/threonine-protein kinase PRP4 homolog	0.63312369
Chymotrypsinogen A	0.622916667
cAMP-specific 3',5'-cyclic phosphodiesterase 4C	0.723214286
Serine/threonine-protein kinase MRCK alpha	0.50273224
Leukotriene A-4 hydrolase	1
Trypanothione reductase	0.799347826
Receptor-type tyrosine-protein phosphatase C	0.842592593
Cationic trypsin	0.657845668
Serine/threonine-protein kinase PRP4 homolog	0.613207547
Cyclin-dependent kinase 2	0.377266046
Tyrosine-protein kinase FRK	0.68577431
Transient receptor potential cation channel subfamily V member 1	0.71640625
Peroxisome proliferator-activated receptor alpha	0.345238095
Cationic trypsin	0.615748709
Coagulation factor X	0.538422715
Nitric oxide synthase, endothelial	0.450269963
Tyrosine-protein kinase JAK2	0.591005291
Serine/threonine-protein kinase Chk2	0.665740741
Casein kinase I isoform gamma-3	0.439655172
Protein kinase C gamma type	0.525925926
Prothrombin	0.434671873
Serine/threonine-protein kinase Chk1	0.594852941
Serine/threonine-protein kinase PLK4	0.61546724
Serine/threonine-protein kinase Chk1	0.63681592
Beta-secretase 1	0.832473467
Nitric oxide synthase, inducible	0.640625
Liver carboxylesterase 1	0.40575509
Abelson tyrosine-protein kinase 2	0.381235828
Heat shock protein HSP 90-alpha	0.883254717
Serine/threonine-protein kinase Chk1	0.620098039
Lysine-specific histone demethylase 1A	0.368421053
Cationic trypsin	0.63238669
Glutathione S-transferase P	0.9

PROTEIN FAMILY	AUC
Aminopeptidase N	1
Macrophage colony-stimulating factor 1 receptor	0.588527303
Baculoviral IAP repeat-containing protein 2	0.4
Tankyrase-2	1
Vascular endothelial growth factor receptor 1	0.575607903
Tyrosine-protein kinase ABL1	0.54290038
Cationic trypsin	0.623214286
S-adenosylmethionine decarboxylase proenzyme	0.509090909
Coagulation factor XI	0.513071895
Phosphoglycerate kinase	0.6875
Thymidylate synthase	0.501683502
Beta-1,4-galactosyltransferase 1	0.53125
Urokinase-type plasminogen activator	0.564912817
Ephrin type-B receptor 4	0.574414716
Dual specificity tyrosine-phosphorylation-regulated kinase 1A	0.579124579
Carboxypeptidase A1	0.163978495
Cathepsin D	0.529105691
Leucine-rich repeat serine/threonine-protein kinase 2	0.530885781
Mitogen-activated protein kinase 14	0.444321689
Peroxisome proliferator-activated receptor gamma	0.494857043
Tyrosine-protein phosphatase non-receptor type 1	0.634108598
Mitogen-activated protein kinase 1	0.672404372
Peptidyl-prolyl cis-trans isomerase A	0.90990991
Insulin-like growth factor 1 receptor	0.569747899
Fibroblast growth factor 1	0.388888889
Suppressor of tumorigenicity 14 protein	0.347484277
Acetylcholinesterase	0.406666667
Gamma-aminobutyric acid receptor subunit alpha-5	0.534711075
Beta-1,4-galactosyltransferase 1	1
Tyrosine-protein phosphatase non-receptor type 1	0.642382178
Casein kinase I isoform gamma-3	0.45146379
Papain	0.496498788
Serine/threonine-protein kinase ULK2	0.459949937
Cationic trypsin	0.678786575
Fibroblast growth factor receptor 1	0.626870748
Kinesin-like protein KIF11	0.634615385
Baculoviral IAP repeat-containing protein 3	nan
Serine/threonine-protein kinase Nek11	0.441326531
Mitogen-activated protein kinase 14	0.699230129
Cholinesterase	0.828836382
3-phosphoinositide-dependent protein kinase 1	0.377828054
Serine/threonine-protein kinase Chk1	0.655147059
Fibroblast growth factor receptor 2	0.577323718
Urokinase-type plasminogen activator	0.55505248
Cathepsin K	0.46986049
3-oxoacyl-[acyl-carrier-protein] synthase 3	1
Mitogen-activated protein kinase kinase kinase 15	0.381578947
Cholinesterase	0.830284553
Peroxisome proliferator-activated receptor alpha	0.236062718
Cyclin-dependent kinase 2	0.367548587
Aurora kinase A	0.52595122
Amine oxidase [flavin-containing] B	0.628029197
Serine/threonine-protein kinase Chk2	0.600925926

PROTEIN FAMILY	AUC
Carbonic anhydrase 1	0.692456694
Urokinase-type plasminogen activator	0.555547697
D(3) dopamine receptor	0.540661545
Vascular endothelial growth factor receptor 2	0.65405569
Maternal embryonic leucine zipper kinase	0.43125
Methionine aminopeptidase 2	0.597306911
Tyrosine-protein kinase ABL1	0.564795553
Tyrosine-protein kinase ABL1	0.519839375
cAMP-specific 3',5'-cyclic phosphodiesterase 4B	0.886461704
Aurora kinase A	0.540097561
Hepatocyte growth factor receptor	0.609601937
cAMP-dependent protein kinase catalytic subunit alpha	0.45919557
cAMP-dependent protein kinase catalytic subunit alpha	0.406587001
Cathepsin S	0.691263015
L-lactate dehydrogenase B chain	0.4375
Carbonic anhydrase 2	0.765755135
Histone deacetylase 7	0.928571429
Mitogen-activated protein kinase 14	0.655898876
Receptor-type tyrosine-protein phosphatase alpha	0.90942029
Ephrin type-A receptor 2	0.615909091
Prothrombin	0.423777313
Queuine tRNA-ribosyltransferase	0.614285714
Disintegrin and metalloproteinase domain-containing protein 17	0.446480938
Kallikrein-7	0.5
Renin	0.756628788
Deoxyuridine 5'-triphosphate nucleotidohydrolase, mitochondrial	0.700808625
Mitogen-activated protein kinase 1	0.645992714
STE20-like serine/threonine-protein kinase	0.495541023
Apoptosis regulator Bcl-2	0.70864726
Cationic trypsin	0.650611838
Tyrosine-protein kinase ABL1	0.576561061
Metabotropic glutamate receptor 4	0.071428571
Myosin light chain kinase, smooth muscle	0.475420875
Glucocorticoid receptor	0.722681596
Coagulation factor VII	0.702892069
Fibroblast growth factor receptor 2	0.601851852
Serum albumin	0.444444444
Neutrophil elastase	0.575191571
Carbonic anhydrase 2	0.7702608
Prothrombin	0.456926608
Glucocorticoid receptor	0.420095408
Dihydroorotate dehydrogenase (quinone), mitochondrial	0.684848485
Papain	0.49394021
Squalene monooxygenase	1
Kallikrein-7	0
Tyrosine-protein kinase JAK2	0.554262199
WD repeat-containing protein 5	0.47826087
Serine/threonine-protein kinase pim-1	0.670013167
Fibroblast growth factor receptor 1	0.675347222
Tyrosine-protein kinase Fgr	0.623144399
Hepatocyte growth factor receptor	0.586482293
Cyclin-dependent kinase 2	0.360464642
Cathepsin K	0.538343424

PROTEIN FAMILY	AUC
Cathepsin B	0.529920255
3-hydroxy-3-methylglutaryl-coenzyme A reductase	0.676470588
A disintegrin and metalloproteinase with thrombospondin motifs 1	1
Neutrophil collagenase	0.670489085
Mitogen-activated protein kinase 14	0.733943682
Serine/threonine-protein kinase PLK1	0.351795264
Tyrosine-protein phosphatase non-receptor type 1	0.672335601
cAMP-specific 3',5'-cyclic phosphodiesterase 4D	0.704092204
Aminopeptidase N	0.539181287
Matrilysin	0.31584596
Baculoviral IAP repeat-containing protein 2	0.1
Glutathione S-transferase P	0.85
Guanine deaminase	0.5
Calcium/calmodulin-dependent protein kinase type II subunit beta	0.370925684
Retinoic acid receptor gamma	0.527006881
Histone deacetylase-like amidohydrolase	0.272727273
Fibroblast growth factor receptor 1	0.593055556
Orotidine 5'-phosphate decarboxylase	0.457142857
Interstitial collagenase	0.434782446
Neuromedin-B receptor	0.930232558
Glycogen phosphorylase, liver form	0
Ribonuclease pancreatic	0.338509317
Thymidylate synthase	0.611111111
Cytochrome P450 130	1
Beta-lactamase	0.209876543
Fibroblast growth factor receptor 1	0.592857143
Collagenase 3	0.402434783
Estrogen receptor	0.590108735
Queuine tRNA-ribosyltransferase	0.6
Cyclin-dependent kinase 2	0.330614341
Nitric oxide synthase, inducible	0.454757463
Prothrombin	0.444620522
Thymidylate synthase	0.524046434
Renin	0.809067235
cAMP-dependent protein kinase catalytic subunit alpha	0.423928884
Estradiol 17-beta-dehydrogenase 1	0.333333333
RAC-alpha serine/threonine-protein kinase	0.63200431
Glucosylceramidase	0.770676692
Pteridine reductase 1	0.746753247
Protein kinase C beta type	0.450909091
Beta-lactamase TEM	0.476470588
Cell division protein ZipA	0
Urokinase-type plasminogen activator	0.536389708
Serine/threonine-protein kinase PLK1	0.449579832
Transketolase	0.464285714
Amine oxidase [flavin-containing] B	0.624768856
Peroxisome proliferator-activated receptor alpha	0.246806039
Acetylcholinesterase	1
Carboxypeptidase B	0.325609756
Serum albumin	0.451388889
Peripheral plasma membrane protein CASK	0.5
Collagenase 3	0.435453416
Queuine tRNA-ribosyltransferase	0.585714286

PROTEIN FAMILY	AUC
P2Y purinoceptor 12	0.565973742
Peroxisome proliferator-activated receptor gamma	0.866753472
Peptidyl-prolyl cis-trans isomerase NIMA-interacting 1	0.78018278
Cationic trypsin	0.672941767
Phosphatidylinositol 4-kinase alpha	0.4904
Dipeptidyl peptidase 9	0.513815789
Androgen receptor	0.644992179
Renin	0.76077178
Thymidylate synthase	0.626984127
Interleukin-1 receptor-associated kinase 4	0.645628415
Peptidyl-prolyl cis-trans isomerase FKBP1A	0.17162743
Papain	0.453002963
Fibroblast growth factor receptor 2	0.634259259
Leukotriene A-4 hydrolase	0.833333333
Serum albumin	0.368055556
Coagulation factor XI	0.656862745
Tyrosine-protein kinase Lck	0.668233177
Aldose reductase	0.527777778
Receptor-type tyrosine-protein phosphatase beta	0.525
Leukotriene A-4 hydrolase	1
Phosphatidylinositol 4-phosphate 5-kinase type-1 alpha	0.527672956
Nitric oxide synthase, endothelial	0.395566922
Cationic trypsin	0.635026927
Glucocorticoid receptor	0.676658053
Glycogen synthase kinase-3 beta	0.574920438
Myosin light chain kinase, smooth muscle	0.392592593
Estrogen receptor	0.680056306
Seprase	0.268023256
Solute carrier family 22 member 6	0.666666667
Receptor tyrosine-protein kinase erbB-4	0.4845
Mitogen-activated protein kinase 14	0.689589402
Macrophage metalloelastase	0.338891807
Stromelysin-1	0.484452243
Peptidyl-prolyl cis-trans isomerase NIMA-interacting 1	0.752765753
Prothrombin	0.562148536
Thymidylate synthase	0.645743146
Prothrombin	0.466455789
Tyrosine-protein kinase JAK1	0.598958333
Beta-lactamase	0.230769231
Serine/threonine-protein kinase Chk2	0.639367816
Serine/threonine-protein kinase PLK1	0.420550038
Cyclin-dependent kinase 2	0.377796832
Nuclear receptor subfamily 1 group I member 2	0
Glutathione S-transferase Mu 2	0.6
Glutathione S-transferase Mu 2	0.5
Tyrosine-protein kinase BTK	0.487637363
Coagulation factor X	0.513863785
Ectonucleoside triphosphate diphosphohydrolase 1	1
Ribosyldihydronicotinamide dehydrogenase [quinone]	0.166666667
Tryptase alpha/beta-1	0.198529412
Peptidyl-prolyl cis-trans isomerase FKBP1A	0.79241773
5'-methylthioadenosine/S-adenosylhomocysteine nucleosidase	0.11627907
Coagulation factor XI	0.39869281

PROTEIN FAMILY	AUC
Cathepsin K	0.573422831
Histone deacetylase 2	0.815789474
Gamma-aminobutyric acid receptor subunit theta	0.333333333
6,7-dimethyl-8-ribityllumazine synthase	0.469387755
Orotidine 5'-phosphate decarboxylase	0.414285714
Dihydrofolate reductase	0.418128655
Cationic trypsin	0.649428571
Glycogen synthase kinase-3 beta	0.563096711
Queuine tRNA-ribosyltransferase	0.6
Aminopeptidase N	0.75
Neutrophil elastase	0.566924375
Coagulation factor X	0.526186895
Thymidylate synthase	0.701575138
Corticosteroid 11-beta-dehydrogenase isozyme 1	0.594405594
STE20-like serine/threonine-protein kinase	0.528537455
Protein kinase C beta type	0.6
Serine/threonine-protein kinase tousled-like 2	0.479284369
Fatty acid-binding protein, adipocyte	0.858552632
Dual specificity tyrosine-phosphorylation-regulated kinase 2	0.394241417
Glycogen phosphorylase, liver form	0
Beta-lactamase	0.5625
Phosphatidylinositol 4,5-bisphosphate 3-kinase catalytic subunit gamma isoform	0.691091954
Corticosteroid 11-beta-dehydrogenase isozyme 1	0.640859141
Aldose reductase	0.583333333
RAC-beta serine/threonine-protein kinase	0.445441663
Somatostatin receptor type 2	0.512104005
Peptidyl-prolyl cis-trans isomerase FKBP1A	0.737058182
Abelson tyrosine-protein kinase 2	0.497785161
Peptidyl-prolyl cis-trans isomerase NIMA-interacting 1	0.445646946
Melanocortin receptor 4	0.583398825
Proto-oncogene tyrosine-protein kinase Src	0.47032967
Fibroblast growth factor receptor 1	0.622222222
Estrogen receptor beta	0.745723057
Neprilysin	0.4739369
Ephrin type-B receptor 1	0.664027149
Endothelin B receptor	0.563612314
4-aminobutyrate aminotransferase, mitochondrial	0.334782609
Aurora kinase A	0.534666667
Peptidyl-prolyl cis-trans isomerase NIMA-interacting 1	0.692400192
Megakaryocyte-associated tyrosine-protein kinase	0.307017544
Amyloid beta A4 protein	0.52173913
Poly [ADP-ribose] polymerase 3	1
Coagulation factor X	0.541609101
Transketolase	0.466269841
Serum albumin	0.645833333
Coagulation factor X	0.568436259
Glucocorticoid receptor	0.546712604
Queuine tRNA-ribosyltransferase	0.614285714
Peroxisome proliferator-activated receptor alpha	0.235481998
Beta-lactamase OXA-10	1
Prothrombin	0.431477948
Cyclin-dependent kinase 2	0.364547607
Serine/threonine-protein kinase pim-1	0.692922976

PROTEIN FAMILY	AUC
Carbonic anhydrase 1	0.705810639
Mitogen-activated protein kinase kinase kinase 19	0.486842105
Cathepsin L1	0.609202006
Chymotrypsinogen A	0.691609589
B2 bradykinin receptor	0.465036645
Glutamate receptor 3	0.242424242
Serine/threonine-protein kinase pim-1	0.674588545
Prostate-specific antigen	0.403571429
Calpain small subunit 1	0.45625
Mast/stem cell growth factor receptor Kit	0.676517526
Mitogen-activated protein kinase 10	0.586471861
Protein kinase C gamma type	0.682010582
Serine/threonine-protein kinase B-raf	0.624362245
Hepatocyte growth factor receptor	0.656492248
Estrogen receptor	0.707009576
TGF-beta receptor type-2	0.717418546
Gamma-aminobutyric acid receptor subunit alpha-4	0.48828125
Peptidyl-prolyl cis-trans isomerase FKBP4	0.476190476
Ephrin type-A receptor 2	0.331439394
Amine oxidase [flavin-containing] B	0.622919708
Coagulation factor X	0.523141702
Cyclin-dependent kinase-like 1	0.488888889
Heat shock protein HSP 90-alpha	0.871462264
cAMP-dependent protein kinase catalytic subunit alpha	0.40556689
Dihydroorotate dehydrogenase (quinone), mitochondrial	0.698333333
Coagulation factor VII	0.640826873
Macrophage migration inhibitory factor	0.875
Nitric oxide synthase, inducible	0.635899698
Peptidyl-prolyl cis-trans isomerase A	0.898648649
Focal adhesion kinase 1	0.476388889
Beta-secretase 1	0.818248821
Retinoic acid receptor RXR-alpha	0.592322643
Ribosyldihydronicotinamide dehydrogenase [quinone]	0.166666667
SNF-related serine/threonine-protein kinase	0.348275862
Serine/threonine-protein kinase BRSK2	0.406666667
Myosin light chain kinase, smooth muscle	0.467676768
3-dehydroquinate dehydratase	0.944444444
Aurora kinase A	0.517501648
Metabotropic glutamate receptor 1	0.634615385
Carbonic anhydrase 2	0.762661522
Cationic trypsin	0.657220497
Serine/threonine-protein kinase B-raf	0.662414966
Caspase-8	0.78021978
Serine/threonine-protein kinase tousled-like 1	0.446070878
Calcium/calmodulin-dependent protein kinase type 1G	0.505867014
Peptidyl-prolyl cis-trans isomerase A	0.896396396
Fatty acid-binding protein, adipocyte	0.773026316
Tyrosine-protein phosphatase non-receptor type 2	0.505877034
Peroxisome proliferator-activated receptor gamma	0.808159722
Coagulation factor VII	0.730023852
Heat shock protein HSP 90-alpha	0.811320755
Serum albumin	0.409722222
Collagenase 3	0.43868323

PROTEIN FAMILY	AUC
Adenosine deaminase	0.804526749
Tryptophan 2,3-dioxygenase	1
Myosin light chain kinase 3	0.575801749
Beta-1,4-galactosyltransferase 1	1
Glutamate receptor ionotropic, kainate 5	0.391025641
Serine/threonine-protein kinase MRCK alpha	0.69057377
Alcohol dehydrogenase class 4 mu/sigma chain	0.35
Aurora kinase A	0.549183007
Collagenase 3	0.432173913
Transthyretin	0.857142857
Cyclin-dependent kinase-like 2	0.600241546
Nitric oxide synthase, endothelial	0.394430236
Serine/threonine-protein kinase pim-1	0.677353522
C-X-C chemokine receptor type 1	0.968085106
Peptidyl-prolyl cis-trans isomerase NIMA-interacting 1	0.697931698
Dihydroorotate dehydrogenase (quinone), mitochondrial	0.693409091
Peroxisome proliferator-activated receptor gamma	0.7484375
Myosin-IIIa	0.324225865
Peroxisome proliferator-activated receptor gamma	0.746354167
Aurora kinase C	0.656944444
Renin	0.726207386
Papain	0.471316994
Prothrombin	0.450589196
Prothrombin	0.589795525
3-oxoacyl-[acyl-carrier-protein] synthase 3	0.928571429
Mitogen-activated protein kinase 10	0.59237013
Leukocyte tyrosine kinase receptor	0.396170213
3-phosphoshikimate 1-carboxyvinyltransferase	0.677777778
Queuine tRNA-ribosyltransferase	0.685714286
cGMP-specific 3',5'-cyclic phosphodiesterase	0.930213465
RAC-alpha serine/threonine-protein kinase	0.605257556
Eukaryotic translation initiation factor 2-alpha kinase 4	0.494331066
3-phosphoinositide-dependent protein kinase 1	0.352488688
Beta-secretase 1	0.791126179
Bacterial leucyl aminopeptidase	0.272727273
Fibroblast growth factor receptor 3	0.593841642
Aurora kinase A	0.615577236
Mitogen-activated protein kinase 10	0.531655844
Peptidyl-prolyl cis-trans isomerase A	0.944256757
Macrophage-stimulating protein receptor	0.613207547
Phosphatidylinositol 5-phosphate 4-kinase type-2 gamma	0.53427896
Androgen receptor	0.610567993
Homeodomain-interacting protein kinase 2	0.517784553
Tyrosine-protein kinase HCK	0.44672619
Tissue-type plasminogen activator	0.455507041
Vascular endothelial growth factor receptor 2	0.551729783
cAMP-dependent protein kinase catalytic subunit alpha	0.452491985
WD repeat-containing protein 5	0.181818182
Fibroblast growth factor receptor 1	0.691836735
Serine hydroxymethyltransferase, cytosolic	0.5
Melanocortin receptor 4	0.347693005
Bromodomain-containing protein 4	0.5
Dipeptidyl peptidase 4	0.70845959

PROTEIN FAMILY	AUC
Mitogen-activated protein kinase kinase kinase 3	0.463414634
Estrogen receptor	0.639430822
Thymidylate synthase	0.532338308
Serine/threonine-protein kinase Nek7	0.561633282
Aurora kinase A	0.580585366
Folylpolyglutamate synthase, mitochondrial	0.791666667
3-phosphoshikimate 1-carboxyvinyltransferase	0.633333333
Proto-oncogene tyrosine-protein kinase ROS	0.665413534
Orotidine 5'-phosphate decarboxylase	0.6
cGMP-dependent protein kinase 1	0.341397849
SRSF protein kinase 3	0.479974969
Glutathione S-transferase A1	0.533333333
Beta-lactamase OXA-10	1
Urokinase-type plasminogen activator	0.530612914
Thyrotropin-releasing hormone receptor	0.138888889
Glutathione S-transferase P	0.875
5'-methylthioadenosine/S-adenosylhomocysteine nucleosidase	0.5
Serine/threonine-protein kinase PAK 6	0.525862069
Proto-oncogene tyrosine-protein kinase receptor Ret	0.488325839
Sodium- and chloride-dependent glycine transporter 1	1
Tryptophan 2,3-dioxygenase	0.75
Macrophage migration inhibitory factor	0.5
cAMP-dependent protein kinase catalytic subunit alpha	0.400320606
Pseudolysin	0.567835366
Aurora kinase A	0.548823529
Tyrosine-protein kinase ABL1	0.525
Macrophage metalloelastase	0.394957983
Serine/threonine-protein kinase pim-1	0.6899605
Dual specificity mitogen-activated protein kinase kinase 4	0.383647799
Cationic trypsin	0.640526391
Receptor tyrosine-protein kinase erbB-2	0.398642534
Endothelin B receptor	0.670297749
Cationic trypsin	0.647339074
Serine/threonine-protein kinase PLK1	0.372039725
Peroxisome proliferator-activated receptor delta	0.693121693
Beta-secretase 1	0.81301592
Methionine aminopeptidase 2	0.575711382
cAMP-dependent protein kinase catalytic subunit alpha	0.505829204
Mitogen-activated protein kinase 14	0.595505618
Vascular endothelial growth factor receptor 2	0.523109244
N-acetylated-alpha-linked acidic dipeptidase 2	0.736842105
Urokinase-type plasminogen activator	0.533923913
Protein kinase C theta type	0.363599677
3-phosphoshikimate 1-carboxyvinyltransferase	0.611111111
Cationic trypsin	0.678075397
Rho-associated protein kinase 1	0.382166667
Endothelin-converting enzyme 1	0.488311688
Lethal(3)malignant brain tumor-like protein 1	0.333333333
RAC-gamma serine/threonine-protein kinase	0.665131579
Phosphatidylinositol 4,5-bisphosphate 3-kinase catalytic subunit gamma isoform	0.546695402
Carbonic anhydrase 2	0.763641738
Cationic trypsin	0.632709409
Dehydrosqualene synthase	0.791666667

PROTEIN FAMILY	AUC
Serine/threonine-protein kinase 32B	0.662068966
Mitogen-activated protein kinase 10	0.568290043
Nitric oxide synthase, inducible	0.643239667
Tyrosine-protein kinase Fes/Fps	nan
Tyrosine-protein kinase Srms	0.449519231
TGF-beta receptor type-1	0.636174636
Arginase-1	0.271428571
Heat shock protein HSP 90-alpha	0.859669811
Non-receptor tyrosine-protein kinase TYK2	0.536755952
Breast cancer type 1 susceptibility protein	0.333333333
Macrophage-stimulating protein receptor	0.668818272
Glutamate receptor ionotropic, kainate 1	0.272596154
Cholinesterase	0.812906504
Mitogen-activated protein kinase 9	0.546057767
Dipeptidyl peptidase 4	0.699319458
Angiotensin-converting enzyme 2	0.695402299
Proto-oncogene tyrosine-protein kinase Src	0.557460317
Serine/threonine-protein kinase pim-2	0.806323529
Myosin-IIIb	0.671220401
Caspase-5	0.775
Trifunctional purine biosynthetic protein adenosine-3	0.703947368
Caspase-1	0.662316623
Serine/threonine-protein kinase 33	0.607336957
Abelson tyrosine-protein kinase 2	0.385105205
Estrogen receptor	0.721787185
Amine oxidase [flavin-containing] B	0.594695864
Orotidine 5'-phosphate decarboxylase	0.428571429
Ribosyldihydronicotinamide dehydrogenase [quinone]	0.161111111
Cathepsin L1	0.558987688
Insulin-like growth factor 1 receptor	0.61302521
Caspase-9	0.333333333
Tissue-type plasminogen activator	0.445634756
Thermolysin	0.569189189
Cyclin-dependent kinase 9	0.330357143
Plasmepsin-2	0.804776039
Prothrombin	0.446200421
Coagulation factor VII	0.809630292
Carbonic anhydrase 12	0.568458345
LIM domain kinase 2	0.625
Mast/stem cell growth factor receptor Kit	0.56187007
Coagulation factor X	0.54678418
Serum albumin	0.368055556
Hydroxyacylglutathione hydrolase, mitochondrial	1
Neprilysin	0.437585734
Cyclin-dependent kinase 2	0.356810387
Thymidylate synthase	0.580016584
Thyroid hormone receptor beta	0.138736264
Tyrosine-protein kinase TXK	0.466521739
Tyrosine-protein phosphatase non-receptor type 1	0.744959245
S-adenosylmethionine decarboxylase proenzyme	0.618181818
Aminopeptidase N	0.666666667
S-methyl-5'-thioadenosine phosphorylase	0.128205128
Transthyretin	0.785714286

PROTEIN FAMILY	AUC
Cathepsin L1	0.603155495
Coagulation factor VII	0.751639833
Tyrosine-protein kinase TXK	0.530612245
Tyrosine-protein kinase FRK	0.480192077
Queuine tRNA-ribosyltransferase	0.671428571
Coagulation factor X	0.543468813
S-adenosylmethionine decarboxylase proenzyme	0.654545455
Sodium- and chloride-dependent glycine transporter 1	0.848484848
Aurora kinase A	0.548154252
Urokinase-type plasminogen activator	0.580638491
Coagulation factor X	0.551761642
Beta-lactamase	0.406666667
Phenylethanolamine N-methyltransferase	0.757169118
Mu-type opioid receptor	0.561311255
E3 ubiquitin-protein ligase XIAP	0.559615385
Uridine 5'-monophosphate synthase	0.15
Prothrombin	0.580687831
Dual specificity mitogen-activated protein kinase kinase 1	0.38851096
Casein kinase I isoform alpha-like	0.555555556
Mitogen-activated protein kinase kinase kinase 10	0.630508475
Cyclin-dependent kinase 9	0.376040428
3-dehydroquinate dehydratase	0.944444444
Mitogen-activated protein kinase kinase kinase 19	0.585106383
Serine/threonine-protein kinase Chk1	0.622303922
Glutamate carboxypeptidase 2	0.658008658
Glycogen synthase kinase-3 beta	0.571074965
Glutathione S-transferase Mu 1	0.2
Caspase-8	0.230769231
Kinesin-like protein KIF11	0.769230769
Activin receptor type-1B	0.59077381
Transient receptor potential cation channel subfamily M member 6	0.147540984
Cathepsin B	0.658313908
Cholinesterase	0.829941565
Coagulation factor XI	0.388888889
Thymidylate synthase	0.637085137
Leukotriene A-4 hydrolase	1
Cathepsin L1	0.632375741
Estradiol 17-beta-dehydrogenase 1	0.666666667
Collagenase 3	0.402409938
Dual serine/threonine and tyrosine protein kinase	0.433673469
Peroxisome proliferator-activated receptor gamma	0.787586806
Plasmepsin-2	0.69562871
Transient receptor potential cation channel subfamily M member 6	0.508196721
Ribosyldihydronicotinamide dehydrogenase [quinone]	0.122222222
Squalene monooxygenase	0.666666667
N-acetylated-alpha-linked acidic dipeptidase 2	0.947368421
Serine/threonine-protein kinase B-raf	0.65795068
Bcl-2-related protein A1	1
Macrophage colony-stimulating factor 1 receptor	0.767788196
Ephrin type-A receptor 3	0.484876543
Peroxisome proliferator-activated receptor gamma	0.744010417
Lethal factor	0.411713287
Mitogen-activated protein kinase kinase kinase 19	0.500548246

PROTEIN FAMILY	AUC
SRSF protein kinase 1	0.5639413
Thyroid hormone receptor beta	0.203846154
Chymotrypsinogen A	0.738020833
5-hydroxytryptamine receptor 1D	0.500527793
Androgen receptor	0.637440121
Peroxisome proliferator-activated receptor gamma	0.705208333
Cyclin-dependent kinase 2	0.50296015
Orotidine 5'-phosphate decarboxylase	0.5
cAMP-dependent protein kinase catalytic subunit alpha	0.433401341
Methionine aminopeptidase 1	0.697463768
Serine/threonine-protein kinase SIK3	0.614379085
Endothelin B receptor	0.386831276
Cathepsin E	0.041666667
Glutamate receptor 3	0.121212121
Neprilysin	0.402263374
Glucocorticoid receptor	0.620614413
Beta-secretase 1	0.817732901
Serine/threonine-protein kinase MRCK alpha	0.478825137
Histone deacetylase-like amidohydrolase	0.090909091
Glutamate receptor ionotropic, kainate 5	0.293040293
Chymotrypsinogen A	0.670890411
Carboxypeptidase A1	0.181899642
Carbonic anhydrase 2	0.757760039
3-dehydroquinate dehydratase	0.944444444
Coagulation factor VII	0.696829656
Rho-associated protein kinase 1	0.363833333
Phenylethanolamine N-methyltransferase	0.751286765
Alpha-2B adrenergic receptor	0.530868694
Serine/threonine-protein kinase Nek4	0.673728814
Dual specificity mitogen-activated protein kinase kinase 5	0.52952953
Beta-lactamase OXA-10	1
Insulin-like growth factor 1 receptor	0.612184874
Myosin light chain kinase, smooth muscle	0.517508418
Amine oxidase [flavin-containing] B	0.613673966
Fibroblast growth factor receptor 2	0.507092199
UDP-3-O-[3-hydroxymyristoyl] N-acetylglucosamine deacetylase	0.4375
Prothrombin	0.434728478
Homeodomain-interacting protein kinase 1	0.56952381
Cyclin-dependent kinase 9	0.383769322
Heat shock protein HSP 90-alpha	0.839622642
Nitric oxide synthase, endothelial	0.42114237
Aldose reductase	0.361111111
Mitogen-activated protein kinase kinase kinase 15	0.357758621
Beta-lactamase	0.193548387
Tyrosine-protein kinase ZAP-70	0.392361111
WD repeat-containing protein 5	0.413043478
Calcium/calmodulin-dependent protein kinase kinase 1	0.614150943
Solute carrier family 15 member 1	0.875458716
Receptor tyrosine-protein kinase erbB-3	0.470748299
Ephrin type-A receptor 7	0.425314465
Thymidylate kinase	0.767857143
Ectonucleotide pyrophosphatase/phosphodiesterase family member 2	0.587931034
Epithelial discoidin domain-containing receptor 1	0.68261563

PROTEIN FAMILY	AUC
Trifunctional purine biosynthetic protein adenosine-3	0.620065789
Beta-lactamase OXA-10	1
cAMP-specific 3',5'-cyclic phosphodiesterase 4C	0.705357143
Aurora kinase A	0.606437908
Peroxisome proliferator-activated receptor gamma	0.750260417
Insulin-like growth factor 1 receptor	0.640756303
Death-associated protein kinase 2	0.556197479
Tyrosine-protein kinase Lck	0.680381962
Thymidylate synthase	0.532338308
Disintegrin and metalloproteinase domain-containing protein 17	0.619868035
Aurora kinase A	0.518829268
Cyclin-dependent kinase 2	0.36062796
Ribonuclease pancreatic	0.40625
Alpha-galactosidase A	1
Serine/threonine-protein kinase pim-1	0.629163924
Orotidine 5'-phosphate decarboxylase	0.435714286
Ectonucleotide pyrophosphatase/phosphodiesterase family member 1	0.383333333
Peptidyl-prolyl cis-trans isomerase A	0.939189189
Receptor tyrosine-protein kinase erbB-4	0.464285714
Queuine tRNA-ribosyltransferase	0.714285714
cAMP-specific 3',5'-cyclic phosphodiesterase 4D	0.806397306
Cyclin-dependent kinase 11A	0.601880878
Dihydroorotate dehydrogenase (quinone), mitochondrial	0.654090909
Growth factor receptor-bound protein 2	0.549242424
Heat shock protein HSP 90-alpha	0.891509434
Cationic trypsin	0.651501138
Granulocyte colony-stimulating factor receptor	0
cAMP-specific 3',5'-cyclic phosphodiesterase 4B	0.844390507
Estradiol 17-beta-dehydrogenase 1	0.666666667
Tryptase alpha/beta-1	0.214572193
Protein kinase C alpha type	0.511290091
Casein kinase I isoform gamma-3	0.410631741
Tyrosine-protein phosphatase non-receptor type 1	0.656891585
Cathepsin B	0.6500259
Acidic mammalian chitinase	1
Excitatory amino acid transporter 1	0.961538462
Prothrombin	0.419613187
Retinoic acid receptor RXR-alpha	0.614431487
Orotidine 5'-phosphate decarboxylase	0.457142857
Bifunctional dihydrofolate reductase-thymidylate synthase	0.522006221
Tyrosine-protein phosphatase non-receptor type 1	0.675813569
Coagulation factor XI	0.620915033
Peptidyl-prolyl cis-trans isomerase A	0.927927928
Amine oxidase [flavin-containing] B	0.605255474
MAP/microtubule affinity-regulating kinase 4	0.670433145
Serine/threonine-protein kinase Chk2	0.568518519
CREB-binding protein	0.333333333
Cationic trypsin	0.655497199
Thymidylate synthase	0.54933665
Coagulation factor VII	0.806946929
Peptidyl-prolyl cis-trans isomerase A	0.935810811
Glucagon receptor	0.78252551
Calcium/calmodulin-dependent protein kinase type II subunit delta	0.481339713

PROTEIN FAMILY	AUC
Angiotensin-converting enzyme	0.514605978
Corticosteroid 11-beta-dehydrogenase isozyme 1	0.56018981
Gamma-aminobutyric acid receptor subunit beta-2	0.234791667
Metabotropic glutamate receptor 3	0.647058824
Transient receptor potential cation channel subfamily M member 6	0.774193548
Beta-secretase 1	0.818027712
Thymidylate synthase	0.613598673
Vascular endothelial growth factor receptor 3	0.618112014
Tyrosine-protein kinase ABL1	0.554206596
Sialidase-2	0.166666667
Transthyretin	0.75
Acidic mammalian chitinase	1
Tyrosine-protein kinase ITK/TSK	0.664753795
Alpha-1A adrenergic receptor	0.799807779
Carboxypeptidase A1	0.683823529
Serine/threonine-protein kinase RIO2	nan
Cyclin-dependent kinase 2	0.377980565
Angiotensin-converting enzyme	0.511209239
Adenosine kinase	0.603298611
Queuine tRNA-ribosyltransferase	0.585714286
Cationic trypsin	0.656015038
Nitric oxide synthase, endothelial	0.419011083
Dipeptidyl peptidase 4	0.71752521
Vascular endothelial growth factor receptor 2	0.651396922
3-phosphoinositide-dependent protein kinase 1	0.479638009
Phosphatidylinositol 4-phosphate 5-kinase type-1 alpha	0.613836478
Abelson tyrosine-protein kinase 2	0.566998893
Amine oxidase [flavin-containing] B	0.614890511
Peptidyl-prolyl cis-trans isomerase A	0.905405405
Mitogen-activated protein kinase 7	0.414035088
Leucyl-cystinyl aminopeptidase	0.856092437
Acetylcholinesterase	0.54
Cathepsin S	0.563991674
cGMP-dependent protein kinase 1	0.670698925
Caspase-1	0.775897759
Mu-type opioid receptor	0.612951843
Cyclin-dependent kinase 2	0.374101748
Lethal(3)malignant brain tumor-like protein 1	0.375
Chymase	0.667128988
Serum albumin	0.381944444
Beta-secretase 1	0.780070755
Tyrosine-protein kinase ABL1	0.612676056
Phosphatidylinositol 4-kinase alpha	0.64
5-hydroxytryptamine receptor 1A	0.636895621
Sialidase-2	0.666666667
Kinesin-like protein KIF11	0.533653846
Misshapen-like kinase 1	0.574074074
Aurora kinase C	0.611111111
Hepatocyte growth factor receptor	0.671203312
Proto-oncogene tyrosine-protein kinase Src	0.645030525
ALK tyrosine kinase receptor	0.617542614
Receptor tyrosine-protein kinase erbB-3	nan
Ribosyldihydronicotinamide dehydrogenase [quinone]	0.1

PROTEIN FAMILY	AUC
Aldose reductase	0.527777778
3-dehydroquinate dehydratase	0.333333333
Stromelysin-1	0.449654883
Ribonuclease pancreatic	0.48136646
Purine nucleoside phosphorylase	0.505952381
Rhodopsin kinase	0.588435374
Renin	0.745975379
Dipeptidyl peptidase 4	0.731260245
Vitamin K-dependent protein C	0.6625
Peroxisome proliferator-activated receptor gamma	0.708246528
Receptor tyrosine-protein kinase erbB-3	0.454861111
Beta-2 adrenergic receptor	0.591392519
Beta-lactamase	0.406666667
Peptidyl-prolyl cis-trans isomerase A	0.916666667
Prostaglandin E2 receptor EP4 subtype	0.457660992
Heat shock protein HSP 90-alpha	0.886792453
N(G),N(G)-dimethylarginine dimethylaminohydrolase 1	0.555555556
DNA polymerase alpha catalytic subunit	0.323529412
Cationic trypsin	0.640986731
Leukotriene A-4 hydrolase	1
Alpha-1A adrenergic receptor	0.185153777
Cyclin-dependent kinase 2	0.396231423
Glutamate carboxypeptidase 2	0.501082251
Alpha-galactosidase A	1
Histone deacetylase-like amidohydrolase	0.545454545
Thymidylate synthase	0.60655058
Coagulation factor VII	0.806102167
Calcium/calmodulin-dependent protein kinase type II subunit gamma	0.474789916
Vascular endothelial growth factor receptor 2	0.600484262
Gamma-aminobutyric acid receptor subunit alpha-1	0.666452499
Vascular endothelial growth factor receptor 2	0.5562043
Dual specificity mitogen-activated protein kinase kinase 6	0.535767511
Glutamate carboxypeptidase 2	0.727272727
Glucosylceramidase	0.78806391
Hepatocyte growth factor receptor	0.603595123
Protein delta homolog 1	0.75
Myosin light chain kinase, smooth muscle	0.442424242
Tyrosine-protein kinase JAK1	0.517515052
Receptor-type tyrosine-protein phosphatase beta	0.45
Neutrophil elastase	0.589365535
Serine/threonine-protein kinase Nek3	0.279661017
Peroxisome proliferator-activated receptor alpha	0.277003484
Peroxisome proliferator-activated receptor alpha	0.253484321
NT-3 growth factor receptor	0.422
Fibroblast growth factor receptor 1	0.644217687
Dihydrofolate reductase	0.372807018
Ectonucleotide pyrophosphatase/phosphodiesterase family member 2	0.339830508
Caspase-3	0.821969697
Stromelysin-1	0.42874965
Proto-oncogene tyrosine-protein kinase Src	0.623150183
Acetylcholinesterase	0.506666667
Beta-secretase 1	0.799086085
Tyrosine-protein kinase JAK2	0.569194591

PROTEIN FAMILY	AUC
Ectonucleotide pyrophosphatase/phosphodiesterase family member 2	0.330508475
3-dehydroquinate dehydratase	0.833333333
Receptor tyrosine-protein kinase erbB-3	0.459027778
Carbonic anhydrase 1	0.70804478
Receptor-type tyrosine-protein phosphatase beta	0.575
Megakaryocyte-associated tyrosine-protein kinase	0.304187192
3-dehydroquinate dehydratase	0.886666667
Interleukin-1 receptor-associated kinase 1	0.403240741
Thymidylate synthase	0.612554113
Sphingosine kinase 2	1
Glutathione S-transferase Mu 2	0.2
Urokinase-type plasminogen activator	0.531966472
Casein kinase II subunit alpha	0.443766938
Dual specificity tyrosine-phosphorylation-regulated kinase 1A	0.521604938
Dihydroorotate dehydrogenase (quinone), mitochondrial	0.705530303
Muscarinic acetylcholine receptor M2	0.607145733
RAC-beta serine/threonine-protein kinase	0.408124705
Peroxisome proliferator-activated receptor gamma	0.7015625
Carboxypeptidase B2	0.931034483
Endothelin B receptor	0.55204551
Dual specificity tyrosine-phosphorylation-regulated kinase 2	0.419712071
Macrophage metalloelastase	0.415966387
Mitogen-activated protein kinase 9	0.559920635
Mitogen-activated protein kinase kinase kinase 5	0.555147059
ALK tyrosine kinase receptor	0.6171875
Thymidylate synthase	0.673160173
Cathepsin K	0.551724138
Inosine-5'-monophosphate dehydrogenase 1	0.452777778
Cholinesterase	0.835518293
RAC-alpha serine/threonine-protein kinase	0.591954023
Thymidylate kinase	0.704589931
Dehydrosqualene synthase	0.777777778
Purine nucleoside phosphorylase	0.616664403
Ribosomal protein S6 kinase alpha-1	0.541508539
Tyrosine-protein kinase ABL1	0.561564301
Dipeptidyl peptidase 1	0.5
Carbonic anhydrase 1	0.703633636
Fatty-acid amide hydrolase 1	0.589777259
Renin	0.759469697
Carbonic anhydrase 2	0.767781467
Casein kinase II subunit alpha	0.623131393
Glutathione S-transferase A1	0.7
cAMP-specific 3',5'-cyclic phosphodiesterase 4B	0.790183387
Mitogen-activated protein kinase 11	0.691145833
Mitogen-activated protein kinase 14	0.707622416
Nitric oxide synthase, endothelial	0.374538221
Transthyretin	0.785714286
Peptidyl-prolyl cis-trans isomerase A	0.938063063
Lethal(3)malignant brain tumor-like protein 1	0
Uridine 5'-monophosphate synthase	0.3
Somatostatin receptor type 2	0.753497456
Integrin alpha-IIb	0.554457953
Tyrosine-protein kinase ABL1	0.561044231

PROTEIN FAMILY	AUC
Epidermal growth factor receptor	0.70657839
Myosin-IIIb	0.493624772
Fatty acid-binding protein, adipocyte	0.838815789
Cationic trypsin	0.663796769
Tyrosine-protein kinase receptor Tie-1	0.446666667
Acetylcholinesterase	1
Protein-tyrosine kinase 2-beta	0.503611971
Peptidyl-prolyl cis-trans isomerase NIMA-interacting 1	0.778499278
Lanosterol 14-alpha demethylase	0.1875
Proto-oncogene tyrosine-protein kinase Src	0.718681319
Thymidylate synthase	0.692918973
Homeodomain-interacting protein kinase 1	0.429563492
Calcium/calmodulin-dependent protein kinase type II subunit beta	0.49934811
E3 ubiquitin-protein ligase XIAP	0.621985447
Tubulin beta-2B chain	0
3-hydroxy-3-methylglutaryl-coenzyme A reductase	0.691176471
Fibroblast growth factor receptor 2	0.574919872
Dual specificity protein kinase TTK	0.507363254
Acetylcholinesterase	1
Adenosine receptor A2b	0.640050955
RAC-beta serine/threonine-protein kinase	0.40198394
Prostaglandin E2 receptor EP1 subtype	0.577399873
Heat shock protein HSP 90-alpha	0.827830189
Androgen receptor	0.632187849
Queuine tRNA-ribosyltransferase	0.728571429
Transient receptor potential cation channel subfamily M member 6	0.295081967
Serine/threonine-protein kinase MARK2	0.459126984
Thymidylate kinase	0.767857143
Urokinase-type plasminogen activator	0.548990121
Cathepsin B	0.620318352
Heat shock protein HSP 90-alpha	0.8125
Glutathione S-transferase P	0.9
Beta-lactamase	0.194444444
Mu-type opioid receptor	0.552642864
Peptidyl-prolyl cis-trans isomerase A	0.986486486
Phosphatidylinositol 4,5-bisphosphate 3-kinase catalytic subunit gamma isoform	0.573994253
Proto-oncogene tyrosine-protein kinase ROS	0.442982456
Growth factor receptor-bound protein 2	0.401515152
Ephrin type-B receptor 6	0.447783251
Peroxisome proliferator-activated receptor gamma	0.705295139
Alpha-1A adrenergic receptor	0.302860697
cAMP-dependent protein kinase catalytic subunit alpha	0.471436899
Cytidine deaminase	0.6
WD repeat-containing protein 5	0.804347826
Histamine N-methyltransferase	0.166666667
Peroxisome proliferator-activated receptor gamma	0.482566248
Heat shock protein HSP 90-alpha	0.880896226
Nischarin	0.428240741
Cyclin-dependent kinase 2	0.359096848
Dihydroorotate dehydrogenase (quinone), mitochondrial	0.6875
Epidermal growth factor receptor	0.697150424
Stromelysin-1	0.416618086
Cathepsin K	0.532482232

PROTEIN FAMILY	AUC
Aldo-keto reductase family 1 member C1	0.854166667
Serine/threonine-protein kinase ULK2	0.53125
Uridine 5'-monophosphate synthase	0.05
Lethal(3)malignant brain tumor-like protein 1	0
Thermolysin	0.617297297
Rho-associated protein kinase 2	0.673571112
Mitogen-activated protein kinase 14	0.696507837
Adenosine receptor A2a	0.35140512
Dual specificity tyrosine-phosphorylation-regulated kinase 2	0.434108527
Mitogen-activated protein kinase 14	0.666475933
Coagulation factor X	0.525622365
Tyrosine-protein kinase HCK	0.545639535
Bacterial leucyl aminopeptidase	0.181818182
Prothrombin	0.419358983
Cyclin-dependent kinase 19	0.691477273
Serine/threonine-protein kinase NLK	0.556573276
Thermolysin	0.545945946
Serine/threonine-protein kinase Chk1	0.648039216
SRSF protein kinase 3	0.442428035
Serine/threonine-protein kinase Chk1	0.63627451
cAMP-dependent protein kinase catalytic subunit alpha	0.490527543
Cell division protein ZipA	0
cAMP-dependent protein kinase catalytic subunit alpha	0.400174876
WD repeat-containing protein 5	0.260869565
3-hydroxy-3-methylglutaryl-coenzyme A reductase	0.698529412
Lanosterol 14-alpha demethylase	0
M-phase inducer phosphatase 1	0.25
Cationic trypsin	0.658240103
Dual specificity protein kinase TTK	0.513674614
Alpha-1A adrenergic receptor	0.805913614
Peroxisome proliferator-activated receptor gamma	0.673378661
Beta-lactamase	0.236686391
Amine oxidase [flavin-containing] B	0.646326034
Cathepsin B	0.611891386
Serine/threonine-protein kinase DCLK1	0.619311876
Tyrosine-protein kinase ABL1	0.568981687
Mitogen-activated protein kinase 14	0.596538137
Estrogen receptor	0.651702613
Serum albumin	0.5625
Prothrombin	0.427559279
Peroxisome proliferator-activated receptor gamma	0.722916667
Heat shock protein HSP 90-alpha	0.816037736
Focal adhesion kinase 1	0.619444444
Growth factor receptor-bound protein 2	0.598484848
Proto-oncogene tyrosine-protein kinase Src	0.476214896
WD repeat-containing protein 5	0.315217391
Cationic trypsin	0.672254376
Scytalone dehydratase	0.629353234
Coagulation factor XI	0.539215686
Purine nucleoside phosphorylase	0.577006655
Myosin-IIIa	0.360655738
Baculoviral IAP repeat-containing protein 3	nan
Glutamate carboxypeptidase 2	0.652597403

PROTEIN FAMILY	AUC
cAMP-specific 3',5'-cyclic phosphodiesterase 4B	0.785329018
Ephrin type-A receptor 7	0.372641509
Prothrombin	0.429915142
AMP deaminase 1	0.551020408
Carbonic anhydrase 2	0.769007815
Adenosylhomocysteinase	0.272151899
Ribosomal protein S6 kinase alpha-1	0.551353875
Estrogen receptor beta	0.68331247
Histamine N-methyltransferase	0.166666667
Fatty-acid amide hydrolase 1	0.535064682
Beta-lactamase	0.666666667
Serine/threonine-protein kinase Chk1	0.637990196
Transthyretin	0.928571429
Prothrombin	0.557106845
Serine/threonine-protein kinase mTOR	0.663245575
Estradiol 17-beta-dehydrogenase 1	0.666666667
Queuine tRNA-ribosyltransferase	0.635714286
cAMP-specific 3',5'-cyclic phosphodiesterase 4B	0.863538296
Cyclin-dependent kinase 2	0.345725135
Dipeptidyl peptidase 4	0.711961651
Carbonic anhydrase 2	0.772717052
Heat shock protein HSP 90-alpha	0.884433962
Heat shock protein HSP 90-alpha	0.863207547
Corticosteroid 11-beta-dehydrogenase isozyme 1	0.638111888
Tyrosine-protein kinase ITK/TSK	0.615230095
Ribonuclease pancreatic	0.419254658
Serine/threonine-protein kinase Chk2	0.636111111
Coagulation factor X	0.566976782
Serine/threonine-protein kinase PLK1	0.453017571
NADPH oxidase 4	0.517857143
Proto-oncogene tyrosine-protein kinase Src	0.584493284
N(G),N(G)-dimethylarginine dimethylaminohydrolase 1	0.666666667
Lanosterol 14-alpha demethylase	0.851851852
Dual specificity protein kinase CLK3	0.53219697
Seminal ribonuclease	0.666666667
Glycogen phosphorylase, muscle form	0.285714286
Peroxisome proliferator-activated receptor gamma	0.677388424
Calcium/calmodulin-dependent protein kinase type II subunit gamma	0.447478992
Serine/threonine-protein kinase D1	0.410130719
Peroxisome proliferator-activated receptor gamma	0.823263889
Homeodomain-interacting protein kinase 4	0.560606061
Mitogen-activated protein kinase kinase kinase 1	0.423868313
Beta-lactamase	0.209876543
CREB-binding protein	0.555555556
Carbonic anhydrase 13	0.758445946
cAMP-specific 3',5'-cyclic phosphodiesterase 4D	0.596218596
Cyclin-dependent kinase 2	0.355524253
B2 bradykinin receptor	0.618106452
Collagenase 3	0.429664596
Ephrin type-A receptor 6	0.428658537
Tyrosine-protein kinase Tec	0.515909091
Cationic trypsin	0.656445646
Heat shock protein HSP 90-alpha	0.891509434

PROTEIN FAMILY	AUC
Casein kinase II subunit alpha	0.487611305
Interstitial collagenase	0.3340383
Heat shock protein HSP 90-alpha	0.884433962
Serine/threonine-protein kinase BRSK2	0.3825
Dual specificity protein kinase TTK	0.535413745
Dual specificity tyrosine-phosphorylation-regulated kinase 1A	0.606060606
Casein kinase II subunit alpha	0.43757259
Matrilysin	0.280050505
Acetylcholinesterase	0.446666667
Myotonin-protein kinase	0.45047619
Cathepsin L1	0.620638395
Phenylethanolamine N-methyltransferase	0.707077206
Peroxisomal N(1)-acetyl-spermine/spermidine oxidase	1
Cationic trypsin	0.669113495
Mitogen-activated protein kinase 14	0.685930781
Glutamate receptor ionotropic, NMDA 1	0.173913043
Nischarin	0.353395062
Fibroblast growth factor receptor 2	0.562885802
Serine/threonine-protein kinase Nek11	0.43622449
Mitogen-activated protein kinase 4	0.626302083
Melanin-concentrating hormone receptor 1	0.640033595
Protein kinase C beta type	0.684848485
Peptidyl-prolyl cis-trans isomerase NIMA-interacting 1	0.737373737
Bile salt-activated lipase	0.282467532
Multidrug resistance-associated protein 1	0.1875
Aurora kinase A	0.562228082
Tyrosine-protein kinase BTK	0.51510989
Tyrosine-protein phosphatase non-receptor type 1	0.697845805
Calcium/calmodulin-dependent protein kinase type II subunit alpha	0.431318681
Metabotropic glutamate receptor 3	0.431372549
E3 ubiquitin-protein ligase XIAP	0.383805179
Trypsin	0.558333333
Cyclin-dependent kinase-like 1	0.366666667
Calcium/calmodulin-dependent protein kinase type II subunit beta	0.599087353
Thymidylate synthase	0.615440115
Inositol-trisphosphate 3-kinase A	0.5
Cationic trypsin	0.651355422
Peptidyl-prolyl cis-trans isomerase FKBP1A	0.814292129
Epidermal growth factor receptor	0.739113248
Serine/threonine-protein kinase Nek6	0.596354167
Serine/threonine-protein kinase Chk1	0.654656863
Peptidyl-prolyl cis-trans isomerase A	0.899774775
SRSF protein kinase 2	0.736680328
Transthyretin	0.821428571
Glycogen synthase kinase-3 beta	0.5260343
Beta-2 adrenergic receptor	0.605047319
UDP-3-O-[3-hydroxymyristoyl] N-acetylglucosamine deacetylase	0.375
Sodium channel protein type 9 subunit alpha	0.494208494
Purine nucleoside phosphorylase	0.43452381
DNA polymerase alpha catalytic subunit	0.382352941
Transthyretin	0.767857143
Serine/threonine-protein kinase N2	0.610491071
cAMP-dependent protein kinase catalytic subunit alpha	0.383865248

PROTEIN FAMILY	AUC
Transthyretin	0.75
cAMP-specific 3',5'-cyclic phosphodiesterase 4B	0.873516721
Matrilysin	0.302967172
C-X-C chemokine receptor type 3	0.652542373
Carbonic anhydrase 2	0.771464253
Poly [ADP-ribose] polymerase 1	0.307605422
Peptidyl-prolyl cis-trans isomerase NIMA-interacting 1	0.762866763
Coagulation factor X	0.515195081
Estrogen receptor	0.58901244
Fatty acid-binding protein, adipocyte	0.789473684
Aurora kinase A	0.592325203
Heat shock protein HSP 90-alpha	0.867924528
Serine/threonine-protein kinase pim-1	0.677814352
Myosin light chain kinase, smooth muscle	0.360942761
Estrogen receptor beta	0.708467454
Mitogen-activated protein kinase 14	0.672527396
3-dehydroquinate dehydratase	0.25
Carbonic anhydrase 7	0.794187325
TRAF2 and NCK-interacting protein kinase	0.630952381
Renin	0.796401515
Apoptosis regulator Bcl-2	0.809655172
Thymidylate synthase	0.534090909
Acetylcholinesterase	1
Urokinase-type plasminogen activator	0.548362418
Ribonuclease pancreatic	0.344720497
Inosine-5'-monophosphate dehydrogenase 2	0.781007752
Baculoviral IAP repeat-containing protein 2	nan
Calcium/calmodulin-dependent protein kinase type II subunit beta	0.385897436
Death-associated protein kinase 3	0.640858209
Glucocorticoid receptor	0.618232558
Proprotein convertase subtilisin/kexin type 9	0.25
S-adenosylmethionine decarboxylase proenzyme	0.545454545
Alcohol dehydrogenase class 4 mu/sigma chain	0.366666667
Sodium- and chloride-dependent glycine transporter 1	0.848484848
Sodium channel protein type 4 subunit alpha	0.307692308
Queuine tRNA-ribosyltransferase	0.707142857
Transthyretin	0.607142857
Thermolysin	0.624864865
Ribosomal protein S6 kinase alpha-6	0.623759921
Metabotropic glutamate receptor 8	0
Coagulation factor VII	0.655088452
Tyrosine-protein kinase SYK	0.375532822
Sodium channel protein type 9 subunit alpha	0.403474903
Beta-lactamase	0.24702381
Androgen receptor	0.615089452
Calcium/calmodulin-dependent protein kinase type II subunit gamma	0.350694444
Trypsin	0.916666667
Type II inositol 1,4,5-trisphosphate 5-phosphatase	0.375
Nischarin	0.324845679
Dipeptidyl peptidase 8	0.504220546
Glutamate carboxypeptidase 2	0.517316017
Peptidyl-prolyl cis-trans isomerase FKBP4	0.357142857
SRSF protein kinase 2	0.531762295

PROTEIN FAMILY	AUC
Muscarinic acetylcholine receptor M4	0.611111111
Serine/threonine-protein kinase pim-1	0.692725477
M1 family aminopeptidase	0
Methionine aminopeptidase 1	0.77173913
Phosphatidylinositol 5-phosphate 4-kinase type-2 gamma	0.547281324
Dehydrosqualene synthase	0.722222222
Microtubule-associated serine/threonine-protein kinase 1	0.349794239
Thymidylate kinase	0.821428571
Dual specificity mitogen-activated protein kinase kinase 6	0.49469697
Ribosomal protein S6 kinase beta-1	0.31723356
Focal adhesion kinase 1	0.53287037
Mitogen-activated protein kinase kinase kinase 7	0.547064306
Kallikrein-7	0
Glucocorticoid receptor	0.419982773
Thymidylate synthase	0.561774461
Kinesin-like protein KIF11	0.826923077
cAMP-dependent protein kinase catalytic subunit alpha	0.441999417
Androgen receptor	0.651481083
Cyclin-dependent kinase 2	0.348746529
Cationic trypsin	0.672127016
Serum albumin	0.354166667
Hepatocyte growth factor receptor	0.472956307
Farnesyl pyrophosphate synthase	0.424305556
Retinoic acid receptor beta	0.616614256
Serum albumin	0.409722222
Serum albumin	0.305555556
Dihydrofolate reductase	0.606483227
Stromelysin-1	0.408124242
Epidermal growth factor receptor	0.746525424
Hydroxyacylglutathione hydrolase, mitochondrial	1
Heat shock protein HSP 90-alpha	0.884433962
Cathepsin B	0.635495985
Mitogen-activated protein kinase 10	0.554274892
Receptor-interacting serine/threonine-protein kinase 4	0.478571429
Ribonuclease pancreatic	0.416149068
Glutamate carboxypeptidase 2	0.689393939
Lysozyme	0.87037037
5-hydroxytryptamine receptor 3A	0.701096491
Dual specificity tyrosine-phosphorylation-regulated kinase 1B	0.442176871
Trifunctional purine biosynthetic protein adenosine-3	0.707236842
Amine oxidase [flavin-containing] A	0.625244413
Beta-lactamase OXA-10	0
Proto-oncogene tyrosine-protein kinase Src	0.611526252
Vitamin D3 receptor	0.244897959
Muscle, skeletal receptor tyrosine-protein kinase	0.384567901
Gamma-aminobutyric acid receptor subunit beta-1	0.388888889
Myosin-IIIb	0.474014337
Mitogen-activated protein kinase 7	0.392397661
Mitogen-activated protein kinase kinase kinase 2	0.436842105
Macrophage metalloelastase	0.365414916
Carboxypeptidase A1	0.162340619
cAMP-specific 3',5'-cyclic phosphodiesterase 4C	0.678571429
Carbonic anhydrase 2	0.765011848

PROTEIN FAMILY	AUC
cAMP-dependent protein kinase catalytic subunit alpha	0.536869717
Activated CDC42 kinase 1	0.603654168
Dual specificity mitogen-activated protein kinase kinase 1	0.403790087
5-hydroxytryptamine receptor 3E	0.7734375
Multidrug resistance-associated protein 1	0.28125
Mitogen-activated protein kinase 10	0.560606061
Coagulation factor X	0.562637393
Collagenase 3	0.378484472
Caspase-3	0.909090909
cGMP-dependent protein kinase 1	0.408602151
Ephrin type-B receptor 3	0.412683824
Prothrombin	0.430903809
Aldose reductase	0.472222222
Orotidine 5'-phosphate decarboxylase	0.457142857
Myotonin-protein kinase	0.485714286
Dipeptidyl peptidase 4	0.708558939
Gamma-aminobutyric acid receptor subunit beta-2	0.512916667
Prostaglandin G/H synthase 1	0.5
Insulin-like growth factor 1 receptor	0.712702472
cAMP-specific 3',5'-cyclic phosphodiesterase 4D	0.60968661
Tyrosine-protein kinase Fer	0.499074074
Acetylcholinesterase	0.46
5-hydroxytryptamine receptor 6	0.487766033
Matrilysin	0.341035354
Serine/threonine-protein kinase TAO3	0.337197581
ALK tyrosine kinase receptor	0.573863636
Queuine tRNA-ribosyltransferase	0.657142857
Carbonic anhydrase 2	0.767668173
Retinoic acid receptor RXR-alpha	0.505709427
Nitric oxide synthase, inducible	0.636624244
Histamine N-methyltransferase	0.833333333
Histone deacetylase 2	0.894736842
[Pyruvate dehydrogenase [lipoamide]] kinase isozyme 1, mitochondrial	0
Tyrosine-protein phosphatase non-receptor type 1	0.734249556
Prostaglandin G/H synthase 1	0.5
Phosphatidylinositol 4,5-bisphosphate 3-kinase catalytic subunit gamma isoform	0.645114943
Egl nine homolog 1	0.3
Casein kinase I isoform gamma-2	0.552663438
Calcium/calmodulin-dependent protein kinase type 1D	0.469732704
Fibroblast growth factor 2	0.75
TGF-beta receptor type-1	0.466675448
Thymidylate synthase	0.701298701
Cyclin-dependent kinase 2	0.3588927
Protein kinase C iota type	0.503225806
Protein S100-B	0
Stromelysin-1	0.464368995
Serine/threonine-protein kinase Nek11	0.380102041
Mitogen-activated protein kinase 14	0.687786101
Carboxypeptidase A1	0.16375448
Lethal(3)malignant brain tumor-like protein 1	0.291666667
Transthyretin	0.857142857
cAMP-dependent protein kinase catalytic subunit alpha	0.441416497
Tyrosine-protein kinase ABL1	0.583474178

PROTEIN FAMILY	AUC
Serine/threonine-protein kinase PRP4 homolog	0.740041929
Glutathione S-transferase Mu 2	0.5
Serine/threonine-protein kinase MRCK alpha	0.518442623
Dipeptidyl peptidase 1	0.5
Trypsin	0.991666667
Carbonic anhydrase 2	0.755141595
S-methyl-5'-thioadenosine phosphorylase	0.397435897
Mitogen-activated protein kinase kinase kinase 4	0.513852814
Prostaglandin G/H synthase 1	0.6
Somatostatin receptor type 2	0.74967623
Mitogen-activated protein kinase 6	0.457885305
Serine/threonine-protein kinase Nek6	0.725260417
Aldose reductase	0.666666667
Serine/threonine-protein kinase pim-1	0.716886109
Corticotropin-releasing factor receptor 2	nan
Estrogen receptor	0.668829425
Vascular endothelial growth factor receptor 2	0.520462731
Integrin beta-2	0.952083333
Cationic trypsin	0.630704365
Calcium/calmodulin-dependent protein kinase type II subunit delta	0.410606061
Adenosine receptor A2a	0.374048287
Integrin alpha-L	0.101626016
Trypsin	0.841666667
Dehydrosqualene synthase	0.736111111
Peroxisome proliferator-activated receptor gamma	0.812413194
Cationic trypsin	0.657773953
Myosin light chain kinase 2, skeletal/cardiac muscle	0.558501684
Receptor-type tyrosine-protein phosphatase alpha	0.921497585
Cathepsin K	0.508081074
TGF-beta receptor type-2	0.496867168
DNA polymerase alpha catalytic subunit	0.421568627
Sodium channel protein type 9 subunit alpha	0.569498069
Cathepsin K	0.483127139
Nischarin	0.775462963
Coagulation factor X	0.550761327
3-phosphoinositide-dependent protein kinase 1	0.525480769
Ephrin type-A receptor 7	0.494212963
Tyrosine-protein kinase Fes/Fps	0.520855615
N-acetylated-alpha-linked acidic dipeptidase 2	0.263157895
Prothrombin	0.441742193
Nitric oxide synthase, endothelial	0.418016482
Inosine-5'-monophosphate dehydrogenase 2	0.784883721
Proto-oncogene tyrosine-protein kinase Src	0.621562882
Fibroblast growth factor 1	0.606837607
SHC-transforming protein 1	1
Urokinase-type plasminogen activator	0.543158369
Cholinesterase	0.830284553
Histone deacetylase 6	0.659574468
Receptor-type tyrosine-protein phosphatase F	0.795454545
Thymidylate synthase	0.61976912
Caspase-4	0.716666667
Gamma-aminobutyric acid receptor subunit alpha-4	0.48046875
Deoxyuridine 5'-triphosphate nucleotidohydrolase, mitochondrial	0.737196765

PROTEIN FAMILY	AUC
3-phosphoinositide-dependent protein kinase 1	0.342533937
Ephrin type-A receptor 7	0.433962264
Phosphatidylinositol 4,5-bisphosphate 3-kinase catalytic subunit alpha isoform	0.799404117
Beta-secretase 1	0.819206958
Insulin-like growth factor 1 receptor	0.724637681
Dipeptidyl peptidase 4	0.727907208
Leukotriene A-4 hydrolase	1
Myosin light chain kinase, smooth muscle	0.437373737
MAP kinase-interacting serine/threonine-protein kinase 1	0.531313131
Histone deacetylase 7	0.375
Cysteinyl leukotriene receptor 1	0.46031746
Phosphatidylinositol 4,5-bisphosphate 3-kinase catalytic subunit gamma isoform	0.670258621
Lanosterol 14-alpha demethylase	0.851851852
Tyrosine-protein phosphatase non-receptor type 11	0.611888112
Lymphokine-activated killer T-cell-originated protein kinase	0
3-phosphoinositide-dependent protein kinase 1	0.558371041
Prothrombin	0.521764032
3-oxoacyl-[acyl-carrier-protein] synthase 3	0.928571429
Prothrombin	0.433824853
Dual specificity mitogen-activated protein kinase kinase 7	0.35380117
Phosphatidylinositol 5-phosphate 4-kinase type-2 beta	0.581118881
Trypsin	0.941666667
Carbonic anhydrase 2	0.770170179
Thymidylate synthase	0.579365079
Tyrosine-protein kinase SYK	0.460441176
5-hydroxytryptamine receptor 4	0.53256705
Cathepsin G	0.420918367
Tyrosine-protein kinase Lyn	0.645531915
Glutathione S-transferase P	0.65
Thymidylate synthase	0.577922078
5'-methylthioadenosine/S-adenosylhomocysteine nucleosidase	0.162790698
Beta-lactamase	0.198275862
Ephrin type-B receptor 4	0.523921833
Arginase-1	0.271428571
STE20/SPS1-related proline-alanine-rich protein kinase	0.395061728
5-hydroxytryptamine receptor 4	0.581736909
Cytidine deaminase	0.695238095
Peptidyl-prolyl cis-trans isomerase FKBP1A	0.758900507
3-hydroxy-3-methylglutaryl-coenzyme A reductase	0.801470588
Alcohol dehydrogenase E chain	0.225
Thymidylate synthase	0.65007215
Dihydrofolate reductase	0.596260577
Gamma-aminobutyric acid receptor subunit alpha-5	0.544221509
D(2) dopamine receptor	0.58484031
Receptor-type tyrosine-protein phosphatase F	0.613636364
RAC-alpha serine/threonine-protein kinase	0.630906769
Galectin-9	0.890740741
Myosin light chain kinase, smooth muscle	0.49023569
Beta-lactamase	0.230769231
Prothrombin	0.438280992
Rho-associated protein kinase 1	0.361333333
Growth factor receptor-bound protein 2	0.484848485
Cationic trypsin	0.649035714

PROTEIN FAMILY	AUC
Mitogen-activated protein kinase kinase kinase 15	0.360632184
Tyrosine-protein kinase Lck	0.678282172
Estrogen receptor	0.740591999
cAMP-specific 3',5'-cyclic phosphodiesterase 4B	0.84600863
Cholinesterase	0.840370935
Uridine 5'-monophosphate synthase	0.2
Papain	0.450605394
Glycogen phosphorylase, liver form	0
Cathepsin S	0.363752335
Purine nucleoside phosphorylase	0.640945946
RAC-alpha serine/threonine-protein kinase	0.602916135
Macrophage migration inhibitory factor	1
Gamma-aminobutyric acid receptor subunit gamma-3	0.333333333
Fibroblast growth factor receptor 2	0.547067901
Beta-1 adrenergic receptor	0.570793748
Heat shock protein HSP 90-alpha	0.875
Peroxisome proliferator-activated receptor delta	0.645502646
Serine/threonine-protein kinase NLK	0.535087719
Heat shock protein HSP 90-alpha	0.891509434
Calcium/calmodulin-dependent protein kinase type II subunit beta	0.444871795
Leukotriene A-4 hydrolase	1
cGMP-dependent protein kinase 2	0.559482759
Bcl2 antagonist of cell death	0.787828947
Prostaglandin G/H synthase 1	0.5
Cholinesterase	0.839519817
Serum albumin	0.368055556
Dihydrofolate reductase	0.133838384
Androgen receptor	0.647057386
Serum albumin	0.465277778
Tryptase alpha/beta-1	0.210561497
cAMP-specific 3',5'-cyclic phosphodiesterase 4B	0.879719525
Peroxisome proliferator-activated receptor gamma	0.733940972
Prothrombin	0.424416978
Proto-oncogene tyrosine-protein kinase Src	0.551330891
Retinoic acid receptor gamma	0.577293578
Dihydroorotate dehydrogenase (quinone), mitochondrial	0.691287879
Caspase-9	0.666666667
A disintegrin and metalloproteinase with thrombospondin motifs 1	1
Poly [ADP-ribose] polymerase 3	1
Cathepsin S	0.702150294
Serine/threonine-protein kinase ULK3	0.52876377
Peptidyl-prolyl cis-trans isomerase FKBP1A	0.744002181
Carboxypeptidase A1	0.199148746
Angiotensin-converting enzyme	0.519361413
Transient receptor potential cation channel subfamily M member 6	0.696721311
Sialidase-2	0.333333333
Nischarin	0.303240741
Proto-oncogene tyrosine-protein kinase Src	0.538608059
Nuclear receptor subfamily 1 group I member 2	0.5
Mitogen-activated protein kinase kinase kinase 19	0.533442982
Casein kinase I isoform delta	0.533532042
Plasmepsin-2	0.769293038
Thymidylate synthase	0.594516595

PROTEIN FAMILY	AUC
Insulin receptor	0.689134809
Dihydrofolate reductase	0.244883041
Receptor-type tyrosine-protein phosphatase C	0.587962963
Urokinase-type plasminogen activator	0.530276958
Tyrosine-protein phosphatase non-receptor type 1	0.621299871
Acetylcholinesterase	0.566666667
Arginase-1	0.328571429
Pteridine reductase 1	0.731601732
Tyrosine-protein kinase BTK	0.567307692
Estrogen receptor	0.637428405
Cyclin-dependent kinase-like 1	0.542372881
Fibroblast growth factor 1	0.47008547
Estrogen receptor	0.780170485
Prostaglandin G/H synthase 1	1
[Pyruvate dehydrogenase [lipoamide]] kinase isozyme 1, mitochondrial	0
Leukotriene A-4 hydrolase	1
Thymidylate synthase	0.540630182
Cationic trypsin	0.664137493
Multidrug resistance-associated protein 1	0.15625
Cyclin-dependent kinase 2	0.371754042
Glutamate receptor ionotropic, kainate 2	0.447568389
Serine/threonine-protein kinase SIK2	0.537272727
Purine nucleoside phosphorylase	0.536351351
Leukotriene A-4 hydrolase	1
Cathepsin L1	0.644176927
3-phosphoinositide-dependent protein kinase 1	0.361213235
Epidermal growth factor receptor	0.710052966
Growth hormone secretagogue receptor type 1	0.531637717
Beta-secretase 1	0.81301592
3-dehydroquinate dehydratase	0.883333333
Baculoviral IAP repeat-containing protein 2	0.5
Mineralocorticoid receptor	0.410922587
Calcium/calmodulin-dependent protein kinase type II subunit delta	0.516746411
Calcium/calmodulin-dependent protein kinase type II subunit delta	0.376555024
Tyrosine-protein kinase HCK	0.568895349
Macrophage migration inhibitory factor	0.625
Uridine 5'-monophosphate synthase	0.45
Coagulation factor VII	0.825581395
Multidrug resistance-associated protein 1	0.28125
Glycogen phosphorylase, muscle form	0.285714286
Tyrosine-protein kinase ABL1	0.574169868
Nitric oxide synthase, endothelial	0.401676613
Papain	0.48451387
Serine/threonine-protein kinase DCLK3	0.413968548
Chymotrypsin-C	0.414274322
Vascular endothelial growth factor receptor 2	0.676688857
Protein delta homolog 1	0.5
Papain	0.448160535
Trypsin	0.958333333
Dihydrofolate reductase	0.593940497
Alcohol dehydrogenase E chain	0.294444444
Thermolysin	0.572972973
Aldose reductase	0.472222222

PROTEIN FAMILY	AUC
Bromodomain-containing protein 4	0.5
Cytoplasmic tyrosine-protein kinase BMX	0.421539961
Dihydrofolate reductase	0.541715949
Suppressor of tumorigenicity 14 protein	0.250786164
Acetylcholinesterase	0.366666667
Integrin alpha-IIb	0.411600811
Mineralocorticoid receptor	0.569989396
Prothrombin	0.523291333
Heat shock protein HSP 90-beta	0.933333333
Uridine 5'-monophosphate synthase	0.2
Glutamate receptor ionotropic, kainate 4	0.75
Leukotriene A-4 hydrolase	1
Vascular endothelial growth factor receptor 2	0.640347048
Serum albumin	0.409722222
Cathepsin K	0.534745986
Breast cancer type 1 susceptibility protein	0.333333333
Transient receptor potential cation channel subfamily M member 6	0.639344262
Integrin alpha-L	0.857723577
MAP/microtubule affinity-regulating kinase 3	0.523668639
Cationic trypsin	0.64911264
Urokinase-type plasminogen activator	0.571898312
Serum albumin	0.465277778
Glutathione S-transferase P	0.625
Dipeptidyl peptidase 4	0.708484427
Renin	0.781841856
Glutamate receptor ionotropic, kainate 4	0.75
Dipeptidyl peptidase 4	0.710471412
Transthyretin	0.785714286
Cyclin-dependent kinase 2	0.349113996
WD repeat-containing protein 5	0.27173913
Casein kinase I isoform gamma-3	0.519592476
Caspase-8	0.549450549
Thymidylate synthase	0.541125541
Transthyretin	0.785714286
cAMP-dependent protein kinase catalytic subunit alpha	0.549402507
Urokinase-type plasminogen activator	0.543498327
Carbonic anhydrase 2	0.771515139
Peptidyl-prolyl cis-trans isomerase A	0.931869369
Ribosomal protein S6 kinase alpha-1	0.500711575
Serine/threonine-protein kinase Sgk3	0.642602496
Inosine-5'-monophosphate dehydrogenase 1	0.506944444
Tyrosine-protein kinase Fer	0.459259259
Nitric oxide synthase, inducible	0.642483619
Cathepsin L1	0.63751938
Casein kinase II subunit alpha	0.498328088
Kinesin-like protein KIF11	0.740384615
Beta-secretase 1	0.837780071
Trypsin	0.875
High affinity nerve growth factor receptor	0.668187744
cAMP-dependent protein kinase catalytic subunit beta	0.637962963
Sodium- and chloride-dependent glycine transporter 1	0.363636364
Metabotropic glutamate receptor 3	0.431372549
Eukaryotic translation initiation factor 2-alpha kinase 4	0.475295381

PROTEIN FAMILY	AUC
Adenosylhomocysteinase	0.274129747
Phosphatidylinositol 4,5-bisphosphate 3-kinase catalytic subunit gamma isoform	0.681034483
Peroxisome proliferator-activated receptor gamma	0.748611111
Cathepsin B	0.591292135
Tyrosine-protein kinase HCK	0.575290698
Peptidyl-prolyl cis-trans isomerase A	0.984234234
Guanine deaminase	0.222222222
Thyroid hormone receptor beta	0.5
Acetylcholinesterase	1
Tyrosine-protein kinase CSK	0.391872279
Nitric oxide synthase, endothelial	0.421568627
Prostaglandin G/H synthase 1	0.6
Leukotriene A-4 hydrolase	0.833333333
Prothrombin	0.446984409
Serum albumin	0.493055556
Receptor tyrosine-protein kinase erbB-3	0.447916667
Carbonic anhydrase 2	0.755697078
Transthyretin	0.928571429
TGF-beta receptor type-1	0.596933472
Coagulation factor X	0.531092307
Casein kinase I isoform gamma-3	0.39968652
Insulin receptor	0.682763246
Nitric oxide synthase, endothelial	0.450980392
Neuronal acetylcholine receptor subunit alpha-7	0.316979317
Cathepsin K	0.50662455
N(G),N(G)-dimethylarginine dimethylaminohydrolase 1	0.222222222
Proto-oncogene tyrosine-protein kinase Src	0.538656899
Urokinase-type plasminogen activator	0.532826391
Serum albumin	0.395833333
Sodium channel protein type 9 subunit alpha	0.480694981
Cyclin-dependent kinase 18	0.419799499
Tyrosine-protein phosphatase non-receptor type 1	0.688959368
Myosin-IIIb	0.551912568
Coagulation factor X	0.517316379
Coagulation factor X	0.53374325
cGMP-specific 3',5'-cyclic phosphodiesterase	0.907224959
NADPH oxidase 4	0.363520408
Sialidase-2	0.666666667
Serum albumin	0.451388889
Proto-oncogene tyrosine-protein kinase Src	0.525225885
Tyrosine-protein kinase Lck	0.672132787
Hepatocyte growth factor receptor	0.600378788
Orotidine 5'-phosphate decarboxylase	0.4
Eukaryotic translation initiation factor 2-alpha kinase 4	0.384920635
Tyrosine-protein kinase Lck	0.673582642
Beta-secretase 1	0.838590802
Beta-secretase 1	0.806382665
Dihydrofolate reductase	0.15993266
Thymidylate synthase	0.570480929
Tyrosine-protein phosphatase non-receptor type 1	0.770637985
Focal adhesion kinase 1	0.453240741
Serine/threonine-protein kinase Nek2	0.546538462
Lethal(3)malignant brain tumor-like protein 1	0

PROTEIN FAMILY	AUC
Coagulation factor X	0.548916836
cAMP-specific 3',5'-cyclic phosphodiesterase 4D	0.76029526
Glutathione S-transferase P	0.7875
Orotidine 5'-phosphate decarboxylase	0.442857143
Fatty acid-binding protein, adipocyte	0.569078947
Mitogen-activated protein kinase 10	0.473593074
BDNF/NT-3 growth factors receptor	0.58030303
Ribosomal protein S6 kinase alpha-1	0.498814042
Guanine deaminase	0.333333333
Chymotrypsinogen A	0.711643836
Queuine tRNA-ribosyltransferase	0.614285714
cAMP-dependent protein kinase catalytic subunit alpha	0.516467502
Bacterial leucyl aminopeptidase	0.303030303
Suppressor of tumorigenicity 14 protein	0.43081761
Carbonic anhydrase 2	0.766990707
Neuronal acetylcholine receptor subunit alpha-7	0.496873497
Tyrosine-protein kinase HCK	0.533430233
Glycogen phosphorylase, liver form	0.142857143
Mineralocorticoid receptor	0.414103924
Receptor-type tyrosine-protein phosphatase C	0.726851852
Serine/threonine-protein kinase 32A	0.632075472
Mitogen-activated protein kinase 14	0.668452629
cGMP-dependent 3',5'-cyclic phosphodiesterase	0.5
Glutathione S-transferase P	0.8375
Aldose reductase	0.611111111
Cyclin-dependent kinase 2	0.361587457
S-adenosylmethionine decarboxylase proenzyme	0.472727273
RAC-alpha serine/threonine-protein kinase	0.609088974
Progesterone receptor	0.503581118
Hepatocyte growth factor receptor	0.608929532
Cathepsin B	0.561329588
Cationic trypsin	0.634981127
Estrogen receptor	0.669131466
Chymase	0.386269071
Cathepsin S	0.679696695
Multidrug resistance-associated protein 1	0.21875
Dihydrofolate reductase	0.6065499
Inosine-5'-monophosphate dehydrogenase 1	0.686805556
Tyrosine-protein kinase Fes/Fps	0.414438503
Kinesin-like protein KIF11	0.836538462
Proteasome subunit beta type-2	0.111111111
Mu-type opioid receptor	0.567921506
Acetylcholinesterase	1
Transthyretin	0.928571429
Receptor tyrosine-protein kinase erbB-3	0.463888889
Fatty-acid amide hydrolase 1	0.55947865
Mitogen-activated protein kinase 14	0.661412817
Liver carboxylesterase 1	0.486519608
Bone morphogenetic protein 1	0.777966102
Tryptase alpha/beta-1	0.222259358
Egl nine homolog 1	0.454545455
Phenylethanolamine N-methyltransferase	0.754595588
Glycogen synthase kinase-3 beta	0.522873939

PROTEIN FAMILY	AUC
Cyclin-dependent kinase 2	0.430732484
cAMP-dependent protein kinase catalytic subunit alpha	0.440687846
Dihydrofolate reductase	0.584842144
Trypanothione reductase	0.740652174
Serine/threonine-protein kinase tousled-like 1	0.591679507
Mineralocorticoid receptor	0.41330859
Retinoic acid receptor RXR-alpha	0.480442177
Vascular endothelial growth factor receptor 2	0.588508131
Serine/threonine-protein kinase MARK1	0.546284224
Alpha-2A adrenergic receptor	0.415664027
Macrophage-stimulating protein receptor	0.644654088
G protein-coupled receptor kinase 4	0.508951407
Retinoic acid receptor RXR-alpha	0.606049563
Hepatocyte growth factor receptor	0.695340028
Macrophage metalloelastase	0.434480042
Gamma-aminobutyric acid receptor subunit gamma-2	0.514355769
Myosin light chain kinase, smooth muscle	0.468686869
Serum albumin	0.395833333
NUAK family SNF1-like kinase 2	0.499125874
Beta-lactamase	0.228021978
Kallikrein-1	0.513429752
cAMP-specific 3',5'-cyclic phosphodiesterase 4B	0.862998921
Myelin-associated glycoprotein	1
Protein kinase C delta type	0.368516344
Urokinase-type plasminogen activator	0.541556414
Proto-oncogene tyrosine-protein kinase Src	0.562686203
Proto-oncogene tyrosine-protein kinase Src	0.567350427
Fibroblast growth factor receptor 3	0.637280059
Peroxisome proliferator-activated receptor delta	0.502645503
Dihydrofolate reductase	0.111111111
Mitogen-activated protein kinase kinase kinase 19	0.568868981
Carbonic anhydrase 2	0.76234059
Nitric oxide synthase, endothelial	0.41332765
ALK tyrosine kinase receptor	0.5625
Neuromedin-K receptor	0.729815456
Fibroblast growth factor receptor 2	0.610339506
Thymidylate synthase	0.585820896
Trypsin	0.958333333
Lanosterol 14-alpha demethylase	0
Prothrombin	0.598733938
Thymidylate kinase	0.794642857
Calcium/calmodulin-dependent protein kinase type 1G	0.430899609
Thymidylate synthase	0.587064677
Serine/threonine-protein kinase N2	0.465959821
Mitogen-activated protein kinase 8	0.552492342
Serine/threonine-protein kinase tousled-like 1	0.466872111
Adenosine receptor A1	0.465320528
Estradiol 17-beta-dehydrogenase 1	0.833333333
Liver carboxylesterase 1	0.558352187
Beta-lactamase	0.708333333
Receptor-type tyrosine-protein phosphatase C	0.685185185
Tyrosine-protein phosphatase non-receptor type 6	0.963636364
Neutrophil collagenase	0.623956894

PROTEIN FAMILY	AUC
Catechol O-methyltransferase	0.2
Heat shock protein HSP 90-alpha	0.887971698
Thyroid hormone receptor beta	0.262820513
Collagenase 3	0.462832298
Beta-lactamase	0.200892857
Progesterone receptor	0.505642973
Kinesin-like protein KIF11	0.740384615
Serine/threonine-protein kinase B-raf	0.636160714
Fibroblast growth factor receptor 2	0.59066358
Tyrosine-protein phosphatase non-receptor type 1	0.641493534
Beta-secretase 1	0.736512382
Heat shock protein HSP 90-alpha	0.858490566
Coagulation factor IX	0.40761079
Aurora kinase B	0.612814591
Glycogen phosphorylase, muscle form	0.285714286
Trypsin-3	0.440677966
Hepatocyte growth factor receptor	0.566287879
Inositol-trisphosphate 3-kinase A	0.5
Cationic trypsin	0.623270751
Angiotensin-converting enzyme	0.548233696
Serine/threonine-protein kinase Chk1	0.649019608
D(2) dopamine receptor	0.526750712
Integrin alpha-IIb	0.504812563
Eukaryotic translation initiation factor 2-alpha kinase 4	0.373582766
Sodium- and chloride-dependent glycine transporter 1	0.363636364
Alcohol dehydrogenase E chain	0.255555556
Platelet-derived growth factor receptor beta	0.498684211
Aldose reductase	0.611111111
Peptidyl-prolyl cis-trans isomerase NIMA-interacting 1	0.788119288
Chymotrypsinogen A	0.618055556
Heat shock protein HSP 90-alpha	0.853773585
Beta-secretase 1	0.823039505
Alcohol dehydrogenase E chain	0.277777778
Dihydrofolate reductase	0.462719298
Phosphatidylinositol 4,5-bisphosphate 3-kinase catalytic subunit gamma isoform	0.655890805
Aldo-keto reductase family 1 member C3	0.666666667
Carbonic anhydrase 1	0.702643144
Acetylcholinesterase	1
Dual specificity tyrosine-phosphorylation-regulated kinase 1B	0.432823129
S-adenosylmethionine decarboxylase proenzyme	0.645454545
Dehydrosqualene synthase	0.881944444
Ribosyldihydronicotinamide dehydrogenase [quinone]	0.133333333
Carbonic anhydrase 2	0.762496638
Alcohol dehydrogenase 4	0.71875
Fibroblast growth factor receptor 2	0.507211538
Trifunctional purine biosynthetic protein adenosine-3	0.720394737
Cathepsin L1	0.616069311
Glucagon receptor	0.316326531
Prothrombin	0.427014354
Coagulation factor X	0.566898357
Cathepsin B	0.583203833
Acetylcholinesterase	1
Tyrosine-protein kinase ABL1	0.560626471

PROTEIN FAMILY	AUC
Estrogen receptor	0.708888939
Acidic mammalian chitinase	1
Eukaryotic translation initiation factor 2-alpha kinase 4	0.37414966
Ribosyldihydronicotinamide dehydrogenase [quinone]	0.116666667
Serine/threonine-protein kinase Nek2	0.505
Furin	0.893939394
Serine/threonine-protein kinase 33	0.603913043
Insulin-like growth factor 1 receptor	0.629831933
Glycogen synthase kinase-3 beta	0.572445191
3-dehydroquinate dehydratase	0.773333333
Lethal(3)malignant brain tumor-like protein 1	0.083333333
Glutamate receptor 1	49122
Orotidine 5'-phosphate decarboxylase	0.571428571
Serine/threonine-protein kinase BRSK2	0.39
Coagulation factor V	0.75
Trifunctional purine biosynthetic protein adenosine-3	0.694078947
Cholinesterase	0.818076728
Equilibrative nucleoside transporter 1	0.633846154
Cholinesterase	0.792301829
Rho-associated protein kinase 1	0.443581081
Peptidyl-prolyl cis-trans isomerase NIMA-interacting 1	0.707311207
Disintegrin and metalloproteinase domain-containing protein 17	0.604210306
Amine oxidase [flavin-containing] B	0.598880779
Prothrombin	0.615594542
3-phosphoinositide-dependent protein kinase 1	0.373755656
Myosin-IIIb	0.399641577
Lysosomal alpha-glucosidase	0.19047619
Glycogen phosphorylase, muscle form	0.285714286
Thermolysin	0.606486486
Integrin alpha-L	0.768292683
Tyrosine-protein kinase ABL1	0.535722811
Purine nucleoside phosphorylase	0.650472973
Mitogen-activated protein kinase kinase kinase 1	0.441358025
Receptor tyrosine-protein kinase erbB-4	0.498979592
Mitogen-activated protein kinase 14	0.566826189
Death-associated protein kinase 3	0.616837687
Serine/threonine-protein kinase MRCK alpha	0.427419355
Heat shock protein HSP 90-alpha	0.884433962
Carbonic anhydrase 2	0.76444882
Aurora kinase A	0.520228758
Peptidyl-prolyl cis-trans isomerase A	0.971846847
Cationic trypsin	0.678857143
Tyrosine-protein phosphatase non-receptor type 1	0.718146718
Receptor-type tyrosine-protein phosphatase beta	0.6
Glutamate receptor 2	0.278125
Tyrosine-protein kinase Lyn	0.647234043
Multidrug resistance-associated protein 1	0.34375
Cyclin-dependent kinase 19	0.555113636
Cathepsin B	0.526685393
Peroxisomal N(1)-acetyl-spermine/spermidine oxidase	1
Serum albumin	0.465277778
UDP-3-O-[3-hydroxymyristoyl] N-acetylglucosamine deacetylase	0.5
Metallo-beta-lactamase L1	0.904761905

PROTEIN FAMILY	AUC
Retinoic acid receptor RXR-beta	0.603885135
Bone morphogenetic protein receptor type-1B	0.585743802
Prostaglandin E2 receptor EP3 subtype	0.514182425
Queuine tRNA-ribosyltransferase	0.657142857
Beta-lactamase	0.413333333
Tankyrase-2	0.75
Neutrophil collagenase	0.644901907
Glutathione S-transferase A1	0.333333333
Amine oxidase [flavin-containing] B	0.6
Insulin-like growth factor 1 receptor	0.70801364
Heat shock protein HSP 90-alpha	0.860849057
Neutrophil collagenase	0.66344294
Amine oxidase [flavin-containing] B	0.631970803
Prothrombin	0.5521262
Mitogen-activated protein kinase 1	0.726229508
5-hydroxytryptamine receptor 2C	0.323236398
Carbonic anhydrase 7	0.826846603
Beta-secretase 1	0.854510613
Thymidylate synthase	0.686868687
Peroxisome proliferator-activated receptor gamma	0.734895833
Cationic trypsin	0.695230836
Insulin-like growth factor 1 receptor	0.660084034
Serine/threonine-protein kinase VRK2	0.394827586
S-adenosylmethionine decarboxylase proenzyme	0.490909091
Eukaryotic translation initiation factor 2-alpha kinase 1	0.325862069
Ephrin type-B receptor 4	0.383423181
Casein kinase II subunit alpha	0.406600852
Multidrug resistance-associated protein 1	0.28125
Myosin light chain kinase, smooth muscle	0.431313131
Serum albumin	0.354166667
Queuine tRNA-ribosyltransferase	0.642857143
Dipeptidyl peptidase 4	0.693184641
Chymotrypsinogen A	0.668125
Acetylcholinesterase	1
Casein kinase II subunit alpha	0.428765002
Vitamin D3 receptor	0.255102041
Cyclin-dependent kinase 16	0.553267974
Thymidylate kinase	0.803571429
Methionine aminopeptidase 2	0.505589431
Coagulation factor XI	0.511029412
Caspase-1	0.579425794
Potassium voltage-gated channel subfamily H member 2	0.61926403
Transthyretin	0.964285714
Glycogen phosphorylase, muscle form	0.285714286
Myotonin-protein kinase	0.465986395
Urokinase-type plasminogen activator	0.547463378
Heat shock protein HSP 90-alpha	0.884433962
Serine/threonine-protein kinase N2	0.506696429
Cholinesterase	0.826105183
Macrophage metalloelastase	0.376838235
Transthyretin	0.375
Thyroid hormone receptor beta	0.086538462
Protein kinase C theta type	0.465722496

PROTEIN FAMILY	AUC
Macrophage metalloelastase	0.417804622
Acetylcholinesterase	1
Protein S100-B	0
Serine/threonine-protein kinase VRK2	0.629310345
Cathepsin L1	0.615312358
Neutrophil collagenase	0.65946394
Beta-lactamase	0.213141026
Peptidyl-prolyl cis-trans isomerase FKBP1A	0.542610174
Integrin alpha-L	0.666666667
Estrogen receptor	0.617527296
Uridine 5'-monophosphate synthase	0.2
Glutamate carboxypeptidase 2	0.374458874
Tyrosine-protein kinase ITK/TSK	0.600803506
Serine/threonine-protein kinase B-raf	0.635416667
Caspase-8	0.774725275
Beta-lactamase	0.219551282
Carbonic anhydrase 2	0.764238208
Tyrosine-protein kinase SYK	0.389526935
Tyrosine-protein kinase Lck	0.673132687
Alpha-galactosidase A	0.666666667
Leucine-rich repeat serine/threonine-protein kinase 2	0.487477639
Beta-3 adrenergic receptor	0.72008547
Peroxisome proliferator-activated receptor gamma	0.720746528
Tyrosine-protein kinase JAK2	0.57430923
Non-receptor tyrosine-protein kinase TYK2	0.603720238
Mitogen-activated protein kinase 10	0.522077922
Cyclin-dependent kinase 3	0.525
Beta-lactamase	0.230769231
Nitric oxide synthase, endothelial	0.410485934
Cyclin-dependent kinase 18	0.535087719
Chymotrypsinogen A	0.667294521
Estrogen receptor beta	0.582349785
Ribosyldihydronicotinamide dehydrogenase [quinone]	0.122222222
Corticotropin-releasing factor receptor 2	0
Inositol-trisphosphate 3-kinase B	0.5
5'-methylthioadenosine/S-adenosylhomocysteine nucleosidase	0.093023256
Tyrosine-protein phosphatase non-receptor type 1	0.610590182
Activated CDC42 kinase 1	0.400073028
Dihydrofolate reductase	0.532845055
Serine/threonine-protein kinase Chk2	0.655555556
E3 ubiquitin-protein ligase XIAP	0.602390852
Thymidylate synthase	0.70537108
AMP deaminase 2	0.802164502
Protein kinase C iota type	0.379032258
Phosphoglycerate kinase	0.7375
M1 family aminopeptidase	0
L-lactate dehydrogenase A chain	0.46875
Serine/threonine-protein kinase Chk2	0.613425926
Cationic trypsin	0.671124928
Ribosomal protein S6 kinase alpha-1	0.509962049
Carboxypeptidase B2	0.448275862
Serum albumin	0.395833333
Phosphatidylinositol 4-kinase alpha	0.5

PROTEIN FAMILY	AUC
Phosphatidylinositol 4,5-bisphosphate 3-kinase catalytic subunit gamma isoform	0.551005747
Carboxypeptidase A1	0.196460573
Cytosol aminopeptidase	0.630952381
Ribonuclease pancreatic	0.313664596
Progesterone receptor	0.518556701
Dihydrofolate reductase	0.44005848
6,7-dimethyl-8-ribityllumazine synthase	0.428571429
Calcium/calmodulin-dependent protein kinase type II subunit beta	0.524771838
Aldo-keto reductase family 1 member C3	0.722222222
Aurora kinase A	0.604052288
LIM domain kinase 2	0.566081871
Phosphatidylinositol 4,5-bisphosphate 3-kinase catalytic subunit gamma isoform	0.560344828
Serine/threonine-protein kinase 17B	0.564397047
Transthyretin	0.928571429
Megakaryocyte-associated tyrosine-protein kinase	0.417293233
Uncharacterized aarF domain-containing protein kinase 4	0.570833333
Serine/threonine-protein kinase ULK2	0.548185232
Cathepsin K	0.514889883
Thymidylate synthase	0.590909091
Ribosomal protein S6 kinase alpha-4	0.505989583
Peroxisome proliferator-activated receptor gamma	0.795920139
M-phase inducer phosphatase 3	0.571428571
TGF-beta receptor type-1	0.639423077
Serine/threonine-protein kinase Chk1	0.642401961
Sodium channel protein type 9 subunit alpha	0.55019305
Ribosyldihydronicotinamide dehydrogenase [quinone]	0.066666667
Fibroblast growth factor receptor 1	0.645138889
CREB-binding protein	0.5
TGF-beta receptor type-2	0.449617347
Serine/threonine-protein kinase PLK1	0.4526356
Tyrosine-protein kinase receptor TYRO3	0.552476415
Alpha-1A adrenergic receptor	0.798846676
Ribonuclease pancreatic	0.332298137
Adenosine deaminase	0.87345679
Peroxisome proliferator-activated receptor gamma	0.727690972
Dual specificity tyrosine-phosphorylation-regulated kinase 2	0.492801772
Epidermal growth factor receptor	0.556853814
Peptidyl-prolyl cis-trans isomerase A	0.896959459
Androgen receptor	0.411587154
Receptor activity-modifying protein 1	0.639455782
Phosphatidylinositol 4,5-bisphosphate 3-kinase catalytic subunit gamma isoform	0.5
Tyrosine-protein kinase ABL1	0.517886983
Transthyretin	0.785714286
Ephrin type-A receptor 5	0.505278592
Cationic trypsin	0.675534991
Adenosine kinase	0.966666667
Tyrosine-protein kinase ABL1	0.547184892
Thymidylate kinase	0.767857143
Carbonic anhydrase 2	0.75105042
Orotidine 5'-phosphate decarboxylase	0.542857143
Amyloid beta A4 protein	0.510764035
Dihydrofolate reductase	0.599672459
5-hydroxytryptamine receptor 3A	0.725657895

PROTEIN FAMILY	AUC
ALK tyrosine kinase receptor	0.588709677
Peptidyl-prolyl cis-trans isomerase NIMA-interacting 1	0.693362193
Caspase-4	0.5
Tyrosine-protein kinase Lck	0.668883112
Dual specificity mitogen-activated protein kinase kinase 6	0.590163934
Serine/threonine-protein kinase TAO3	0.530241935
3-phosphoinositide-dependent protein kinase 1	0.342533937
Prothrombin	0.617122358
Nitric oxide synthase, endothelial	0.450127877
5-hydroxytryptamine receptor 1B	0.530968432
C-C chemokine receptor type 5	0.62585034
Hepatocyte growth factor receptor	0.604133046
Carboxypeptidase A1	0.188069217
Serine/threonine-protein kinase Chk1	0.620833333
Cathepsin B	0.659414659
Cationic trypsin	0.60890697
cAMP-dependent protein kinase catalytic subunit alpha	0.395657243
Cathepsin S	0.686713445
Acetylcholinesterase	1
Leukotriene A-4 hydrolase	1
Alpha-galactosidase A	0
Carbonic anhydrase 1	0.690247485
Serine/threonine-protein kinase Nek2	0.519230769
Peptidyl-prolyl cis-trans isomerase FKBP1A	0.724597473
Cyclin-dependent kinase 13	0.481283422
Matrix metalloproteinase-9	0.342832793
TRAF2 and NCK-interacting protein kinase	0.546768707
3-oxoacyl-[acyl-carrier-protein] synthase 3	1
Orotidine 5'-phosphate decarboxylase	0.6
Serine/threonine-protein kinase SIK1	0.528061224
Coagulation factor X	0.543184245
Cyclin-dependent kinase 18	0.51754386
Thermolysin	0.605405405
Serum albumin	0.284722222
Estrogen receptor	0.680508323
Dihydrofolate reductase	0.369152047
Tyrosine-protein phosphatase non-receptor type 1	0.691793835
Cathepsin B	0.561227424
Nitric oxide synthase, endothelial	0.397271952
Chymotrypsinogen A	0.706487342
Renin	0.752604167
Phosphatidylinositol 4,5-bisphosphate 3-kinase catalytic subunit beta isoform	0.744350282
Cytidine deaminase	0.543269231
Somatostatin receptor type 1	0.515676568
Serine/threonine-protein kinase PknB	0.422011662
Maternal embryonic leucine zipper kinase	0.584775087
Multidrug resistance-associated protein 1	0.59375
Serine/threonine-protein kinase pim-1	0.68915863
Estrogen receptor	0.661971989
Estrogen receptor beta	0.726171316
Deoxyuridine 5'-triphosphate nucleotidohydrolase, mitochondrial	0.632075472
Serine/threonine-protein kinase MRCK beta	0.707589286
MAP kinase-activated protein kinase 2	0.663030303

PROTEIN FAMILY	AUC
Cyclin-dependent kinase 8	0.485632184
Renin	0.780894886
Thyroid hormone receptor beta	0.169871795
Phenylethanolamine N-methyltransferase	0.769117647
Trypanothione reductase	0.727826087
ATP-citrate synthase	0.782828283
Nitric oxide synthase, endothelial	0.410628019
Serum albumin	0.409722222
Aldose reductase	0.472222222
Histamine N-methyltransferase	0.166666667
Mitogen-activated protein kinase 14	0.623283396
Beta-secretase 1	0.840423874
cAMP-specific 3',5'-cyclic phosphodiesterase 4C	0.669642857
Dual specificity protein phosphatase 3	0.607142857
Peptidyl-prolyl cis-trans isomerase FKBP4	0.5
Cationic trypsin	0.663773478
Angiotensin-converting enzyme	0.571331522
Heat shock protein HSP 90-alpha	0.870283019
Deoxyuridine 5'-triphosphate nucleotidohydrolase, mitochondrial	0.602425876
Beta-secretase 1	0.784271816
Peroxisome proliferator-activated receptor gamma	0.773177083
Kinesin-like protein KIF11	0.572115385
Corticosteroid 11-beta-dehydrogenase isozyme 1	0.593406593
Estrogen receptor beta	0.650527539
Dihydrofolate reductase	0.539168411
Integrin alpha-L	0.890243902
Receptor-type tyrosine-protein phosphatase F	0.704545455
Tyrosine-protein phosphatase non-receptor type 6	0.940909091
Alcohol dehydrogenase E chain	0.294444444
Glutamate receptor ionotropic, kainate 1	0.413461538
Fibroblast growth factor receptor 3	0.585714286
Serine/threonine-protein kinase MARK2	0.400793651
Nitric oxide synthase, endothelial	0.419295254
Heat shock protein HSP 90-alpha	0.875
Dihydroorotate dehydrogenase (quinone), mitochondrial	0.699393939
Caspase-3	0.867424242
Proto-oncogene tyrosine-protein kinase Src	0.559242979
Thymidylate synthase	0.637085137
Hepatocyte growth factor receptor	0.657549331
Dual specificity mitogen-activated protein kinase kinase 1	0.426020408
Macrophage-stimulating protein receptor	0.535115304
Inosine-5'-monophosphate dehydrogenase 1	0.398611111
Acetylcholinesterase	0.46
Gamma-aminobutyric acid receptor subunit alpha-5	0.519382022
Serine/threonine-protein kinase LATS2	0.638310185
Coagulation factor X	0.56301066
Mu-type opioid receptor	0.593786352
Proto-oncogene tyrosine-protein kinase Src	0.604957265
Peripheral plasma membrane protein CASK	0.628342246
Synaptic vesicular amine transporter	0.833333333
Estrogen receptor beta	0.718913925
Phosphorylase b kinase gamma catalytic chain, skeletal muscle/heart isoform	0.616185897
Acetylcholinesterase	1

PROTEIN FAMILY	AUC
S-adenosylmethionine decarboxylase proenzyme	0.654545455
Chymase	0.705270458
Kinesin-like protein KIF11	0.836538462
Serine/threonine-protein kinase Chk1	0.640441176
Beta-1,4-galactosyltransferase 1	1
Kappa-type opioid receptor	0.585662688
Acetylcholinesterase	0.46
Aminopeptidase N	0.666666667
Orotidine 5'-phosphate decarboxylase	0.478571429
Glucocorticoid receptor	0.57751938
Adenosylhomocysteinase	0.290348101
Thermolysin	0.510810811
Coagulation factor X	0.537989513
Chymotrypsinogen A	0.755136986
Cyclin-dependent kinase 5	0.46700707
Protein-tyrosine kinase 2-beta	0.599019608
NT-3 growth factor receptor	0.571
Peroxisome proliferator-activated receptor delta	0.727513228
Renin	0.798413826
Carboxypeptidase B2	0.931034483
Mitogen-activated protein kinase 7	0.409356725
Thymidylate synthase	0.330016584
Vascular endothelial growth factor receptor 2	0.579122558
Macrophage migration inhibitory factor	1
Glutathione S-transferase P	0.75
Uridine 5'-monophosphate synthase	0.35
Tyrosine-protein kinase Fyn	0.550501557
Lysophosphatidic acid receptor 3	0.263636364
Serine/threonine-protein kinase mTOR	0.757454819
Aldo-keto reductase family 1 member C3	0.777777778
Thymidylate synthase	0.68387257
Mitogen-activated protein kinase 10	0.477408854
Receptor-interacting serine/threonine-protein kinase 4	0.497857143
Serine/threonine-protein kinase tousled-like 1	0.593990755
Alcohol dehydrogenase E chain	0.291666667
Beta-secretase 1	0.724867335
Nitric oxide synthase, endothelial	0.442313157
Ephrin type-A receptor 4	0.456410256
Acetylcholinesterase	1
cAMP-dependent protein kinase catalytic subunit alpha	0.481055086
Tyrosine-protein kinase JAK1	0.529947917
Casein kinase I isoform delta	0.543219076
Cationic trypsin	0.643
Tyrosine-protein kinase Lck	0.658634137
Adenosine deaminase	0.859053498
Deoxyuridine 5'-triphosphate nucleotidohydrolase, mitochondrial	0.53638814
Tyrosine-protein kinase ZAP-70	0.599353322
cAMP-dependent protein kinase catalytic subunit alpha	0.400029146
Proto-oncogene tyrosine-protein kinase Src	0.627252747
Vascular endothelial growth factor receptor 2	0.647713631
Uncharacterized aarF domain-containing protein kinase 4	0.526785714
Proto-oncogene tyrosine-protein kinase Src	0.493431013
Tyrosine-protein kinase ABL1	0.55962896

PROTEIN FAMILY	AUC
Phenylethanolamine N-methyltransferase	0.716176471
Dihydrofolate reductase	0.382309942
Serine/threonine-protein kinase pim-1	0.710566162
MAP kinase-interacting serine/threonine-protein kinase 2	0.587868481
Tyrosine-protein phosphatase non-receptor type 1	0.65142183
Adenosine kinase	0.578125
3-phosphoshikimate 1-carboxyvinyltransferase	0.6
Heat shock protein HSP 90-alpha	0.827830189
Serine/threonine-protein kinase pim-1	0.679789335
3-phosphoinositide-dependent protein kinase 1	0.557918552
Muscarinic acetylcholine receptor M4	0.588568376
Metabotropic glutamate receptor 3	0.549019608
Proto-oncogene tyrosine-protein kinase receptor Ret	0.566240055
Cationic trypsin	0.647497188
Carbonic anhydrase 2	0.75399571
Prostaglandin G/H synthase 1	0.7
Mitogen-activated protein kinase 10	0.559469697
Nitric oxide synthase, endothelial	0.395282751
Tyrosine-protein kinase Fyn	0.483050847
Tyrosine-protein kinase Lyn	0.66212766
Focal adhesion kinase 1	0.443981481
Vasopressin V2 receptor	0.527426673
Tyrosine-protein kinase BTK	0.474244505
Transthyretin	0.785714286
Histamine H2 receptor	0.676510989
Gamma-aminobutyric acid receptor subunit alpha-3	0.491756736
Neprilysin	0.442043896
Acetylcholinesterase	0.46
Serum albumin	0.576388889
Tyrosine-protein kinase ABL1	0.564463049
Serine/threonine-protein kinase MARK2	0.523015873
Caspase-3	0.85479798
Transient receptor potential cation channel subfamily M member 6	0.822580645
Phosphatidylinositol 4,5-bisphosphate 3-kinase catalytic subunit gamma isoform	0.676005747
Sodium-dependent serotonin transporter	0.642132846
Tyrosine-protein kinase Lck	0.673932607
Ephrin type-A receptor 3	0.588888889
Ephrin type-B receptor 3	0.59141791
Tyrosine-protein kinase ABL1	0.563908877
P2Y purinoceptor 1	0.382591093
Gamma-aminobutyric acid receptor subunit gamma-2	0.569208333
Glutamyl aminopeptidase	0.666666667
Ribosyldihydronicotinamide dehydrogenase [quinone]	0.2
Serine/threonine-protein kinase BRSK1	0.532407407
Serine/threonine-protein kinase 4	0.501572327
Carbonic anhydrase 2	0.772841769
Beta-lactamase	0.199404762
Suppressor of tumorigenicity 14 protein	0.757978723
Angiotensin-converting enzyme	0.540081522
Serine/threonine-protein kinase Chk2	0.667592593
Receptor-type tyrosine-protein phosphatase F	0.863636364
Aminopeptidase N	1
Serine/threonine-protein kinase pim-3	0.771929825

PROTEIN FAMILY	AUC
Scytalone dehydratase	0.616915423
Neutral alpha-glucosidase AB	0.409722222
LIM domain kinase 1	0.484577114
Renin	0.760298295
Eukaryotic translation initiation factor 2-alpha kinase 4	0.337868481
Fibroblast growth factor receptor 4	0.602120536
Glutathione S-transferase P	0.6625
Transthyretin	0.785714286
Disintegrin and metalloproteinase domain-containing protein 17	0.56137411
Peptidyl-prolyl cis-trans isomerase NIMA-interacting 1	0.73953824
Tyrosine-protein phosphatase non-receptor type 1	0.673653245
3-phosphoinositide-dependent protein kinase 1	0.366063348
Serine/threonine-protein kinase MRCK gamma	0.718027735
Acetylcholinesterase	0.446666667
Thyroid hormone receptor beta	0.173534799
cGMP-dependent protein kinase 1	0.663978495
Coagulation factor X	0.546251586
MAP kinase-activated protein kinase 2	0.588484848
Cationic trypsin	0.671201814
cAMP-dependent protein kinase catalytic subunit beta	0.524482109
Transthyretin	0.928571429
Coagulation factor X	0.540910679
Kinesin-like protein KIF11	0.774038462
Ectonucleotide pyrophosphatase/phosphodiesterase family member 2	0.397457627
Baculoviral IAP repeat-containing protein 2	nan
Ephrin type-B receptor 1	0.528258362
Thymidylate synthase	0.587301587
Carbonic anhydrase 2	0.761839645
Retinoic acid receptor RXR-alpha	0.601554908
Solute carrier family 22 member 6	0.777777778
Bromodomain-containing protein 4	0.375
Casein kinase I isoform gamma-2	0.452586207
Beta-1,4-galactosyltransferase 1	1
Dihydrofolate reductase	0.538011696
Aldose reductase	0.444444444
Carbonic anhydrase 1	0.707165876
Thymidylate synthase	0.673974741
cAMP-specific 3',5'-cyclic phosphodiesterase 4D	0.689199689
Nitric oxide synthase, inducible	0.633064516
Orotidine 5'-phosphate decarboxylase	0.45
High affinity nerve growth factor receptor	0.536831812
Fatty-acid amide hydrolase 1	0.560840385
Cyclin-dependent kinase 2	0.498325984
Hepatocyte growth factor receptor	0.569840416
Ribosomal protein S6 kinase alpha-5	0.564925373
Prostaglandin G/H synthase 1	0.5
Angiotensin-converting enzyme	0.460258152
Mast/stem cell growth factor receptor Kit	0.58230265
cAMP-specific 3',5'-cyclic phosphodiesterase 4D	0.742683243
Serine/threonine-protein kinase ULK2	0.543218085
Protein S100-B	0.166666667
5-hydroxytryptamine receptor 2C	0.571060038
Serine/threonine-protein kinase Chk2	0.588425926

PROTEIN FAMILY	AUC
Glutamate receptor ionotropic, NMDA 2B	0.452380952
Fibroblast growth factor receptor 3	0.469758065
Alcohol dehydrogenase E chain	0.261111111
Thymidylate synthase	0.624819625
Renin	0.775804924
Phosphorylase b kinase gamma catalytic chain, skeletal muscle/heart isoform	0.643830128
Thymidylate synthase	0.583333333
Bacterial leucyl aminopeptidase	0.272727273
DNA polymerase alpha catalytic subunit	0.401960784
Orotidine 5'-phosphate decarboxylase	0.457142857
Acidic mammalian chitinase	1
Tyrosine-protein phosphatase non-receptor type 11	0.615384615
Orotidine 5'-phosphate decarboxylase	0.485714286
Calcium/calmodulin-dependent protein kinase type 1G	0.474137931
Cathepsin S	0.691760978
Beta-secretase 1	0.832399764
Heat shock protein HSP 90-alpha	0.872641509
Coagulation factor V	0.5625
Cationic trypsin	0.66274189
Calcium/calmodulin-dependent protein kinase type II subunit gamma	0.527836134
Retinoic acid receptor alpha	0.624605263
Lethal(3)malignant brain tumor-like protein 1	0.333333333
Transthyretin	0.75
Leucine-rich repeat serine/threonine-protein kinase 2	0.470781157
Estrogen receptor beta	0.731670243
TGF-beta receptor type-1	0.647089397
C-X-C chemokine receptor type 1	0.792553191
Coagulation factor VII	0.744583582
Fatty acid-binding protein, adipocyte	0.868421053
Heat shock protein HSP 90-alpha	0.872641509
Prothrombin	0.458054175
Serine/threonine-protein kinase Chk1	0.625735294
Peroxisome proliferator-activated receptor gamma	0.759288194
Gonadotropin-releasing hormone receptor	0.533816425
Dual specificity protein kinase CLK1	0.473391813
Prostaglandin E2 receptor EP3 subtype	0.675795251
Serine/threonine-protein kinase 17A	0.518939394
U4/U6 small nuclear ribonucleoprotein Prp4	0.666666667
Sialidase-2	0.833333333
Vascular endothelial growth factor receptor 2	0.650169159
Progesterone receptor	0.575510942
Adenosine kinase	0.524305556
Homeodomain-interacting protein kinase 3	0.475897436
Glutamate receptor ionotropic, kainate 1	0.208653846
Tyrosine-protein kinase Fes/Fps	0.468487395
Mast/stem cell growth factor receptor Kit	0.677749781
Nitric oxide synthase, endothelial	0.385763001
Purine nucleoside phosphorylase	0.543851351
Mitogen-activated protein kinase kinase kinase kinase 1	0.548664944
Beta-lactamase	0.212962963
Peptidyl-prolyl cis-trans isomerase A	0.926289926
Plasmepsin-2	0.833917971
Stromelysin-1	0.4263828

PROTEIN FAMILY	AUC
Serine/threonine-protein kinase Nek2	0.511538462
Angiotensin-converting enzyme	0.561820652
Glutathione S-transferase P	0.675
Serine/threonine-protein kinase MRCK beta	0.447916667
Alcohol dehydrogenase 4	0.6875
Integrin alpha-IIb	0.439716312
Tyrosine-protein kinase ABL1	0.540676261
Carbonic anhydrase 2	0.764590441
Lanosterol 14-alpha demethylase	0.833333333
Beta-1,4-galactosyltransferase 1	0.46875
Tyrosine-protein phosphatase non-receptor type 1	0.730097444
Cyclin-dependent kinase 2	0.359648048
Mitogen-activated protein kinase 14	0.703218199
Tyrosine-protein kinase Lck	0.660633937
Urokinase-type plasminogen activator	0.53729832
Caspase-1	0.727387274
Serine/threonine-protein kinase SIK3	0.524509804
Beta-lactamase	0.413333333
Androgen receptor	0.623240297
Dipeptidyl peptidase 2	0.444646098
Coagulation factor X	0.57539107
Growth factor receptor-bound protein 2	0.606060606
Estrogen receptor	0.686146411
Prostaglandin G/H synthase 2	0.975609756
Peptidyl-prolyl cis-trans isomerase NIMA-interacting 1	0.744348244
Adenosine deaminase	0.916666667
Nitric oxide synthase, endothelial	0.395424837
Glycogen synthase kinase-3 beta	0.50366867
STE20-like serine/threonine-protein kinase	0.53745541
Epidermal growth factor receptor	0.736980932
Somatostatin receptor type 1	0.522970085
Mitogen-activated protein kinase 14	0.715875988
Carbonic anhydrase 2	0.767170411
Cationic trypsin	0.643212863
Acetylcholinesterase	1
Mitogen-activated protein kinase 10	0.56737013
Cationic trypsin	0.659178571
Substance-K receptor	0.48099158
Neutrophil collagenase	0.653854656
Cyclin-dependent kinase 2	0.376674016
Vasopressin V2 receptor	0.308491516
Glucocorticoid receptor	0.534079816
Stromelysin-1	0.405162765
Tyrosine-protein kinase ABL1	0.508512238
Cationic trypsin	0.659291406
Peroxisome proliferator-activated receptor gamma	0.692274306
Aldose reductase	0.5
Nitric oxide synthase, inducible	0.638986895
Growth factor receptor-bound protein 2	0.556818182
Tyrosine-protein kinase ABL1	0.537854244
Neutrophil collagenase	0.603813208
Serine/threonine-protein kinase SIK3	0.645424837
Peptidyl-prolyl cis-trans isomerase FKBP1A	0.732279171

PROTEIN FAMILY	AUC
Serine/threonine-protein kinase MRCK alpha	0.672715054
Glycogen synthase kinase-3 beta	0.541394095
Serine/threonine-protein kinase 33	0.427083333
3-phosphoinositide-dependent protein kinase 1	0.381900452
Galectin-9	0.925
Serine/threonine-protein kinase pim-1	0.696872943
Tyrosine-protein kinase Fes/Fps	0.422994652
Adenosine kinase	0.592881944
Peroxisome proliferator-activated receptor gamma	0.715711806
Cyclin-dependent kinase-like 5	0.606603774
Matrix metalloproteinase-9	0.302974304
Angiotensin-converting enzyme	0.558763587
Queuine tRNA-ribosyltransferase	0.742857143
Orotidine 5'-phosphate decarboxylase	0.535714286
Serine/threonine-protein kinase Chk1	0.616169154
Sodium- and chloride-dependent glycine transporter 1	0.96969697
Phenylethanolamine N-methyltransferase	0.762683824
S-methyl-5'-thioadenosine phosphorylase	0.224358974
Phosphatidylinositol 4,5-bisphosphate 3-kinase catalytic subunit gamma isoform	0.624281609
Renin	0.717211174
Gamma-aminobutyric acid receptor subunit gamma-2	0.540641026
Thermolysin	0.562222222
Beta-lactamase	0.236467236
Peroxisome proliferator-activated receptor gamma	0.761631944
Tyrosine-protein kinase JAK2	0.597472075
Serine/threonine-protein kinase Nek9	0.592741935
Dihydrofolate reductase	0.399122807
Myosin light chain kinase, smooth muscle	0.438720539
Serine/threonine-protein kinase SIK3	0.615196078
Cationic trypsin	0.659459266
Coagulation factor X	0.538563831
Prokineticin receptor 2	0.5
Receptor tyrosine-protein kinase erbB-3	0.679861111
Gamma-aminobutyric acid receptor subunit gamma-1	0.333333333
Nuclear receptor subfamily 1 group I member 2	0.25
Acetylcholinesterase	1
Thermolysin	0.682222222
Serine/threonine-protein kinase Chk1	0.604901961
Peroxisome proliferator-activated receptor alpha	0.323461092
Peroxisome proliferator-activated receptor gamma	0.743489583
Mitogen-activated protein kinase kinase kinase 4	0.465909091
Purine nucleoside phosphorylase	0.291666667
Myosin-IIIa	0.550179211
Amine oxidase [flavin-containing] B	0.630754258
Prothrombin	0.421257669
Maltase-glucoamylase, intestinal	0.466666667
Growth factor receptor-bound protein 2	0.401515152
Serine/threonine-protein kinase OSR1	nan
Lanosterol 14-alpha demethylase	0.0625
Serine/threonine-protein kinase Chk1	0.639705882
S-adenosylmethionine decarboxylase proenzyme	0.609090909
Ephrin type-B receptor 4	0.593227425
Aldose reductase	0.555555556

PROTEIN FAMILY	AUC
Cathepsin K	0.503772923
Cathepsin D	0.667235772
Cationic trypsin	0.661923734
Aurora kinase A	0.535950551
Chymase	0.935506241
Tyrosine-protein kinase SYK	0.331853496
ALK tyrosine kinase receptor	0.568892045
cGMP-dependent 3',5'-cyclic phosphodiesterase	0
Mitogen-activated protein kinase 9	0.500198413
Cathepsin S	0.654640109
MAP kinase-interacting serine/threonine-protein kinase 1	0.520576132
Tyrosine-protein kinase Lck	0.639286071
Coagulation factor VII	0.717451799
Angiotensin-converting enzyme	0.542459239
Hypoxanthine-guanine phosphoribosyltransferase	0.509698276
Rho-associated protein kinase 1	0.402027027
Tyrosine-protein phosphatase non-receptor type 1	0.647698719
Seminal ribonuclease	0.333333333
Dipeptidyl peptidase 4	0.684590929
Dihydrofolate reductase	0.57097245
Protein-tyrosine kinase 2-beta	0.609803922
Angiotensin-converting enzyme	0.539402174
Protein kinase C iota type	0.538709677
Tyrosine-protein kinase Mer	0.625615764
Gonadotropin-releasing hormone receptor	0.472333705
Dihydrofolate reductase	0.527158584
Dihydrofolate reductase	0.69861705
Hepatocyte growth factor receptor	0.630329457
Protein kinase C theta type	0.312348668
M1 family aminopeptidase	0
cAMP-dependent protein kinase catalytic subunit alpha	0.489944623
Cytidine deaminase	0.695238095
Fatty acid-binding protein, adipocyte	0.865131579
Protein kinase C delta type	0.359806224
Macrophage metalloelastase	0.402704832
Orotidine 5'-phosphate decarboxylase	0.521428571
Aldose reductase	0.347222222
Nitric oxide synthase, inducible	0.636844758
Aldose reductase	0.416666667
Orotidine 5'-phosphate decarboxylase	0.464285714
Metallo-beta-lactamase L1	0.884920635
Excitatory amino acid transporter 1	0.923076923
Oxytocin receptor	0.42294686
Glycogen synthase kinase-3 beta	0.595120226
Serine/threonine-protein kinase Nek2	0.522692308
Tyrosine-protein kinase Lck	0.681281872
5-hydroxytryptamine receptor 4	0.639208174
Cathepsin K	0.475853295
Endothelin-1 receptor	0.383031815
Cyclin-dependent kinase 2	0.557161522
Cationic trypsin	0.653882302
Thyroid hormone receptor alpha	0.05
Peroxisome proliferator-activated receptor gamma	0.753125

PROTEIN FAMILY	AUC
Catechol O-methyltransferase	0.133333333
Nischarin	0.790123457
Serine/threonine-protein kinase PAK 2	0.565047022
cAMP-specific 3',5'-cyclic phosphodiesterase 4D	0.663170163
Beta-1,4-galactosyltransferase 1	0.40625
Peptidyl-prolyl cis-trans isomerase A	0.907094595
Carbonic anhydrase 2	0.757473334
Neuropeptide Y receptor type 5	0.701367488
Mandelate racemase	0.369047619
Carboxypeptidase B2	0.603448276
cAMP-specific 3',5'-cyclic phosphodiesterase 4A	0.731829574
5-hydroxytryptamine receptor 1A	0.565746927
Casein kinase II subunit alpha	0.451509872
Ribonuclease pancreatic	0.357142857
NT-3 growth factor receptor	0.5745
Prothrombin	0.474925771
Epidermal growth factor receptor	0.727008547
Tyrosine-protein phosphatase non-receptor type 1	0.706425814
Lactoylglutathione lyase	0.68519656
Transthyretin	0.75
Glutamate carboxypeptidase 2	0.54004329
Prothrombin	0.43981157
Dihydroorotate dehydrogenase (quinone), mitochondrial	0.677954545
ATP-sensitive inward rectifier potassium channel 11	0.34375
Acetylcholinesterase	1
Serine/threonine-protein kinase Chk1	0.615931373
Acetylcholinesterase	1
Pteridine reductase 1	0.677489177
Urokinase-type plasminogen activator	0.526704039
Leukotriene A-4 hydrolase	1
Integrin alpha-L	0.43495935
Egl nine homolog 1	0.4
Dihydrofolate reductase	0.5
Metabotropic glutamate receptor 3	0.450980392
Inosine-5'-monophosphate dehydrogenase 1	0.630555556
Focal adhesion kinase 1	0.559259259
Cationic trypsin	0.664186508
AP2-associated protein kinase 1	0.495419309
Suppressor of tumorigenicity 14 protein	0.362421384
Cyclin-dependent kinase 2	0.369998367
Glutamate carboxypeptidase 2	0.707792208
Myelin-associated glycoprotein	1
Calcium/calmodulin-dependent protein kinase kinase 2	0.500347222
Serine/threonine-protein kinase pim-1	0.714285714
Serine/threonine-protein kinase 4	0.477163462
Tyrosine-protein kinase Fyn	0.51937046
Angiotensin-converting enzyme	0.575407609
Caspase-1	0.729907299
S-adenosylmethionine decarboxylase proenzyme	0.509090909
Cruzipain	0.944444444
Citron Rho-interacting kinase	0.603448276
Deoxyuridine 5'-triphosphate nucleotidohydrolase, mitochondrial	0.65902965
Steroid hormone receptor ERR1	1

PROTEIN FAMILY	AUC
Sodium channel protein type 9 subunit alpha	0.438223938
Sodium- and chloride-dependent glycine transporter 1	0.575757576
Beta-2 adrenergic receptor	0.649256422
Cyclin-dependent kinase 2	0.357504491
Cathepsin K	0.529762218
Transthyretin	0.464285714
Glutathione reductase, mitochondrial	0.688888889
Leukotriene A-4 hydrolase	1
Cationic trypsin	0.624710816
Prothrombin	0.438480428
Dipeptidyl peptidase 4	0.697854056
MAP kinase-interacting serine/threonine-protein kinase 2	0.4925
Acidic mammalian chitinase	1
Cathepsin B	0.652318052
Matrilysin	0.302967172
Dehydrosqualene synthase	0.819444444
Misshapen-like kinase 1	0.539272031
Substance-K receptor	0.512630388
Serine/threonine-protein kinase tousled-like 1	0.61633282
cAMP-dependent protein kinase catalytic subunit alpha	0.439522005
cAMP-specific 3',5'-cyclic phosphodiesterase 4B	0.838996764
Beta-secretase 1	0.792526533
Cationic trypsin	0.643494898
Beta-lactamase OXA-10	1
Serine/threonine-protein kinase B-raf	0.642431973
Ribonuclease pancreatic	0.468944099
Tankyrase-2	0.833333333
Mitogen-activated protein kinase kinase kinase kinase 1	0.639119601
cGMP-inhibited 3',5'-cyclic phosphodiesterase B	0.64
Fatty acid-binding protein, intestinal	0
Peptidyl-prolyl cis-trans isomerase A	0.894144144
cAMP-dependent protein kinase catalytic subunit alpha	0.398426115
Mitogen-activated protein kinase kinase kinase 12	0.542307692
Cyclin-dependent kinase 1	0.696517413
Glycogen phosphorylase, muscle form	0.428571429
3-phosphoinositide-dependent protein kinase 1	0.353400735
Macrophage metalloelastase	0.469931723
Orotidine 5'-phosphate decarboxylase	0.457142857
Amyloid beta A4 protein	0.623891938
5-hydroxytryptamine receptor 2C	0.360791119
Serine/threonine-protein kinase RIO1	0.501633987
Glucosylceramidase	0.768327068
Tyrosine-protein kinase JAK2	0.638145083
Neutrophil collagenase	0.655927052
Prothrombin	0.528732341
Tyrosine-protein kinase receptor UFO	0.46796875
Ectonucleoside triphosphate diphosphohydrolase 2	0.55
MAP/microtubule affinity-regulating kinase 4	0.52354049
Fibroblast growth factor 1	0.538461538
Fibroblast growth factor receptor 2	0.501201923
Stromelysin-1	0.424354071
Purine nucleoside phosphorylase	0.398809524
Growth factor receptor-bound protein 2	0.545454545

PROTEIN FAMILY	AUC
Receptor-type tyrosine-protein phosphatase beta	0.725
Peroxisome proliferator-activated receptor gamma	0.814149306
Protein kinase C iota type	0.583870968
Carbonic anhydrase 2	0.761741175
cAMP-specific 3',5'-cyclic phosphodiesterase 4D	0.552173913
Coagulation factor X	0.53374337
Urokinase-type plasminogen activator	0.535115199
Angiopoietin-1 receptor	0.657169118
Calcitonin receptor	
Phosphatidylinositol 4,5-bisphosphate 3-kinase catalytic subunit gamma isoform	0.656609195
Neutrophil collagenase	0.640950539
Muscarinic acetylcholine receptor M4	0.461538462
Thermolysin	0.563783784
Thyrotropin-releasing hormone receptor	0.055555556
Serine/threonine-protein kinase tousled-like 2	0.527306968
RAC-alpha serine/threonine-protein kinase	0.618667518
Casein kinase II subunit alpha	0.432830043
Cyclin-dependent kinase 9	0.354042806
Carbonic anhydrase 2	0.765689266
Serum albumin	0.423611111
5-hydroxytryptamine receptor 2A	0.640705573
Mitogen-activated protein kinase 14	0.665712998
Glycogen synthase kinase-3 beta	0.57032355
Ribonuclease pancreatic	0.409937888
Myosin-IIIa	0.504553734
Thymidylate synthase	0.537313433
Tyrosine-protein kinase Lck	0.617338266
cAMP-specific 3',5'-cyclic phosphodiesterase 4B	0.887001079
Cationic trypsin	0.666122242
Muscarinic acetylcholine receptor M1	0.576135442
cAMP-dependent protein kinase catalytic subunit beta	0.667608286
Estrogen receptor	0.632181851
Tyrosine-protein phosphatase non-receptor type 1	0.714193786
Dipeptidyl peptidase 1	0.166666667
Testis-specific serine/threonine-protein kinase 1	0.437788018
Aldose reductase	0.472222222
Phosphatidylinositol 4,5-bisphosphate 3-kinase catalytic subunit gamma isoform	0.590517241
Progesterone receptor	0.529101103
Dual specificity protein kinase TTK	0.477910238
Tyrosine-protein kinase CSK	0.507026627
Delta-type opioid receptor	0.54298799
Chymotrypsinogen A	0.669125395
Methionine aminopeptidase 1	0.768115942
Estrogen receptor	0.622852157
Prothrombin	0.614246522
Estrogen receptor	0.717748792
Maltase-glucoamylase, intestinal	0.166666667
Adenosine deaminase	0.797325103
Kinesin-like protein KIF11	0.798076923
Cationic trypsin	0.649280068
Nuclear receptor corepressor 2	0.5
Peptidyl-prolyl cis-trans isomerase FKBP1A	0.741131567
Dual specificity protein kinase CLK3	0.434387352

PROTEIN FAMILY	AUC
Chaperone activity of bc1 complex-like, mitochondrial	0.550847458
Epidermal growth factor receptor	0.712457627
cAMP-specific 3',5'-cyclic phosphodiesterase 4B	0.868662352
Glycogen synthase kinase-3 beta	0.580953854
Tyrosine-protein kinase Lck	0.65008058
Mitogen-activated protein kinase 14	0.563809127
Abelson tyrosine-protein kinase 2	0.388321995
Estradiol 17-beta-dehydrogenase 1	0.5
Neuropeptide Y receptor type 5	0.684899846
Microtubule-associated serine/threonine-protein kinase 1	0.400205761
Phenylethanolamine N-methyltransferase	0.762591912
Aminopeptidase N	0.498272196
Myosin light chain kinase 2, skeletal/cardiac muscle	0.581128748
Dual specificity mitogen-activated protein kinase kinase 4	0.40408805
Proto-oncogene tyrosine-protein kinase ROS	0.476817043
cAMP-specific 3',5'-cyclic phosphodiesterase 4B	0.857874865
Serum albumin	0.465277778
cAMP-specific 3',5'-cyclic phosphodiesterase 4B	0.887001079
D(1A) dopamine receptor	0.590815688
Receptor-type tyrosine-protein phosphatase beta	0.6375
Carnitine O-acetyltransferase	0.222222222
Interleukin-1 receptor-associated kinase 4	0.738552855
Phosphatidylinositol 4-kinase beta	0.322511536
Retinoic acid receptor alpha	0.481052632
Glucosylceramidase	0.745300752
Cathepsin K	0.52777924
Fibroblast growth factor receptor 3	0.559435626
Farnesyl pyrophosphate synthase	0.599305556
Glycogen synthase kinase-3 alpha	0.465872156
Ribonuclease pancreatic	0.313664596
Beta-lactamase TEM	0.477058824
Amine oxidase [flavin-containing] B	0.641557178
Dual specificity mitogen-activated protein kinase kinase 5	0.592592593
Methionine aminopeptidase 2	0.488313008
Receptor-type tyrosine-protein phosphatase beta	0.6625
Carbonic anhydrase 13	0.77027027
Protein kinase C eta type	0.421670117
Beta-1,4-galactosyltransferase 1	0.5
Ephrin type-A receptor 8	0.5513573
Serine/threonine-protein kinase mTOR	0.73621988
Trypsin	0.641666667
Estrogen receptor beta	0.595433953
Nitric oxide synthase, inducible	0.62953629
Thymidylate synthase	0.666666667
Prothrombin	0.438138474
Mu-type opioid receptor	0.544555033
Aminopeptidase N	1
Angiotensin-converting enzyme	0.519021739
Serine/threonine-protein kinase Chk1	0.686764706
Stromelysin-1	0.420279125
Coagulation factor V	0.875
Phenylethanolamine N-methyltransferase	0.742738971
Nitric oxide synthase, endothelial	0.377522023

PROTEIN FAMILY	AUC
Peroxisome proliferator-activated receptor gamma	0.783767361
Cationic trypsin	0.643505184
Cationic trypsin	0.606929425
Nitric oxide synthase, endothelial	0.425973288
Peroxisome proliferator-activated receptor alpha	0.268292683
Peroxisome proliferator-activated receptor delta	0.783068783
Queuine tRNA-ribosyltransferase	0.714285714
Nitric oxide synthase, endothelial	0.379511225
Mitogen-activated protein kinase kinase kinase 19	0.563829787
Serum albumin	0.479166667
5-hydroxytryptamine receptor 1B	0.485721142
Serine/threonine-protein kinase pim-1	0.699934167
Queuine tRNA-ribosyltransferase	0.685714286
Dipeptidyl peptidase 4	0.693507526
Growth factor receptor-bound protein 2	0.492424242
Beta-lactamase	0.233727811
Papain	0.49798007
Beta-secretase 1	0.811910377
Macrophage metalloelastase	0.359243697
Mitogen-activated protein kinase 10	0.559361472
Aurora kinase A	0.510373984
Nitric oxide synthase, inducible	0.611706149
MAP kinase-interacting serine/threonine-protein kinase 1	0.522119342
Dipeptidyl peptidase 4	0.694202971
Multidrug resistance-associated protein 1	0.28125
Abelson tyrosine-protein kinase 2	0.54223356
Estrogen receptor beta	0.740581784
Muscarinic acetylcholine receptor M3	0.551240913
Queuine tRNA-ribosyltransferase	0.7
Cationic trypsin	0.611314403
NUAK family SNF1-like kinase 2	0.532828283
Chymotrypsinogen A	0.711472603
Egl nine homolog 1	0.454545455
Prothrombin	0.446910847
Thermolysin	0.630810811
Estrogen receptor	0.691873993
S-ribosylhomocysteine lyase	0.815934066
Mandelate racemase	0.351190476
Peroxisome proliferator-activated receptor gamma	0.777951389
5'-AMP-activated protein kinase catalytic subunit alpha-1	0.566818182
Kinesin-like protein KIF11	0.163461538
TRAF2 and NCK-interacting protein kinase	0.593253968
Methionine aminopeptidase 1	0.572463768
Alpha-2A adrenergic receptor	0.33623972
Serine/threonine-protein kinase Chk1	0.625245098
Peroxisome proliferator-activated receptor delta	0.642857143
Beta-lactamase	0.225641026
Cathepsin L1	0.609129047
Serine/threonine-protein kinase PLK1	0.437738732
Interleukin-1 receptor-associated kinase 1	0.323361823
Carboxypeptidase N catalytic chain	0
Mitogen-activated protein kinase 14	0.707934526
Nitric oxide synthase, endothelial	0.409065075

PROTEIN FAMILY	AUC
Acetylcholinesterase	0.446666667
Tryptase alpha/beta-1	0.218917112
3-phosphoinositide-dependent protein kinase 1	0.433936652
Mast/stem cell growth factor receptor Kit	0.632285276
Thyroid hormone receptor beta	0.170787546
Leucine-rich repeat serine/threonine-protein kinase 2	0.399475524
Tyrosine-protein phosphatase non-receptor type 1	0.709459459
Dipeptidyl peptidase 4	0.719810243
Aldose reductase	0.722222222
Amine oxidase [flavin-containing] A	0.601326816
Fibroblast growth factor receptor 2	0.554086538
Mitogen-activated protein kinase 14	0.689242613
Muscarinic acetylcholine receptor M4	0.45357906
Melanocortin receptor 3	0.504160799
fMet-Leu-Phe receptor	1
Alcohol dehydrogenase E chain	0.280555556
Transthyretin	0.892857143
LIM domain kinase 1	0.636318408
Beta-lactamase OXA-10	1
Phosphatidylinositol 4,5-bisphosphate 3-kinase catalytic subunit gamma isoform	0.645833333
Matrix metalloproteinase-15	0.792307692
cGMP-dependent protein kinase 1	0.63844086
Mitogen-activated protein kinase 9	0.597580016
Dihydrofolate reductase	0.622145392
Lysozyme	0.731481481
Nitric oxide synthase, inducible	0.643554688
Proto-oncogene tyrosine-protein kinase Src	0.496068376
Carbonic anhydrase 2	0.762030214
Peroxisomal N(1)-acetyl-spermine/spermidine oxidase	1
Calpain small subunit 1	0.50625
Amine oxidase [flavin-containing] B	0.615523114
Phosphatidylinositol 4-kinase beta	0.418211382
Nitric oxide synthase, endothelial	0.438192668
Thermolysin	0.560810811
Amine oxidase [flavin-containing] A	0.626117318
Cholinesterase	0.82242124
S-adenosylmethionine decarboxylase proenzyme	0.581818182
Amine oxidase [flavin-containing] B	0.63323601
Mitogen-activated protein kinase 10	0.549404762
Dipeptidyl peptidase 4	0.706969351
Proto-oncogene tyrosine-protein kinase receptor Ret	0.618989969
Receptor tyrosine-protein kinase erbB-4	0.3265
Gamma-aminobutyric acid receptor subunit alpha-5	0.577648475
Queuine tRNA-ribosyltransferase	0.657142857
Tyrosine-protein phosphatase non-receptor type 1	0.616550224
Metabotropic glutamate receptor 3	0.490196078
Sigma non-opioid intracellular receptor 1	0.506408983
cAMP-dependent protein kinase catalytic subunit alpha	0.439522005
M-phase inducer phosphatase 1	0
Cathepsin K	0.484934632
Cellular retinoic acid-binding protein 1	0.375
Protein kinase C beta type	0.454545455
Bacterial leucyl aminopeptidase	0.212121212

PROTEIN FAMILY	AUC
Dipeptidyl peptidase 4	0.714246684
Serine/threonine-protein kinase TAO3	0.488407258
Calcium/calmodulin-dependent protein kinase type 1D	0.449292453
Coagulation factor X	0.552311356
Serum albumin	0.354166667
Tyrosine-protein phosphatase non-receptor type 1	0.599972421
Glutathione S-transferase A1	0.7
Rho-associated protein kinase 1	0.361833333
Poly [ADP-ribose] polymerase 1	0.44503012
Serine/threonine-protein kinase pim-1	0.714549045
Leukotriene A-4 hydrolase	1
Calpain small subunit 1	0.5
MAP/microtubule affinity-regulating kinase 3	0.672781065
Cathepsin E	0.166666667
Glycogen synthase kinase-3 beta	0.59114215
Glutathione S-transferase A1	0.533333333
Prostasin	0.847826087
Queuine tRNA-ribosyltransferase	0.642857143
Cellular retinoic acid-binding protein 1	0.5
Multidrug resistance-associated protein 1	0.40625
Cathepsin S	0.702557718
Serine/threonine-protein kinase Chk2	0.635648148
Fibroblast growth factor 1	0.572649573
Phosphatidylinositol 4,5-bisphosphate 3-kinase catalytic subunit gamma isoform	0.646551724
Serine/threonine-protein kinase Chk2	0.623352166
Beta-1,4-galactosyltransferase 1	0.5
Peroxisome proliferator-activated receptor delta	0.592592593
Beta-lactamase TEM	0.475882353
Receptor tyrosine-protein kinase erbB-4	0.440816327
Phenylethanolamine N-methyltransferase	0.730330882
Adenosine receptor A2a	0.3599814
Tyrosine-protein phosphatase non-receptor type 1	0.709842496
Alcohol dehydrogenase 1A	0.673076923
Coagulation factor IX	0.35761079
Epidermal growth factor receptor	0.723294492
Excitatory amino acid transporter 2	0.413461538
Ribonuclease pancreatic	0.319875776
Carbonic anhydrase 2	0.766979329
BDNF/NT-3 growth factors receptor	0.621186441
S-adenosylmethionine decarboxylase proenzyme	0.536363636
3-phosphoinositide-dependent protein kinase 1	0.313122172
Nuclear receptor corepressor 2	0.833333333
Nitric oxide synthase, endothelial	0.393009378
Integrin alpha-L	0.835365854
Arginase-1	0.271428571
Estradiol 17-beta-dehydrogenase 1	0.666666667
Carbonic anhydrase 4	0.825150221
Cathepsin K	0.521575853
Queuine tRNA-ribosyltransferase	0.671428571
Adenosine deaminase	0.773662551
Dipeptidyl peptidase 4	0.720331827
Fibroblast growth factor receptor 2	0.545138889
Death-associated protein kinase 2	0.542542017

PROTEIN FAMILY	AUC
Ribosomal protein S6 kinase alpha-1	0.543650794
Serine/threonine-protein kinase Chk1	0.634068627
C-X-C chemokine receptor type 3	0.949579832
Retinoic acid receptor RXR-alpha	0.590500486
Beta-secretase 1	0.671506486
Chaperone activity of bc1 complex-like, mitochondrial	0.389830508
Dihydroorotate dehydrogenase (quinone), mitochondrial	0.667651515
Beta-lactamase	0.23325062
Purine nucleoside phosphorylase	0.593581081
Caspase-1	0.694854449
Kinesin-like protein KIF11	0.105769231
Tyrosine-protein kinase SYK	0.35794367
Prostaglandin F2-alpha receptor	0.587037037
Phosphatidylinositol 4,5-bisphosphate 3-kinase catalytic subunit gamma isoform	0.622844828
3-phosphoinositide-dependent protein kinase 1	0.381900452
Casein kinase I isoform gamma-3	0.435736677
Coagulation factor X	0.544514541
ALK tyrosine kinase receptor	0.59457478
Tyrosine-protein kinase Lck	0.680581942
Prostasin	0.891304348
Acetylcholinesterase	1
MAP kinase-activated protein kinase 2	0.723636364
Dipeptidyl peptidase 4	0.696488004
Prothrombin	0.408837399
MAP kinase-activated protein kinase 2	0.715151515
Proto-oncogene tyrosine-protein kinase Src	0.515555556
Arginase-1	0.214285714
Pro-cathepsin H	0.45
cAMP-dependent protein kinase catalytic subunit alpha	0.430486739
High affinity nerve growth factor receptor	0.624745763
Leukotriene A-4 hydrolase	1
Glutathione S-transferase A1	0.683333333
cAMP-dependent protein kinase catalytic subunit alpha	0.701981929
Bcl-2-like protein 2	0.45
Corticosteroid 11-beta-dehydrogenase isozyme 1	0.59040959
Phosphatidylinositol 4-kinase beta	0.366018457
Glutamate receptor ionotropic, kainate 1	0.286057692
Corticosteroid 11-beta-dehydrogenase isozyme 1	0.644605395
Microtubule-associated serine/threonine-protein kinase 1	0.438383838
Cathepsin S	0.704391127
Protein kinase C theta type	0.466954023
High affinity nerve growth factor receptor	0.533898305
Mitogen-activated protein kinase kinase kinase 4	0.463181818
Arginase-1	0.328571429
Serum albumin	0.340277778
Amyloid beta A4 protein	0.496623048
Thymidylate synthase	0.538239538
Cruzipain	0.988888889
Cyclin-dependent kinase 2	0.476522946
Protein-tyrosine kinase 2-beta	0.608333333
Heparanase	0.1
Dihydrofolate reductase	0.329678363
Integrin alpha-IIb	0.432411674

PROTEIN FAMILY	AUC
Cyclin-dependent kinase 2	0.365813327
Lysophosphatidic acid receptor 3	0.236363636
Lactoylglutathione lyase	0.694410319
Acetylcholinesterase	1
Mitogen-activated protein kinase 14	0.67765987
Hepatocyte growth factor receptor	0.632751938
Peptidyl-prolyl cis-trans isomerase FKBP1A	0.79977869
Urokinase-type plasminogen activator	0.539499606
Beta-secretase 1	0.79739092
Queuine tRNA-ribosyltransferase	0.657142857
Calpain small subunit 1	0.475
Beta-secretase 1	0.808225236
Beta-2 adrenergic receptor	0.616493916
Thymidylate synthase	0.581260365
Focal adhesion kinase 1	0.539351852
Renin	0.773792614
Cathepsin B	0.616573034
Amiloride-sensitive amine oxidase [copper-containing]	0.222222222
Androgen receptor	0.433082413
Integrin alpha-L	0.756097561
Mitogen-activated protein kinase 3	0.484444444
Macrophage migration inhibitory factor	0.9375
Serine/threonine-protein kinase SIK3	0.573529412
Activin receptor type-2A	0.408163265
Aldose reductase	0.458333333
Nitric oxide synthase, endothelial	0.403097471
Amine oxidase [flavin-containing] B	0.604184915
Purine nucleoside phosphorylase	0.563851351
Tyrosine-protein kinase Mer	0.614121511
Heat shock protein HSP 90-alpha	0.880896226
Multidrug resistance-associated protein 1	0.15625
Insulin-like growth factor-binding protein 3	0.964102564
Acetylcholinesterase	1
Thymidylate synthase	0.668334043
Vascular endothelial growth factor receptor 2	0.562315835
Prostasin	0.858695652
Carbonic anhydrase 2	0.761281724
Coagulation factor VII	0.824587557
Somatostatin receptor type 2	0.415256538
Serine/threonine-protein kinase MRCK alpha	0.704918033
Serine/threonine-protein kinase mTOR	0.71121988
Cyclin-dependent kinase-like 1	0.522222222
Serine/threonine-protein kinase B-raf	0.67485119
Serine/threonine-protein kinase MRCK alpha	0.730191257
Prostaglandin E2 receptor EP1 subtype	0.666136893
Mitogen-activated protein kinase 1	0.646539162
Prothrombin	0.439724764
Mitogen-activated protein kinase 9	0.550793651
Serine/threonine-protein kinase Chk1	0.675963489
Queuine tRNA-ribosyltransferase	0.671428571
Mitogen-activated protein kinase 11	0.691854508
Serine/threonine-protein kinase PLK1	0.406417112
Nuclear receptor subfamily 1 group I member 2	0.5

PROTEIN FAMILY	AUC
Acetylcholinesterase	0.406666667
Queuine tRNA-ribosyltransferase	0.628571429
Dual specificity protein kinase TTK	0.475105189
Cationic trypsin	0.66399633
3-phosphoinositide-dependent protein kinase 1	0.35520362
Estrogen receptor beta	0.754023605
Neutrophil collagenase	0.646255872
Nitric oxide synthase, inducible	0.636025706
Tyrosine-protein phosphatase non-receptor type 1	0.663433842
Fibroblast growth factor 1	0.487179487
Ephrin type-B receptor 4	0.537207358
Purine nucleoside phosphorylase	0.498378378
Nitric oxide synthase, inducible	0.427472015
Peptidyl-prolyl cis-trans isomerase NIMA-interacting 1	0.735690236
Proto-oncogene tyrosine-protein kinase receptor Ret	0.538395019
N(G),N(G)-dimethylarginine dimethylaminohydrolase 1	0.222222222
Type II inositol 1,4,5-trisphosphate 5-phosphatase	0.375
Pteridine reductase 1	0.714285714
Ephrin type-A receptor 8	0.599413059
Arginase-1	0.328571429
Platelet-derived growth factor receptor beta	0.511208311
Uncharacterized aarF domain-containing protein kinase 4	0.498787879
Leukotriene A-4 hydrolase	1
Tyrosine-protein phosphatase non-receptor type 11	0.559440559
cAMP-dependent protein kinase catalytic subunit alpha	0.493296415
Bromodomain-containing protein 4	0
Tyrosine-protein kinase Lck	0.683981602
Tyrosine-protein kinase JAK2	0.583809524
Nitric oxide synthase, endothelial	0.410628019
Serine/threonine-protein kinase MARK2	0.53452381
Calcium/calmodulin-dependent protein kinase type II subunit gamma	0.525297619
Fibroblast growth factor receptor 2	0.552083333
Tyrosine-protein kinase Lck	0.685131487
Multidrug resistance-associated protein 1	0.28125
Ribonuclease pancreatic	0.437888199
Aldose reductase	0.472222222
Myosin-IIIb	0.35154827
Peptidyl-prolyl cis-trans isomerase NIMA-interacting 1	0.767436267
Dipeptidyl peptidase 4	0.663752422
D(1A) dopamine receptor	0.449790047
Fibroblast growth factor receptor 3	0.683467742
Serine/threonine-protein kinase Nek2	0.568076923
Metabotropic glutamate receptor 1	0.709134615
Mitogen-activated protein kinase 8	0.474241158
Collagenase 3	0.486807453
Cationic trypsin	0.598836364
Progesterone receptor	0.596400796
Cathepsin K	0.575309292
Neutrophil collagenase	0.646532191
Glutathione S-transferase P	0.8
Nitric oxide synthase, endothelial	0.435350952
Prothrombin	0.427160518
Cathepsin L1	0.688682171

PROTEIN FAMILY	AUC
Delta-type opioid receptor	0.544762497
Indolethylamine N-methyltransferase	0.666666667
Serum albumin	0.381944444
Tyrosine-protein kinase ZAP-70	0.470017637
Trypsin	0.958333333
Carnitine O-acetyltransferase	0.555555556
Sodium- and chloride-dependent glycine transporter 1	0.818181818
Mitogen-activated protein kinase 10	0.524512987
Peptidyl-prolyl cis-trans isomerase A	0.905405405
Death-associated protein kinase 1	0.522424242
Estrogen receptor	0.662654376
Cathepsin S	0.6230421
cGMP-dependent protein kinase 1	0.560483871
Beta-lactamase	0.206730769
Peroxisome proliferator-activated receptor gamma	0.750260417
Glutamate receptor ionotropic, kainate 3	0.525
RAC-alpha serine/threonine-protein kinase	0.602809706
Cationic trypsin	0.642296919
Androgen receptor	0.527996383
Peroxisome proliferator-activated receptor gamma	0.740538194
Gamma-aminobutyric acid receptor subunit alpha-6	0.497222222
Thyroid hormone receptor beta	0.171703297
Cyclin-dependent kinase 2	0.364955904
Orotidine 5'-phosphate decarboxylase	0.471428571
Tyrosine-protein kinase ABL1	0.570320226
Carbonic anhydrase 2	0.760243485
Dipeptidyl peptidase 4	0.698922061
Liver carboxylesterase 1	0.393830128
Gamma-aminobutyric acid receptor subunit gamma-1	0.333333333
cAMP-dependent protein kinase catalytic subunit alpha	0.641795395
Prothrombin	0.443598015
cGMP-specific 3',5'-cyclic phosphodiesterase	0.901477833
Dihydrofolate reductase	0.637476117
Ribonuclease pancreatic	0.254658385
Serine/threonine-protein kinase 24	0.484195402
Coagulation factor XI	0.428104575
Serum albumin	0.416666667
Calcium/calmodulin-dependent protein kinase type II subunit delta	0.42020202
Tyrosine-protein kinase Fes/Fps	0.419117647
Orotidine 5'-phosphate decarboxylase	0.442857143
Beta-secretase 1	0.867998231
Thymidylate synthase	0.52238806
Wee1-like protein kinase	0.596428571
Bcl-2-related protein A1	1
Poly [ADP-ribose] polymerase 1	0.357304217
Beta-secretase 1	0.841244104
Epidermal growth factor receptor	0.718867521
Cathepsin K	0.502105817
Peptidyl-prolyl cis-trans isomerase FKBP1A	0.818926807
Somatostatin receptor type 4	0.489035088
3-hydroxy-3-methylglutaryl-coenzyme A reductase	0.698529412
Myosin light chain kinase, smooth muscle	0.468686869
Interleukin-1 receptor-associated kinase 4	0.629451668

PROTEIN FAMILY	AUC
Carbonic anhydrase 2	0.761684529
Protein delta homolog 1	0.75
Serine/threonine-protein kinase Chk1	0.687254902
Dual specificity protein kinase CLK1	0.518306636
Breast cancer type 1 susceptibility protein	0.5
RAC-alpha serine/threonine-protein kinase	0.598339719
Serine/threonine-protein kinase TAO3	0.527217742
AMP deaminase 3	0.611426768
Phosphatidylinositol 4,5-bisphosphate 3-kinase catalytic subunit gamma isoform	0.662356322
Fibroblast growth factor receptor 3	0.661656891
Tyrosine-protein kinase Lck	0.622637736
Trypsin	0.908333333
Beta-galactosidase	0.850649351
Cathepsin K	0.540422918
Ephrin type-B receptor 2	0.369747899
Bromodomain-containing protein 4	0.5
Serine/threonine-protein kinase MARK2	0.609920635
Glutamate carboxypeptidase 2	0.391774892
Beta-lactamase	0.203703704
ALK tyrosine kinase receptor	0.540127841
S-ribosylhomocysteine lyase	0.799450549
Ephrin type-A receptor 7	0.492616899
Urokinase-type plasminogen activator	0.577325448
Fibroblast growth factor 1	0.615384615
Glutamate receptor 3	0.393939394
Receptor activity-modifying protein 1	0.632653061
Dual specificity protein kinase CLK2	0.418333333
Serine/threonine-protein kinase 35	0.417270531
Tyrosine-protein phosphatase non-receptor type 1	0.635763314
Glycogen synthase kinase-3 beta	0.601882956
Nitric oxide synthase, inducible	0.622542843
Proto-oncogene tyrosine-protein kinase receptor Ret	0.492909028
Estrogen receptor beta	0.760491178
Aurora kinase A	0.546339869
Corticosteroid 11-beta-dehydrogenase isozyme 1	0.616383616
3-hydroxy-3-methylglutaryl-coenzyme A reductase	0.772058824
Glycogen synthase kinase-3 beta	0.598081683
Caspase-1	0.69800198
Mitogen-activated protein kinase kinase kinase 11	0.438690476
Delta-type opioid receptor	0.524597574
Beta-lactamase OXA-10	1
D(3) dopamine receptor	0.439470374
Phosphatidylinositol 4,5-bisphosphate 3-kinase catalytic subunit gamma isoform	0.675287356
Mitogen-activated protein kinase 14	0.631224858
Fibroblast growth factor receptor 1	0.644444444
Dipeptidyl peptidase 4	0.720927922
Mitogen-activated protein kinase 8	0.495126706
Growth factor receptor-bound protein 2	0.446969697
Mitogen-activated protein kinase 12	0.784382767
Cyclin-dependent kinase 2	0.374142577
Phosphatidylinositol 4-kinase alpha	0.4736
Ornithine carbamoyltransferase, mitochondrial	0.55
Xanthine dehydrogenase/oxidase	0.791666667

PROTEIN FAMILY	AUC
Serine/threonine-protein kinase 32B	0.673275862
Integrin alpha-L	0.894308943
Caspase-1	0.603096031
Receptor tyrosine-protein kinase erbB-4	0.4
Phosphatidylinositol 4-kinase alpha	0.604
NUAK family SNF1-like kinase 1	0.641818182
Myosin-IIIa	0.440801457
Macrophage migration inhibitory factor	0.9375
Pteridine reductase 1	0.70995671
Cationic trypsin	0.626376341
Interleukin-1 receptor-associated kinase 4	0.723007349
Acetylcholinesterase	1
Nitric oxide synthase, endothelial	0.424836601
Lysozyme	0.814814815
Proto-oncogene tyrosine-protein kinase Src	0.592673993
Adenosine kinase	0.59375
Calcium/calmodulin-dependent protein kinase type II subunit beta	0.477183833
Cathepsin K	0.529121699
Beta-1,4-galactosyltransferase 1	0.5
Ribonuclease pancreatic	0.50621118
Transient receptor potential cation channel subfamily M member 6	0.838709677
Nitric oxide synthase, inducible	0.644247732
Tyrosine-protein kinase ABL1	0.515209903
Mitogen-activated protein kinase 9	0.590749415
Focal adhesion kinase 1	0.514351852
Scytalone dehydratase	0.445273632
Estrogen receptor beta	0.746870529
Vascular endothelial growth factor receptor 3	0.524250441
Orotidine 5'-phosphate decarboxylase	0.464285714
Carbonic anhydrase 6	0.700059102
Dihydroorotate dehydrogenase (quinone), mitochondrial	0.706363636
S-adenosylmethionine decarboxylase proenzyme	0.572727273
Trifunctional purine biosynthetic protein adenosine-3	0.690789474
Proto-oncogene tyrosine-protein kinase Src	0.696688447
Ribonuclease pancreatic	0.416149068
Ribosyldihydronicotinamide dehydrogenase [quinone]	0.133333333
Mitogen-activated protein kinase 8	0.466026177
CREB-binding protein	0.555555556
Ribonuclease pancreatic	0.5
Deoxyuridine 5'-triphosphate nucleotidohydrolase, mitochondrial	0.623315364
3-phosphoinositide-dependent protein kinase 1	0.425339367
Myotonin-protein kinase	0.52866861
1-deoxy-D-xylulose 5-phosphate reductoisomerase	0.104166667
Carbonic anhydrase 2	0.760384836
S-ribosylhomocysteine lyase	0.804945055
Calpain small subunit 1	0.603125
Angiopoietin-1 receptor	0.642314991
Dihydrofolate reductase	0.578354693
Fibroblast growth factor 1	0.709401709
Stromelysin-1	0.48396838
Protein kinase C iota type	0.603278689
Phosphatidylinositol 4,5-bisphosphate 3-kinase catalytic subunit gamma isoform	0.693965517
Leukotriene A-4 hydrolase	1

PROTEIN FAMILY	AUC
Alpha-1A adrenergic receptor	0.683683853
Mineralocorticoid receptor	0.412778367
Tissue-type plasminogen activator	0.457481527
Peroxisome proliferator-activated receptor alpha	0.287166086
Nitric oxide synthase, inducible	0.402402052
Melanin-concentrating hormone receptor 1	0.594890817
Renin	0.761126894
Beta-1 adrenergic receptor	0.604780876
Calcium/calmodulin-dependent protein kinase type II subunit gamma	0.548115079
Serine/threonine-protein kinase 17B	0.587418301
Thermolysin	0.598918919
Peroxisome proliferator-activated receptor gamma	0.760329861
Glutathione S-transferase Mu 2	0.4
Serine/threonine-protein kinase MRCK beta	0.667410714
Cationic trypsin	0.61833004
Gamma-aminobutyric acid receptor subunit alpha-5	0.541332263
Myosin light chain kinase, smooth muscle	0.51010101
Peptidyl-prolyl cis-trans isomerase A	0.996621622
Mu-type opioid receptor	0.536035242
Coagulation factor XI	0.41503268
Urokinase-type plasminogen activator	0.570886595
Serine/threonine-protein kinase ICK	0.493440233
Tyrosine-protein kinase Mer	0.613095238
Fibroblast growth factor 1	0.555555556
Proto-oncogene tyrosine-protein kinase Src	0.528913309
Tyrosine-protein kinase Srms	0.402043269
Protein-tyrosine kinase 2-beta	0.592647059
Myosin-IIIb	0.463261649
Nitric oxide synthase, inducible	0.428754664
Gamma-aminobutyric acid receptor subunit beta-3	0.527679236
Cyclin-dependent kinase 3	0.499305556
Serine/threonine-protein kinase receptor R3	0.657559199
5-hydroxytryptamine receptor 1D	0.515997785
Phosphatidylinositol 4,5-bisphosphate 3-kinase catalytic subunit gamma isoform	0.625
Serine/threonine-protein kinase Chk1	0.644607843
Dihydrofolate reductase	0.55408971
Tyrosine-protein kinase Lck	0.646785321
Glyceraldehyde-3-phosphate dehydrogenase	0.642857143
Queuine tRNA-ribosyltransferase	0.571428571
Carboxypeptidase A1	0.202508961
Prothrombin	0.435742573
Prostaglandin E2 receptor EP4 subtype	0.657401925
Caspase-9	0.833333333
Mitogen-activated protein kinase 14	0.667030795
Serine/threonine-protein kinase 33	0.591938406
Mitogen-activated protein kinase 14	0.619711472
Beta-1,4-galactosyltransferase 1	1
Mitogen-activated protein kinase 10	0.606764069
Thymidylate synthase	0.614842454
Serine/threonine-protein kinase STK11	0.637704918
Prostasin	0.902173913
WD repeat-containing protein 5	0.347826087
Protein delta homolog 1	0.25

PROTEIN FAMILY	AUC
Cathepsin K	0.555435641
Cyclin-dependent kinase 18	0.513157895
Myosin light chain kinase, smooth muscle	0.51043771
Endothelin B receptor	0.559791818
Estrogen receptor	0.744686325
Thermolysin	0.621081081
Thermolysin	0.55518018
Beta-secretase 1	0.857900943
Fibroblast growth factor receptor 2	0.537660256
N(G),N(G)-dimethylarginine dimethylaminohydrolase 1	0.333333333
Thymidylate synthase	0.506218905
Myosin light chain kinase, smooth muscle	0.392592593
Prothrombin	0.590241035
Serine/threonine-protein kinase 24	0.502192982
Tyrosine-protein phosphatase non-receptor type 1	0.689388368
Mitogen-activated protein kinase kinase kinase 2	0.531052632
Tyrosine-protein kinase BTK	0.402815934
Tyrosine-protein kinase Lck	0.666933307
cAMP-specific 3',5'-cyclic phosphodiesterase 4A	0.596491228
Coagulation factor XI	0.369281046
Peptidyl-prolyl cis-trans isomerase FKBP1A	0.742671114
Plasmepsin-2	0.799514301
Caspase-3	0.797979798
Ribonuclease pancreatic	0.332298137
Serine/threonine-protein phosphatase 2B catalytic subunit alpha isoform	1
Serine/threonine-protein kinase B-raf	0.658163265
Cyclin-dependent kinase 11A	0.567783094
Corticosteroid 11-beta-dehydrogenase isozyme 1	0.616633367
Tyrosine-protein phosphatase non-receptor type 1	0.646089968
Phosphatidylinositol 4,5-bisphosphate 3-kinase catalytic subunit gamma isoform	0.70545977
Peroxisome proliferator-activated receptor gamma	0.705381944
Amine oxidase [flavin-containing] B	0.591094891
Orotidine 5'-phosphate decarboxylase	0.5
Trypsin	0.975
Leukotriene A-4 hydrolase	1
Mitogen-activated protein kinase 14	0.607955334
Prothrombin	0.587795049
Calpain small subunit 1	0.45
Protein-tyrosine kinase 2-beta	0.66127451
Heat shock protein HSP 90-alpha	0.867924528
Mast/stem cell growth factor receptor Kit	0.715353856
Glutamate receptor ionotropic, NMDA 2B	0.202503682
Serine/threonine-protein kinase pim-1	0.691342989
Death-associated protein kinase 1	0.408024691
Androgen receptor	0.632882002
Tyrosine-protein kinase ABL1	0.516360877
Carbonic anhydrase 2	0.764325112
Trypsin	0.975
Beta-lactamase	0.198275862
Cationic trypsin	0.647730507
N-acetylated-alpha-linked acidic dipeptidase 2	0.947368421
Peroxisome proliferator-activated receptor alpha	0.289488966
Beta-lactamase	0.209876543

PROTEIN FAMILY	AUC
Beta-1,4-galactosyltransferase 1	1
Granulocyte colony-stimulating factor receptor	0
Ectonucleotide pyrophosphatase/phosphodiesterase family member 2	0.592372881
Inosine-5'-monophosphate dehydrogenase 1	0.703472222
Tyrosine-protein kinase ABL1	0.539346247
Ribosyldihydronicotinamide dehydrogenase [quinone]	0.155555556
Fibroblast growth factor receptor 1	0.589795918
Thermolysin	0.628108108
Protein kinase C iota type	0.416393443
3-phosphoshikimate 1-carboxyvinyltransferase	0.688888889
High affinity nerve growth factor receptor	0.312907432
Proto-oncogene tyrosine-protein kinase ROS	0.522807018
Serine/threonine-protein kinase ICK	0.551020408
Heat shock protein HSP 90-alpha	0.863207547
Beta-secretase 1	0.853552476
Cyclin-dependent kinase 2	0.402294627
Glucocorticoid receptor	0.710938846
Phosphatidylinositol 4,5-bisphosphate 3-kinase catalytic subunit alpha isoform	0.716847237
Squalene monooxygenase	1
Mitogen-activated protein kinase 14	0.673983909
TRAF2 and NCK-interacting protein kinase	0.610741971
Serine/threonine-protein kinase mTOR	0.693961029
Nitric oxide synthase, endothelial	0.443165672
Corticosteroid 11-beta-dehydrogenase isozyme 1	0.601898102
Coagulation factor VII	0.717004572
Glycogen phosphorylase, liver form	0
M-phase inducer phosphatase 3	0.5
Activin receptor type-1	0.59691358
Serum albumin	0.381944444
Serine/threonine-protein kinase SIK1	0.562244898
Thermolysin	0.669189189
Alcohol dehydrogenase E chain	0.277777778
Fibroblast growth factor receptor 1	0.715646259
Homeodomain-interacting protein kinase 1	0.558531746
Serum albumin	0.409722222
Thyroid hormone receptor alpha	0.046323529
Hepatocyte growth factor receptor	0.662438337
Carbonic anhydrase 5B, mitochondrial	0.772579542
Proto-oncogene tyrosine-protein kinase Src	0.725787546
Bone morphogenetic protein 1	0.801694915
cAMP-specific 3',5'-cyclic phosphodiesterase 4B	0.864347357
Receptor activity-modifying protein 1	0.503759398
Mitogen-activated protein kinase 3	0.846491228
Thymidylate synthase	0.621212121
Serine/threonine-protein kinase tousled-like 1	0.493066256
Uridine 5'-monophosphate synthase	0.2
Mitogen-activated protein kinase 3	0.557777778
Peroxisomal N(1)-acetyl-spermine/spermidine oxidase	1
Dual specificity mitogen-activated protein kinase kinase 6	0.545454545
Hepatocyte growth factor receptor	0.584390416
Cationic trypsin	0.637897043
Hepatocyte growth factor receptor	0.623502467
SRSF protein kinase 3	0.621158392

PROTEIN FAMILY	AUC
G protein-coupled receptor kinase 4	0.472506394
Heat shock protein HSP 90-alpha	0.884433962
3-phosphoshikimate 1-carboxyvinyltransferase	0.677777778
Dual specificity mitogen-activated protein kinase kinase 1	0.430393586
N-acetylated-alpha-linked acidic dipeptidase 2	0.894736842
Bcl-2-like protein 1	0.67468257
Aldose reductase	0.472222222
Serine/threonine-protein kinase pim-1	0.610302831
Glycogen synthase kinase-3 beta	0.54583628
Acetylcholinesterase	0.393333333
Death-associated protein kinase 2	0.605779154
Plasminogen	0.478172589
Nitric oxide synthase, endothelial	0.405797101
Beta-lactamase	0.232193732
Fibroblast growth factor receptor 1	0.664285714
M-phase inducer phosphatase 1	0
Mitogen-activated protein kinase 1	0.703096539
Galectin-7	0.611494253
Activated CDC42 kinase 1	0.430379747
Androgen receptor	0.405269332
Orotidine 5'-phosphate decarboxylase	0.521428571
Aldehyde oxidase	0.571428571
Phosphorylase b kinase gamma catalytic chain, liver/testis isoform	0.516690856
Plasminogen	0.456007029
Fatty acid-binding protein, adipocyte	0.865131579
Proto-oncogene tyrosine-protein kinase Src	0.555824176
Nuclear receptor subfamily 1 group I member 2	0
Beta-lactamase	0.406666667
Fibroblast growth factor 1	0.517094017
Dual specificity protein phosphatase 3	0.714285714
Transthyretin	0.928571429
Acidic mammalian chitinase	1
Melanocortin receptor 4	0.595952598
5-hydroxytryptamine receptor 1E	0.416666667
Aldo-keto reductase family 1 member C3	0.777777778
Proto-oncogene tyrosine-protein kinase Src	0.447972973
Mitogen-activated protein kinase 14	0.696386461
3-phosphoinositide-dependent protein kinase 1	0.371493213
Serine/threonine-protein kinase Nek2	0.480384615
Dihydroorotate dehydrogenase (quinone), mitochondrial	0.661287879
Cationic trypsin	0.6531178
Alpha-galactosidase A	0.833333333
Proto-oncogene tyrosine-protein kinase Src	0.495213675
Lanosterol 14-alpha demethylase	0.125
Gastrin/cholecystokinin type B receptor	0.695642508
Trypsin	0.958333333
MAP kinase-activated protein kinase 5	0.488262911
Glycogen synthase kinase-3 beta	0.590678041
Cyclin-dependent kinase 2	0.347542055
Fibroblast growth factor receptor 2	0.520061728
Receptor tyrosine-protein kinase erbB-4	0.415816327
Dihydrofolate reductase	0.530433991
Microtubule-associated serine/threonine-protein kinase 1	0.377572016

PROTEIN FAMILY	AUC
Prothrombin	0.551333717
RAC-alpha serine/threonine-protein kinase	0.616271552
cAMP-specific 3',5'-cyclic phosphodiesterase 4B	0.743257821
Serum albumin	0.493055556
Amine oxidase [flavin-containing] B	0.62973236
Thymidylate synthase	0.653679654
Thymidylate kinase	0.875
Eukaryotic translation initiation factor 2-alpha kinase 1	0.286206897
Carbonic anhydrase 2	0.753457303
Proto-oncogene tyrosine-protein kinase Src	0.485396825
Serine/threonine-protein kinase PAK 4	0.644914216
MAP kinase-activated protein kinase 2	0.747878788
Fatty acid-binding protein, adipocyte	0.868421053
Glutamate receptor ionotropic, kainate 5	0.33974359
Estrogen receptor	0.628109898
Liver carboxylesterase 1	0.502097474
Beta-secretase 1	0.792231722
Prothrombin	0.476043982
Dual specificity mitogen-activated protein kinase kinase 1	0.461734694
Cationic trypsin	0.654737903
Cytochrome P450 19A1	0.544103774
Hepatocyte growth factor receptor	0.605708245
Ribonuclease pancreatic	0.319875776
RAC-beta serine/threonine-protein kinase	0.366084081
Ephrin type-A receptor 5	0.416069295
Hypoxanthine-guanine phosphoribosyltransferase	0.47737069
Ephrin type-A receptor 5	0.562756598
Melanocortin receptor 5	0.742456317
N(G),N(G)-dimethylarginine dimethylaminohydrolase 1	0.555555556
Tyrosine-protein kinase Lck	0.662633737
Serine/threonine-protein kinase B-raf	0.640412415
Fibroblast growth factor receptor 1	0.627430556
Dihydrofolate reductase	0.658402329
Serine/threonine-protein kinase TAO1	0.586080586
Serine/threonine-protein kinase TAO2	0.444816054
Histone deacetylase 7	0.975
Histone deacetylase-like amidohydrolase	0.363636364
Caspase-1	0.733417334
Insulin-like growth factor 1 receptor	0.678151261
Ephrin type-B receptor 4	0.552897574
Hepatocyte growth factor receptor	0.672348485
Growth factor receptor-bound protein 2	0.681818182
Mitogen-activated protein kinase kinase kinase 1	0.495884774
Thyroid hormone receptor beta	0.170787546
Ribosomal protein S6 kinase alpha-1	0.454948646
Beta-1,4-galactosyltransferase 1	0.46875
Homeodomain-interacting protein kinase 4	0.427083333
Histone deacetylase 7	0.547619048
Glycogen phosphorylase, muscle form	0.285714286
Eukaryotic translation initiation factor 2-alpha kinase 4	0.4138322
Poly [ADP-ribose] polymerase 3	0.333333333
Vascular endothelial growth factor receptor 2	0.574457056
WD repeat-containing protein 5	0.369565217

PROTEIN FAMILY	AUC
Disintegrin and metalloproteinase domain-containing protein 17	0.56362589
Collagenase 3	0.454236025
Dipeptidyl peptidase 4	0.68310069
Serum albumin	0.409722222
Serine/threonine-protein kinase DCLK2	0.410606061
Cyclin-dependent kinase 2	0.372652295
Histamine H4 receptor	0.401808786
Dipeptidyl peptidase 4	0.688068154
Ribonuclease pancreatic	0.338509317
Amine oxidase [flavin-containing] B	0.606618005
Beta-lactamase OXA-10	1
Carbonic anhydrase 1	0.709945517
Glutathione S-transferase A1	0.933333333
Cyclin-dependent kinase 2	0.365017148
Beta-1,4-galactosyltransferase 1	1
1-deoxy-D-xylulose 5-phosphate reductoisomerase	0.333333333
Nitric oxide synthase, inducible	0.627173639
Serum albumin	0.326388889
Carbonic anhydrase 5A, mitochondrial	0.849301202
Thermolysin	0.597297297
N(G),N(G)-dimethylarginine dimethylaminohydrolase 1	0.277777778
Integrin alpha-L	0.697154472
Cathepsin S	0.701833409
Hepatocyte growth factor receptor	0.611213883
Thymidylate synthase	0.607503608
Tyrosine-protein kinase ABL1	0.571138697
Insulin-like growth factor 1 receptor	0.637815126
Melanocortin receptor 5	0.543075903
Eukaryotic translation initiation factor 2-alpha kinase 4	0.456349206
Heat shock protein HSP 90-alpha	0.889150943
Tryptase delta	0.46875
Stromelysin-1	0.48359528
Renin	0.780421402
Nitric oxide synthase, inducible	0.651934224
Acetylcholinesterase	0.46
Protein kinase C zeta type	1
Cyclin-dependent kinase 2	0.398272905
Neuraminidase	0.333333333
Serum albumin	0.4375
Beta-2 adrenergic receptor	0.663767463
Aurora kinase A	0.55624183
Serine/threonine-protein kinase tousled-like 1	0.386748844
Serine/threonine-protein kinase Chk2	0.833333333
Cationic trypsin	0.627484159
Growth factor receptor-bound protein 2	0.553030303
Glycogen synthase kinase-3 alpha	0.552197802
Ephrin type-B receptor 2	0.56197479
Thymidylate synthase	0.691919192
Angiopoietin-1 receptor	0.595625
Prothrombin	0.410493977
Testis-specific serine/threonine-protein kinase 1	0.324884793
Aurora kinase A	0.519803922
Serine/threonine-protein kinase Nek2	0.497975709

PROTEIN FAMILY	AUC
Phenylethanolamine N-methyltransferase	0.516727941
Calcium/calmodulin-dependent protein kinase kinase 2	0.488864943
Aurora kinase A	0.534764228
Scytalone dehydratase	0.73880597
ALK tyrosine kinase receptor	0.621803977
TGF-beta receptor type-1	0.524818087
Hepatocyte growth factor receptor	0.582526448
Aldose reductase	0.458333333
Cathepsin B	0.537921348
Carboxypeptidase A1	0.208109319
Heat shock protein HSP 90-alpha	0.83254717
Cholinesterase	0.815955285
Macrophage migration inhibitory factor	1
Lysozyme	0.574074074
Caspase-3	0.804292929
Acetylcholinesterase	1
Uridine 5'-monophosphate synthase	0.3
Poly [ADP-ribose] polymerase 1	0.331701807
Epidermal growth factor receptor	0.726536017
Thymidylate synthase	0.613275613
Insulin receptor-related protein	0.376373626
Queuine tRNA-ribosyltransferase	0.685714286
Fibroblast growth factor receptor 1	0.566319444
Stromelysin-1	0.43896325
Beta-secretase 1	0.821860259
Purine nucleoside phosphorylase	0.61222973
cAMP-dependent protein kinase catalytic subunit alpha	0.409647333
Neutrophil elastase	0.556695858
Growth factor receptor-bound protein 2	0.537878788
Serine/threonine-protein kinase RIO1	0.598039216
Transthyretin	0.75
Protein S100-B	0.166666667
Serine/threonine-protein kinase Nek11	0.484693878
Prothrombin	0.428611858
Phosphatidylinositol 4-kinase beta	0.365029664
Thymidylate synthase	0.551409619
Alpha-galactosidase A	0.333333333
Peroxisome proliferator-activated receptor delta	0.560846561
Maltase-glucoamylase, intestinal	0.066666667
Receptor activity-modifying protein 1	0.62585034
Purine nucleoside phosphorylase	0.529761905
Peroxisome proliferator-activated receptor gamma	0.727430556
Mitogen-activated protein kinase 8	0.570732387
Orotidine 5'-phosphate decarboxylase	0.457142857
3-oxoacyl-[acyl-carrier-protein] synthase 3	0.857142857
Prothrombin	0.550389864
M1 family aminopeptidase	0.071428571
Serine/threonine-protein kinase pim-2	0.739485294
Alcohol dehydrogenase 1C	0.383333333
Acetylcholinesterase	0.46
SRSF protein kinase 3	0.602628285
Death-associated protein kinase 1	0.447878788
Alcohol dehydrogenase E chain	0.277777778

PROTEIN FAMILY	AUC
Prothrombin	0.430184578
Phosphatidylinositol 4,5-bisphosphate 3-kinase catalytic subunit gamma isoform	0.49137931
Thymidylate synthase	0.571969697
Urokinase-type plasminogen activator	0.540158746
Dihydrofolate reductase	0.556148513
5'-AMP-activated protein kinase catalytic subunit alpha-2	0.576802508
Hypoxanthine-guanine phosphoribosyltransferase	0.514008621
Macrophage metalloelastase	0.415572479
Beta-2 adrenergic receptor	0.597476341
Proto-oncogene tyrosine-protein kinase ROS	0.69047619
Caspase-1	0.68702187
Poly [ADP-ribose] polymerase 1	0.328298087
Alcohol dehydrogenase 1A	0.211538462
cAMP-dependent protein kinase catalytic subunit alpha	0.395511513
Cyclin-dependent kinase 2	0.354748489
Coagulation factor VII	0.827320612
Poly [ADP-ribose] polymerase 3	1
Purine nucleoside phosphorylase	0.590675676
Glutathione S-transferase Mu 1	0.1
Glutamate carboxypeptidase 2	0.606060606
Aminopeptidase N	0.666666667
Collagenase 3	0.400968944
Bromodomain-containing protein 4	0.375
3-dehydroquinate dehydratase	0.240740741
Prostaglandin G/H synthase 1	0.6
Tyrosine-protein kinase JAK3	0.640862069
Cathepsin K	0.521514434
Insulin-like growth factor-binding protein 3	0.953846154
Cathepsin B	0.636959337
Coagulation factor X	0.475257146
Myotonin-protein kinase	0.461127308
cAMP-specific 3',5'-cyclic phosphodiesterase 4A	0.596491228
Coagulation factor X	0.546137594
Metallo-beta-lactamase L1	0.855820106
Heat shock protein HSP 90-alpha	0.886792453
Alpha-1A adrenergic receptor	0.811793306
Insulin receptor	0.708920188
Tyrosine-protein kinase SYK	0.383237626
Glycogen phosphorylase, liver form	0.142857143
Tryptase alpha/beta-1	0.189171123
Cyclin-dependent kinase 13	0.535650624
Corticosteroid 11-beta-dehydrogenase isozyme 1	0.574925075
Neutrophil elastase	0.587712096
Cathepsin L1	0.617858456
Rho-associated protein kinase 1	0.4005
Nitric oxide synthase, inducible	0.644720262
Peptidyl-prolyl cis-trans isomerase NIMA-interacting 1	0.736652237
Beta-lactamase	0.203869048
Tyrosine-protein phosphatase non-receptor type 1	0.714806643
Queuine tRNA-ribosyltransferase	0.721428571
Acetylcholinesterase	0.5
cAMP-specific 3',5'-cyclic phosphodiesterase 4B	0.86596548
cAMP-dependent protein kinase catalytic subunit beta	0.68079096

PROTEIN FAMILY	AUC
Acetylcholinesterase	1
Cruzipain	0.988888889
C-X-C chemokine receptor type 2	0.711279461
Serine/threonine-protein kinase Chk1	0.643872549
Cyclin-dependent kinase 2	0.330470516
Ribosyldihydronicotinamide dehydrogenase [quinone]	0.133333333
Serine/threonine-protein kinase Chk2	0.609259259
Dipeptidyl peptidase 4	0.698847549
Tyrosine-protein kinase ABL1	0.572144733
Chymotrypsinogen A	0.619135802
Caspase-3	0.887626263
Maltase-glucoamylase, intestinal	0.2
Acidic mammalian chitinase	1
Glycogen phosphorylase, muscle form	0.714285714
Calcium/calmodulin-dependent protein kinase type 1	0.427579365
Bifunctional dihydrofolate reductase-thymidylate synthase	0.512169518
Progesterone receptor	0.637818774
Mitogen-activated protein kinase 14	0.682809682
Dipeptidyl peptidase 4	0.694724554
Uridine 5'-monophosphate synthase	0.15
Integrin alpha-L	0.867886179
Calcium/calmodulin-dependent protein kinase type II subunit beta	0.466025641
Chymotrypsinogen A	0.629965753
Gamma-aminobutyric acid receptor subunit beta-2	0.620833333
5-hydroxytryptamine receptor 1E	0.410353535
Carbonic anhydrase 2	0.762552142
Platelet-derived growth factor receptor beta	0.581558125
Tryptase alpha/beta-1	0.353609626
Glutathione S-transferase P	0.675
Renin	0.760653409
Coagulation factor VII	0.763157895
Somatostatin receptor type 5	0.659782609
Ephrin type-A receptor 5	0.579450418
Tyrosine-protein kinase ABL1	0.555885617
MAP kinase-activated protein kinase 2	0.672727273
Sodium/glucose cotransporter 1	0.477272727
Receptor-type tyrosine-protein phosphatase C	0.583333333
Caspase-6	0.35
Proto-oncogene tyrosine-protein kinase Src	0.55023199
Trypsin	0.841666667
Peptidyl-prolyl cis-trans isomerase FKBP1A	0.789835782
Integrin alpha-L	0.077235772
Dual specificity protein kinase CLK2	0.404722222
Mandelate racemase	0.375
Nitric oxide synthase, endothelial	0.400966184
Cationic trypsin	0.674734653
cAMP-dependent protein kinase catalytic subunit alpha	0.420139901
Mitogen-activated protein kinase 10	0.584307359
Ephrin type-A receptor 5	0.510155317
Aldose reductase	0.611111111
Interleukin-1 receptor-associated kinase 1	0.592788462
Myosin light chain kinase, smooth muscle	0.427946128
Dipeptidyl peptidase 4	0.696711539

PROTEIN FAMILY	AUC
Peptidyl-prolyl cis-trans isomerase FKBP1A	0.787750978
Gonadotropin-releasing hormone receptor	0.529591758
Ephrin type-B receptor 6	0.505555556
Tyrosine-protein kinase Lck	0.616288371
Estrogen receptor	0.537128602
3-hydroxy-3-methylglutaryl-coenzyme A reductase	0.757352941
Cyclin-dependent kinase 2	0.344765638
Serine/threonine-protein kinase Chk1	0.60245098
Epithelial discoidin domain-containing receptor 1	0.663476874
Dipeptidyl peptidase 4	0.710272714
Poly [ADP-ribose] polymerase 1	0.333584337
Phosphatidylinositol 4,5-bisphosphate 3-kinase catalytic subunit gamma isoform	0.607758621
Dual specificity mitogen-activated protein kinase kinase 1	0.441326531
Nitric oxide synthase, inducible	0.631961946
Glutamate carboxypeptidase 2	0.621065375
Integrin alpha-IIb	0.474670719
Ectonucleotide pyrophosphatase/phosphodiesterase family member 1	0.416666667
Tyrosine-protein kinase ABL1	0.53420923
Tankyrase-2	0.916666667
SHC-transforming protein 1	0
Acetylcholinesterase	0.446666667
Proto-oncogene tyrosine-protein kinase Src	0.504957265
Tyrosine-protein kinase Yes	0.401500938
Glutamate carboxypeptidase 2	0.346320346
Dehydrosqualene synthase	0.777777778
Poly [ADP-ribose] polymerase 1	0.313629518
MAP kinase-interacting serine/threonine-protein kinase 1	0.536363636
Estrogen receptor beta	0.730433357
Proto-oncogene tyrosine-protein kinase ROS	0.293859649
Bifunctional dihydrofolate reductase-thymidylate synthase	0.498639191
Purine nucleoside phosphorylase	0.546013514
Serine/threonine-protein kinase PLK1	0.432391138
M-phase inducer phosphatase 3	0.5
[Pyruvate dehydrogenase [lipoamide]] kinase isozyme 1, mitochondrial	0
Beta-2 adrenergic receptor	0.624515548
Serum albumin	0.5
Eukaryotic translation initiation factor 2-alpha kinase 4	0.441043084
Mitogen-activated protein kinase 10	0.546103896
Chymotrypsin-like elastase family member 1	0.505882353
Sodium channel protein type 4 subunit alpha	0.820512821
Poly [ADP-ribose] polymerase 1	0.366716867
Casein kinase II subunit alpha'	0.415374677
Neutrophil collagenase	0.636363636
Renin	0.794981061
Mitogen-activated protein kinase 14	0.708246636
Phenylethanolamine N-methyltransferase	0.761764706
Beta-lactamase	0.188131313
Chymotrypsinogen A	0.718918919
Ephrin type-A receptor 5	0.535777126
Tyrosine-protein kinase ABL1	0.5776012
Beta-secretase 1	0.829967571
Prothrombin	0.436149083
Casein kinase I isoform delta	0.490021383

PROTEIN FAMILY	AUC
Beta-lactamase	0.413333333
Neuropeptide Y receptor type 1	0.71611667
Kinesin-like protein KIF11	0.701923077
Progesterone receptor	0.607994212
Thymidylate synthase	0.608946609
Thymidylate synthase	0.629148629
Purine nucleoside phosphorylase	0.60972973
Coagulation factor XI	0.421568627
Cyclin-dependent kinase 2	0.364588437
Retinoic acid receptor alpha	0.794210526
Macrophage colony-stimulating factor 1 receptor	0.622724766
Receptor-type tyrosine-protein phosphatase beta	0.65
Tyrosine-protein phosphatase non-receptor type 1	0.708877245
Vascular endothelial growth factor receptor 3	0.590560072
Mitogen-activated protein kinase 14	0.579050492
Bromodomain-containing protein 4	0.25
RAC-beta serine/threonine-protein kinase	0.42867265
Activated CDC42 kinase 1	0.416017527
Coagulation factor X	0.548814115
Serine/threonine-protein kinase Nek4	0.637711864
Heat shock protein HSP 90-alpha	0.883254717
cAMP-specific 3',5'-cyclic phosphodiesterase 4D	0.618363118
Induced myeloid leukemia cell differentiation protein Mcl-1	0.838461538
Receptor-type tyrosine-protein phosphatase epsilon	0
Serine/threonine-protein kinase Chk2	0.687962963
Leukotriene A-4 hydrolase	0.333333333
Dual specificity tyrosine-phosphorylation-regulated kinase 1B	0.522108844
Acetylcholinesterase	1
Ephrin type-A receptor 3	0.558950617
Tryptase alpha/beta-1	0.219251337
cGMP-specific 3',5'-cyclic phosphodiesterase	0.903119869
5'-methylthioadenosine/S-adenosylhomocysteine nucleosidase	0.11627907
Cationic trypsin	0.664449345
Ribonuclease pancreatic	0.456521739
Wee1-like protein kinase 2	0.474937343
Lethal(3)malignant brain tumor-like protein 1	0.166666667
Cyclin-dependent kinase 5	0.527101335
Heat shock protein HSP 90-alpha	0.851415094
Prothrombin	0.422636675
Coagulation factor VII	0.770373683
Tyrosine-protein kinase Blk	0.641049383
Ribonuclease pancreatic	0.437888199
Carbonic anhydrase 2	0.763174507
cAMP-specific 3',5'-cyclic phosphodiesterase 4A	0.78320802
Chymotrypsinogen A	0.697260274
Microtubule-associated serine/threonine-protein kinase 1	0.419753086
Papain	0.447490453
Purine nucleoside phosphorylase	0.626621622
Adenosine receptor A2a	0.350711703
Tyrosine-protein kinase ABL1	0.527367595
Leukotriene A-4 hydrolase	1
Phosphatidylinositol 4,5-bisphosphate 3-kinase catalytic subunit gamma isoform	0.676724138
Peptidyl-prolyl cis-trans isomerase A	0.939189189

PROTEIN FAMILY	AUC
Epidermal growth factor receptor	0.731313559
Adenosine kinase	0.7
Nischarin	0.43904321
Aldose reductase	0.388888889
Serum albumin	0.395833333
Prothrombin	0.578414352
Myosin light chain kinase, smooth muscle	0.462289562
Carboxypeptidase A1	0.718137255
Glutathione S-transferase A1	0.65
Orotidine 5'-phosphate decarboxylase	0.442857143
Sodium channel protein type 9 subunit alpha	0.588803089
Lethal factor	0.387237762
Estrogen receptor	0.678270986
Cationic trypsin	0.643905437
Cyclin-dependent kinase 5	0.557344855
cAMP-dependent protein kinase catalytic subunit alpha	0.550422617
Thermolysin	0.413913914
Beta-lactamase OXA-10	1
cAMP-specific 3',5'-cyclic phosphodiesterase 4B	0.857065804
RAC-gamma serine/threonine-protein kinase	0.553947368
Prothrombin	0.514266818
Acetylcholinesterase	1
Casein kinase II subunit alpha	0.438540457
Dihydrofolate reductase	0.658265854
Glutamate receptor 3	0.363636364
Receptor-type tyrosine-protein phosphatase F	0.784090909
Cholinesterase	0.848869411
3-phosphoinositide-dependent protein kinase 1	0.415384615
Serine/threonine-protein kinase 33	0.533913043
Aldose reductase	0.416666667
Alpha-galactosidase A	1
Multidrug resistance-associated protein 1	0.21875
cAMP-dependent protein kinase catalytic subunit alpha	0.610171962
Mitogen-activated protein kinase 14	0.72258635
Type-1 angiotensin II receptor	0.760742188
Tyrosine-protein phosphatase non-receptor type 1	0.675323282
Nitric oxide synthase, inducible	0.481693097
Receptor-type tyrosine-protein phosphatase beta	0.875
Aminopeptidase N	1
Estrogen receptor	0.648424915
Tyrosine-protein kinase ITK/TSK	0.700328707
Endochitinase	0.666666667
Urokinase-type plasminogen activator	0.539888874
Proto-oncogene tyrosine-protein kinase Src	0.595531136
ALK tyrosine kinase receptor	0.595170455
Ephrin type-B receptor 3	0.615671642
Coagulation factor X	0.522770943
Cationic trypsin	0.666317235
Thromboxane A2 receptor	0.451500326
Galectin-7	0.740229885
Phenylethanolamine N-methyltransferase	0.537040441
Serine/threonine-protein kinase PLK1	0.40565317
Thyroid hormone receptor beta	0.059090909

PROTEIN FAMILY	AUC
Mitogen-activated protein kinase kinase kinase 19	0.401864035
Glucocorticoid receptor	0.608383577
Bacterial leucyl aminopeptidase	0.333333333
Alcohol dehydrogenase E chain	0.247222222
RAC-gamma serine/threonine-protein kinase	0.660666667
Cyclin-dependent kinase 11A	0.472089314
cAMP-dependent protein kinase catalytic subunit alpha	0.435004372
Serine/threonine-protein kinase pim-1	0.703522054
Serine/threonine-protein kinase SIK2	0.579545455
Transthyretin	0.428571429
Macrophage-stimulating protein receptor	0.485324948
Caspase-8	0.978021978
Cathepsin L1	0.653479252
Dihydrofolate reductase	0.168224299
Amine oxidase [flavin-containing] B	0.611922141
Rho-associated protein kinase 1	0.476351351
Neutrophil collagenase	0.66999171
Insulin receptor	0.731052985
cAMP-specific 3',5'-cyclic phosphodiesterase 4A	0.180451128
Beta-secretase 1	0.795400943
Purine nucleoside phosphorylase	0.43452381
Somatostatin receptor type 1	0.46105919
Aurora kinase A	0.559707317
Protein S100-B	1
Glutathione S-transferase P	0.875
Receptor activity-modifying protein 1	0.646258503
Ribosyldihydronicotinamide dehydrogenase [quinone]	0.055555556
Myosin light chain kinase 3	0.642142857
Aldose reductase	0.638888889
Serine/threonine-protein kinase Nek11	0.330357143
Histamine H1 receptor	0.60737416
Estrogen receptor beta	0.733950286
Type I inositol 1,4,5-trisphosphate 5-phosphatase	0.621428571
Cyclin-dependent kinase 9	0.399821641
Nuclear receptor subfamily 1 group I member 2	0.5
Proto-oncogene tyrosine-protein kinase Src	0.523296703
Peptidyl-prolyl cis-trans isomerase A	0.904842342
Sodium channel protein type 4 subunit alpha	0.512820513
Calcium/calmodulin-dependent protein kinase type II subunit gamma	0.467757937
Peptidyl-prolyl cis-trans isomerase FKBP4	0.261904762
Ribosomal protein S6 kinase alpha-1	0.503968254
Beta-lactamase	0.200892857
Insulin receptor	0.706908115
Serine/threonine-protein kinase tousled-like 1	0.389830508
Heat shock protein HSP 90-alpha	0.879716981
Alcohol dehydrogenase E chain	0.277777778
5-hydroxytryptamine receptor 7	0.506599455
Tyrosine-protein kinase BTK	0.455357143
AMP deaminase 3	0.644886364
Cyclin-dependent kinase 2	0.356524579
Cathepsin K	0.540686145
Fatty acid-binding protein, adipocyte	0.733552632
Proto-oncogene tyrosine-protein kinase Src	0.591672772

PROTEIN FAMILY	AUC
Caspase-3	0.948232323
Serine/threonine-protein kinase Chk1	0.637990196
Casein kinase II subunit alpha	0.432238395
Aurora kinase A	0.54198374
Cyclin-dependent kinase 2	0.408259708
5-hydroxytryptamine receptor 3C	0.866666667
5-hydroxytryptamine receptor 2C	0.726950823
Proto-oncogene tyrosine-protein kinase Src	0.67978022
Dipeptidyl peptidase 4	0.717972282
SRSF protein kinase 2	0.629098361
Retinoic acid receptor RXR-alpha	0.504980564
Coagulation factor XI	0.470588235
Phenylethanolamine N-methyltransferase	0.761764706
Carbonic anhydrase 2	0.759776548
Receptor tyrosine-protein kinase erbB-4	0.4975
Tyrosine-protein kinase CSK	0.481132075
Serine/threonine-protein kinase 24	0.405172414
cAMP-dependent protein kinase catalytic subunit alpha	0.427863597
Retinoic acid receptor alpha	0.753355263
Acidic mammalian chitinase	1
Amine oxidase [flavin-containing] B	0.601557178
Fatty-acid amide hydrolase 1	0.566360276
Thermolysin	0.633888889
Proto-oncogene tyrosine-protein kinase Src	0.59980464
Angiopoietin-1 receptor	0.648590686
Suppressor of tumorigenicity 14 protein	0.422169811
Serum albumin	0.451388889
T-cell surface glycoprotein CD4	0.357142857
Serum albumin	0.381944444
Caspase-3	0.933080808
Alcohol dehydrogenase E chain	0.225
Carbonic anhydrase 2	0.747958807
Cationic trypsin	0.680142857
Mitogen-activated protein kinase 10	0.577164502
Serine/threonine-protein kinase Nek11	0.441326531
Dihydrofolate reductase	0.557137658
Beta-lactamase OXA-10	1
Fibroblast growth factor receptor 2	0.571714744
Prothrombin	0.425270613
Myosin light chain kinase, smooth muscle	0.530639731
Serine/threonine-protein kinase B-raf	0.65795068
Mitogen-activated protein kinase 3	0.372807018
Peptidyl-prolyl cis-trans isomerase A	0.885749386
Serine/threonine-protein kinase ULK2	0.444148936
Amine oxidase [flavin-containing] B	0.612408759
Vascular endothelial growth factor receptor 1	0.578647416
Fibroblast growth factor receptor 1	0.710544218
Tyrosine-protein phosphatase non-receptor type 1	0.65831648
cAMP-specific 3',5'-cyclic phosphodiesterase 4D	0.670810671
Dihydroorotate dehydrogenase (quinone), mitochondrial	0.694772727
Heat shock protein HSP 90-alpha	0.850235849
SRSF protein kinase 2	0.648565574
Tyrosine-protein phosphatase non-receptor type 1	0.663464485

PROTEIN FAMILY	AUC
Serine/threonine-protein kinase tousled-like 1	0.437596302
Tyrosine-protein phosphatase non-receptor type 1	0.753615861
Cathepsin B	0.612470862
Substance-P receptor	0.601556639
Mitogen-activated protein kinase 14	0.693508115
Peptidyl-prolyl cis-trans isomerase A	0.914977477
Disintegrin and metalloproteinase domain-containing protein 17	0.634059489
Glutathione S-transferase P	0.7
Muscarinic acetylcholine receptor M5	0.498435871
Serine/threonine-protein kinase VRK2	0.563793103
Eukaryotic translation initiation factor 2-alpha kinase 1	0.407017544
Disintegrin and metalloproteinase domain-containing protein 17	0.618873062
Peptidyl-prolyl cis-trans isomerase A	0.90990991
Coagulation factor VII	0.785032797
Thymidylate synthase	0.591750842
Chymotrypsinogen A	0.636815068
Neutrophil elastase	0.609081372
Aurora kinase A	0.559642276
Serine/threonine-protein kinase NLK	0.517241379
Aurora kinase A	0.559479675
Cathepsin K	0.533824691
Nitric oxide synthase, endothelial	0.425262859
Hepatocyte growth factor receptor	0.592230444
BDNF/NT-3 growth factors receptor	0.403813559
Tyrosine-protein kinase Lck	0.616388361
Prothrombin	0.47276745
Proto-oncogene tyrosine-protein kinase Src	0.591013431
BDNF/NT-3 growth factors receptor	0.585956416
Uridine 5'-monophosphate synthase	0.15
Cell division protein ZipA	0
Ribonuclease pancreatic	0.338509317
Cytosol aminopeptidase	0.623626374
Tyrosine-protein phosphatase non-receptor type 1	0.660461482
Cathepsin L1	0.580907828
Serine/threonine-protein kinase NLK	0.612068966
Glutamine--tRNA ligase	1
Beta-lactamase	0.215384615
Serine/threonine-protein kinase B-raf	0.633822279
5'-methylthioadenosine/S-adenosylhomocysteine nucleosidase	0.162790698
Cationic trypsin	0.665105981
Thymidylate synthase	0.709235209
Gamma-aminobutyric acid receptor subunit beta-1	0.5
Glycogen phosphorylase, liver form	0
Casein kinase II subunit alpha	0.447348045
Caspase-1	0.681486815
3-oxoacyl-[acyl-carrier-protein] synthase 3	1
Beta-1 adrenergic receptor	0.624568393
Death-associated protein kinase 2	0.563467492
Trypsin	0.841666667
Dipeptidyl peptidase 4	0.713749938
Caspase-3	0.950757576
Mitogen-activated protein kinase kinase kinase kinase 4	0.536625514
Receptor tyrosine-protein kinase erbB-4	0.5175

PROTEIN FAMILY	AUC
Lysosomal alpha-glucosidase	0.259259259
Protein S100-B	0
Mitogen-activated protein kinase 14	0.727267998
Dihydrofolate reductase	0.33625731
Protein kinase C theta type	0.442897498
cGMP-specific 3',5'-cyclic phosphodiesterase	0.920361248
Serine/threonine-protein kinase Nek1	0.51814059
Cathepsin L1	0.623246694
Retinoic acid receptor alpha	0.725592105
cAMP-specific 3',5'-cyclic phosphodiesterase 4D	0.564361564
Cationic trypsin	0.678786575
Kallikrein-1	0.488716957
Serine hydroxymethyltransferase, cytosolic	0.5
Dual specificity mitogen-activated protein kinase kinase 3	0.552406417
Carbonic anhydrase 2	0.768771343
Lethal factor	0.56993007
Serine/threonine-protein kinase pim-1	0.686240948
Nitric oxide synthase, endothelial	0.461068485
Protein kinase C theta type	0.460451977
Endothelin B receptor	0.392519971
Alcohol dehydrogenase 1C	0.441666667
Tyrosine-protein phosphatase non-receptor type 1	0.638306674
Acetylcholinesterase	1
3-dehydroquinate dehydratase	0.826666667
Vasopressin V2 receptor	0.424931129
Ribonuclease pancreatic	0.369565217
3-dehydroquinate dehydratase	0.906666667
Tyrosine-protein kinase Lck	0.662983702
Urokinase-type plasminogen activator	0.469093476
Leukocyte tyrosine kinase receptor	0.487083333
Serine/threonine-protein kinase Chk1	0.603431373
Cationic trypsin	0.682626636
Cationic trypsin	0.680234239
RAC-alpha serine/threonine-protein kinase	0.621753938
Heat shock protein HSP 90-alpha	0.885613208
Adenosine deaminase	0.749475891
Cathepsin K	0.520610687
Mitogen-activated protein kinase 14	0.630115134
Cyclin-dependent kinase 16	0.479084967
Neuromedin-K receptor	0.770184544
Platelet-derived growth factor receptor alpha	0.49025974
Pseudolysin	0.62449187
WD repeat-containing protein 5	0.369565217
Histone deacetylase 7	0.404761905
Coagulation factor X	0.536975024
Ephrin type-A receptor 4	0.661923077
cAMP-dependent protein kinase catalytic subunit alpha	0.531769164
Leukotriene A-4 hydrolase	1
5'-methylthioadenosine/S-adenosylhomocysteine nucleosidase	0.162790698
Disintegrin and metalloproteinase domain-containing protein 17	0.626256808
Chymotrypsinogen A	0.6640625
Queuine tRNA-ribosyltransferase	0.685714286
Caspase-3	0.897727273

PROTEIN FAMILY	AUC
RAC-alpha serine/threonine-protein kinase	0.624308216
Heat shock protein HSP 90-alpha	0.882075472
Receptor tyrosine-protein kinase erbB-3	0.482993197
UDP-3-O-[3-hydroxymyristoyl] N-acetylglucosamine deacetylase	0.125
Multidrug resistance-associated protein 1	0.03125
Neuropeptide Y receptor type 1	0.611677116
Acetylcholinesterase	1
ALK tyrosine kinase receptor	0.635298295
Mitogen-activated protein kinase 14	0.689485366
Disintegrin and metalloproteinase domain-containing protein 17	0.594627147
Excitatory amino acid transporter 1	0.884615385
Alcohol dehydrogenase 1A	0.682692308
Beta-2 adrenergic receptor	0.532492114
Peptidyl-prolyl cis-trans isomerase A	0.927927928
Caspase-9	0.166666667
Urokinase-type plasminogen activator	0.56239002
Cyclin-dependent kinase 15	0.525326797
cAMP-dependent protein kinase catalytic subunit alpha	0.394491402
Serine/threonine-protein kinase SBK1	0.546310832
Retinoic acid receptor RXR-alpha	0.452259475
Type-1 angiotensin II receptor	0.833007813
Epidermal growth factor receptor	0.730338983
Prothrombin	0.453246388
Heat shock protein HSP 90-beta	0.6
Mitogen-activated protein kinase 1	0.667304189
Receptor tyrosine-protein kinase erbB-3	0.476190476
Cyclin-dependent kinase 18	0.44619883
Urokinase-type plasminogen activator	0.539521828
Carnitine O-acetyltransferase	0.333333333
Chymotrypsinogen A	0.725342466
Thermolysin	0.596216216
Prothrombin	0.422455835
Macrophage migration inhibitory factor	1
Receptor-type tyrosine-protein phosphatase epsilon	0.45
Coagulation factor V	0.5
Growth factor receptor-bound protein 2	0.553030303
Vascular endothelial growth factor receptor 2	0.572547201
Ribosyldihydronicotinamide dehydrogenase [quinone]	0.127777778
Receptor tyrosine-protein kinase erbB-4	0.5435
Thymidylate synthase	0.709235209
Urokinase-type plasminogen activator	0.553140718
Cathepsin L1	0.648217054
Carbonic anhydrase 13	0.763513514
Orotidine 5'-phosphate decarboxylase	0.35
Mitogen-activated protein kinase 14	0.647246497
Endochitinase	0.666666667
Serine/threonine-protein kinase PLK1	0.464858671
Glutamate receptor 3	0.484848485
Prolyl endopeptidase	0.222727273
Purine nucleoside phosphorylase	0.410714286
Cathepsin E	0.125
Insulin receptor	0.666666667
Gamma-aminobutyric acid receptor subunit gamma-3	0.333333333

PROTEIN FAMILY	AUC
Serine/threonine-protein kinase D3	0.532352941
Serine/threonine-protein kinase Chk1	0.607711443
Ribonuclease pancreatic	0.450310559
Receptor tyrosine-protein kinase erbB-3	0.448611111
Caspase-1	0.661596616
Bacterial leucyl aminopeptidase	0.212121212
Ornithine carbamoyltransferase, mitochondrial	0.7
Protein S100-B	0
Cathepsin E	0.666666667
Leukotriene A-4 hydrolase	1
Dipeptidyl peptidase 9	0.542032164
Macrophage-stimulating protein receptor	0.585429769
Cationic trypsin	0.687219888
Heat shock protein HSP 90-alpha	0.870283019
Interstitial collagenase	0.476563904
Thymidylate kinase	0.767857143
Leukotriene A-4 hydrolase	1
Estrogen receptor	0.706204135
Nuclear receptor corepressor 2	0.666666667
Serine/threonine-protein kinase pim-3	0.818459191
Nitric oxide synthase, endothelial	0.426115374
Estradiol 17-beta-dehydrogenase 1	0.666666667
Corticosteroid 11-beta-dehydrogenase isozyme 1	0.61038961
Baculoviral IAP repeat-containing protein 2	nan
Casein kinase I isoform gamma-3	0.449922958
Myosin light chain kinase, smooth muscle	0.559259259
Heat shock protein HSP 90-alpha	0.818396226
Renin	0.722419508
Aurora kinase B	0.631610385
Mitogen-activated protein kinase 14	0.663895903
Lethal(3)malignant brain tumor-like protein 1	0.375
Ephrin type-A receptor 6	0.602134146
Transient receptor potential cation channel subfamily M member 6	0.180327869
Urokinase-type plasminogen activator	0.546803353
Sodium- and chloride-dependent glycine transporter 1	0.96969697
Casein kinase II subunit alpha'	0.413221361
3-phosphoshikimate 1-carboxyvinyltransferase	0.644444444
Epidermal growth factor receptor	0.709480932
Acetylcholinesterase	0.446666667
Acetylcholinesterase	0.46
Acetylcholinesterase	1
Amine oxidase [flavin-containing] B	0.603990268
Androgen receptor	0.629607
Kinesin-like protein KIF11	0.788461538
S-methyl-5'-thioadenosine phosphorylase	0.435897436
Nitric oxide synthase, endothelial	0.41943734
Melanocortin receptor 3	0.683812379
Glycogen phosphorylase, muscle form	0.714285714
Dihydrofolate reductase	0.604449095
Aurora kinase A	0.549788618
Cathepsin B	0.672507123
Lethal(3)malignant brain tumor-like protein 1	0.041666667
Sodium-dependent serotonin transporter	0.663505685

PROTEIN FAMILY	AUC
Fibroblast growth factor receptor 1	0.522762346
Protein kinase C iota type	0.577419355
Inosine-5'-monophosphate dehydrogenase 2	0.769379845
Acetylcholinesterase	1
Cyclin-dependent kinase 9	0.374076355
Cationic trypsin	0.703169822
Thymidylate synthase	0.611111111
Adenosine receptor A3	0.455125716
WD repeat-containing protein 5	0.282608696
Serine/threonine-protein kinase Sgk1	0.428571429
Adenosine deaminase	0.836419753
Steroid hormone receptor ERR1	1
Serine/threonine-protein kinase B-raf	0.627338435
Tyrosine-protein kinase Lck	0.631486851
Acetylcholinesterase	0.46
Dual serine/threonine and tyrosine protein kinase	0.390670554
Serum albumin	0.4375
Thymidylate synthase	0.616915423
Nitric oxide synthase, inducible	0.63719128
3-phosphoinositide-dependent protein kinase 1	0.357211538
Heat shock protein HSP 90-alpha	0.847877358
Serine/threonine-protein kinase Chk1	0.600245098
Alcohol dehydrogenase E chain	0.3
Serine/threonine-protein kinase Chk1	0.635539216
Seminal ribonuclease	0.666666667
Cathepsin B	0.653367003
Bromodomain-containing protein 4	0.625
Nitric oxide synthase, inducible	0.63202495
3-phosphoinositide-dependent protein kinase 1	0.386877828
Estrogen receptor beta	0.738316643
Wee1-like protein kinase 2	0.451530612
Glycogen synthase kinase-3 beta	0.572975601
Dipeptidyl peptidase 4	0.70401371
Retinoic acid receptor RXR-beta	0.576858108
Bile salt-activated lipase	0.392857143
Receptor-type tyrosine-protein phosphatase C	0.643518519
Ribosyldihydronicotinamide dehydrogenase [quinone]	0.122222222
Cathepsin S	0.739271163
Galectin-7	0.698850575
Phosphatidylinositol 4-phosphate 5-kinase type-1 alpha	0.612578616
3-hydroxy-3-methylglutaryl-coenzyme A reductase	0.617647059
Cyclin-dependent kinase 2	0.35107382
Beta-secretase 1	0.861438679
3-hydroxy-3-methylglutaryl-coenzyme A reductase	0.757352941
Cannabinoid receptor 2	0.500326232
M-phase inducer phosphatase 2	0.4
Retinoic acid receptor alpha	0.701052632
Receptor tyrosine-protein kinase erbB-3	0.555555556
Thymidylate kinase	0.719355986
Tyrosine-protein kinase JAK3	0.524827586
Proto-oncogene tyrosine-protein kinase Src	0.702832723
Calcium/calmodulin-dependent protein kinase type II subunit delta	0.436868687
SNF-related serine/threonine-protein kinase	0.471929825

PROTEIN FAMILY	AUC
Thymidylate synthase	0.611832612
Progesterone receptor	0.513727618
Beta-secretase 1	0.796875
Neutrophil collagenase	0.62959381
Urokinase-type plasminogen activator	0.556597666
Cyclin-dependent kinase 2	0.339233219
Mitogen-activated protein kinase 8	0.546545667
Cationic trypsin	0.583333333
Beta-lactamase	0.190860215
Coagulation factor X	0.540214803
Carbonic anhydrase 2	0.770006545
Xaa-Pro dipeptidase	0.5
Kappa-type opioid receptor	0.492689551
Tyrosine-protein phosphatase non-receptor type 1	0.724842189
Seminal ribonuclease	0.666666667
Serum albumin	0.416666667
Phosphatidylinositol 4,5-bisphosphate 3-kinase catalytic subunit gamma isoform	0.664473684
Maltase-glucoamylase, intestinal	0.133333333
Transthyretin	0.892857143
Nitric oxide synthase, endothelial	0.374822393
Dihydrofolate reductase	0.499269006
Mitogen-activated protein kinase 14	0.665782355
Acetylcholinesterase	0.353333333
Mu-type opioid receptor	0.555126937
Gastricsin	0.490740741
Serine/threonine-protein kinase pim-1	0.673337722
Thymidylate synthase	0.670995671
Peroxisome proliferator-activated receptor delta	0.671957672
Muscarinic acetylcholine receptor M4	0.588995726
Integrin alpha-L	0.975609756
Urokinase-type plasminogen activator	0.557910252
Protein kinase C zeta type	0.6
Prothrombin	0.440741575
Activin receptor type-2A	0.388888889
Glycogen phosphorylase, muscle form	0.714285714
Phosphatidylinositol 4,5-bisphosphate 3-kinase catalytic subunit delta isoform	0.49122807
Abelson tyrosine-protein kinase 2	0.57641196
STE20-like serine/threonine-protein kinase	0.561533888
Carbonic anhydrase 4	0.813864451
Chymotrypsinogen A	0.72058307
Ribonuclease pancreatic	0.419254658
Nitric oxide synthase, inducible	0.446944963
Mitogen-activated protein kinase 7	0.410526316
Coagulation factor XI	0.549019608
Cyclin-dependent kinase 5	0.477219167
Nitric oxide synthase, brain	0.432063231
Glutathione S-transferase P	0.7875
Prostaglandin G/H synthase 1	0.8
Cyclin-dependent kinase 15	0.448529412
Prothrombin	0.640563436
Coagulation factor X	0.562100765
MAP kinase-activated protein kinase 5	0.73943662
Macrophage-stimulating protein receptor	0.573899371

PROTEIN FAMILY	AUC
Ephrin type-B receptor 6	0.52059387
Histamine N-methyltransferase	0.166666667
Ephrin type-A receptor 1	0.591094771
Mitogen-activated protein kinase 4	0.393229167
S-methyl-5'-thioadenosine phosphorylase	0.5
Multidrug resistance-associated protein 1	0.625
Metabotropic glutamate receptor 8	0
Cholinesterase	0.827959858
Matrix metalloproteinase-14	0.606435644
Caspase-1	0.710692107
Peptidyl-prolyl cis-trans isomerase NIMA-interacting 1	0.732563733
Serum albumin	0.409722222
cGMP-specific 3',5'-cyclic phosphodiesterase	0.924466338
Estrogen receptor beta	0.732832618
Interleukin-1 receptor-associated kinase 1	0.588461538
cGMP-specific 3',5'-cyclic phosphodiesterase	0.917898194
Fibroblast growth factor receptor 2	0.46720679
E3 ubiquitin-protein ligase XIAP	0.562214137
Melatonin receptor type 1B	0.652515091
Receptor-type tyrosine-protein phosphatase F	0.920454545
Purine nucleoside phosphorylase	0.482142857
Beta-secretase 1	0.852373231
Androgen receptor	0.583744745
Tyrosine-protein phosphatase non-receptor type 1	0.710685175
Integrin alpha-L	0.410569106
MAP kinase-activated protein kinase 2	0.721818182
Beta-lactamase	0.20545977
Aurora kinase A	0.541601307
Interferon-induced, double-stranded RNA-activated protein kinase	0.590435606
Plasmepsin-2	0.794252563
SRSF protein kinase 1	0.657398213
cGMP-specific 3',5'-cyclic phosphodiesterase	0.885057471
5-hydroxytryptamine receptor 3A	0.688412207
Alcohol dehydrogenase E chain	0.238888889
Tyrosine-protein kinase Lck	0.669067284
Renin	0.765506629
Serine/threonine-protein kinase PAK 4	0.742953431
Cyclin-dependent kinase 11A	0.519936204
Tyrosine-protein kinase SYK	0.374487285
Mitogen-activated protein kinase 3	0.688888889
Carbonic anhydrase 2	0.764677263
Cathepsin S	0.672159348
Macrophage metalloelastase	0.307510504
Queuine tRNA-ribosyltransferase	0.657142857
cGMP-specific 3',5'-cyclic phosphodiesterase	0.937602627
Estrogen receptor beta	0.681881855
Activin receptor type-2A	0.438616071
Caspase-8	0.379120879
Interleukin-1 receptor-associated kinase 4	0.721857923
Macrophage-stimulating protein receptor	0.422431866
Glucocorticoid receptor	0.618805627
Calcium/calmodulin-dependent protein kinase type 1D	0.45322327
Dihydrofolate reductase	0.523837685

PROTEIN FAMILY	AUC
Beta-secretase 1	0.826798349
Cathepsin G	0.392006803
Mitogen-activated protein kinase kinase kinase kinase 3	0.561333333
Sodium channel protein type 9 subunit alpha	0.366795367
Mast/stem cell growth factor receptor Kit	0.442034768
Alcohol dehydrogenase E chain	0.255555556
UDP-3-O-[3-hydroxymyristoyl] N-acetylglucosamine deacetylase	0.375
Glucocorticoid receptor	0.608871662
TRAF2 and NCK-interacting protein kinase	0.600623583
Serine/threonine-protein kinase Chk1	0.637009804
Caspase-8	0.846153846
NADPH oxidase 4	0.461734694
Tyrosine-protein phosphatase non-receptor type 1	0.717365325
Proto-oncogene tyrosine-protein kinase Src	0.615311355
Glutamate receptor 4	0.23125
Serine/threonine-protein kinase Sgk3	0.660427807
Serine/threonine-protein kinase PAK 1	0.565217391
Beta-lactamase	0.230769231
3-hydroxy-3-methylglutaryl-coenzyme A reductase	0.654411765
Choline O-acetyltransferase	0.866666667
Coagulation factor XI	0.37254902
Glucosylceramidase	0.795582707
Cationic trypsin	0.595202664
cAMP-dependent protein kinase catalytic subunit beta	0.634920635
Serine/threonine-protein kinase ULK2	0.54587766
Amiloride-sensitive amine oxidase [copper-containing]	0.333333333
MAP kinase-activated protein kinase 2	0.628484848
Proto-oncogene tyrosine-protein kinase ROS	0.487468672
Peroxisome proliferator-activated receptor gamma	0.777864583
Nitric oxide synthase, endothelial	0.404944587
Nitric oxide synthase, endothelial	0.454532538
Phenylethanolamine N-methyltransferase	0.772794118
Cationic trypsin	0.646068605
Arginase-1	0.328571429
TGF-beta receptor type-1	0.680483368
Dual specificity tyrosine-phosphorylation-regulated kinase 2	0.433001107
Corticotropin-releasing factor receptor 2	nan
Homeodomain-interacting protein kinase 1	0.444761905
Urokinase-type plasminogen activator	0.558295669
Phenylethanolamine N-methyltransferase	0.703676471
Aldose reductase	0.444444444
Histamine H4 receptor	0.546996124
Thyroid hormone receptor beta	0.168040293
Cathepsin K	0.554145828
Dihydrofolate reductase	0.260233918
Protein kinase C beta type	0.436363636
Macrophage-stimulating protein receptor	0.524109015
Gamma-aminobutyric acid receptor subunit gamma-2	0.540361486
Beta-secretase 1	0.79635908
Heat shock protein HSP 90-alpha	0.859669811
5-hydroxytryptamine receptor 3D	0.608333333
Serine/threonine-protein kinase Chk1	0.654901961
Xaa-Pro dipeptidase	0.166666667

PROTEIN FAMILY	AUC
Insulin-like growth factor 1 receptor	0.678991597
Receptor-type tyrosine-protein phosphatase alpha	0.995169082
Gamma-aminobutyric acid receptor subunit beta-2	0.485416667
TRAF2 and NCK-interacting protein kinase	0.590808416
Estrogen receptor	0.698093789
Carnitine O-acetyltransferase	0.444444444
Beta-1,4-galactosyltransferase 1	1
Dual specificity tyrosine-phosphorylation-regulated kinase 1B	0.466725821
Tyrosine-protein kinase Mer	0.610119048
Beta-lactamase	0.206790123
Tyrosine-protein kinase Fyn	0.581808586
Prothrombin	0.421478909
Guanine deaminase	0.444444444
Cruzipain	0.988888889
Androgen receptor	0.635643758
RAC-gamma serine/threonine-protein kinase	0.705333333
Cationic trypsin	0.640341553
Serine/threonine-protein kinase LATS1	0.531609195
Receptor tyrosine-protein kinase erbB-2	0.352036199
Angiopoietin-1 receptor	0.668198529
Angiotensin-converting enzyme	0.594769022
Tyrosine-protein kinase BTK	0.457417582
Glutamate receptor ionotropic, kainate 4	0.75
Mineralocorticoid receptor	0.518027572
3-phosphoshikimate 1-carboxyvinyltransferase	0.766666667
Chymotrypsinogen A	0.748287671
Cholinesterase	0.836814024
Peroxisome proliferator-activated receptor gamma	0.797135417
Mitogen-activated protein kinase 10	0.494372294
Coagulation factor IX	0.439884393
Integrin alpha-L	0.829268293
Fibroblast growth factor receptor 3	0.635273369
C-X-C chemokine receptor type 2	0.890572391
Beta-secretase 1	0.810510024
Wee1-like protein kinase	0.533333333
Thyroid hormone receptor beta	0.1753663
Activated CDC42 kinase 1	0.738237401
Protein kinase C beta type	0.467272727
Glutathione S-transferase A1	0.366666667
Gamma-aminobutyric acid receptor subunit alpha-3	0.529487782
Dihydroorotate dehydrogenase (quinone), mitochondrial	0.688181818
Cationic trypsin	0.672511474
Furin	0.734898833
Adenosine deaminase	0.772633745
Carboxypeptidase A1	0.196012545
Type II inositol 1,4,5-trisphosphate 5-phosphatase	0.375
Retinoic acid receptor RXR-alpha	0.660835763
Nitric oxide synthase, inducible	0.599451865
cAMP-dependent protein kinase catalytic subunit alpha	0.522005246
Mitogen-activated protein kinase 14	0.676827577
UDP-3-O-[3-hydroxymyristoyl] N-acetylglucosamine deacetylase	0.875
Thymidylate kinase	0.75
Focal adhesion kinase 1	0.661574074

PROTEIN FAMILY	AUC
Insulin-like growth factor-binding protein 3	0.969230769
S-ribosylhomocysteine lyase	0.744505495
Renin	0.728101326
Gamma-aminobutyric acid receptor subunit gamma-2	0.476800733
Prothrombin	0.450547173
Macrophage migration inhibitory factor	0.9375
Ribonuclease pancreatic	0.447204969
Carbonic anhydrase 2	0.766534352
Aldose reductase	0.527777778
Serine/threonine-protein kinase MARK1	0.651856764
M-phase inducer phosphatase 1	0
Peptidyl-prolyl cis-trans isomerase A	0.923986486
Ribonuclease pancreatic	0.394409938
Beta-lactamase OXA-10	1
Peroxisome proliferator-activated receptor gamma	0.81484375
Peptidyl-prolyl cis-trans isomerase A	0.902027027
Heat shock protein HSP 90-alpha	0.840801887
Matrix metalloproteinase-14	0.451894102
Glucocorticoid receptor	0.584984209
Serine/threonine-protein kinase BRSK2	0.5375
3-phosphoinositide-dependent protein kinase 1	0.338461538
Transthyretin	0.660714286
Tryptase alpha/beta-1	0.192513369
Prothrombin	0.417095077
Gamma-aminobutyric acid receptor subunit beta-3	0.582888584
Beta-lactamase	0.223958333
Insulin receptor	0.67806841
Cationic trypsin	0.650433978
Heat shock protein HSP 90-alpha	0.820754717
3-phosphoinositide-dependent protein kinase 1	0.60678733
Receptor-type tyrosine-protein phosphatase beta	0.55
Glutamate receptor 3	0.424242424
Hydroxyacylglutathione hydrolase, mitochondrial	1
Macrophage metalloelastase	0.365546218
Orotidine 5'-phosphate decarboxylase	0.657142857
Retinoic acid receptor gamma	0.59065367
Androgen receptor	0.38346857
Estrogen receptor	0.721138357
Fibroblast growth factor receptor 1	0.66875
Prothrombin	0.580772262
Peroxisome proliferator-activated receptor delta	0.634920635
NADPH oxidase 4	0.451530612
Tyrosine-protein kinase ABL1	0.573725992
Proto-oncogene tyrosine-protein kinase Src	0.499462759
cGMP-inhibited 3',5'-cyclic phosphodiesterase B	0.44
Prothrombin	0.461986437
Seminal ribonuclease	0.333333333
TRAF2 and NCK-interacting protein kinase	0.60175737
Tyrosine-protein kinase ZAP-70	0.361111111
Coagulation factor X	0.515703658
Cytidine deaminase	0.456730769
Coagulation factor VII	0.671144645
Prothrombin	0.441404145

PROTEIN FAMILY	AUC
Phosphatidylinositol 4-kinase alpha	0.6232
Dipeptidyl peptidase 8	0.515625
Adenosine deaminase	0.826131687
Acetylcholinesterase	1
Glutathione S-transferase Mu 2	0.4
Fatty acid-binding protein, adipocyte	0.835526316
Tyrosine-protein kinase TXK	0.519130435
Gastrin/cholecystokinin type B receptor	0.70636048
Plasmepsin-2	0.790070157
Thymidylate synthase	0.642135642
Alcohol dehydrogenase E chain	0.291666667
Homeodomain-interacting protein kinase 1	0.472380952
Aurora kinase A	0.592
Mitogen-activated protein kinase 14	0.689918851
Peptidyl-prolyl cis-trans isomerase NIMA-interacting 1	0.717412217
Cruzipain	0.988888889
Serine/threonine-protein kinase PLK1	0.432773109
Delta-type opioid receptor	0.579694572
3-oxo-5-alpha-steroid 4-dehydrogenase 1	0.488970588
Beta-lactamase	0.413333333
Serine/threonine-protein kinase MARK1	0.562997347
Estrogen receptor beta	0.602780758
Tyrosine-protein kinase JAK2	0.626187085
Dual specificity mitogen-activated protein kinase kinase 1	0.434766764
Bcl-2-like protein 2	0.735714286
3-phosphoinositide-dependent protein kinase 1	0.363348416
Prothrombin	0.417096887
Glutathione S-transferase Mu 2	0.4
Serine/threonine-protein kinase Nek11	0.371173469
Integrin alpha-L	0.983739837
Chymotrypsinogen A	0.748458904
Serine/threonine-protein kinase NLK	0.595942982
RAF proto-oncogene serine/threonine-protein kinase	0.632352941
Acidic mammalian chitinase	1
Glutamate receptor 2	0.336111111
Carbonic anhydrase 6	0.720375296
Vascular endothelial growth factor receptor 1	0.527562112
Tankyrase-2	0.916666667
Estrogen receptor beta	0.733890677
3-phosphoshikimate 1-carboxyvinyltransferase	0.666666667
Fibroblast growth factor receptor 1	0.553472222
Transient receptor potential cation channel subfamily M member 6	0.844827586
Cyclin-dependent kinase 13	0.437611408
Macrophage colony-stimulating factor 1 receptor	0.666574738
Wee1-like protein kinase 2	0.516581633
Mitogen-activated protein kinase 14	0.688376824
Cyclin-dependent kinase 5	0.53692066
Protein kinase C delta type	0.413304253
3-oxoacyl-[acyl-carrier-protein] synthase 3	0.785714286
Prothrombin	0.529840722
Growth factor receptor-bound protein 2	0.549242424
Estrogen receptor	0.68986039
Cathepsin S	0.711747397

PROTEIN FAMILY	AUC
cAMP-dependent protein kinase catalytic subunit alpha	0.528563101
Acetylcholinesterase	0.46
Thymidylate synthase	0.682524479
Nitric oxide synthase, inducible	0.639900454
Dual specificity protein kinase TTK	0.509467041
Arginase-1	0.271428571
Cationic trypsin	0.659351692
Chymotrypsinogen A	0.639695946
Cationic trypsin	0.655161173
Vasopressin V1b receptor	0.59168956
Peroxisome proliferator-activated receptor gamma	0.719965278
Prothrombin	0.538491371
Serine/threonine-protein kinase B-raf	0.616177721
Cationic trypsin	0.661137775
Dipeptidyl peptidase 4	0.691073469
Vascular endothelial growth factor receptor 2	0.561661028
Peptidyl-prolyl cis-trans isomerase A	0.921171171
Cationic trypsin	0.650566552
Proto-oncogene tyrosine-protein kinase receptor Ret	0.633344863
Carbonic anhydrase 2	0.763024154
Liver carboxylesterase 1	0.616940045
Prothrombin	0.4409149
cAMP-dependent protein kinase catalytic subunit alpha	0.424220344
Protein kinase C theta type	0.40455665
Sialidase-2	0.666666667
Phosphorylase b kinase gamma catalytic chain, liver/testis isoform	0.495108316
Phenylethanolamine N-methyltransferase	0.761764706
Farnesyl pyrophosphate synthase	0.870138889
Dipeptidyl peptidase 1	0.5
Muscarinic acetylcholine receptor M1	0.679004461
Thermolysin	0.601621622
Beta-lactamase	0.204022989
Protein kinase C alpha type	0.651817661
3-phosphoinositide-dependent protein kinase 1	0.533484163
SHC-transforming protein 1	0.85
Vitamin D3 receptor	0.244897959
Histone deacetylase 7	0.357142857
Coagulation factor XI	0.529411765
Serine/threonine-protein kinase 24	0.568247126
Thymidylate synthase	0.627705628
Leukotriene A-4 hydrolase	1
Casein kinase I isoform gamma-1	0.49795082
Tyrosine-protein kinase ABL1	0.539508236
Growth factor receptor-bound protein 2	0.515151515
Mitogen-activated protein kinase 14	0.744000555
Carbonic anhydrase 6	0.735667849
Carbonic anhydrase 2	0.773941662
cGMP-dependent protein kinase 1	0.408602151
Serine/threonine-protein kinase pim-1	0.696148782
Cathepsin S	0.685694885
Serine/threonine-protein kinase Chk1	0.636029412
Kinesin-like protein KIF11	0.798076923
Farnesyl pyrophosphate synthase	0.5

PROTEIN FAMILY	AUC
Thermolysin	0.48972973
Mu-type opioid receptor	0.581103674
Tyrosine-protein kinase ABL1	0.580798349
SRSF protein kinase 3	0.606973995
Ribosyldihydronicotinamide dehydrogenase [quinone]	0.144444444
S-adenosylmethionine decarboxylase proenzyme	0.509090909
Vascular endothelial growth factor receptor 2	0.576148641
Deoxyuridine 5'-triphosphate nucleotidohydrolase, mitochondrial	0.541778976
Beta-2 adrenergic receptor	0.572735466
Tyrosine-protein kinase HCK	0.48255814
Metallo-beta-lactamase L1	0.779100529
Serine/threonine-protein kinase pim-1	0.723897301
Dihydrofolate reductase	0.557748538
Serine/threonine-protein kinase 10	0.530982906
Tyrosine-protein kinase Fer	0.437037037
Adenosine receptor A2a	0.353516752
Coagulation factor IX	0.385260116
Mitogen-activated protein kinase 9	0.545667447
Phenylethanolamine N-methyltransferase	0.725091912
Cyclin-dependent kinase 1	0.655334439
Methionine aminopeptidase 2	0.522865854
Death-associated protein kinase 1	0.543030303
Proprotein convertase subtilisin/kexin type 7	0.222222222
Glucocorticoid receptor	0.603158197
Peroxisome proliferator-activated receptor delta	0.825396825
cAMP-dependent protein kinase catalytic subunit alpha	0.449140192
Ephrin type-A receptor 6	0.521646341
Thermolysin	0.425925926
Metallo-beta-lactamase L1	0.895502646
Dihydrofolate reductase	0.334064327
Alcohol dehydrogenase 1A	0.769230769
Thymidylate synthase	0.651057187
Interstitial collagenase	0.409652557
Renin	0.759114583
Mitogen-activated protein kinase kinase kinase 10	0.574166667
Vascular endothelial growth factor receptor 1	0.477603583
Melanocortin receptor 3	0.487020625
Tubulin--tyrosine ligase	0.294117647
Aurora kinase A	0.542759671
Acetylcholinesterase	1
TRAF2 and NCK-interacting protein kinase	0.620155039
Aldose reductase	0.416666667
Acetylcholinesterase	1
Tyrosine-protein phosphatase non-receptor type 6	0.95
Lysozyme	0.444444444
Stromelysin-1	0.43038779
Metabotropic glutamate receptor 1	0.639423077
Tyrosine-protein kinase SYK	0.431774679
Phosphatidylinositol 4-phosphate 3-kinase C2 domain-containing subunit gamma	0.448275862
5-hydroxytryptamine receptor 1A	0.597747172
Cationic trypsin	0.649864826
Peroxisome proliferator-activated receptor gamma	0.686371528
Dihydroorotate dehydrogenase (quinone), mitochondrial	0.697045455

PROTEIN FAMILY	AUC
Muscarinic acetylcholine receptor M5	0.640622672
Seminal ribonuclease	0
Mitogen-activated protein kinase 15	0.4725
Coagulation factor VII	0.642665474
Neuraminidase	0.666666667
Ribosyldihydronicotinamide dehydrogenase [quinone]	0.138888889
Disintegrin and metalloproteinase domain-containing protein 17	0.557132384
Histone deacetylase 7	0.928571429
S-adenosylmethionine decarboxylase proenzyme	0.527272727
Peptidyl-prolyl cis-trans isomerase A	0.920045045
Mitogen-activated protein kinase kinase kinase 5	0.820895522
Beta-secretase 1	0.808225236
5-hydroxytryptamine receptor 3A	0.693311404
Nuclear receptor subfamily 1 group I member 2	0
Lethal(3)malignant brain tumor-like protein 1	0.125
Serine/threonine-protein kinase PknB	0.410349854
Serine/threonine-protein kinase LATS2	0.690448113
Tyrosine-protein kinase Lck	0.690180982
Thermolysin	0.556216216
Peptidyl-prolyl cis-trans isomerase NIMA-interacting 1	0.769119769
Mitogen-activated protein kinase kinase kinase 15	0.391812865
Prothrombin	0.444172454
Cholinesterase	0.825215955
Beta-secretase 1	0.785229953
Coagulation factor X	0.568421456
Serine/threonine-protein kinase tousled-like 2	0.562037037
Proto-oncogene tyrosine-protein kinase Src	0.614700855
Breast cancer type 1 susceptibility protein	0.375
Macrophage migration inhibitory factor	1
Serine/threonine-protein kinase Chk1	0.655425963
Hepatocyte growth factor receptor	0.586548626
Macrophage metalloelastase	0.343093487
Ephrin type-A receptor 5	0.579151732
Metallo-beta-lactamase L1	0.818783069
Thermolysin	0.503783784
Urokinase-type plasminogen activator	0.549561657
Baculoviral IAP repeat-containing protein 5	0.551724138
Mineralocorticoid receptor	0.477200424
Tyrosine-protein kinase BTK	0.34478022
Chymotrypsinogen A	0.63510274
S-adenosylmethionine decarboxylase proenzyme	0.554545455
Aldose reductase	0.416666667
Aldose reductase	0.722222222
Acetylcholinesterase	0.38
Estrogen receptor beta	0.788388174
Phosphatidylinositol 4-phosphate 5-kinase type-1 gamma	0.745825603
Sodium-dependent serotonin transporter	0.646203748
Renin	0.730823864
Tyrosine-protein phosphatase non-receptor type 1	0.709643317
Tyrosine-protein phosphatase non-receptor type 1	0.752282895
Hepatocyte growth factor receptor	0.524268851
Estradiol 17-beta-dehydrogenase 1	0.666666667
Galectin-9	0.850925926

PROTEIN FAMILY	AUC
Mitogen-activated protein kinase 7	0.364327485
Activin receptor type-1	0.60308642
Growth factor receptor-bound protein 2	0.446969697
Carbonic anhydrase 2	0.770145898
Tyrosine-protein kinase Lck	0.665133487
Dipeptidyl peptidase 4	0.697978243
Granulocyte colony-stimulating factor receptor	0
P2Y purinoceptor 1	0.283906883
Angiotensin-converting enzyme 2	0.683908046
Dihydrofolate reductase	0.485380117
Peptidyl-prolyl cis-trans isomerase NIMA-interacting 1	0.729196729
Uridine 5'-monophosphate synthase	0
Death-associated protein kinase 1	0.518181818
Aldo-keto reductase family 1 member C1	0.833333333
Serine/threonine-protein kinase SIK3	0.615384615
Protein kinase C theta type	0.415456013
Glycogen phosphorylase, liver form	0
Mitogen-activated protein kinase kinase kinase 19	0.589585666
Transketolase	0.581349206
Cyclin-dependent kinase 7	0.45625
Serine/threonine-protein kinase Sgk1	0.857142857
S-methyl-5'-thioadenosine phosphorylase	0.358974359
Thymidylate synthase	0.546897547
Caspase-8	0.714285714
Caspase-1	0.688101881
Mitogen-activated protein kinase kinase kinase 15	0.483918129
Urokinase-type plasminogen activator	0.540005468
Activated CDC42 kinase 1	0.44778481
Retinoic acid receptor alpha	0.542368421
Macrophage metalloelastase	0.344537815
Beta-secretase 1	0.759581368
Bacterial leucyl aminopeptidase	0.242424242
NT-3 growth factor receptor	0.5295
Cytidine deaminase	0.757142857
Aldo-keto reductase family 1 member C3	0.666666667
Estrogen receptor beta	0.737452313
Beta-secretase 1	0.878611439
Substance-P receptor	0.76292532
Prothrombin	0.439477932
Fatty-acid amide hydrolase 1	0.585983854
Prothrombin	0.597687635
Mitogen-activated protein kinase 9	0.538251366
Kappa-type opioid receptor	0.506214812
Nitric oxide synthase, endothelial	0.383631714
Kallikrein-5	0
Myosin-IIIb	0.519125683
Cyclin-dependent kinase 2	0.39896701
Fibroblast growth factor 1	0.52991453
ALK tyrosine kinase receptor	0.600852273
3-hydroxy-3-methylglutaryl-coenzyme A reductase	0.639705882
Phenylethanolamine N-methyltransferase	0.744944853
Dual specificity protein kinase CLK1	0.499122807
Thymidylate synthase	0.657868597

PROTEIN FAMILY	AUC
Serine/threonine-protein kinase LATS1	0.627118644
Tyrosine-protein phosphatase non-receptor type 1	0.615079365
Wee1-like protein kinase	0.561904762
Death-associated protein kinase 1	0.532121212
Gamma-aminobutyric acid receptor subunit alpha-1	0.603886033
cAMP-dependent protein kinase catalytic subunit alpha	0.438356164
Transthyretin	0.642857143
Serine/threonine-protein kinase Chk1	0.668905473
Mitogen-activated protein kinase 10	0.552164502
Ribonuclease pancreatic	0.50310559
Casein kinase II subunit alpha	0.456155633
C-C chemokine receptor type 5	0.454648526
Mu-type opioid receptor	0.583872291
Dual specificity mitogen-activated protein kinase kinase 1	0.439504373
D(3) dopamine receptor	0.575712693
Transthyretin	0.642857143
Glutamate receptor ionotropic, NMDA 1	0.5
Prostacyclin receptor	0.793185789
Alpha-1A adrenergic receptor	0.847071461
Nitric oxide synthase, endothelial	0.460358056
Nitric oxide synthase, endothelial	0.404518329
Transthyretin	0.857142857
cAMP-dependent protein kinase catalytic subunit alpha	0.478286214
Alcohol dehydrogenase E chain	0.288888889
Mineralocorticoid receptor	0.396871686
RAC-gamma serine/threonine-protein kinase	0.424
cAMP-dependent protein kinase catalytic subunit beta	0.648148148
Orotidine 5'-phosphate decarboxylase	0.464285714
cAMP-specific 3',5'-cyclic phosphodiesterase 4B	0.853290183
Fibroblast growth factor receptor 1	0.550347222
Mitogen-activated protein kinase 14	0.681405188
4-aminobutyrate aminotransferase, mitochondrial	0.739130435
Vitamin D3 receptor	0.224489796
Carbonic anhydrase 2	0.748602896
Beta-secretase 1	0.744914505
Insulin receptor	0.684104628
Homeodomain-interacting protein kinase 3	0.427179487
Ribonuclease pancreatic	0.372670807
Collagenase 3	0.433291925
Protein kinase C delta type	0.457618717
Peroxisome proliferator-activated receptor gamma	0.752777778
Glycogen phosphorylase, muscle form	0.285714286
Gastricsin	0.518518519
Hypoxanthine-guanine phosphoribosyltransferase	0.554956897
Protein kinase C iota type	0.558064516
Phenylethanolamine N-methyltransferase	0.690992647
Cationic trypsin	0.634824624
Ephrin type-A receptor 7	0.543209877
Peptidyl-prolyl cis-trans isomerase FKBP1A	0.775065751
Eukaryotic translation initiation factor 2-alpha kinase 4	0.420515575
Bacterial leucyl aminopeptidase	0.272727273
Galectin-9	0.893518519
NUAK family SNF1-like kinase 2	0.537587413

PROTEIN FAMILY	AUC
Carboxypeptidase B2	0.362068966
Calcium/calmodulin-dependent protein kinase type II subunit gamma	0.480567227
Tyrosine-protein phosphatase non-receptor type 1	0.649414721
Glutathione S-transferase P	0.75
Hypoxanthine-guanine phosphoribosyltransferase	0.595905172
Adenosine receptor A3	0.44334826
Coagulation factor X	0.560978723
Kinesin-like protein KIF11	0.807692308
Peptidyl-prolyl cis-trans isomerase FKBP1A	0.813217653
Adenosylhomocysteinase	0.275316456
Serine/threonine-protein kinase Chk2	0.52306968
Urokinase-type plasminogen activator	0.551904382
Corticotropin-releasing factor receptor 2	nan
Peptidyl-prolyl cis-trans isomerase FKBP1A	0.779347617
Tyrosine-protein phosphatase non-receptor type 1	0.617913832
MAP kinase-activated protein kinase 2	0.695151515
Hepatocyte growth factor receptor	0.592688938
Dihydrofolate reductase	0.519005848
Carbonic anhydrase 2	0.767140851
Serine/threonine-protein kinase MRCK alpha	0.632392473
Tyrosine-protein phosphatase non-receptor type 1	0.630906417
Dipeptidyl peptidase 4	0.718642889
Disintegrin and metalloproteinase domain-containing protein 17	0.578917051
Adenosine receptor A2a	0.353794983
Tyrosine-protein kinase JAK3	0.532899367
Collagenase 3	0.341987578
Chymotrypsinogen B	0.279310345
Ribonuclease pancreatic	0.381987578
Mitogen-activated protein kinase 14	0.676844916
Thymidylate kinase	0.767857143
Serine/threonine-protein kinase Chk2	0.628060264
Serine/threonine-protein kinase 3	0.664444444
Catechol O-methyltransferase	0.416666667
Heat shock protein HSP 90-alpha	0.85259434
Glycogen phosphorylase, muscle form	0.285714286
Aurora kinase A	0.570796748
Serine/threonine-protein kinase Chk1	0.64754902
Heat shock protein HSP 90-beta	1
Receptor-type tyrosine-protein phosphatase C	0.569444444
Dehydrosqualene synthase	0.638888889
Cyclin-dependent kinase 15	0.520424837
Trace amine-associated receptor 1	0
Phosphatidylinositol 4,5-bisphosphate 3-kinase catalytic subunit gamma isoform	0.477011494
cAMP-specific 3',5'-cyclic phosphodiesterase 4A	0.644736842
Serine/threonine-protein kinase VRK2	0.30862069
Phosphatidylinositol 4,5-bisphosphate 3-kinase catalytic subunit gamma isoform	0.66954023
Baculoviral IAP repeat-containing protein 2	0.62
Proto-oncogene tyrosine-protein kinase Src	0.480659341
Tyrosine-protein kinase JAK3	0.610517241
Mitogen-activated protein kinase kinase kinase 10	0.546610169
Cyclin-dependent kinase 2	0.342520007
Serine/threonine-protein kinase Chk1	0.62622549
Chymase	0.59223301

PROTEIN FAMILY	AUC
Cationic trypsin	0.639716262
Thymidylate kinase	0.767857143
Serum albumin	0.4375
Phosphorylase b kinase gamma catalytic chain, liver/testis isoform	0.543541364
Cationic trypsin	0.655541383
5'-AMP-activated protein kinase catalytic subunit alpha-2	0.719435737
Serine/threonine-protein kinase Chk2	0.577777778
Fatty acid-binding protein, heart	0.296703297
Acetylcholinesterase	0.393333333
Hepatocyte growth factor receptor	0.557846988
D(2) dopamine receptor	0.604063466
Dual specificity mitogen-activated protein kinase kinase 4	0.468621399
Proto-oncogene tyrosine-protein kinase Src	0.591941392
3-phosphoshikimate 1-carboxyvinyltransferase	0.733333333
Serum albumin	0.520833333
Thymidylate kinase	0.690357588
Ectonucleotide pyrophosphatase/phosphodiesterase family member 1	0.35
Thymidylate kinase	0.767857143
Cationic trypsin	0.667178571
Serine/threonine-protein kinase PLK1	0.433919022
Tyrosine-protein phosphatase non-receptor type 1	0.653520868
Calcium/calmodulin-dependent protein kinase type II subunit delta	0.563157895
Serine/threonine-protein kinase tousled-like 1	0.493939394
Tyrosine-protein phosphatase non-receptor type 11	0.587412587
Cyclin-dependent kinase 11A	0.501567398
Carboxypeptidase A1	0.171594982
Plasminogen	0.333666575
Cathepsin S	0.686283386
Glucocorticoid receptor	0.579758829
cAMP-specific 3',5'-cyclic phosphodiesterase 4D	0.630665631
Glutathione S-transferase Mu 1	0.2
Serum albumin	0.5625
Ephrin type-A receptor 3	0.380997475
Scytalone dehydratase	0.529850746
Acetylcholinesterase	0.393333333
Serine/threonine-protein kinase 17A	0.469155844
Thromboxane A2 receptor	0.473352903
Interleukin-1 receptor-associated kinase 4	0.678688525
Adenosylhomocysteinase	0.294699367
Tyrosine-protein kinase JAK3	0.571034483
Transient receptor potential cation channel subfamily M member 6	0.229508197
Purine nucleoside phosphorylase	0.538503327
Estrogen receptor beta	0.715829161
Serine hydroxymethyltransferase, cytosolic	0.5
Renin	0.739938447
Bone morphogenetic protein receptor type-2	0.5
Proprotein convertase subtilisin/kexin type 9	0.25
Retinoic acid receptor RXR-alpha	0.518586006
Serine/threonine-protein kinase MRCK alpha	0.717213115
Calcium/calmodulin-dependent protein kinase type II subunit gamma	0.301995798
Serine/threonine-protein kinase Chk1	0.677941176
MAP kinase-interacting serine/threonine-protein kinase 1	0.531378601
Proto-oncogene tyrosine-protein kinase Src	0.502124542

PROTEIN FAMILY	AUC
Tyrosine-protein kinase receptor UFO	0.52820122
Kinesin-like protein KIF11	0.677884615
Integrin alpha-L	0.756097561
Dipeptidyl peptidase 4	0.725647012
Beta-secretase 1	0.746757075
Serine/threonine-protein kinase mTOR	0.71310241
Interstitial collagenase	0.422946066
Phosphatidylinositol 4,5-bisphosphate 3-kinase catalytic subunit gamma isoform	0.655890805
Beta-1,4-galactosyltransferase 1	0.375
Beta-1,4-galactosyltransferase 1	1
Orotidine 5'-phosphate decarboxylase	0.428571429
Cathepsin L1	0.631901505
Mitogen-activated protein kinase 14	0.748318075
Cysteinyl leukotriene receptor 1	0.695019554
Wee1-like protein kinase	0.570909091
Neutrophil elastase	0.619674786
Plasmepsin-2	0.747976255
Serine/threonine-protein kinase PLK1	0.446142093
Glutamate receptor ionotropic, NMDA 2B	0.461904762
Trifunctional purine biosynthetic protein adenosine-3	0.703947368
Serine/threonine-protein kinase 33	0.486413043
Calcium/calmodulin-dependent protein kinase kinase 1	0.54009434
Orotidine 5'-phosphate decarboxylase	0.485714286
Abelson tyrosine-protein kinase 2	0.53820598
Transient receptor potential cation channel subfamily V member 1	0.678125
Prostaglandin E2 receptor EP4 subtype	0.733578792
Tyrosine-protein kinase Lck	0.54252451
3-phosphoinositide-dependent protein kinase 1	0.371493213
Neuraminidase	0.583333333
Coagulation factor VII	0.627410058
Proto-oncogene tyrosine-protein kinase Src	0.707765568
Estradiol 17-beta-dehydrogenase 1	0.333333333
Rhodopsin kinase	0.58707483
Abelson tyrosine-protein kinase 2	0.563399779
Thymidylate synthase	0.700120619
Hepatocyte growth factor receptor	0.602448908
Thyroid hormone receptor beta	0.5
Beta-secretase 1	0.799380896
Trypsin	0.933333333
Serine/threonine-protein kinase MRCK alpha	0.478494624
Retinoic acid receptor gamma	0.581651376
Acetylcholinesterase	1
Growth factor receptor-bound protein 2	0.484848485
Squalene monooxygenase	0.833333333
Tubulin beta-2B chain	0
cAMP-specific 3',5'-cyclic phosphodiesterase 4D	0.720668221
Tyrosine-protein phosphatase non-receptor type 1	0.688300545
Homeodomain-interacting protein kinase 1	0.467261905
MAP/microtubule affinity-regulating kinase 3	0.607692308
Fatty-acid amide hydrolase 1	0.560451318
Integrin alpha-IIb	0.46301925
Disintegrin and metalloproteinase domain-containing protein 17	0.612012987
Ribonuclease pancreatic	0.288819876

PROTEIN FAMILY	AUC
Non-receptor tyrosine-protein kinase TYK2	0.551934524
Serine/threonine-protein kinase TBK1	0.420343137
Ribonuclease pancreatic	0.332298137
Myosin-IIIb	0.644808743
Androgen receptor	0.642413726
Thymidylate synthase	0.627705628
Matrix metalloproteinase-16	0.3875
Vascular endothelial growth factor receptor 2	0.661655571
Vitamin D3 receptor	0.244897959
Fibroblast growth factor receptor 2	0.938271605
Peptidyl-prolyl cis-trans isomerase A	0.96509009
Cathepsin B	0.655898876
Cationic trypsin	0.660785147
Serine/threonine-protein kinase ULK2	0.455452128
Prostaglandin G/H synthase 1	0.9
Carboxypeptidase A1	0.16733871
Thymidylate synthase	0.564262023
Arginase-1	0.328571429
Purine nucleoside phosphorylase	0.611418919
Mitogen-activated protein kinase 14	0.676914274
Cytochrome P450 130	1
M-phase inducer phosphatase 3	0.5
Protein-tyrosine kinase 2-beta	0.53250774
Urokinase-type plasminogen activator	0.58553109
Beta-1,4-galactosyltransferase 1	1
Prostaglandin E2 receptor EP3 subtype	0.491599462
Pteridine reductase 1	0.727272727
Phosphatidylinositol 4,5-bisphosphate 3-kinase catalytic subunit gamma isoform	0.5
Serine/threonine-protein kinase PAK 1	0.560200669
ALK tyrosine kinase receptor	0.5546875
Fatty acid-binding protein, adipocyte	0.881578947
Acetylcholinesterase	1
Urokinase-type plasminogen activator	0.538625942
Dual specificity protein kinase TTK	0.486325386
Lanosterol 14-alpha demethylase	0.15625
cAMP-dependent protein kinase catalytic subunit alpha	0.387350627
Eukaryotic translation initiation factor 2-alpha kinase 4	0.419501134
Complement C1s subcomponent	0.304347826
Beta-1,4-galactosyltransferase 1	1
Dual specificity protein kinase CLK3	0.416205534
Gamma-aminobutyric acid receptor subunit alpha-3	0.548539317
Fibroblast growth factor receptor 2	0.618189103
Tyrosine-protein kinase HCK	0.512790698
Growth factor receptor-bound protein 2	0.477272727
cGMP-dependent protein kinase 1	0.497311828
Mitogen-activated protein kinase 14	0.676584825
Hepatocyte growth factor receptor	0.650105708
Mitogen-activated protein kinase 10	0.573376623
Estradiol 17-beta-dehydrogenase 1	0.5
Cytoplasmic tyrosine-protein kinase BMX	0.467942584
Serine/threonine-protein kinase Chk2	0.624537037
Serine/threonine-protein kinase pim-1	0.654147465
Nitric oxide synthase, endothelial	0.469167377

PROTEIN FAMILY	AUC
Farnesyl pyrophosphate synthase	0.494444444
Dual specificity mitogen-activated protein kinase kinase 1	0.395408163
Phosphatidylinositol 4,5-bisphosphate 3-kinase catalytic subunit gamma isoform	0.743534483
Serine/threonine-protein kinase tousled-like 2	0.450094162
Nitric oxide synthase, endothelial	0.378232452
Glutathione S-transferase Mu 2	0.4
Caspase-3	0.940656566
Serine/threonine-protein kinase DCLK3	0.385753932
Carbonic anhydrase 7	0.825069803
Serine/threonine-protein kinase PknB	0.529761905
Tyrosine-protein kinase SYK	0.403882964
Serine/threonine-protein kinase pim-1	0.660006583
Fibroblast growth factor receptor 1	0.67755102
Calcium/calmodulin-dependent protein kinase type II subunit beta	0.437179487
cAMP-specific 3',5'-cyclic phosphodiesterase 4D	0.716265216
Cyclin-dependent kinase 2	0.344194023
Mitogen-activated protein kinase 10	0.568722944
Heat shock protein HSP 90-alpha	0.877358491
3-hydroxy-3-methylglutaryl-coenzyme A reductase	0.602941176
Casein kinase I isoform gamma-3	0.469183359
Melatonin receptor type 1B	0.786317907
Interstitial collagenase	0.421884495
Phosphatidylinositol 4-kinase alpha	0.54
Adenosylhomocysteinase	0.223892405
Galectin-9	0.887962963
Peptidyl-prolyl cis-trans isomerase FKBP1A	0.559914042
Abelson tyrosine-protein kinase 2	0.38482835
Cationic trypsin	0.671686747
Nitric oxide synthase, inducible	0.642830141
Thymidylate kinase	0.683953033
Nischarin	0.273148148
Serine/threonine-protein kinase Chk1	0.61127451
cAMP-dependent protein kinase catalytic subunit alpha	0.665986593
Thymidylate synthase	0.709202498
Peripheral plasma membrane protein CASK	0.548181818
cAMP-dependent protein kinase catalytic subunit alpha	0.426552026
Cyclin-dependent kinase-like 3	0.584770115
Bifunctional dihydrofolate reductase-thymidylate synthase	0.518856921
Dihydrofolate reductase	0.566508962
Proto-oncogene tyrosine-protein kinase Src	0.608034188
Tankyrase-2	0.916666667
Myosin light chain kinase, smooth muscle	0.408417508
Thymidylate synthase	0.575870647
Kinesin-like protein KIF11	0.788461538
Transthyretin	0.75
Nitric oxide synthase, endothelial	0.434640523
Beta-secretase 1	0.848982901
Serine/threonine-protein kinase LATS2	0.587264151
TGF-beta receptor type-1	0.648388773
Caspase-3	0.920454545
Mast/stem cell growth factor receptor Kit	0.601937874
Coagulation factor XI	0.68627451
Serine/threonine-protein kinase Chk1	0.66004902

PROTEIN FAMILY	AUC
Fibroblast growth factor receptor 2	0.588141026
cAMP-specific 3',5'-cyclic phosphodiesterase 4B	0.85167206
Dipeptidyl peptidase 4	0.719984104
Plasmepsin-2	0.792093902
Caspase-3	0.88510101
Multidrug resistance-associated protein 1	0.96875
Serine/threonine-protein kinase MRCK alpha	0.587365591
Nitric oxide synthase, inducible	0.644153226
Trypsin	0.99122807
L-lactate dehydrogenase A chain	0.489583333
RAC-alpha serine/threonine-protein kinase	0.593103448
Mitogen-activated protein kinase kinase kinase 15	0.456896552
Growth factor receptor-bound protein 2	0.46969697
Receptor-interacting serine/threonine-protein kinase 4	0.495714286
Galectin-8	0.465201465
Receptor tyrosine-protein kinase erbB-3	0.606944444
Glutathione S-transferase Mu 2	0.3
Carboxypeptidase A1	0.175403226
Platelet-activating factor receptor	0.62962963
WD repeat-containing protein 5	0.608695652
MAP kinase-interacting serine/threonine-protein kinase 2	0.538333333
Acetylcholinesterase	0.42
Fatty-acid amide hydrolase 1	0.540706157
Growth factor receptor-bound protein 2	0.636363636
Bromodomain-containing protein 4	0.5
5-hydroxytryptamine receptor 7	0.436052587
Peroxisome proliferator-activated receptor gamma	0.650086806
Serine/threonine-protein kinase PRP4 homolog	0.570230608
Beta-lactamase	0.230769231
Aldose reductase	0.555555556
Platelet-derived growth factor receptor beta	0.58775287
Carbonic anhydrase 2	0.763765467
Carbonic anhydrase 2	0.777119843
Tyrosine-protein kinase ITK/TSK	0.669284149
Myelin-associated glycoprotein	1
Tyrosine-protein kinase BTK	0.477678571
Serine/threonine-protein kinase pim-1	0.669289006
Coagulation factor X	0.541027978
Homeodomain-interacting protein kinase 3	0.546153846
Carbonic anhydrase 2	0.775420696
High affinity nerve growth factor receptor	0.667457627
Mitogen-activated protein kinase 1	0.67295082
Acetylcholinesterase	1
3-phosphoinositide-dependent protein kinase 1	0.341628959
3-hydroxy-3-methylglutaryl-coenzyme A reductase	0.919117647
Cationic trypsin	0.656016925
G protein-coupled receptor kinase 4	0.476342711
Amine oxidase [flavin-containing] B	0.620437956
Dipeptidyl peptidase 4	0.701132582
Transthyretin	1
Coagulation factor X	0.576852286
Nitric oxide synthase, endothelial	0.476413754
Tyrosine-protein kinase Fyn	0.61242083

PROTEIN FAMILY	AUC
Caspase-3	0.953282828
Glutathione S-transferase P	0.75
Serine/threonine-protein kinase Nek2	0.536538462
Fatty acid-binding protein, adipocyte	0.838815789
3-dehydroquinate dehydratase	0.853333333
Integrin alpha-L	0.914634146
Peroxisome proliferator-activated receptor gamma	0.681510417
Xanthine dehydrogenase/oxidase	0.297619048
Lanosterol 14-alpha demethylase	0.62962963
Angiopoietin-1 receptor	0.5075
Caspase-3	0.897727273
Insulin receptor	0.668103448
Farnesyl pyrophosphate synthase	0.529861111
Disintegrin and metalloproteinase domain-containing protein 17	0.587819439
Interleukin-1 receptor-associated kinase 3	0.551282051
Aurora kinase A	0.54403252
Prothrombin	0.429731319
Alcohol dehydrogenase E chain	0.216666667
Serine/threonine-protein kinase MRCK beta	0.796783626
Serine/threonine-protein kinase Sgk1	0.785714286
Beta-lactamase	0.195833333
Dual specificity tyrosine-phosphorylation-regulated kinase 1B	0.558333333
Ephrin type-A receptor 4	0.574615385
6,7-dimethyl-8-ribityllumazine synthase	0.397959184
Activated CDC42 kinase 1	0.583830905
Beta-secretase 1	0.806308962
Ephrin type-A receptor 6	0.593597561
STE20-like serine/threonine-protein kinase	0.5460761
Ephrin type-B receptor 4	0.410377358
Aldose reductase	0.5
Fibroblast growth factor receptor 2	0.540509259
Tyrosine-protein kinase ABL1	0.509253094
Heat shock protein HSP 90-alpha	0.883254717
MAP kinase-activated protein kinase 2	0.676969697
Serine/threonine-protein kinase 24	0.488505747
Choline O-acetyltransferase	0.885714286
Poly [ADP-ribose] polymerase 1	0.475075529
3-phosphoinositide-dependent protein kinase 1	0.374208145
Caspase-6	0.416666667
TGF-beta receptor type-1	0.62993763
Cyclin-dependent kinase 9	0.409036861
Caspase-1	0.705517055
Chymotrypsinogen A	0.706678082
Androgen receptor	0.457510509
Thymidine kinase, cytosolic	0.784615385
Lymphokine-activated killer T-cell-originated protein kinase	0
Fibroblast growth factor receptor 2	0.494391026
N(G),N(G)-dimethylarginine dimethylaminohydrolase 1	0.305555556
Nitric oxide synthase, inducible	0.633253528
Serine/threonine-protein kinase PLK1	0.456455309
Stromelysin-1	0.404760517
Gamma-aminobutyric acid receptor subunit alpha-5	0.5644061
Fibroblast growth factor 1	0.611111111

PROTEIN FAMILY	AUC
Kinesin-like protein KIF11	0.778846154
3-phosphoshikimate 1-carboxyvinyltransferase	0.677777778
Alpha-2A adrenergic receptor	0.375856686
Aurora kinase C	0.634953704
Wee1-like protein kinase	0.52797619
Ubiquitin carboxyl-terminal hydrolase isozyme L1	0.833333333
Acetylcholinesterase	0.513333333
Cruzipain	0.955555556
Amiloride-sensitive amine oxidase [copper-containing]	0
ALK tyrosine kinase receptor	0.605823864
S-adenosylmethionine decarboxylase proenzyme	0.5
Renin	0.791577189
cGMP-specific 3',5'-cyclic phosphodiesterase	0.91954023
Serine/threonine-protein kinase D2	0.281868132
Tyrosine-protein kinase ABL1	0.551574904
Serum albumin	0.4375
Acidic mammalian chitinase	1
Seminal ribonuclease	0.666666667
Tyrosine-protein kinase Lck	0.625437456
1-deoxy-D-xylulose 5-phosphate reductoisomerase	0.229166667
Mitogen-activated protein kinase 10	0.53538961
Baculoviral IAP repeat-containing protein 2	0.3
Phosphatidylinositol 4-kinase beta	0.506427159
Death-associated protein kinase 3	0.661147388
Peptidyl-prolyl cis-trans isomerase A	0.916666667
Macrophage metalloelastase	0.363314076
Calpain small subunit 1	0.540625
Prothrombin	0.435055397
Beta-lactamase OXA-10	0
cGMP-dependent 3',5'-cyclic phosphodiesterase	0.5
Stromelysin-1	0.444221621
Androgen receptor	0.627822857
Seminal ribonuclease	0.333333333
Coagulation factor X	0.562681056
Ribosomal protein S6 kinase alpha-2	0.364858907
Orotidine 5'-phosphate decarboxylase	0.514285714
Cannabinoid receptor 1	0.560709614
Peroxisome proliferator-activated receptor gamma	0.751041667
Acetylcholinesterase	0.46
Dual specificity tyrosine-phosphorylation-regulated kinase 2	0.455703212
Liver carboxylesterase 1	0.567661199
Dipeptidyl peptidase 8	0.556932471
3-phosphoshikimate 1-carboxyvinyltransferase	0.755555556
Serum albumin	0.548611111
Retinoic acid receptor RXR-beta	0.571790541
Epidermal growth factor receptor	0.736080508
Serum albumin	0.534722222
Urokinase-type plasminogen activator	0.581962451
Carbonic anhydrase 9	0.679042002
Death-associated protein kinase 1	0.484848485
cAMP-specific 3',5'-cyclic phosphodiesterase 4D	0.672105672
Serine/threonine-protein kinase DCLK3	0.375115634
Mitogen-activated protein kinase 14	0.734810653

PROTEIN FAMILY	AUC
Trace amine-associated receptor 1	0.666666667
Carbonic anhydrase 1	0.470534763
D(3) dopamine receptor	0.491817798
Calcium/calmodulin-dependent protein kinase type II subunit gamma	0.442226891
Beta-lactamase OXA-10	1
Nuclear receptor subfamily 1 group I member 2	0
5-hydroxytryptamine receptor 2A	0.526099655
Dual specificity protein kinase TTK	0.559498208
Peroxisome proliferator-activated receptor gamma	0.686458333
Beta-lactamase	0.236467236
Estrogen receptor	0.680843923
Vascular endothelial growth factor receptor 1	0.541463415
Estrogen receptor beta	0.696366834
Breast cancer type 1 susceptibility protein	0.125
Peptidyl-prolyl cis-trans isomerase NIMA-interacting 1	0.759259259
Tyrosine-protein phosphatase non-receptor type 1	0.601075565
Heat shock protein HSP 90-alpha	0.808962264
Methionine aminopeptidase 2	0.49847561
Beta-lactamase	0.625
Cathepsin K	0.521066947
Lactoylglutathione lyase	0.770884521
Urokinase-type plasminogen activator	0.530214457
Sialidase-2	0.833333333
Receptor-type tyrosine-protein phosphatase F	0.897727273
Triosephosphate isomerase	0.40625
Dihydroorotate dehydrogenase (quinone), mitochondrial	0.698560606
Phosphatidylinositol 4,5-bisphosphate 3-kinase catalytic subunit gamma isoform	0.640804598
Vitamin D3 receptor	0.244897959
Alcohol dehydrogenase class 4 mu/sigma chain	0.383333333
cGMP-dependent protein kinase 1	0.686827957
Cruzipain	0.988888889
Cruzipain	0.933333333
Sialidase-2	0.666666667
Chymase	0.552011096
Disintegrin and metalloproteinase domain-containing protein 17	0.569438626
RAF proto-oncogene serine/threonine-protein kinase	0.630718954
Cathepsin S	0.689090086
Tyrosine-protein kinase Lck	0.587241276
Mitogen-activated protein kinase 14	0.678838951
Fibroblast growth factor receptor 4	0.542410714
Orotidine 5'-phosphate decarboxylase	0.414285714
Purine nucleoside phosphorylase	0.588415048
Phosphatidylinositol 5-phosphate 4-kinase type-2 gamma	0.68321513
Serine/threonine-protein kinase Chk1	0.588970588
Dual specificity tyrosine-phosphorylation-regulated kinase 1B	0.478316327
Glutamate carboxypeptidase 2	0.70021645
TGF-beta receptor type-2	0.439413265
S-methyl-5'-thioadenosine phosphorylase	0.333333333
Tyrosine-protein kinase Lck	0.677282272
Beta-lactamase	0.201388889
Peptidyl-prolyl cis-trans isomerase NIMA-interacting 1	0.687109187
Retinoic acid receptor gamma	0.566800459
cAMP-specific 3',5'-cyclic phosphodiesterase 4A	0.718671679

PROTEIN FAMILY	AUC
Dihydrofolate reductase	0.582840506
Nitric oxide synthase, endothelial	0.375959079
Disintegrin and metalloproteinase domain-containing protein 17	0.554618768
Phenylethanolamine N-methyltransferase	0.687867647
Cell division protein ZipA	1
Transthyretin	0.928571429
Trypsin	0.991666667
Prolyl endopeptidase	0.240909091
Wee1-like protein kinase	0.526785714
Coagulation factor X	0.529973481
Thymidylate synthase	0.572871573
Glutamate receptor ionotropic, NMDA 1	0.869565217
Neutrophil elastase	0.582489509
Tyrosine-protein phosphatase non-receptor type 1	0.684623399
Tyrosine-protein kinase JAK2	0.589594356
Proto-oncogene tyrosine-protein kinase receptor Ret	0.561570391
Tankyrase-2	0.833333333
Cathepsin K	0.500877424
LIM domain kinase 2	0.56374269
Serine/threonine-protein kinase Chk1	0.63995098
Death-associated protein kinase 1	0.48969697
Tyrosine-protein kinase JAK2	0.640065359
Tyrosine-protein phosphatase non-receptor type 1	0.69980695
Serine/threonine-protein kinase PAK 1	0.592650104
Thymidylate synthase	0.574314574
Phosphatidylinositol 4,5-bisphosphate 3-kinase catalytic subunit gamma isoform	0.586925287
Casein kinase II subunit alpha	0.408923732
Homeodomain-interacting protein kinase 1	0.478095238
Metabotropic glutamate receptor 2	0.695924765
cGMP-specific 3',5'-cyclic phosphodiesterase	0.889162562
Cholinesterase	0.838808435
Dihydroorotate dehydrogenase (quinone), mitochondrial	0.686363636
cAMP-dependent protein kinase catalytic subunit beta	0.359698682
Glutathione S-transferase Mu 1	0.1
Beta-lactamase	0.209935897
Beta-secretase 1	0.838590802
Protein-tyrosine kinase 2-beta	0.51127451
Alcohol dehydrogenase E chain	0.302777778
Arginase-1	0.214285714
Mitogen-activated protein kinase 14	0.71213067
Prostaglandin G/H synthase 2	0.829268293
TGF-beta receptor type-2	0.548245614
Tyrosine-protein kinase Blk	0.652469136
Myosin light chain kinase, smooth muscle	0.477104377
Nuclear receptor subfamily 1 group I member 2	0
Tryptase alpha/beta-1	0.210227273
Serine/threonine-protein kinase pim-1	0.701053325
Macrophage metalloelastase	0.354516807
1-deoxy-D-xylulose 5-phosphate reductoisomerase	0.25
Glutamate receptor ionotropic, kainate 2	0.311984368
3-phosphoinositide-dependent protein kinase 1	0.349773756
Neuroendocrine convertase 2	0.5
Peptidyl-prolyl cis-trans isomerase NIMA-interacting 1	0.727032227

PROTEIN FAMILY	AUC
Tyrosine-protein phosphatase non-receptor type 1	0.673116995
Solute carrier family 28 member 3	0.8125
Peroxisome proliferator-activated receptor delta	0.648148148
Cathepsin B	0.570692884
Leukotriene A-4 hydrolase	1
Dual specificity protein kinase CLK3	0.554166667
Vasopressin V1b receptor	0.552136752
cGMP-specific 3',5'-cyclic phosphodiesterase	0.916943522
Beta-secretase 1	0.799454599
NUAK family SNF1-like kinase 2	0.460664336
Mitogen-activated protein kinase kinase kinase kinase 5	0.52966381
Cathepsin S	0.661679493
Tyrosine-protein kinase Fgr	0.529689609
Acetylcholinesterase	0.446666667
cAMP-specific 3',5'-cyclic phosphodiesterase 4B	0.886192017
5-hydroxytryptamine receptor 5A	0.550774659
Serum albumin	0.597222222
Beta-secretase 1	0.833726415
Retinoic acid receptor gamma	0.589277523
Beta-lactamase	0.213333333
Neutral alpha-glucosidase AB	0.479166667
Estrogen receptor	0.727649007
3-phosphoinositide-dependent protein kinase 1	0.552941176
Glucosylceramidase	0.747180451
Eukaryotic translation initiation factor 2-alpha kinase 1	0.450877193
Serine/threonine-protein kinase pim-1	0.671527321
E3 ubiquitin-protein ligase XIAP	0.559095634
Receptor tyrosine-protein kinase erbB-4	0.321938776
Caspase-9	0.166666667
Serine/threonine-protein kinase VRK2	0.49122807
Thymidylate synthase	0.588744589
3-phosphoinositide-dependent protein kinase 1	0.559728507
Peroxisome proliferator-activated receptor gamma	0.761024306
Thymidine phosphorylase	0.473522167
Rho-associated protein kinase 1	0.389
Carboxypeptidase A1	0.176075269
Bacterial leucyl aminopeptidase	0.212121212
Myelin-associated glycoprotein	0
Nitric oxide synthase, inducible	0.633474042
Progesterone receptor	0.502495931
Casein kinase I isoform gamma-3	0.419106317
Fibroblast growth factor receptor 2	0.629407051
Myosin light chain kinase, smooth muscle	0.405723906
3-phosphoshikimate 1-carboxyvinyltransferase	0.644444444
Trypsin	0.908333333
3-phosphoinositide-dependent protein kinase 1	0.479638009
Cathepsin K	0.494814425
Thermolysin	0.563243243
Transient receptor potential cation channel subfamily M member 6	0.387096774
Collagenase 3	0.455354037
Estrogen receptor	0.699838912
Cyclin-dependent kinase 13	0.48573975
Mitogen-activated protein kinase 1	0.68151184

PROTEIN FAMILY	AUC
Dipeptidyl peptidase 4	0.697406984
cAMP-dependent protein kinase catalytic subunit alpha	0.463421743
Thymidylate synthase	0.591625207
Matrilysin	0.339835859
TGF-beta receptor type-1	0.600632244
Integrin alpha-L	0.823170732
Glutathione S-transferase P	0.975
Acetylcholinesterase	0.54
Ribosyldihydronicotinamide dehydrogenase [quinone]	0.088888889
TRAF2 and NCK-interacting protein kinase	0.535430839
cGMP-specific 3',5'-cyclic phosphodiesterase	0.863711002
Orotidine 5'-phosphate decarboxylase	0.514285714
TRAF2 and NCK-interacting protein kinase	0.628117914
Collagenase 3	0.439701863
Prothrombin	0.655888693
Hypoxanthine-guanine phosphoribosyltransferase	0.506465517
Adenosine deaminase	0.895061728
Fibroblast growth factor 1	0.444444444
Serine/threonine-protein kinase Chk2	0.633796296
Beta-lactamase	0.201149425
Poly [ADP-ribose] polymerase 1	0.291039157
Queuine tRNA-ribosyltransferase	0.657142857
Carnitine O-acetyltransferase	0.777777778
Androgen receptor	0.647118487
Alpha-1A adrenergic receptor	0.665140208
Nitric oxide synthase, inducible	0.644279234
Insulin-like growth factor 1 receptor	0.677310924
C-C chemokine receptor type 3	0.32991453
Aldo-keto reductase family 1 member C3	0.777777778
Serine/threonine-protein kinase tousled-like 2	0.430555556
Prostaglandin G/H synthase 2	0.951219512
Epidermal growth factor receptor	0.706377119
Amine oxidase [flavin-containing] B	0.624963504
Nitric oxide synthase, endothelial	0.466041489
5-hydroxytryptamine receptor 3C	0.583333333
Multidrug resistance-associated protein 1	0.21875
Complement C1s subcomponent	0.398097826
Glutathione S-transferase Mu 1	0.4
Serum albumin	0.430555556
Thymidylate kinase	0.701388889
Inositol-trisphosphate 3-kinase A	0.5
Disintegrin and metalloproteinase domain-containing protein 10	0.45
Myosin-IIIb	0.461469534
6,7-dimethyl-8-ribityllumazine synthase	0.428571429
Mitogen-activated protein kinase 1	0.685701275
Bacterial leucyl aminopeptidase	0.303030303
Serine/threonine-protein kinase MARK2	0.532936508
Serine/threonine-protein kinase 10	0.523148148
Kallikrein-7	0.5
MAP kinase-interacting serine/threonine-protein kinase 2	0.445011338
Histamine N-methyltransferase	0.833333333
Thymidylate synthase	0.595538721
Mitogen-activated protein kinase 14	0.664794007

PROTEIN FAMILY	AUC
Chaperone activity of bc1 complex-like, mitochondrial	0.522270115
Cathepsin S	0.699253056
Dehydrosqualene synthase	0.833333333
N(G),N(G)-dimethylarginine dimethylaminohydrolase 1	0.361111111
Estrogen receptor beta	0.625551383
Cocaine esterase	0.652457002
Mast/stem cell growth factor receptor Kit	0.684041037
Estrogen receptor beta	0.702715188
Prothrombin	0.438869241
Adenosine deaminase	0.741769547
Mu-type opioid receptor	0.601291396
Cruzipain	0.922222222
Thymidylate synthase	0.649212431
Metabotropic glutamate receptor 2	0.769592476
Angiotensin-converting enzyme 2	0.793103448
C5a anaphylatoxin chemotactic receptor 1	0.909814324
Urokinase-type plasminogen activator	0.534737129
WD repeat-containing protein 5	0.793478261
Gamma-aminobutyric acid receptor subunit alpha-2	0.547393048
STE20-like serine/threonine-protein kinase	0.591046582
3-dehydroquinate dehydratase	0.886666667
Inositol-trisphosphate 3-kinase B	0.5
Serine/threonine-protein kinase PAK 4	0.670957096
Bromodomain-containing protein 4	0.25
Receptor-type tyrosine-protein phosphatase beta	0.75
Death-associated protein kinase 1	0.444848485
Gastrin-releasing peptide receptor	0.728985507
Chymotrypsinogen A	0.657621951
Fibroblast growth factor receptor 1	0.689115646
Coagulation factor VII	0.662045319
Cationic trypsin	0.661654135
Cathepsin K	0.527121172
Mitogen-activated protein kinase 14	0.616607713
Thymidylate synthase	0.559701493
Purine nucleoside phosphorylase	0.410714286
Sodium channel protein type 9 subunit alpha	0.467181467
Glycogen phosphorylase, muscle form	0.285714286
Receptor-type tyrosine-protein phosphatase epsilon	0.3
Carboxypeptidase A1	0.239023297
Growth hormone secretagogue receptor type 1	0.496277916
Estradiol 17-beta-dehydrogenase 1	0.333333333
Ribonuclease pancreatic	0.338509317
RAF proto-oncogene serine/threonine-protein kinase	0.659041394
Insulin receptor	0.708584842
Vitamin D3 receptor	0.244897959
Mu-type opioid receptor	0.540205334
Cationic trypsin	0.629301619
Protein kinase C zeta type	0.9
Estrogen receptor	0.61832155
Peptidyl-prolyl cis-trans isomerase A	0.903153153
Aldose reductase	0.416666667
Dipeptidyl peptidase 4	0.734886493
Thyroid hormone receptor alpha	0.127205882

PROTEIN FAMILY	AUC
Macrophage metalloelastase	0.372242647
Mitogen-activated protein kinase 8	0.553571429
Cruzipain	0.933333333
Ankyrin repeat and protein kinase domain-containing protein 1	0.438292964
Myosin light chain kinase, smooth muscle	0.464285714
Dual specificity protein phosphatase 3	0.633928571
cAMP-specific 3',5'-cyclic phosphodiesterase 4A	0.793859649
Neutrophil collagenase	0.644432164
Peroxisome proliferator-activated receptor alpha	0.263646922
Vitamin D3 receptor	0.224489796
Cyclin-dependent kinase 9	0.416871921
Caspase-3	0.935606061
Glutathione S-transferase P	0.7
Fatty acid-binding protein, adipocyte	0.805921053
Estrogen receptor	0.600601844
Tissue-type plasminogen activator	0.430810811
Aldose reductase	0.555555556
cAMP-dependent protein kinase catalytic subunit alpha	0.397551734
Serine/threonine-protein kinase Chk1	0.612322515
Mitogen-activated protein kinase kinase kinase 3	0.448568399
Cholinesterase	0.794131098
cGMP-specific 3',5'-cyclic phosphodiesterase	0.867816092
Serine/threonine-protein kinase pim-1	0.727452271
ALK tyrosine kinase receptor	0.648826979
Tryptase gamma	0.397058824
Dual specificity mitogen-activated protein kinase kinase 6	0.557377049
Transthyretin	0.821428571
Estrogen receptor beta	0.694310324
Cyclin-dependent kinase 13	0.517825312
Serine/threonine-protein kinase receptor R3	0.716757741
Glutathione S-transferase A1	0.666666667
Methionine aminopeptidase 1	0.71557971
Aminopeptidase N	0.550239234
Phosphatidylinositol 4,5-bisphosphate 3-kinase catalytic subunit gamma isoform	0.659482759
Phosphorylase b kinase gamma catalytic chain, liver/testis isoform	0.500725689
Histamine H3 receptor	0.650176222
Renin	0.763660224
Androgen receptor	0.408593215
Serine/threonine-protein kinase PLK3	0.532563025
Lethal factor	0.530594406
Beta-1,4-galactosyltransferase 1	1
Serine/threonine-protein kinase PLK1	0.387318564
Insulin receptor	0.678403756
Bacterial leucyl aminopeptidase	0.181818182
Integrin beta-2	0.654166667
MAP kinase-activated protein kinase 2	0.759393939
Phosphatidylinositol 4,5-bisphosphate 3-kinase catalytic subunit gamma isoform	0.61566092
cAMP-specific 3',5'-cyclic phosphodiesterase 4B	0.853020496
Mitogen-activated protein kinase 14	0.670741434
Tyrosine-protein kinase HCK	0.590697674
Transthyretin	0.785714286
Cyclin-dependent kinase-like 5	0.647169811
Fatty acid-binding protein, adipocyte	0.832236842

PROTEIN FAMILY	AUC
Mitogen-activated protein kinase 15	0.624019608
Ephrin type-A receptor 4	0.434901961
Tyrosine-protein phosphatase non-receptor type 1	0.768967948
Protein kinase C beta type	0.483636364
Peptidyl-prolyl cis-trans isomerase A	0.90990991
Queuine tRNA-ribosyltransferase	0.714285714
Cyclin-dependent kinase 2	0.345929283
Nuclear receptor corepressor 2	0.666666667
Amiloride-sensitive amine oxidase [copper-containing]	0.111111111
Urokinase-type plasminogen activator	0.536863552
6,7-dimethyl-8-ribityllumazine synthase	0.397959184
Beta-secretase 1	0.775574882
Orotidine 5'-phosphate decarboxylase	0.442857143
Urokinase-type plasminogen activator	0.561990764
Death-associated protein kinase 1	0.451515152
Phosphatidylinositol 4,5-bisphosphate 3-kinase catalytic subunit gamma isoform	0.686781609
Alpha-1A adrenergic receptor	0.286917684
Myosin light chain kinase, smooth muscle	0.438311688
Nitric oxide synthase, endothelial	0.385620915
Retinoic acid receptor alpha	0.471118421
Dihydrofolate reductase	0.551360204
Chymotrypsinogen A	0.727951389
Thymidine phosphorylase	0.573891626
Mitogen-activated protein kinase 10	0.513474026
Serum albumin	0.451388889
Urokinase-type plasminogen activator	0.56576547
Receptor tyrosine-protein kinase erbB-3	0.479166667
Receptor-type tyrosine-protein phosphatase beta	0.5875
Mitogen-activated protein kinase 15	0.471568627
Beta-lactamase OXA-10	1
Serine/threonine-protein kinase PLK1	0.461420932
Leucine-rich repeat serine/threonine-protein kinase 2	0.384913536
Serine/threonine-protein kinase TAO1	0.562334218
Serine/threonine-protein kinase PAK 2	0.589341693
Cathepsin K	0.530525577
Histamine H3 receptor	0.513704032
Renin	0.729048295
Caspase-3	0.916666667
Estradiol 17-beta-dehydrogenase 1	0.333333333
3-dehydroquinate dehydratase	0.886666667
Androgen receptor	0.624401212
Phosphatidylinositol 4,5-bisphosphate 3-kinase catalytic subunit gamma isoform	0.716954023
5-hydroxytryptamine receptor 7	0.434248977
Urokinase-type plasminogen activator	0.520350892
Serine/threonine-protein kinase 32C	0.03030303
3-phosphoinositide-dependent protein kinase 1	0.368269231
Macrophage migration inhibitory factor	0.6875
Heat shock protein HSP 90-alpha	0.859669811
Disintegrin and metalloproteinase domain-containing protein 17	0.628718056
Myosin light chain kinase, smooth muscle	0.408754209
Beta-secretase 1	0.86379717
Papain	0.470812875
Phosphatidylinositol 4-kinase type 2-alpha	0.592

PROTEIN FAMILY	AUC
Renin	0.740885417
Serine/threonine-protein kinase MARK2	0.695767196
Queuine tRNA-ribosyltransferase	0.671428571
Phenylethanolamine N-methyltransferase	0.760661765
Renin	0.744318182
Pteridine reductase 1	0.766233766
L-lactate dehydrogenase B chain	0.645833333
Estrogen receptor	0.613940959
Cathepsin B	0.600187266
Arginase-1	0.214285714
Serine/threonine-protein kinase MARK1	0.629310345
Proto-oncogene tyrosine-protein kinase ROS	0.54887218
Fatty acid-binding protein, adipocyte	0.743421053
Serine/threonine-protein kinase B-raf	0.628295068
cAMP-dependent protein kinase catalytic subunit alpha	0.430778199
Dual specificity protein kinase CLK2	0.393333333
Cholinesterase	0.844308943
Wee1-like protein kinase	0.560714286
Alcohol dehydrogenase E chain	0.283333333
cAMP-dependent protein kinase catalytic subunit alpha	0.466190615
Coagulation factor XI	0.849673203
Angiotensin-converting enzyme	0.55638587
Dipeptidyl peptidase 4	0.679573792
Tyrosine-protein phosphatase non-receptor type 1	0.663724949
Serine/threonine-protein kinase Chk1	0.635539216
Proto-oncogene tyrosine-protein kinase Src	0.618852259
Tyrosine-protein kinase Lck	0.620787921
Angiotensin-converting enzyme	0.6015625
72 kDa type IV collagenase	0.740267437
Sodium/glucose cotransporter 1	0.596590909
Cathepsin B	0.552902622
Protein kinase C beta type	0.489090909
cAMP-dependent protein kinase catalytic subunit alpha	0.599096473
Lysine-specific histone demethylase 1A	0.200657895
Tyrosine-protein kinase ABL1	0.576109198
Glycogen phosphorylase, liver form	0
Prothrombin	0.452870579
Phosphatidylinositol 4,5-bisphosphate 3-kinase catalytic subunit beta isoform	0.756355932
Serine/threonine-protein kinase 17B	0.587826797
Histamine H4 receptor	0.559350775
Glucosylceramidase	0.76174812
Transthyretin	0.857142857
Beta-lactamase	0.189285714
Coagulation factor X	0.544917352
Corticosteroid 11-beta-dehydrogenase isozyme 1	0.620879121
Serum albumin	0.493055556
Prostasin	0.836956522
Estrogen receptor	0.742303562
Ribonuclease pancreatic	0.400621118
Ribonuclease pancreatic	0.397515528
Leukocyte tyrosine kinase receptor	0.51787234
Coagulation factor VII	0.758397933
Somatostatin receptor type 1	0.577945402

PROTEIN FAMILY	AUC
Tyrosine-protein kinase ITK/TSK	0.674945215
Liver carboxylesterase 1	0.373538839
3-phosphoinositide-dependent protein kinase 1	0.38959276
cAMP-dependent protein kinase catalytic subunit alpha	0.514479905
Angiotensin-converting enzyme	0.558423913
Nitric oxide synthase, inducible	0.636183216
Beta-secretase 1	0.873304835
Estradiol 17-beta-dehydrogenase 1	0.666666667
Prothrombin	0.457238827
Cathepsin B	0.562911163
Fibroblast growth factor receptor 1	0.621768707
Cyclin-dependent kinase 15	0.359477124
Peptidyl-prolyl cis-trans isomerase A	0.92454955
Beta-secretase 1	0.807340802
Leucine-rich repeat serine/threonine-protein kinase 2	0.49504662
Cationic trypsin	0.638178571
Cathepsin L1	0.667578659
Insulin-like growth factor-binding protein 3	0.974358974
Serum albumin	0.534722222
Adenosine receptor A2b	0.486580882
Activin receptor type-1B	0.566220238
Serine/threonine-protein kinase MARK2	0.618253968
Beta-lactamase	0.206790123
Serine/threonine-protein kinase NLK	0.645285088
Arginase-1	0.328571429
D(1A) dopamine receptor	0.54579648
Purine nucleoside phosphorylase	0.553571429
Cationic trypsin	0.635551948
Carbonic anhydrase 1	0.705435598
Beta-1,4-galactosyltransferase 1	1
Serine/threonine-protein kinase MRCK alpha	0.505464481
Transient receptor potential cation channel subfamily M member 6	0.491803279
TGF-beta receptor type-2	0.493734336
Heat shock protein HSP 90-alpha	0.830188679
Proto-oncogene tyrosine-protein kinase Src	0.543907204
Sodium channel protein type 9 subunit alpha	0.027777778
Acetylcholinesterase	1
Collagenase 3	0.5067
Alcohol dehydrogenase E chain	0.305555556
Carboxypeptidase A1	0.27003643
Serine/threonine-protein kinase PLK1	0.4289534
Suppressor of tumorigenicity 14 protein	0.397798742
Nitric oxide synthase, endothelial	0.393719807
Renin	0.746212121
Lysozyme	0.981481481
RAC-beta serine/threonine-protein kinase	0.504723666
cAMP-dependent protein kinase catalytic subunit alpha	0.460798601
SHC-transforming protein 1	0.15
Mitogen-activated protein kinase 11	0.613802083
S-methyl-5'-thioadenosine phosphorylase	0.115384615
Serine/threonine-protein kinase VRK2	0.338181818
Receptor-interacting serine/threonine-protein kinase 4	0.475714286
Coagulation factor XI	0.359477124

PROTEIN FAMILY	AUC
Proto-oncogene tyrosine-protein kinase Src	0.488156288
S-adenosylmethionine decarboxylase proenzyme	0.645454545
cAMP-dependent protein kinase catalytic subunit alpha	0.440979306
3-phosphoinositide-dependent protein kinase 1	0.415900735
Myotonin-protein kinase	0.465714286
Tyrosine-protein kinase ABL1	0.561988903
Serine/threonine-protein kinase 32B	0.648275862
Thymidylate synthase	0.532338308
Dihydroorotate dehydrogenase (quinone), mitochondrial	0.698636364
Glycogen synthase kinase-3 beta	0.577903996
Beta-1,4-galactosyltransferase 1	1
Calcium/calmodulin-dependent protein kinase type II subunit beta	0.465449804
Seminal ribonuclease	0.666666667
Receptor-type tyrosine-protein phosphatase beta	0.725
Nitric oxide synthase, inducible	0.431319963
Cyclin-dependent kinase 2	0.363690185
3-dehydroquinate dehydratase	0.944444444
Ribonuclease pancreatic	0.375776398
Cruzipain	0.977777778
Cathepsin G	0.396258503
Cationic trypsin	0.639278628
Serum albumin	0.3125
Cathepsin B	0.586142322
Dipeptidyl peptidase 4	0.741642243
Prothrombin	0.454484693
Tyrosine-protein phosphatase non-receptor type 2	0.444394213
Glutamate carboxypeptidase 2	0.67965368
Chymotrypsinogen A	0.730479452
Cationic trypsin	0.616177721
cAMP-dependent protein kinase catalytic subunit alpha	0.60769455
Estrogen receptor beta	0.767212089
UDP-glucuronosyltransferase 2B7	1
Collagenase 3	0.460770186
D(1B) dopamine receptor	0.482116788
Fatty acid-binding protein, adipocyte	0.838815789
Carboxypeptidase A1	0.182123656
Peroxisome proliferator-activated receptor delta	0.846560847
Histamine N-methyltransferase	0.166666667
Cyclin-dependent kinase 2	0.376061571
Gamma-aminobutyric acid receptor subunit beta-1	0.422222222
Glutamate carboxypeptidase 2	0.485930736
Aldose reductase	0.625
Mitogen-activated protein kinase 1	0.674225865
Phenylethanolamine N-methyltransferase	0.750919118
Macrophage migration inhibitory factor	1
Peroxisome proliferator-activated receptor delta	0.552910053
Coagulation factor X	0.55360838
cAMP-dependent protein kinase catalytic subunit alpha	0.502040222
M1 family aminopeptidase	0
Cathepsin S	0.684155727
Mitogen-activated protein kinase 14	0.692970592
Chymotrypsinogen A	0.730993151
cAMP-specific 3',5'-cyclic phosphodiesterase 4A	0.776315789

PROTEIN FAMILY	AUC
Leukotriene A-4 hydrolase	1
Serine/threonine-protein kinase 3	0.641737892
Serine/threonine-protein kinase pim-1	0.652304147
Cyclin-dependent kinase 9	0.398929845
Tyrosine-protein kinase Tec	0.57
Ephrin type-B receptor 4	0.478773585
Chaperone activity of bc1 complex-like, mitochondrial	0.438218391
Bromodomain-containing protein 4	0.375
Activated CDC42 kinase 1	0.439694292
TRAF2 and NCK-interacting protein kinase	0.561184939
Stromelysin-1	0.431069863
Arginase-1	0.328571429
Ephrin type-A receptor 3	0.544507576
Transthyretin	0.714285714
Glycogen synthase kinase-3 beta	0.580887553
Transthyretin	1
NADPH oxidase 4	0.37244898
cAMP-dependent protein kinase catalytic subunit alpha	0.384581755
Alpha-1D adrenergic receptor	0.766500316
Purine nucleoside phosphorylase	0.398809524
Phosphatidylinositol 4-kinase alpha	0.5168
Serine/threonine-protein kinase pim-1	0.674786043
Cathepsin L1	0.626803466
Tyrosine-protein kinase ITK/TSK	0.652118335
Estradiol 17-beta-dehydrogenase 1	0.666666667
Serine/threonine-protein kinase pim-1	0.685549704
Myosin-IIIa	0.50273224
Leukotriene A-4 hydrolase	1
Phenylethanolamine N-methyltransferase	0.722794118
Phenylethanolamine N-methyltransferase	0.676194853
Thymidylate synthase	0.67027417
Carboxypeptidase A1	0.21953405
Nitric oxide synthase, endothelial	0.40991759
Tyrosine-protein kinase JAK2	0.610875955
C-C chemokine receptor type 3	0.502564103
Proto-oncogene tyrosine-protein kinase Src	0.690573871
Acetylcholinesterase	0.406666667
Leukotriene A-4 hydrolase	1
Tyrosine-protein kinase BTK	0.492445055
Androgen receptor	0.662210382
Macrophage colony-stimulating factor 1 receptor	0.520551802
Glycogen synthase kinase-3 alpha	0.469230769
Mitogen-activated protein kinase 10	0.506114719
Proto-oncogene tyrosine-protein kinase ROS	0.619047619
Serine/threonine-protein kinase pim-1	0.701909151
Trypsin	0.791666667
Mitogen-activated protein kinase kinase kinase 19	0.572228443
Trifunctional purine biosynthetic protein adenosine-3	0.697368421
Lactoylglutathione lyase	0.631449631
Adenosylhomocysteinase	0.293908228
Ephrin type-A receptor 3	0.412962963
Nitric oxide synthase, inducible	0.638167843
Thymidylate synthase	0.598258706

PROTEIN FAMILY	AUC
Peptidyl-prolyl cis-trans isomerase A	0.904842342
Dihydrofolate reductase	0.529478664
Excitatory amino acid transporter 2	0.394230769
Farnesyl pyrophosphate synthase	0.433333333
Heat shock protein HSP 90-alpha	0.877358491
Maltase-glucoamylase, intestinal	0.133333333
Disintegrin and metalloproteinase domain-containing protein 17	0.6359447
5-hydroxytryptamine receptor 6	0.519969664
Serine/threonine-protein kinase PLK1	0.460275019
NT-3 growth factor receptor	0.4715
Calcium/calmodulin-dependent protein kinase type II subunit gamma	0.49947479
Leucine-rich repeat serine/threonine-protein kinase 2	0.52444842
Multidrug resistance-associated protein 1	0.96875
Proto-oncogene tyrosine-protein kinase Src	0.599438339
Thymidylate kinase	0.741071429
Macrophage metalloelastase	0.400735294
Thermolysin	0.535135135
L-lactate dehydrogenase A chain	0.59375
Peroxisome proliferator-activated receptor delta	0.791005291
Serine/threonine-protein kinase B-raf	0.625974026
Myosin light chain kinase, smooth muscle	0.455555556
Cationic trypsin	0.656852324
Fatty-acid amide hydrolase 1	0.567235677
Peptidyl-prolyl cis-trans isomerase NIMA-interacting 1	0.750601251
Transient receptor potential cation channel subfamily V member 1	0.79921875
3-dehydroquinate dehydratase	0.944444444
Purine nucleoside phosphorylase	0.267857143
Alpha-1B adrenergic receptor	0.761392754
Stromelysin-1	0.437272642
Beta-secretase 1	0.807488208
Collagenase 3	0.43736646
Ribosyldihydronicotinamide dehydrogenase [quinone]	0.1
Chymotrypsinogen A	0.62859589
Glutathione S-transferase P	0.75
Plasmepsin-2	0.778737183
Prostaglandin G/H synthase 1	0.6
Beta-lactamase	0.4
Macrophage migration inhibitory factor	1
Cathepsin B	0.66964517
Nitric oxide synthase, endothelial	0.42895709
Tyrosine-protein kinase Lyn	0.624255319
Serine/threonine-protein kinase PLK4	0.667561762
Heat shock protein HSP 90-alpha	0.83254717
Insulin-like growth factor 1 receptor	0.592016807
Purine nucleoside phosphorylase	0.535675676
Hepatocyte growth factor receptor	0.493769051
Insulin receptor	0.610663984
Phenylethanolamine N-methyltransferase	0.523161765
Homeodomain-interacting protein kinase 1	0.419146825
Peroxisome proliferator-activated receptor gamma	0.750347222
Insulin-like growth factor 1 receptor	0.698739496
Coagulation factor X	0.56085424
Glutamate carboxypeptidase 2	0.568181818

PROTEIN FAMILY	AUC
Chymotrypsinogen A	0.734589041
Homeodomain-interacting protein kinase 1	0.356150794
Coagulation factor VII	0.809878752
Serum albumin	0.409722222
Caspase-8	0.846153846
5-hydroxytryptamine receptor 3A	0.661951754
Mitogen-activated protein kinase 10	0.548917749
Coagulation factor X	0.560505324
Integrin alpha-L	0.731707317
Coagulation factor X	0.559986066
Farnesyl pyrophosphate synthase	0.6625
Cyclin-dependent kinase 9	0.38882283
Receptor-type tyrosine-protein phosphatase F	0.875
Trypsin-3	0.974576271
Transthyretin	0.928571429
Acetylcholinesterase	1
Coagulation factor X	0.560736786
Galectin-7	0.614942529
Beta-lactamase	0.243076923
Thyroid hormone receptor alpha	0.125735294
Nitric oxide synthase, inducible	0.63734879
Serine/threonine-protein kinase PLK1	0.451489687
cAMP-dependent protein kinase catalytic subunit alpha	0.448703002
Suppressor of tumorigenicity 14 protein	0.377358491
Macrophage metalloelastase	0.428702731
Lethal factor	0.524475524
Ribonuclease pancreatic	0.301242236
Membrane-associated tyrosine- and threonine-specific cdc2-inhibitory kinase	0.209230769
Galectin-9	0.916666667
Serum albumin	0.4375
Tryptase alpha/beta-1	0.223262032
M-phase inducer phosphatase 2	0.4
Queuine tRNA-ribosyltransferase	0.671428571
Alcohol dehydrogenase E chain	0.305555556
Nuclear receptor subfamily 1 group I member 2	0.25
Microtubule-associated serine/threonine-protein kinase 1	0.391975309
Nitric oxide synthase, inducible	0.429221082
Prothrombin	0.434754627
Cationic trypsin	0.645892151
MAP/microtubule affinity-regulating kinase 3	0.608391608
Collagenase 3	0.449142857
Orotidine 5'-phosphate decarboxylase	0.442857143
Phosphatidylinositol 4,5-bisphosphate 3-kinase catalytic subunit gamma isoform	0.58908046
Fibroblast growth factor receptor 1	0.587053571
E3 ubiquitin-protein ligase XIAP	0.616839917
Dual specificity mitogen-activated protein kinase kinase 5	0.55955956
Bromodomain-containing protein 4	0.5
Ephrin type-A receptor 7	0.577830189
Tyrosine-protein kinase JAK2	0.616274929
Phenylethanolamine N-methyltransferase	0.746507353
TRAF2 and NCK-interacting protein kinase	0.563492063
Protein kinase C alpha type	0.693609561
Cyclin-dependent kinase 4	0.485712005

PROTEIN FAMILY	AUC
Galectin-9	0.903703704
SRSF protein kinase 2	0.738729508
Leukotriene A-4 hydrolase	1
Trypanothione reductase	0.757173913
Myosin light chain kinase, smooth muscle	0.507544582
Heat shock protein HSP 90-alpha	0.872641509
MAP/microtubule affinity-regulating kinase 3	0.572261072
Nitric oxide synthase, endothelial	0.423415743
Tyrosine-protein phosphatase non-receptor type 1	0.710823068
Estrogen receptor	0.638972168
Serine/threonine-protein kinase LATS1	0.588474026
Dihydrofolate reductase	0.639705213
Mitogen-activated protein kinase 14	0.525090165
Interstitial collagenase	0.431587636
5-hydroxytryptamine receptor 3D	0.608333333
Adenosine deaminase	0.778806584
Peptidyl-prolyl cis-trans isomerase NIMA-interacting 1	0.743386243
Serine/threonine-protein kinase Chk2	0.631481481
Beta-secretase 1	0.829967571
Putative peripheral benzodiazepine receptor-related protein	0.266666667
Ribonuclease pancreatic	0.363354037
G protein-coupled receptor kinase 7	0.497607656
Acetylcholinesterase	0.486666667
Beta-1,4-galactosyltransferase 1	1
Beta-lactamase	0.201149425
Baculoviral IAP repeat-containing protein 2	0.78
Multidrug resistance-associated protein 1	0.65625
Queuine tRNA-ribosyltransferase	0.685714286
Serine/threonine-protein kinase PAK 1	0.623706004
Beta-lactamase	0.206730769
Androgen receptor	0.582803793
Peroxisome proliferator-activated receptor gamma	0.705815972
Estrogen receptor	0.623735905
Epithelial discoidin domain-containing receptor 1	0.720494418
Orotidine 5'-phosphate decarboxylase	0.478571429
Dipeptidyl peptidase 4	0.706149719
S-adenosylmethionine decarboxylase proenzyme	0.536363636
Acetylcholinesterase	1
Nitric oxide synthase, endothelial	0.409775504
Tyrosine-protein kinase Lck	0.663483652
Cathepsin B	0.58988764
Cationic trypsin	0.644742459
Lethal(3)malignant brain tumor-like protein 1	0.166666667
Prothrombin	0.448585736
Aurora kinase A	0.559281046
5'-AMP-activated protein kinase catalytic subunit alpha-2	0.642633229
Corticotropin-releasing factor receptor 2	nan
Dehydrosqualene synthase	0.794117647
Tyrosine-protein phosphatase non-receptor type 11	0.534965035
Dual specificity mitogen-activated protein kinase kinase 1	0.392857143
Cathepsin L1	0.615211397
Potassium voltage-gated channel subfamily H member 2	0.64287273
Androgen receptor	0.556921498

PROTEIN FAMILY	AUC
Urokinase-type plasminogen activator	0.612106421
Vascular endothelial growth factor receptor 2	0.492716296
Phosphatidylinositol 4,5-bisphosphate 3-kinase catalytic subunit gamma isoform	0.624281609
Serine/threonine-protein kinase PLK1	0.475935829
Kappa-type opioid receptor	0.582898392
Peptidyl-prolyl cis-trans isomerase A	0.936373874
Serum albumin	0.423611111
Cationic trypsin	0.662758176
Mitogen-activated protein kinase 14	0.596632681
cAMP-dependent protein kinase catalytic subunit alpha	0.421014282
Serine/threonine-protein kinase pim-1	0.720013167
Disintegrin and metalloproteinase domain-containing protein 17	0.525450356
Acidic mammalian chitinase	1
Methionine aminopeptidase 2	0.518038618
Caspase-3	0.933080808
Lethal factor	0.58041958
5-hydroxytryptamine receptor 4	0.580140485
Multidrug resistance protein 1	0.650735294
Tyrosine-protein phosphatase non-receptor type 1	0.75116443
Thyrotropin-releasing hormone receptor	0.208333333
Heat shock protein HSP 90-alpha	0.896226415
Serine/threonine-protein kinase PAK 4	0.665441176
Beta-lactamase	0.413333333
Seprase	0.26744186
RAC-beta serine/threonine-protein kinase	0.507085498
Peroxisomal N(1)-acetyl-spermine/spermidine oxidase	1
Peroxisome proliferator-activated receptor gamma	0.82421875
Mitogen-activated protein kinase 7	0.321052632
Growth factor receptor-bound protein 2	0.484848485
3-dehydroquinate dehydratase	0.314814815
Macrophage metalloelastase	0.333902311
Ribonuclease pancreatic	0.357142857
Calcium/calmodulin-dependent protein kinase type II subunit beta	0.488917862
Casein kinase I isoform gamma-2	0.520935961
Receptor-type tyrosine-protein phosphatase F	0.897727273
Urokinase-type plasminogen activator	0.539429641
Breast cancer type 1 susceptibility protein	0.5
Uridine 5'-monophosphate synthase	0.275
Aurora kinase A	0.571739837
Carboxypeptidase A1	0.173611111
Tankyrase-2	1
Carbonic anhydrase 5B, mitochondrial	0.751967157
Death-associated protein kinase 1	0.646666667
Serine/threonine-protein kinase SBK1	0.480376766
Dual specificity tyrosine-phosphorylation-regulated kinase 2	0.492801772
Coagulation factor XI	0.37254902
Alpha-1D adrenergic receptor	0.712832457
Acetylcholinesterase	0.46
Collagenase 3	0.389863354
Acetylcholinesterase	0.46
Aldose reductase	0.5
Leukotriene A-4 hydrolase	0.833333333
Protein kinase C eta type	0.299829932

PROTEIN FAMILY	AUC
Urokinase-type plasminogen activator	0.561283186
Somatostatin receptor type 2	0.761312421
Papain	0.482951446
Dual specificity mitogen-activated protein kinase kinase 1	0.379440665
Focal adhesion kinase 1	0.37037037
Cathepsin K	0.553610599
Inosine-5'-monophosphate dehydrogenase 1	0.388888889
Proto-oncogene tyrosine-protein kinase Src	0.571428571
Serine/threonine-protein kinase MRCK alpha	0.392759563
Disks large homolog 4	0.593440428
Pteridine reductase 1	0.727272727
Ribonuclease pancreatic	0.375776398
Heat shock protein HSP 90-beta	0.8
Uridine 5'-monophosphate synthase	0.45
Hepatocyte growth factor receptor	0.630109232
Tyrosine-protein kinase Lck	0.667683232
Glycogen phosphorylase, muscle form	0.285714286
BMP-2-inducible protein kinase	0.422939068
Chymotrypsinogen A	0.708390411
cAMP-dependent protein kinase catalytic subunit PRKX	0.521464646
Aurora kinase A	0.569268293
Heat shock protein HSP 90-alpha	0.872641509
Prostaglandin E2 receptor EP4 subtype	0.696336047
Proto-oncogene tyrosine-protein kinase Src	0.628742369
Gastricsin	0.351851852
Insulin-like growth factor 1 receptor	0.588235294
Serine/threonine-protein kinase pim-3	0.713577422
Methionine aminopeptidase 2	0.541666667
Mitogen-activated protein kinase 14	0.698241781
Serine/threonine-protein kinase pim-1	0.702699144
Steroid hormone receptor ERR1	1
Carbonic anhydrase 2	0.766079346
5-hydroxytryptamine receptor 2A	0.475731712
Serine/threonine-protein kinase BRSK2	0.442622951
Beta-3 adrenergic receptor	0.773096886
Thymidylate synthase	0.684704185
Serine/threonine-protein kinase MRCK alpha	0.567204301
WD repeat-containing protein 5	0.413043478
STE20-like serine/threonine-protein kinase	0.569262782
Abelson tyrosine-protein kinase 2	0.623754153
Mitogen-activated protein kinase kinase kinase 12	0.460769231
Peroxisome proliferator-activated receptor delta	0.46031746
Hypoxanthine-guanine phosphoribosyltransferase	0.520474138
Epithelial discoidin domain-containing receptor 1	0.472488038
Cyclin-dependent kinase-like 1	0.358757062
MAP kinase-interacting serine/threonine-protein kinase 2	0.524722222
Purine nucleoside phosphorylase	0.446428571
Poly [ADP-ribose] polymerase 3	1
Insulin receptor-related protein	0.405906593
Estrogen receptor beta	0.738048402
Transthyretin	0.946428571
Monocarboxylate transporter 1	0.556818182
Ribonuclease pancreatic	0.313664596

PROTEIN FAMILY	AUC
Nitric oxide synthase, endothelial	0.428388747
Cyclin-dependent kinase 4	0.469117647
Prothrombin	0.623489279
Proto-oncogene tyrosine-protein kinase Src	0.560634921
Collagenase 3	0.438136646
Tyrosine-protein kinase JAK1	0.589559387
Serum albumin	0.555555556
Serine/threonine-protein kinase Nek2	0.466923077
Phosphatidylinositol 4-kinase alpha	0.6112
Macrophage colony-stimulating factor 1 receptor	0.596249311
Calcium/calmodulin-dependent protein kinase type II subunit beta	0.548076923
Cathepsin K	0.50720365
Chymotrypsinogen A	0.650860555
Plasminogen	0.348870389
Caspase-3	0.939393939
Cyclin-dependent kinase 2	0.339498612
Cyclin-dependent kinase 2	0.49640699
Myosin light chain kinase, smooth muscle	0.530639731
Peptidyl-prolyl cis-trans isomerase A	0.927364865
Lymphokine-activated killer T-cell-originated protein kinase	0
Myosin-IIIb	0.508196721
Death-associated protein kinase 1	0.495679012
Ephrin type-A receptor 8	0.469148936
Alcohol dehydrogenase E chain	0.294444444
Proto-oncogene tyrosine-protein kinase Src	0.616507937
Prolyl endopeptidase	0.244318182
Prothrombin	0.453446203
Neutrophil collagenase	0.630837248
Tyrosine-protein kinase Mer	0.609693878
Glutathione S-transferase A1	0.733333333
Peroxisome proliferator-activated receptor delta	0.682539683
Histone deacetylase-like amidohydrolase	0
Retinoic acid receptor gamma	0.568233945
Fatty acid-binding protein, adipocyte	0.792763158
Tyrosine-protein kinase Fes/Fps	0.4375
Methionine aminopeptidase 2	0.532520325
3-phosphoinositide-dependent protein kinase 1	0.374660633
Cationic trypsin	0.652597403
Cathepsin S	0.666364871
Serine/threonine-protein kinase mTOR	0.690813253
Chymotrypsinogen A	0.651883562
Serine/threonine-protein kinase pim-1	0.696675444
Mitogen-activated protein kinase 15	0.526
Transthyretin	0.821428571
1-deoxy-D-xylulose 5-phosphate reductoisomerase	0.291666667
AP2-associated protein kinase 1	0.472515856
Peroxisome proliferator-activated receptor gamma	0.700607639
Protein kinase C alpha type	0.601975097
MAP/microtubule affinity-regulating kinase 4	0.596045198
Peptidyl-prolyl cis-trans isomerase A	0.909346847
cAMP-specific 3',5'-cyclic phosphodiesterase 4D	0.628852629
Signal transducer and activator of transcription 3	0.243589744
Serine/threonine-protein kinase pim-1	0.665339039

PROTEIN FAMILY	AUC
Arginase-1	0.328571429
Insulin receptor	0.655264923
Gastricsin	0.462962963
Orotidine 5'-phosphate decarboxylase	0.514285714
Tyrosine-protein kinase JAK2	0.578012934
Multidrug resistance-associated protein 1	0.15625
Cyclin-dependent kinase-like 2	0.549872123
Beta-secretase 1	0.821270637
Glutathione reductase, mitochondrial	0.555555556
Serine/threonine-protein phosphatase 2B catalytic subunit alpha isoform	0.666666667
Serine/threonine-protein kinase TBK1	0.479738562
Caspase-3	0.917929293
Lanosterol 14-alpha demethylase	0.25
Serine/threonine-protein kinase N1	0.524417314
Ephrin type-A receptor 5	0.490442055
Coagulation factor X	0.519846359
D(2) dopamine receptor	0.570391911
Prolyl endopeptidase	0.277272727
Retinoic acid receptor gamma	0.524942661
Motilin receptor	0.372093023
3-phosphoinositide-dependent protein kinase 1	0.355656109
Death-associated protein kinase 3	0.620265152
Cytoplasmic tyrosine-protein kinase BMX	0.638755981
Alcohol dehydrogenase E chain	0.216666667
Carbonic anhydrase 2	0.761365124
Serine/threonine-protein kinase Chk1	0.640931373
Cyclin-dependent kinase 13	0.393939394
Serine/threonine-protein kinase PAK 7	0.593117409
cGMP-inhibited 3',5'-cyclic phosphodiesterase A	0.76
Estrogen receptor	0.685419277
Aldose reductase	0.444444444
Nitric oxide synthase, inducible	0.638356855
Tankyrase-2	0.833333333
Ornithine carbamoyltransferase, mitochondrial	0.55
Leukotriene A-4 hydrolase	1
SRSF protein kinase 2	0.485428051
Tyrosine-protein phosphatase non-receptor type 1	0.661350126
Amine oxidase [flavin-containing] B	0.616642336
Beta-lactamase	0.223333333
Proto-oncogene tyrosine-protein kinase receptor Ret	0.622794881
Acetylcholinesterase	1
Muscarinic acetylcholine receptor M2	0.624245169
Trypsin	0.925
Tissue-type plasminogen activator	0.501881712
Hepatocyte growth factor receptor	0.65935518
Transthyretin	0.75
Mitogen-activated protein kinase 14	0.568161326
Serine hydroxymethyltransferase, cytosolic	0.5
Neutral alpha-glucosidase AB	0.604166667
Nitric oxide synthase, endothelial	0.502841716
Hydroxyacylglutathione hydrolase, mitochondrial	1
Caspase-1	0.719152192
Peptidyl-prolyl cis-trans isomerase A	0.953265766

PROTEIN FAMILY	AUC
Ephrin type-B receptor 4	0.569070081
Collagenase 3	0.435503106
Epithelial discoidin domain-containing receptor 1	0.695374801
3-dehydroquinate dehydratase	0.944444444
Tyrosine-protein kinase ABL1	0.59311803
Dihydrofolate reductase	0.593622054
3-dehydroquinate dehydratase	0.944444444
Nitric oxide synthase, inducible	0.447877799
Plasmepsin-2	0.792768484
S-adenosylmethionine decarboxylase proenzyme	0.436363636
Peroxisome proliferator-activated receptor gamma	0.712760417
Serine/threonine-protein kinase 24	0.5
Epidermal growth factor receptor	0.72002039
Histone deacetylase 6	0.541666667
Mitogen-activated protein kinase 1	0.691074681
Dipeptidyl peptidase 4	0.719636382
Beta-lactamase	0.666666667
Fatty-acid amide hydrolase 1	0.603224395
Hepatocyte growth factor receptor	0.513960358
Renin	0.744436553
Serine/threonine-protein kinase MRCK alpha	0.49726776
Caspase-8	0.675824176
Carbonic anhydrase 4	0.827304712
cAMP-specific 3',5'-cyclic phosphodiesterase 4D	0.738927739
Acetylcholinesterase	0.693838114
Lethal factor	0.314685315
Beta-lactamase	0.200892857
Mitogen-activated protein kinase 15	0.617
Mu-type opioid receptor	0.577696533
Serine/threonine-protein kinase Nek2	0.585769231
Nitric oxide synthase, endothelial	0.410059676
Ephrin type-A receptor 2	0.708705357
Acetylcholinesterase	0.406666667
Complement C1s subcomponent	0.205163043
Dihydrofolate reductase	0.584614685
Orotidine 5'-phosphate decarboxylase	0.6
Leukotriene A-4 hydrolase	1
Phenylethanolamine N-methyltransferase	0.750091912
Serine/threonine-protein kinase Nek2	0.483461538
Nitric oxide synthase, inducible	0.641916583
Peroxisome proliferator-activated receptor gamma	0.752517361
Serum albumin	0.4375
Beta-lactamase OXA-10	1
Serum albumin	0.458333333
Serine/threonine-protein kinase Chk1	0.617900609
Serine/threonine-protein kinase PAK 7	0.534461153
cAMP-specific 3',5'-cyclic phosphodiesterase 4B	0.874056095
Galectin-9	0.591346154
RAC-beta serine/threonine-protein kinase	0.283819629
Orotidine 5'-phosphate decarboxylase	0.542857143
Endochitinase	0.666666667
Serine/threonine-protein kinase B-raf	0.578138528
1-deoxy-D-xylulose 5-phosphate reductoisomerase	0.416666667

PROTEIN FAMILY	AUC
Cathepsin L1	0.645216598
Maltase-glucoamylase, intestinal	0.3
Serine/threonine-protein kinase Nek2	0.465
Coagulation factor X	0.556495641
Death-associated protein kinase 1	0.404938272
Cathepsin B	0.593164794
Breast cancer type 1 susceptibility protein	0.125
Bifunctional purine biosynthesis protein PURH	0.467647059
3-phosphoinositide-dependent protein kinase 1	0.388235294
Proto-oncogene tyrosine-protein kinase Src	0.59015873
Glutathione S-transferase P	0.675
Vascular endothelial growth factor receptor 2	0.632401497
3-phosphoinositide-dependent protein kinase 1	0.358823529
Androgen receptor	0.645383224
Phosphorylase b kinase gamma catalytic chain, liver/testis isoform	0.809116809
cAMP-dependent protein kinase catalytic subunit alpha	0.43208977
MAP kinase-activated protein kinase 5	0.389705882
Serine/threonine-protein kinase pim-1	0.676267281
Estrogen receptor	0.715052801
Gamma-aminobutyric acid receptor subunit alpha-3	0.557800752
Breast cancer type 1 susceptibility protein	0.333333333
Queuine tRNA-ribosyltransferase	0.714285714
Sodium channel protein type 9 subunit alpha	0.49034749
Thyroid hormone receptor beta	0.089285714
Fibroblast growth factor receptor 2	0.594551282
Mitogen-activated protein kinase kinase kinase 2	0.505263158
Beta-2 adrenergic receptor	0.536683191
Cyclin-dependent kinase 2	0.359913441
Amine oxidase [flavin-containing] B	0.614890511
Serine/threonine-protein kinase MARK2	0.54047619
Caspase-3	0.88510101
Progesterone receptor	0.58618195
E3 ubiquitin-protein ligase XIAP	0.541580042
RAC-alpha serine/threonine-protein kinase	0.578437633
Macrophage colony-stimulating factor 1 receptor	0.397683398
STE20/SPS1-related proline-alanine-rich protein kinase	0.449588477
Peptidyl-prolyl cis-trans isomerase NIMA-interacting 1	0.646224146
Carbonic anhydrase 14	0.524003984
Glutamate receptor ionotropic, NMDA 2B	0.179675994
Aurora kinase C	0.623611111
Serine/threonine-protein kinase Sgk1	0.142857143
Mitogen-activated protein kinase 14	0.618272299
Peptidyl-prolyl cis-trans isomerase FKBP1A	0.759044839
Coagulation factor XI	0.294117647
Prothrombin	0.488319342
Integrin alpha-IIb	0.605990783
Glutathione S-transferase A1	0.5
Receptor-type tyrosine-protein phosphatase beta	0.575
AP2-associated protein kinase 1	0.552149401
Cyclin-dependent kinase 2	0.359770537
Mitogen-activated protein kinase 10	0.536525974
Dual specificity tyrosine-phosphorylation-regulated kinase 2	0.461580087
Mitogen-activated protein kinase 14	0.665556943

PROTEIN FAMILY	AUC
Proto-oncogene tyrosine-protein kinase Src	0.528034188
Transthyretin	0.946428571
Multidrug resistance-associated protein 1	0.40625
Heat shock protein HSP 90-alpha	0.879716981
Peptidyl-prolyl cis-trans isomerase A	0.934684685
Cationic trypsin	0.658107143
Nitric oxide synthase, endothelial	0.402387042
Androgen receptor	0.406992375
Serum albumin	0.409722222
Mitogen-activated protein kinase 4	0.319230769
Mu-type opioid receptor	0.556443192
Estrogen receptor	0.674903795
Ribonuclease pancreatic	0.413043478
Tyrosine-protein phosphatase non-receptor type 1	0.710547282
Protein kinase C iota type	0.270967742
Queuine tRNA-ribosyltransferase	0.642857143
Renin	0.774029356
Acetylcholinesterase	1
MAP kinase-activated protein kinase 2	0.728484848
Tyrosine-protein kinase ABL1	0.54341302
Beta-lactamase	0.204022989
Tyrosine-protein kinase ABL1	0.514792143
Misshapen-like kinase 1	0.570881226
Gastrin-releasing peptide receptor	0.815942029
MAP/microtubule affinity-regulating kinase 3	0.59704142
Prolyl endopeptidase	0.289772727
Growth factor receptor-bound protein 2	0.598484848
Cationic trypsin	0.669877345
Macrophage metalloelastase	0.393776261
Thymidylate synthase	0.644300144
Cathepsin L1	0.55624259
Serine/threonine-protein kinase DCLK3	0.499537465
Carboxypeptidase A1	0.184139785
Receptor-type tyrosine-protein phosphatase beta	0.625
E3 ubiquitin-protein ligase XIAP	0.555123217
Disintegrin and metalloproteinase domain-containing protein 17	0.559960201
Thermolysin	0.602162162
Glucosylceramidase	0.765507519
Neprilysin	0.451303155
Peptidyl-prolyl cis-trans isomerase A	0.915540541
Synaptic vesicular amine transporter	0.666666667
Cationic trypsin	0.659291406
Serum albumin	0.395833333
Estrogen receptor beta	0.698229614
Chymotrypsinogen A	0.595245446
Stromelysin-1	0.442525184
Thermolysin	0.577837838
Beta-secretase 1	0.850604363
Corticosteroid 11-beta-dehydrogenase isozyme 1	0.600649351
Leukotriene A-4 hydrolase	1
Nitric oxide synthase, endothelial	0.414038079
Cyclin-dependent kinase 5	0.475648075
Mitogen-activated protein kinase 14	0.664325843

PROTEIN FAMILY	AUC
Estrogen receptor beta	0.734680496
Beta-lactamase	0.666666667
cAMP-specific 3',5'-cyclic phosphodiesterase 4B	0.878910464
Bacterial leucyl aminopeptidase	0.303030303
Dihydrofolate reductase	0.173400673
Fructose-bisphosphate aldolase class 2	0.4
Beta-lactamase	0.230769231
Kinesin-like protein KIF11	0.798076923
Retinoic acid receptor beta	0.565338924
Aldose reductase	0.444444444
Ribonuclease pancreatic	0.350931677
Nitric oxide synthase, inducible	0.647555444
3-beta-hydroxysteroid-Delta(8),Delta(7)-isomerase	0.127819549
Carbonic anhydrase 2	0.767650754
Vascular endothelial growth factor receptor 2	0.637345847
Beta-lactamase OXA-10	1
Ephrin type-A receptor 3	0.50462963
Coagulation factor IX	0.391668328
Tankyrase-2	0.833333333
Uridine 5'-monophosphate synthase	0.35
Retinoic acid receptor alpha	0.748684211
Tyrosine-protein phosphatase non-receptor type 1	0.759009009
Serine/threonine-protein kinase Nek2	0.463846154
CREB-binding protein	0.666666667
Histamine H3 receptor	0.716155135
Serine/threonine-protein kinase Chk1	0.633333333
Cationic trypsin	0.67375
Nitric oxide synthase, endothelial	0.387752202
MAP kinase-interacting serine/threonine-protein kinase 2	0.552154195
Nuclear receptor subfamily 1 group I member 2	0
Phenylethanolamine N-methyltransferase	0.541544118
Prothrombin	0.599884859
Cationic trypsin	0.64229249
MAP kinase-activated protein kinase 5	0.754761905
cAMP-specific 3',5'-cyclic phosphodiesterase 4B	0.858144552
Dihydrofolate reductase	0.225146199
Cruzipain	0.988888889
Dehydrosqualene synthase	0.75
SRSF protein kinase 2	0.645491803
Lethal factor	0.318181818
Bifunctional dihydrofolate reductase-thymidylate synthase	0.486741835
Beta-secretase 1	0.830114976
Cationic trypsin	0.665304073
S-methyl-5'-thioadenosine phosphorylase	0.230769231
Cathepsin B	0.57911985
Tyrosine-protein kinase SYK	0.439705882
Beta-1,4-galactosyltransferase 1	1
Dihydrofolate reductase	0.520467836
Mineralocorticoid receptor	0.410922587
Serine/threonine-protein kinase BRSK1	0.631481481
Glutathione S-transferase Mu 1	0.3
Chymotrypsinogen A	0.722945205
Serine/threonine-protein kinase BRSK1	0.55

PROTEIN FAMILY	AUC
Bacterial leucyl aminopeptidase	0.303030303
Thymidine kinase, cytosolic	0.815384615
Cruzipain	0.977777778
Coagulation factor X	0.538375947
Cationic trypsin	0.671071429
Tyrosine-protein kinase ITK/TSK	0.588385683
Integrin alpha-IIb	0.581053698
Glycogen synthase kinase-3 beta	0.741623939
Epidermal growth factor receptor	0.72407839
Methionine aminopeptidase 2	0.529471545
Serine/threonine-protein kinase Nek2	0.429554656
Carbonic anhydrase 2	0.765748759
MAP kinase-activated protein kinase 2	0.64969697
Metallo-beta-lactamase L1	0.892857143
Mitogen-activated protein kinase 14	0.649587321
Serine/threonine-protein kinase LATS2	0.644457547
RAC-beta serine/threonine-protein kinase	0.44378838
Serine/threonine-protein kinase tousled-like 1	0.606060606
Tyrosine-protein kinase JAK2	0.562316285
Serum albumin	0.395833333
Cannabinoid receptor 2	0.494754161
Corticosteroid 11-beta-dehydrogenase isozyme 1	0.607892108
Thymidylate synthase	0.757575758
Testis-specific serine/threonine-protein kinase 1	0.491935484
Transthyretin	1
Tissue-type plasminogen activator	0.641949153
Nuclear receptor corepressor 2	0.166666667
Renin	0.776515152
Prothrombin	0.609844055
Beta-secretase 1	0.834537146
Steroid hormone receptor ERR1	1
Cyclin-dependent kinase-like 3	0.619883041
5-hydroxytryptamine receptor 4	0.536945813
Amine oxidase [flavin-containing] B	0.612116788
Dual specificity mitogen-activated protein kinase kinase 1	0.401239067
Fatty acid-binding protein, adipocyte	0.799342105
Neuraminidase	0.666666667
Estrogen receptor	0.674814301
Carbonic anhydrase 1	0.708262429
Dihydrofolate reductase	0.631289237
Proto-oncogene tyrosine-protein kinase Src	0.531282051
M-phase inducer phosphatase 3	0.571428571
Peroxisome proliferator-activated receptor delta	0.608465608
Beta-lactamase	0.23964497
Mitogen-activated protein kinase kinase kinase 3	0.493107105
Activated CDC42 kinase 1	0.567351326
Abelson tyrosine-protein kinase 2	0.625138427
Gamma-aminobutyric acid receptor subunit alpha-2	0.583703481
Transthyretin	0.5
Macrophage migration inhibitory factor	0.8125
Leukotriene B4 receptor 1	0.377862595
Proto-oncogene tyrosine-protein kinase Src	0.576459096
Cathepsin S	0.64196469

PROTEIN FAMILY	AUC
Muscarinic acetylcholine receptor M2	0.452341137
Growth factor receptor-bound protein 2	0.488636364
Beta-secretase 1	0.795622052
Ephrin type-B receptor 4	0.50606469
A disintegrin and metalloproteinase with thrombospondin motifs 1	1
Nuclear receptor corepressor 2	0.333333333
MAP kinase-activated protein kinase 5	0.619047619
Ribonuclease pancreatic	0.406832298
Dihydrofolate reductase	0.532163743
N(G),N(G)-dimethylarginine dimethylaminohydrolase 1	0.444444444
Transient receptor potential cation channel subfamily M member 6	0.131147541
Aldose reductase	0.638888889
Estrogen receptor	0.709202166
Peptidyl-prolyl cis-trans isomerase A	0.927364865
Dipeptidyl peptidase 4	0.719984104
Carboxypeptidase A1	0.671568627
Fatty-acid amide hydrolase 1	0.569399864
Protein kinase C eta type	0.386818496
Uridine 5'-monophosphate synthase	0.05
Cyclin-dependent kinase 2	0.358484403
Fibroblast growth factor receptor 2	0.663461538
Purine nucleoside phosphorylase	0.548513514
Cationic trypsin	0.660149633
Methionine aminopeptidase 2	0.518800813
Chymotrypsinogen A	0.690410959
Beta-secretase 1	0.763119104
Peroxisome proliferator-activated receptor gamma	0.770138889
Bifunctional dihydrofolate reductase-thymidylate synthase	0.558087092
Fibroblast growth factor 1	0.504273504
Caspase-3	0.948232323
Serum albumin	0.493055556
Macrophage metalloelastase	0.335346639
Protein arginine N-methyltransferase 1	0.5
Urokinase-type plasminogen activator	0.553044084
SRSF protein kinase 3	0.55068836
Calcium/calmodulin-dependent protein kinase type II subunit gamma	0.425420168
Leucine-rich repeat serine/threonine-protein kinase 2	0.447525343
Serine/threonine-protein kinase tousled-like 2	0.548964218
Caspase-8	0.181318681
Serine/threonine-protein kinase BRSK2	0.409166667
Mitogen-activated protein kinase 14	0.709616452
Dual specificity tyrosine-phosphorylation-regulated kinase 1A	0.625925926
Serine/threonine-protein kinase TBK1	0.5625
Protein-tyrosine kinase 6	0.643665158
cGMP-specific 3',5'-cyclic phosphodiesterase	0.896551724
Beta-lactamase OXA-10	1
Glutamate receptor ionotropic, kainate 1	0.223076923
Estrogen receptor beta	0.739985694
Corticosteroid 11-beta-dehydrogenase isozyme 1	0.616883117
3-phosphoshikimate 1-carboxyvinyltransferase	0.677777778
Caspase-3	0.820707071
Lanosterol 14-alpha demethylase	0.833333333
Cationic trypsin	0.674357143

PROTEIN FAMILY	AUC
Mitogen-activated protein kinase 14	0.698866001
UDP-3-O-[3-hydroxymyristoyl] N-acetylglucosamine deacetylase	0.3125
MAP kinase-activated protein kinase 2	0.684242424
Serine hydroxymethyltransferase, cytosolic	0.5
Melanocortin receptor 5	0.610080851
Tyrosine-protein phosphatase non-receptor type 1	0.640237176
A disintegrin and metalloproteinase with thrombospondin motifs 1	1
Somatostatin receptor type 2	0.714386602
Acetylcholinesterase	0.5
Macrophage metalloelastase	0.35727416
Serine/threonine-protein kinase PRP4 homolog	0.493710692
Androgen receptor	0.646910744
Lethal factor	0.437062937
Tyrosine-protein kinase ABL1	0.542014511
Insulin receptor-related protein	0.595467033
Kinesin-like protein KIF11	0.798076923
Mitogen-activated protein kinase kinase kinase 5	0.738970588
Caspase-3	0.86489899
Peptidyl-prolyl cis-trans isomerase A	0.935810811
Serine/threonine-protein kinase pim-1	0.749901251
Thyroid hormone receptor beta	0.170787546
Integrin alpha-L	0.510162602
Glutathione S-transferase P	0.675
Proto-oncogene tyrosine-protein kinase Src	0.611965812
Acetylcholinesterase	1
Prothrombin	0.639278752
Cathepsin B	0.605337079
Interleukin-1 receptor-associated kinase 1	0.567307692
cAMP-specific 3',5'-cyclic phosphodiesterase 4A	0.778195489
Receptor-type tyrosine-protein phosphatase epsilon	0.4
Aldose reductase	0.527777778
Cathepsin B	0.540730337
Aldose reductase	0.5
Peptidyl-prolyl cis-trans isomerase NIMA-interacting 1	0.753487253
Aldose reductase	0.472222222
Tyrosine-protein phosphatase non-receptor type 1	0.625589876
Corticosteroid 11-beta-dehydrogenase isozyme 1	0.615384615
Mitogen-activated protein kinase 11	0.645572917
Serum albumin	0.479166667
Glycogen phosphorylase, liver form	0.285714286
Caspase-1	0.716317163
Prothrombin	0.413938197
Fibroblast growth factor receptor 2	0.542438272
Estrogen receptor	0.682600233
Alpha-2A adrenergic receptor	0.367365976
Protein kinase C iota type	0.51147541
Estrogen receptor	0.759866655
Thermolysin	0.548108108
Fatty acid-binding protein, adipocyte	0.904605263
Serine/threonine-protein kinase Chk1	0.589460784
Serum albumin	0.506944444
Heat shock protein HSP 90-alpha	0.846698113
5-hydroxytryptamine receptor 3A	0.724780702

PROTEIN FAMILY	AUC
Methionine aminopeptidase 2	0.524136179
Carbonic anhydrase 2	0.758031625
Steroid hormone receptor ERR1	1
Thyroid hormone receptor beta	0.043956044
Fatty acid-binding protein, adipocyte	0.865131579
Fibroblast growth factor 1	0.826923077
Bifunctional dihydrofolate reductase-thymidylate synthase	0.526399689
3-dehydroquinate dehydratase	0.883333333
Serine/threonine-protein kinase MRCK alpha	0.633879781
Suppressor of tumorigenicity 14 protein	0.436170213
Queuine tRNA-ribosyltransferase	0.614285714
Epidermal growth factor receptor	0.697923729
Glutamate carboxypeptidase 2	0.436147186
Neutrophil elastase	0.473294107
E3 ubiquitin-protein ligase XIAP	0.57047817
Nitric oxide synthase, endothelial	0.401676613
Orotidine 5'-phosphate decarboxylase	0.528571429
Cyclin-dependent kinase 17	0.668402778
Prothrombin	0.462100548
Fatty acid-binding protein, adipocyte	0.779605263
Gamma-aminobutyric acid receptor subunit theta	0.333333333
Insulin receptor	0.684104628
Abelson tyrosine-protein kinase 2	0.532945736
Tyrosine-protein phosphatase non-receptor type 1	0.756281792
Calcium/calmodulin-dependent protein kinase type 1D	0.371069182
Mandelate racemase	0.273809524
Cathepsin G	0.44047619
Caspase-6	0.45
Dual specificity mitogen-activated protein kinase kinase 5	0.547325103
Estradiol 17-beta-dehydrogenase 1	0.666666667
Thymidylate kinase	0.75
Peptidyl-prolyl cis-trans isomerase NIMA-interacting 1	0.77970178
Purine nucleoside phosphorylase	0.539594595
Nitric oxide synthase, brain	0.511513158
Cationic trypsin	0.648164085
Peptidyl-prolyl cis-trans isomerase NIMA-interacting 1	0.727272727
Dual specificity mitogen-activated protein kinase kinase 5	0.529320988
Pseudolysin	0.647103659
Tyrosine-protein kinase ABL1	0.534903969
Dipeptidyl peptidase 4	0.727559485
Death-associated protein kinase 1	0.508024691
Orotidine 5'-phosphate decarboxylase	0.485714286
Death-associated protein kinase 1	0.65030303
Macrophage migration inhibitory factor	0.875
Peptidyl-prolyl cis-trans isomerase NIMA-interacting 1	0.765031265
Acetylcholinesterase	0.406666667
Peroxisome proliferator-activated receptor delta	0.764550265
3-hydroxy-3-methylglutaryl-coenzyme A reductase	0.713235294
Mast/stem cell growth factor receptor Kit	0.699658703
Growth factor receptor-bound protein 2	0.602272727
Fibroblast growth factor receptor 2	0.53433642
Nitric oxide synthase, inducible	0.647586946
Retinoic acid receptor RXR-alpha	0.513241011

PROTEIN FAMILY	AUC
E3 ubiquitin-protein ligase XIAP	0.535914761
Purine nucleoside phosphorylase	0.55812848
Collagenase 3	0.369590062
Carbonic anhydrase 2	0.76849109
Carbonic anhydrase 2	0.764121553
CREB-binding protein	0.555555556
Metabotropic glutamate receptor 5	0.673657254
Casein kinase II subunit alpha	0.452380952
1-deoxy-D-xylulose 5-phosphate reductoisomerase	0.1875
Heat shock protein HSP 90-alpha	0.891509434
Hepatocyte growth factor receptor	0.542675273
Receptor-type tyrosine-protein phosphatase beta	0.825
Mitogen-activated protein kinase 9	0.555555556
cAMP-dependent protein kinase catalytic subunit alpha	0.409501603
Hormonally up-regulated neu tumor-associated kinase	0.6114082
Dihydrofolate reductase	0.298976608
Fibroblast growth factor receptor 1	0.670486111
Gamma-aminobutyric acid receptor subunit alpha-2	0.508653784
Cationic trypsin	0.649719888
Dipeptidyl peptidase 4	0.692365009
Dihydrofolate reductase	0.595238095
Beta-1,4-galactosyltransferase 1	1
Glutamate receptor ionotropic, kainate 5	0.324175824
Papain	0.457037643
Adenosine receptor A2a	0.38771747
cAMP-specific 3',5'-cyclic phosphodiesterase 4B	0.849244876
Uridine 5'-monophosphate synthase	0.25
Coagulation factor X	0.571082827
Dual specificity mitogen-activated protein kinase kinase 5	0.576576577
STE20-like serine/threonine-protein kinase	0.543859649
Prothrombin	0.436943509
Mitogen-activated protein kinase 9	0.534738486
Disintegrin and metalloproteinase domain-containing protein 17	0.556608714
Integrin alpha-IIb	0.480798771
Serine/threonine-protein kinase VRK2	0.470175439
Liver carboxylesterase 1	0.407334087
Heat shock protein HSP 90-alpha	0.818396226
Alpha-galactosidase A	0.333333333
cAMP-specific 3',5'-cyclic phosphodiesterase 4A	0.744987469
Plasminogen	0.360488953
Lethal factor	0.522727273
Vascular endothelial growth factor receptor 3	0.715898826
Peroxisome proliferator-activated receptor delta	0.431216931
Prothrombin	0.57659492
Tryptase alpha/beta-1	0.201537433
Dihydrofolate reductase	0.385964912
Cathepsin K	0.503316662
Peptidyl-prolyl cis-trans isomerase A	0.905405405
Dipeptidyl peptidase 4	0.693010779
CREB-binding protein	0
Eukaryotic translation initiation factor 2-alpha kinase 4	0.468820862
Cyclin-dependent kinase 2	0.524926507
Fibroblast growth factor receptor 1	0.610416667

PROTEIN FAMILY	AUC
Renin	0.747514205
5'-methylthioadenosine/S-adenosylhomocysteine nucleosidase	0.162790698
Thymidylate synthase	0.584162521
5-hydroxytryptamine receptor 4	0.563537676
Serine/threonine-protein kinase B-raf	0.636267007
Corticosteroid 11-beta-dehydrogenase isozyme 1	0.607892108
Beta-secretase 1	0.777048939
S-ribosylhomocysteine lyase	0.837912088
Tankyrase-2	0.833333333
cAMP-specific 3',5'-cyclic phosphodiesterase 4D	0.683760684
Cationic trypsin	0.691165796
Fibroblast growth factor receptor 2	0.627003205
Fibroblast growth factor 1	0.555555556
Tyrosine-protein kinase ABL1	0.561427889
Estrogen receptor beta	0.760893538
Papain	0.500954719
M1 family aminopeptidase	0
Tyrosine-protein phosphatase non-receptor type 1	0.619752405
Serine/threonine-protein kinase Chk1	0.630578093
cAMP-specific 3',5'-cyclic phosphodiesterase 4C	0.638392857
Prostaglandin G/H synthase 1	0.7
Aurora kinase A	0.53872549
Aldose reductase	0.375
Androgen receptor	0.524134813
Glutathione S-transferase A1	0.833333333
Mitogen-activated protein kinase 10	0.518722944
Mitogen-activated protein kinase 10	0.555627706
Chymotrypsinogen A	0.707020548
Serum albumin	0.451388889
Activin receptor type-2A	0.386160714
Tyrosine-protein kinase Lck	0.674332567
C-X-C chemokine receptor type 3	0.838983051
Serine/threonine-protein kinase Nek2	0.550769231
Rho-associated protein kinase 1	0.408666667
Proto-oncogene tyrosine-protein kinase Src	0.503076923
Proto-oncogene tyrosine-protein kinase Src	0.60043956
Glutamate receptor 1	0.68221831
Alpha-2B adrenergic receptor	0.487525041
Tyrosine-protein kinase HCK	0.505523256
Ephrin type-A receptor 3	0.523765432
Progesterone receptor	0.621106891
Dual specificity protein kinase CLK1	0.535964912
Substance-K receptor	0.505733945
Dehydrosqualene synthase	0.791666667
Insulin-like growth factor 1 receptor	0.624040921
Trypsin	0.975
Cyclin-dependent kinase-like 5	0.667924528
Fructose-bisphosphate aldolase class 2	0.6
Ephrin type-A receptor 8	0.446453901
Dihydrofolate reductase	0.597766679
Fibroblast growth factor 1	0.572649573
Epithelial discoidin domain-containing receptor 1	0.507770008
3-phosphoshikimate 1-carboxyvinyltransferase	0.6

PROTEIN FAMILY	AUC
Tyrosine-protein kinase SYK	0.371616079
Estradiol 17-beta-dehydrogenase 1	0.5
Xaa-Pro dipeptidase	0.5
Beta-secretase 1	0.794737618
Caspase-1	0.687426874
Corticosteroid 11-beta-dehydrogenase isozyme 1	0.60014985
Integrin beta-2	0.67816092
Proto-oncogene tyrosine-protein kinase Src	0.601782662
Glutamate receptor ionotropic, NMDA 2B	0.466666667
Queuine tRNA-ribosyltransferase	0.7
Mast/stem cell growth factor receptor Kit	0.683665644
TRAF2 and NCK-interacting protein kinase	0.575396825
Insulin receptor	0.572183099
Mitogen-activated protein kinase 9	0.54265873
Dual specificity protein phosphatase 3	0.443452381
Potassium voltage-gated channel subfamily H member 2	0.671018442
Transthyretin	0.875
Calcium/calmodulin-dependent protein kinase type II subunit delta	0.545454545
Cyclin-dependent kinase 2	0.593826556
Epidermal growth factor receptor	0.701302966
Orotidine 5'-phosphate decarboxylase	0.535714286
Tyrosine-protein phosphatase non-receptor type 1	0.732533554
Neutrophil collagenase	0.670212766
Acetylcholinesterase	1
Serine/threonine-protein kinase DCLK3	0.342275671
Beta-lactamase	0.233516484
Dihydrofolate reductase	0.466374269
Dipeptidyl peptidase 1	0.5
Gamma-aminobutyric acid receptor subunit beta-1	0.488888889
Homeodomain-interacting protein kinase 2	0.517276423
Alpha-galactosidase A	0.666666667
Ribonuclease pancreatic	0.332298137
Tyrosine-protein phosphatase non-receptor type 1	0.759989581
Galectin-7	0.682758621
Dihydrofolate reductase	0.499269006
Heat shock protein HSP 90-alpha	0.880896226
Peroxisome proliferator-activated receptor gamma	0.802777778
Estrogen receptor	0.566148649
Neutrophil collagenase	0.631389887
Retinoic acid receptor alpha	0.607302632
Orotidine 5'-phosphate decarboxylase	0.471428571
Trifunctional purine biosynthetic protein adenosine-3	0.715460526
Sodium channel protein type 9 subunit alpha	0.252895753
Prostaglandin G/H synthase 1	0.5
Dual specificity protein kinase TTK	0.558781362
Estrogen receptor beta	0.759924893
Serine/threonine-protein kinase B-raf	0.675063776
Alpha-1A adrenergic receptor	0.77074853
Serum albumin	0.4375
Cytochrome P450 130	1
Coagulation factor X	0.542051613
Hepatocyte growth factor receptor	0.676663081
Growth hormone secretagogue receptor type 1	0.370967742

PROTEIN FAMILY	AUC
Cationic trypsin	0.662884178
Tyrosine-protein phosphatase non-receptor type 1	0.663510449
MAP kinase-activated protein kinase 2	0.60995086
Aurora kinase A	0.519837398
Dual specificity tyrosine-phosphorylation-regulated kinase 1B	0.420493197
Ornithine carbamoyltransferase, mitochondrial	0.55
Serum albumin	0.520833333
Prothrombin	0.544909893
Ephrin type-A receptor 3	0.540740741
Serum albumin	0.451388889
Poly [ADP-ribose] polymerase 1	0.58
Queuine tRNA-ribosyltransferase	0.642857143
Heat shock protein HSP 90-alpha	0.882075472
Serine/threonine-protein kinase Nek2	0.472469636
Proto-oncogene tyrosine-protein kinase receptor Ret	0.547350519
Aurora kinase A	0.567647059
Myosin light chain kinase, smooth muscle	0.457239057
D(4) dopamine receptor	0.562132862
Complement C1s subcomponent	0.326086957
Estrogen receptor	0.662307589
Serine/threonine-protein kinase 24	0.53874269
Inosine-5'-monophosphate dehydrogenase 1	0.7625
Plasmepsin-2	0.783459255
Cannabinoid receptor 1	0.599931773
Mitogen-activated protein kinase 14	0.635611735
5-hydroxytryptamine receptor 2B	0.453418404
Mitogen-activated protein kinase 8	0.440866511
5'-methylthioadenosine/S-adenosylhomocysteine nucleosidase	0.279069767
Queuine tRNA-ribosyltransferase	0.678571429
Disintegrin and metalloproteinase domain-containing protein 10	0.2625
Coagulation factor XI	0.673202614
Transthyretin	0.571428571
Tyrosine-protein phosphatase non-receptor type 1	0.63953239
Retinoic acid receptor RXR-alpha	0.605077745
Carboxypeptidase A1	0.247087814
Coagulation factor X	0.557639636
Serine/threonine-protein kinase tousled-like 1	0.528505393
Thymidylate synthase	0.705406556
Myosin light chain kinase, smooth muscle	0.532792208
Vascular endothelial growth factor receptor 3	0.676151762
Solute carrier family 22 member 6	0.666666667
Peroxisome proliferator-activated receptor gamma	0.840190972
Casein kinase II subunit beta	1
Lethal factor	0.585664336
Protein kinase C alpha type	0.450050093
Receptor-type tyrosine-protein phosphatase C	0.486111111
Thymidylate synthase	0.715091528
Cathepsin B	0.593164794
E3 ubiquitin-protein ligase XIAP	0.59506237
Tissue-type plasminogen activator	0.46923597
TRAF2 and NCK-interacting protein kinase	0.638888889
cAMP-dependent protein kinase catalytic subunit alpha	0.370300204
Mitogen-activated protein kinase 1	0.666211293

PROTEIN FAMILY	AUC
Serine/threonine-protein kinase ULK3	0.455741627
Fibroblast growth factor receptor 1	0.471786834
Cell division protein ZipA	0
Adenosine receptor A2b	0.492759933
Hepatocyte growth factor receptor	0.647595137
Protein S100-B	0
Melatonin receptor type 1A	0.654166667
Glutathione S-transferase A1	0.366666667
Neuronal acetylcholine receptor subunit alpha-3	0
Histone deacetylase 7	0.261904762
Glucocorticoid receptor	0.649296583
Serine/threonine-protein kinase MRCK alpha	0.674180328
Phosphatidylinositol 4-kinase beta	0.358767304
D(2) dopamine receptor	0.610324363
Dihydrofolate reductase	0.513157895
Interferon-induced, double-stranded RNA-activated protein kinase	0.562973485
RAC-alpha serine/threonine-protein kinase	0.601213282
Glutamate receptor 4	0.05625
Amyloid beta A4 protein	0.641198818
Epithelial discoidin domain-containing receptor 1	0.454545455
Aurora kinase B	0.657733355
Glycogen phosphorylase, brain form	0.721428571
Papain	0.405751238
Dual specificity tyrosine-phosphorylation-regulated kinase 2	0.409632035
Cholinesterase	0.806122967
Acetylcholinesterase	0.446666667
Retinoic acid receptor RXR-alpha	0.590500486
Lethal(3)malignant brain tumor-like protein 1	0.333333333
Papain	0.489496364
Neprilysin	0.43484225
Neprilysin	0.401577503
Death-associated protein kinase 1	0.583030303
Glutamate receptor 3	0.181818182
Nitric oxide synthase, endothelial	0.43677181
Hypoxanthine-guanine phosphoribosyltransferase	0.462284483
Serine/threonine-protein kinase Nek2	0.532307692
Estradiol 17-beta-dehydrogenase 1	0.666666667
Chymotrypsinogen A	0.685759494
Macrophage migration inhibitory factor	0.9375
Receptor-type tyrosine-protein phosphatase C	0.615740741
Cyclin-dependent kinase 2	0.337620447
Ribosyldihydronicotinamide dehydrogenase [quinone]	0.094444444
Tyrosine-protein kinase receptor UFO	0.541539634
Chymotrypsinogen A	0.632534247
Histamine H4 receptor	0.48005491
Glycogen phosphorylase, liver form	0
Vascular endothelial growth factor receptor 2	0.664111099
Transthyretin	0.678571429
Prothrombin	0.443161951
cAMP-specific 3',5'-cyclic phosphodiesterase 4D	0.665371665
Dipeptidyl peptidase 1	0.5
Rho-associated protein kinase 1	0.399833333
Serine/threonine-protein kinase MARK2	0.631141345

PROTEIN FAMILY	AUC
Beta-lactamase OXA-10	1
Thermolysin	0.551351351
Myosin-IIIb	0.461748634
Proto-oncogene tyrosine-protein kinase Src	0.626129426
Transthyretin	0.892857143
Glycogen synthase kinase-3 beta	0.569660537
Cyclin-dependent kinase 2	0.370508738
Serine/threonine-protein kinase Nek1	0.272108844
Fibroblast growth factor receptor 2	0.561698718
cAMP-specific 3',5'-cyclic phosphodiesterase 4D	0.638435638
Thymidylate synthase	0.592171717
Insulin-like growth factor 1 receptor	0.677310924
Peptidyl-prolyl cis-trans isomerase A	0.905405405
BMP-2-inducible protein kinase	0.497916667
Papain	0.487611096
Phosphatidylinositol 4-kinase alpha	0.612
Fibroblast growth factor receptor 2	0.487179487
Disintegrin and metalloproteinase domain-containing protein 17	0.58101173
D(2) dopamine receptor	0.636354665
Thermolysin	0.567567568
Arginase-1	0.271428571
Phosphatidylinositol 4,5-bisphosphate 3-kinase catalytic subunit gamma isoform	0.619252874
Peroxisome proliferator-activated receptor gamma	0.728125
Corticosteroid 11-beta-dehydrogenase isozyme 1	0.625124875
Pteridine reductase 1	0.670995671
Serum albumin	0.4375
Cathepsin L1	0.600698529
Androgen receptor	0.609761463
Cationic trypsin	0.661635488
Urokinase-type plasminogen activator	0.540669066
Tyrosine-protein kinase Lck	0.666213531
Trifunctional purine biosynthetic protein adenosine-3	0.730263158
Dihydrofolate reductase	0.562278228
Excitatory amino acid transporter 1	0.807692308
Calcium/calmodulin-dependent protein kinase type II subunit delta	0.448989899
Cruzipain	0.933333333
Kinesin-like protein KIF11	0.807692308
Heat shock protein HSP 90-alpha	0.875
Caspase-3	0.868686869
Arginase-1	0.271428571
Prostacyclin receptor	0.819676795
Cathepsin L2	0.181818182
Peroxisome proliferator-activated receptor alpha	0.24796748
Mitogen-activated protein kinase 14	0.625491021
Retinoic acid receptor beta	0.60290007
Amine oxidase [flavin-containing] B	0.597226277
Peptidyl-prolyl cis-trans isomerase NIMA-interacting 1	0.756132756
Beta-2 adrenergic receptor	0.568859847
Queuine tRNA-ribosyltransferase	0.685714286
Estrogen receptor beta	0.763873987
Tyrosine-protein kinase Lck	0.670232977
Serine/threonine-protein kinase PLK1	0.39763178
Nitric oxide synthase, inducible	0.627835181

PROTEIN FAMILY	AUC
LIM domain kinase 1	0.395588235
Ribonuclease pancreatic	0.288819876
Serine/threonine-protein kinase 35	0.422222222
Tyrosine-protein kinase ZAP-70	0.303240741
Nitric oxide synthase, endothelial	0.422705314
Retinoic acid receptor RXR-alpha	0.541423712
Dipeptidyl peptidase 4	0.699493319
Proto-oncogene tyrosine-protein kinase Src	0.506959707
Stromelysin-1	0.459495383
Galectin-9	0.877777778
Peroxisome proliferator-activated receptor delta	0.669312169
Thymidylate synthase	0.65928764
Alcohol dehydrogenase E chain	0.272222222
Alcohol dehydrogenase E chain	0.311111111
Tyrosine-protein kinase ABL1	0.571309211
Epidermal growth factor receptor	0.723707265
Leukotriene B4 receptor 1	0.394561069
Cathepsin K	0.492954286
Beta-secretase 1	0.799012382
Cationic trypsin	0.656734079
Plasmepsin-2	0.820966001
Peroxisome proliferator-activated receptor gamma	0.625173611
Serine/threonine-protein kinase MRCK alpha	0.581989247
Peptidyl-prolyl cis-trans isomerase A	0.890765766
Alcohol dehydrogenase class 4 mu/sigma chain	0.375
Cyclin-dependent kinase-like 2	0.523550725
Interleukin-1 receptor-associated kinase 4	0.726502732
Serine/threonine-protein kinase PLK1	0.35828877
Prothrombin	0.625295508
Acetylcholinesterase	1
Mineralocorticoid receptor	0.549575822
cGMP-specific 3',5'-cyclic phosphodiesterase	0.900656814
Alpha-1D adrenergic receptor	0.734378295
Beta-lactamase	0.206730769
Casein kinase I isoform gamma-3	0.403697997
Carbonic anhydrase 2	0.76521948
Caspase-8	0.065934066
Protein delta homolog 1	0.25
Vascular endothelial growth factor receptor 2	0.652515552
Neuraminidase	0.333333333
Breast cancer type 1 susceptibility protein	0.25
Tyrosine-protein kinase JAK3	0.482413793
Cationic trypsin	0.649642857
Myosin-IIIb	0.622759857
Tyrosine-protein kinase ABL1	0.540606061
Tyrosine-protein kinase BTK	0.536363636
Estrogen receptor beta	0.526630305
Serine/threonine-protein kinase Nek3	0.575862069
Multidrug resistance-associated protein 1	0.15625
Platelet-derived growth factor receptor alpha	0.488039867
Epidermal growth factor receptor	0.707722458
Nitric oxide synthase, inducible	0.634671119
Fatty acid-binding protein, adipocyte	0.819078947

PROTEIN FAMILY	AUC
Gamma-aminobutyric acid receptor subunit pi	0.333333333
Lethal(3)malignant brain tumor-like protein 1	0.291666667
Interferon-induced, double-stranded RNA-activated protein kinase	0.492424242
Receptor tyrosine-protein kinase erbB-3	0.472916667
Multidrug resistance-associated protein 1	0.84375
Tyrosine-protein kinase JAK3	0.568275862
Leukotriene A-4 hydrolase	1
Poly [ADP-ribose] polymerase 1	0.385918675
Cyclin-dependent kinase 2	0.336456802
Fatty acid-binding protein, adipocyte	0.944078947
Purine nucleoside phosphorylase	0.418243243
Serine/threonine-protein kinase Chk1	0.628676471
Acetylcholinesterase	0.406666667
Orotidine 5'-phosphate decarboxylase	0.464285714
Cationic trypsin	0.642857143
Lethal(3)malignant brain tumor-like protein 1	0.041666667
Cationic trypsin	0.643533011
Cyclin-dependent kinase 13	0.478609626
Epidermal growth factor receptor	0.725572034
Neuronal acetylcholine receptor subunit alpha-7	0.448773449
Serine/threonine-protein kinase pim-1	0.692560895
RAC-beta serine/threonine-protein kinase	0.541568257
Aldose reductase	0.583333333
Methionine aminopeptidase 1	0.887681159
Tyrosine-protein kinase Lck	0.668583142
Myosin-IIIb	0.379781421
5-hydroxytryptamine receptor 1B	0.538593276
Phenylethanolamine N-methyltransferase	0.736856618
Urokinase-type plasminogen activator	0.570030871
CREB-binding protein	0.333333333
Chymotrypsinogen A	0.729539867
cAMP-dependent protein kinase catalytic subunit alpha	0.526231419
Lethal(3)malignant brain tumor-like protein 1	0.166666667
Glutathione S-transferase P	0.925
Bone morphogenetic protein receptor type-2	0.493421053
Thymidylate synthase	0.629148629
Eukaryotic translation initiation factor 2-alpha kinase 4	0.402792696
